# Combined Therapy Sensitivity Index Based on a 13-Gene Signature Predicts Prognosis for IDH Wild-type and MGMT Promoter Unmethylated Glioblastoma Patients

**DOI:** 10.7150/jca.30614

**Published:** 2019-08-29

**Authors:** Ningrong Ye, Nian Jiang, Chengyuan Feng, Feiyifan Wang, Hanwen Zhang, Harrusin Xiao Bai, Li Yang, Yandong Su, Chunhai Huang, Siyi Wanggou, Xuejun Li

**Affiliations:** 1 Department of Neurosurgery, Xiangya Hospital, Central South University, Changsha, Hunan, China;; 2 Department of Radiology, Hospital of the University of Pennsylvania, Philadelphia, Pennsylvania;; 3 Department of Neurology, The Second Xiangya Hospital, Central South University, Changsha, Hunan, China.; 4 Department of Neurosurgery, The First Affiliated Hospital of Jishou University, Jishou, Hunan China.

**Keywords:** Glioblastoma, Gene signature, CTSI, Prognosis

## Abstract

Glioblastoma (GBM) is one of the lethal tumors with poor prognosis. However, prognostic prediction approaches need to be further explored. Therefore, we developed an evaluation system that could be used for prognostic prediction of GBM patients. Published mRNA expression datasets from The Cancer Genome Atlas (TCGA), Gene Expression Omnibus (GEO) and Chinese Glioma Genome Atlas (CGGA) were analyzed. Quantitative Realtime-PCR of signature genes and molecular aberrations of 178 Xiangya GBM patients were used for confirmation. Gene set enrichment analysis (GSEA) was performed for functional annotation. As a result, we established a 13-gene signature which named Combined Therapy Sensitivity Index (CTSI). Based on a cutoff point, we divided patients into high-risk group and low-risk group. Based on Kaplan-Meier analysis and multivariate Cox regression analysis, we found that patients in the high-risk group had a shorter overall survival time than patients in the low-risk group (p<0.001 in TCGA and CGGA datasets, p=0.047 in GSE4271 dataset, p=0.008 in Xiangya GBM cohort, HR: 1.65-3.42). By comparing the status of IDH mutation, TERT promoter mutation (TERTp-mut) and MGMT promoter methylation, CTSI was predictable in IDH wild-type (IDH-wt)/MGMT promoter unmethylated (MGMTp-unmeth) patients (p=0.037 in IDH-wt/TERTp-mut/MGMTp-unmeth subgroup, HR: 1.98; p=0.032 in IDH-wt/TERTp-wt/MGMTp-unmeth subgroup, HR: 2.09). Based on GESA, the Gene Ontology (GO) gene sets were enriched differently between CTSI high-risk and low-risk groups. Our results showed CTSI risk score can predict the prognosis of IDH-wt/MGMTp-unmeth GBM patients. Based on CTSI, combined with the status of IDH mutation, TERT promoter mutation and MGMT promoter methylation, a stepwise prognosis evaluation system which can provide precise prognosis prediction for GBM patients was established.

## Introduction

Glioblastoma (GBM) is one of the most common and lethal malignant primary brain tumors in adults with a median survival time of only 14 months. Less than 5% of the patients live more than 5 years after diagnosis 1, 2. The mainstay of treatment is maximal resection, followed by radiation and chemotherapy. Despite multimodality therapy, the outcomes are dismal 3, 4. Therefore, novel diagnostic/prognostic biomarkers and better therapeutic targets are urgently needed 5.

In the past two decades, IDH mutation, MGMT promoter methylation, TERT promoter mutation, RTK, PI3K and p53 pathways alterations, molecular subtype and G-CIMP phenotype were identified to play crucial roles in the pathogenesis of GBM and also be of prognostic value 6-17. These studies above made a better understanding of GBM's landscape and disrupted signaling pathways, and provided more practical pathological classifications and personalized treatment regimens 18-22. However, they can't fully cover all cases such as IDH-wt/MGMTp-unmeth patients. Therefore, unearthing novel prognostic biomarkers, molecular targets and molecular signatures, and illustrating their expression with molecular features of GBM, are meaningful for improving the outcome of GBM patients.

In this study, we analyzed gene expression profiles of GBM patients in TCGA dataset, CGGA dataset and GEO dataset, respectively. By using these datasets, we identified and validated 13 signature genes that have prognostic value and proposed a risk score index named as Combined Therapy Sensitivity Index, CTSI. We tested 178 GBM samples from Xiangya GBM cohort and identified the prognostic value of CTSI in IDH-wt/MGMTp-unmeth patients. As a result, we established a stepwise algorithm for outcome prediction of the all GBM patients from the worst to the best outcome.

## Results

### Identification of signature genes for CTSI risk scoring model

**Figure 1:**
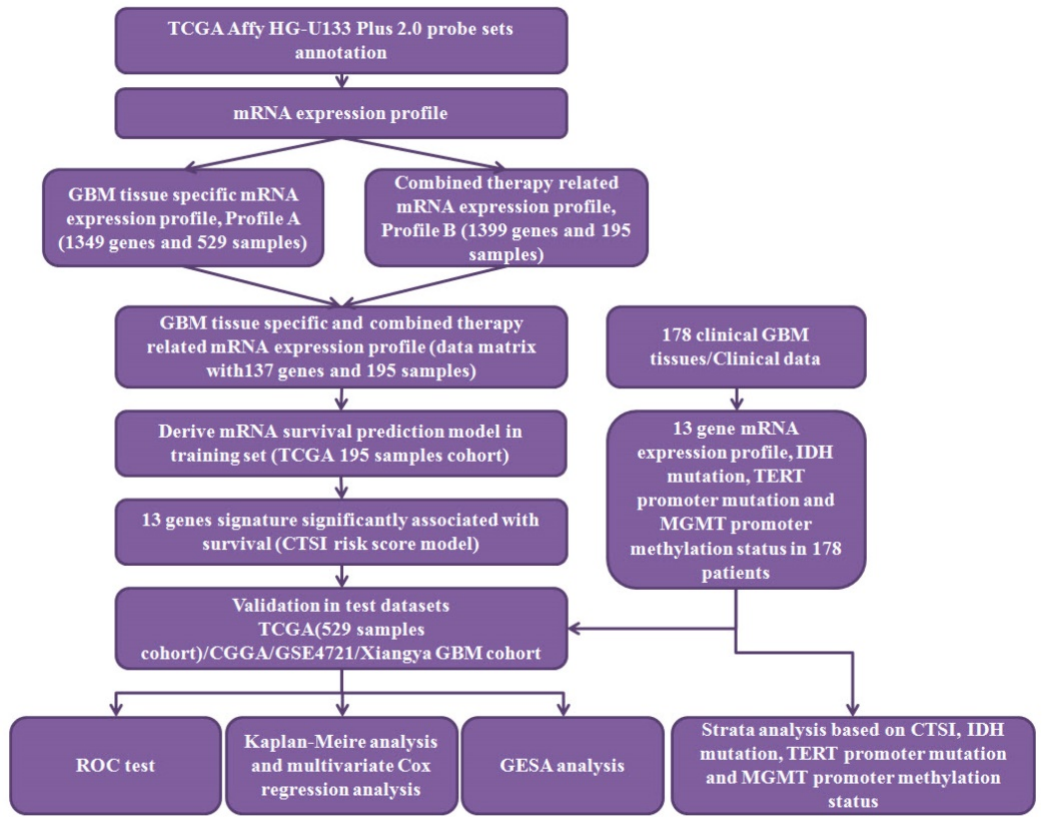
** The workflow of data processing.** 529 TCGA GBM patients' mRNA expression profile from Affymetrix HG-U133 Plus 2.0 was acquired from published database. By data filtering, a matrix with 137 genes and 195 samples was generated as training set to establish the CTSI risk score model. CGGA GBM dataset, GSE 4721 GBM dataset, TCGA 529 patient cohort and 178 Xiangya GBM cohort were used as validation sets. In Xiangya GBM cohort, TERT promoter mutation, MGMT promoter methylation and IDH mutation were sequenced for strata analysis.

**Figure 2:**
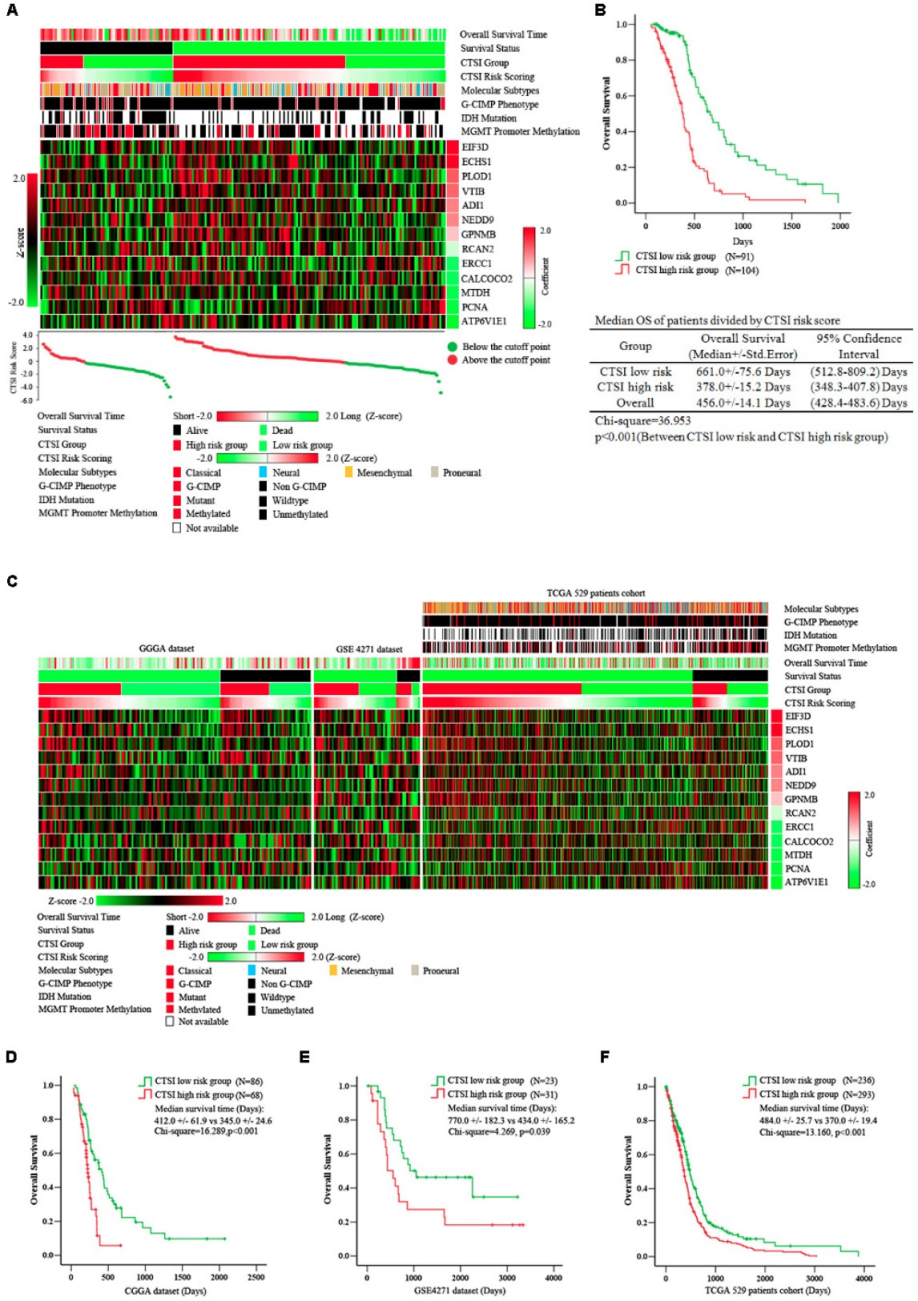
** Prognostic value of CTSI risk score. A.** Distribution of gene expression, CTSI risk score, overall survival and molecular features in TCGA 195 patient cohort. Overall survival time, CTSI risk scoring and expression of signature genes were converted into Z-score to regain the heatmap. Survival status, CTSI group, molecular subtypes, G-CIMP phenotypes, IDH mutation and MGMT promoter methylation were added for annotation. Signature genes were ranked by coefficients from multivariate Cox regression analysis. The CTSI risk score of each patient was plotted under the heatmap. **B.** Kaplan-Meier analysis for CTSI risk score model in TCGA 195 patient cohort. **C.** Distribution of gene expression, CTSI risk score, overall survival and molecular pathological features in CGGA dataset, GSE4271 dataset and TCGA 529 patient cohort. Kaplan-Meier analysis revealed the prognostic value of CTSI risk score model in CGGA dataset **(D)**, GSE4271 dataset **(E)** and TCGA 529 patient cohort **(F)**.

The workflow of data processing and data filtering is shown in Figure 1. 529 TCGA GBM patients' mRNA expression profile from Affymetrix HG-U133 Plus 2.0 was acquired from published database. First, we picked out 1349 GBM tissue specific genes (profile A) of 529 GBM patients comparing to normal brain tissue. Then we chose 195 patients who receive both radio- and chemotherapy and found out 1399 related gene (profile B). After filtering profile B in profile A, we discovered 137 GBM specific and radio-chemotherapy sensitivity correlated genes. We evaluated the prognostic impact of those genes in training set using univariate Cox regression analysis. Finally, 13 genes were identified as significantly associated with survival (*P*<0.01) (Supplementary Table S2). And their expression levels were shown in Figure 2A. All these13 genes were further analyzed using multivariable Cox regression analysis. Finally, we calculated a risk score by integrating the gene expression data and the corresponding coefficients derived from the multivariate Cox regression analysis. This risk scoring model was named as Combined Therapy Sensitivity Index (CTSI). The CTSI calculation formula is described as below:

CTSI risk score= (-1.18 × PCNA) + (1.84 × EIF3D) + (-1.93 × ATP6V1E1) + (1.52 × ECHS1) + (0.93 × PLOD1) + (-0.95 × ERCC1) + (0.72 × ADI1) + (-0.97 × CALCOCO2) + (0.70 × NEDD9) + (-1.09 × MTDH) + (-0.26 × RCAN2) + (0.34 × GPNMB) + (0.87 × VTI1B).

We calculated the CTSI risk score for each patient in the training set (TCGA 195 patient cohort) first. Then we ranked them according to their scores and calculated the cutoff point by maximizing Youden index through ROC analysis. As a result, -0.2248 was set as the cutoff point. Subsequently, the patients were divided into CTSI low-risk (n=91) and CTSI high-risk (n=104) groups (Supplementary Figure S1).

A significant difference in overall survival (OS) between the CTSI high-risk group and the low-risk group. The median OS in the high-risk group was 378.0 days versus 661.0 days in the low-risk group (p<0.001) (Figure 2B). We also performed multivariate Cox regression analysis to examine the independence of CTSI risk score on prognosis prediction. Clinical data including age, gender, KPS, extent of resection, G-CIMP phenotype and molecular subtype were used for multifactor analysis. The result showed that G-CIMP phenotype and CTSI risk score were both prognostic for GBM patients' prognosis. The coefficient of CTSI risk score was 1.26 with the hazard ratio (HR) at 3.54 (95% confidence interval: 2.26-5.46) (Table 1).

### Validation of CTSI risk score on survival prediction

To examine the reliability of CTSI on survival prediction, we analyzed the CGGA dataset (n=144), the GSE4271 dataset (n=54) and all GBM patients in the TCGA dataset (n=529) as validation dataset. Based on the CTSI cutoff point defined by the training dataset, patients in all these validation datasets were divided into CTSI high-risk group and CTSI low-risk group, respectively. The Kaplan-Meier survival analysis verified that patients in the low-risk group had better prognosis in each dataset (median OS, days, CTSI low-risk vs CTSI high-risk: 412.0+/-61.9 vs 345.0+/-24.6, p<0.001, in CGGA dataset; 770.0+/-182.3 vs 434.0+/-165.2, p=0.039, in GES4271 dataset; 484.0+/-25.7 vs 370.0+/-19.4, p<0.001, in TCGA 529 patients cohort) (Figure 2 C, D, E & F). More importantly, the multivariate Cox regression analysis confirmed that the CTSI risk score was a significant predictive factor. The hazard ratio of CTSI high-risk group ranged from 1.45 to 4.65 (Table 1).

### Evaluation of CTSI risk score performance

We performed receiver operating characteristic (ROC) analysis to examine the sensitivity and specificity of CTSI on prognosis prediction, using MGMT promoter methylation, IDH mutation and TERT promoter mutation as references. The area under the curve (AUC) of CTSI was 0.820 (p=0.004) (Supplementary Figure S2A).

### IDH mutation, MGMT promoter methylation, TERT promoter mutation and CTSI risk score

To further investigate the influence of MGMT promoter methylation, IDH mutation and TERT promoter mutation on prognostic value of CTSI, we performed subgroup analysis based on TCGA dataset. For IDH-wt patients, the high CTSI risk score was associated with the worse outcome (median OS, days, CTSI low-risk vs CTSI high-risk: 422.0+/-24.1 vs 357.0+/-20.3, p=0.012). However, for IDH-mut patients, the CTSI risk score cannot show prognostic value (Supplementary Figure S2B). For MGMTp-unmeth patients, CTSI low-risk score group showed a better prognosis (median OS, days, CTSI low-risk vs CTSI high-risk:485.0+/-34.7 days vs 362.0+/-23.0, p=0.013). However, for MGMTp-meth patients, there was no significant difference (Supplementary Figure S2C). For TERT promoter mutation, patients in the CTSI high-risk group had shorter overall survival time in both strata (median OS, days, CTSI low-risk vs CTSI high-risk: 484.0+/-45.2 vs 382.0+/-34.8, p=0.018, in TERTp-wt group; 485.0+/-46.7 vs 372.0+/-24.2, p=0.002, in TERTp-mut group) (Supplementary Figure S2D). These results demonstrated that CTSI risk score can predict the prognosis of IDH-wt/MGMTp-unmeth GBM patients.

### Combined CTSI risk score and TERT promoter mutation for prognosis prediction in IDH-wt and MGMTp-unmeth patients

**Figure 3:**
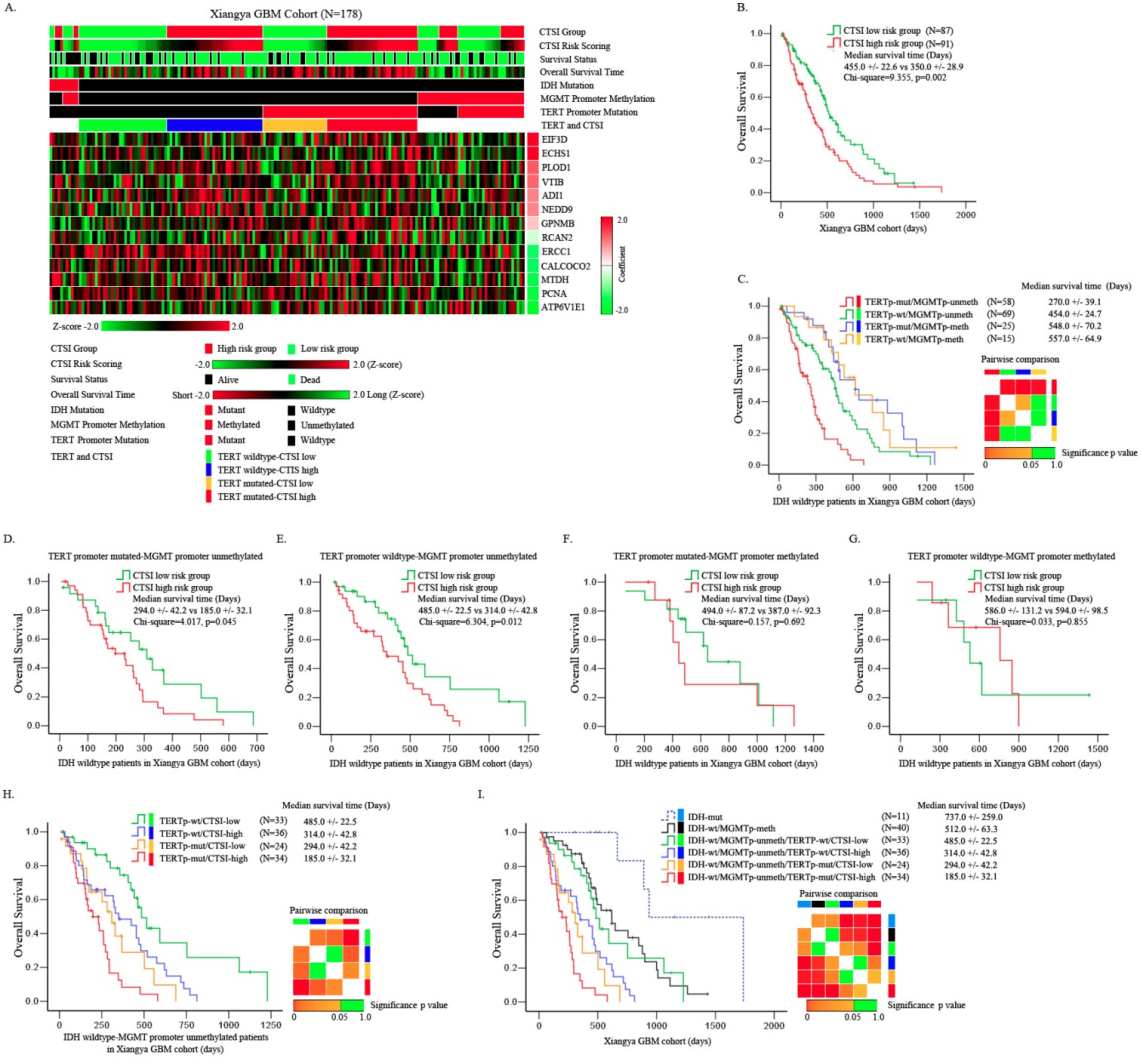
** Identification of prognostic value of CTSI risk score in IDH-wt/MGMTp-unmeth patients. A.** Distribution of gene expression, CTSI risk score, overall survival, IDH mutation, MGMT promoter methylation and TERT promoter mutation in Xiangya GBM cohort. **B.** Overall survival of the two groups according to CTSI risk score. **C.** Pairwise comparison of subgroups' overall survival according to TERT promoter mutation and MGMT promoter methylation for IDH-wt GBM patients. TERT promoter mutation was a prognostic factor for MGMTp-unmeth patients, but not for MGMTp-meth patients. **D.** Overall survival of CTSI in IDH-wt/TERTp-mut/MGMTp-unmeth patients.** E.** Overall survival of CTSI in IDH-wt/TERTp-wt/MGMTp-unmeth patients. **F.** Overall survival of CTSI in IDH-wt/TERTp-mut/MGMTp-meth patients.** G.** Overall survival of CTSI in IDH-wt/TERTp-wt/MGMTp-meth patients. **H.** Pairwise comparison of subgroups' overall survival based on TERT promoter mutation and CTSI in IDH-wt/MGMTp-unmeth patients. CTSI was a distinct prognostic factor in both the TERTp-wt and TERTp-mut subgroups. **I.** Survival of the six subgroups combining IDH mutation, TERT promoter mutation, MGMT promoter methylation and CTSI risk score together. IDH-mut patients received the best outcomes and IDH-wt/MGMTp-unmeth/TERTp-mut/CTSI-high patients received the worst.

For further validation, we performed quantitative real-time PCR for the 13 signature genes and detected the status of IDH mutation, TERT promoter mutation and MGMT promoter methylation in 178 Xiangya GBM patients. Compared with normal brain tissue, PCNA, CALCOCO2, ADI1, GPNMB, MTDH, EIF3D, NEDD9, ERCC1 and PLOD1 were up-regulated while ATP6V1E1, VTI1B, ECHS1 and RCAN2 were down-regulated in GBM patients (Supplementary Figure S3). The mutation rate of IDH was only 6.2%. Most of the GBM patients showed an IDH-wt phenotype. The TERT promoter had a mutation rate of 46.6%. The methylation rate of MGMT promoter was 25.8% (Supplementary Table S3). The mRNA expression of these 13 genes are transferred into z score and plotted in heatmap as shown in Figure 3A.

We validated the prognostic value of CTSI risk score in all patients of Xiangya GBM cohort. The median OS of CTSI high-risk group was 350.0+/-22.6 days versus 455.0 +/- 22.6 days in CTSI low-risk group (p=0.002) (Figure 3B). CTSI was a significant prognostic factor on multivariate Cox regression analysis with a hazard ratio of 1.68 for CTSI high-risk group (Table 1).

For IDH-wt patients, depending on TERT promoter mutation and MGMT promoter methylation together, we divided these patients into four subgroups: TERT promoter mutated (TERTp-mut)/MGMTp-unmeth, TERT promoter wild-type (TERTp-wt)/MGMTp-unmeth, TERTp-mut/MGMTp-meth and TERTp-wt/MGMTp-meth. The Kaplan-Meier analysis showed that the pronosis of TERTp-mut/MGMTp-unmeth subgroup was the worst, the TERTp-wt/MGMTp-unmeth subgroup was moderate while other two subgroups were relatively good (Figure 3C). Pairwise comparison between each subgroup showed that for MGMTp-meth patients, the status of TERT mutation didn't affect the OS (Figure 3C). These results were similar as reported by Hideyuki Arita et al. previously 23.

Next, we tested the prognostic value of CTSI in each of the four subgroups. We found that in both TERTp-mut/MGMTp-unmeth and TERTp-wt/MGMTp-unmeth subgroups, CTSI was an independent prognostic factor on Kaplan-Meier analysis (median OS, CTSI low-risk vs CTSI high-risk: 294.0 +/-42.2 vs 185.0+/-32.1 days, p=0.045, in TERTp-mut/MGMTp-unmeth subgroup; 485.0+/-22.5 vs 314.0+/-42.8 days, p=0.012, in TERTp-wt/MGMTp-unmeth subgroup) and multivariate Cox regression analysis (CTSI low-risk vs CTSI high-risk: p=0.037, HR=1.98, in TERTp-mut/MGMTp-unmeth subgroup; p=0.032, HR=2.09, in TERTp-wt/MGMTp-unmeth subgroup) (Figure 3D,E, Table 2 and Table 3). For TERTp-mut/MGMTp-meth and TERT-wt/MGMTp-unmeth subgroups, CTSI risk score was not a significant prognostic factor (Figure 3F and Figure 3G).

Then we combined CTSI and TERT promoter mutation in IDH-wt/MGMTp-unmeth patients and divided these patients into four subgroups: TERTp-mut/CTSI-low, TERTp-mut /CTSI-high, TERTp-wt/CTSI-low and TERTp-wt/CTSI-high. The TERTp-wt/CTSI-low subgroup had the best outcome, while the TERTp-mut/CTSI-high subgroup suffered the worst outcome (Figure 3H).

Finally, combing IDH mutation, MGMT promoter methylation, TERT promoter mutation and CTSI risk score together, we made pairwise comparisons among these subgroups. We found that the IDH-mut patients had the best outcome followed by IDH-wt/MGMTp-meth patients, IDH-wt/MGMTp-meth patients, IDH-wt/MGMTp-unmeth/TERTp-wt/CTSI-low patients, IDH-wt/MGMTp-unmeth/TERTp-wt/CTSI-high patients and IDH-wt/MGMTp-unmeth/TERTp-mut/CTSI-low patients. IDH-wt/MGMTp-unmeth/TERTp-mut/CTSI-high patients had the worst outcome (Figure 3I).

### Functional annotation of CTSI phenotypes

For Gene Set Enrichment Analysis, tumors were classified into CTSI high-risk and CTSI low-risk phenotypes. According to GO gene sets, 1842 out of 4058 gene sets were enriched in the CTSI high risk phenotype, while others were enriched in CTSI low-risk phenotype. 674 gene sets in the CTSI high-risk phenotype and 369 gene sets in the CTSI low-risk phenotype were significantly enriched (*P*<0.05). Many differences were unearthed between two phenotypes on Go biological process, GO cellular component and GO molecular function. Detailed information including plot of p-value vs NES, global ES histogram, heat map and gene list correlation profiles were exhibit exhibited in Supplementary Figure S4.

**Figure 4:**
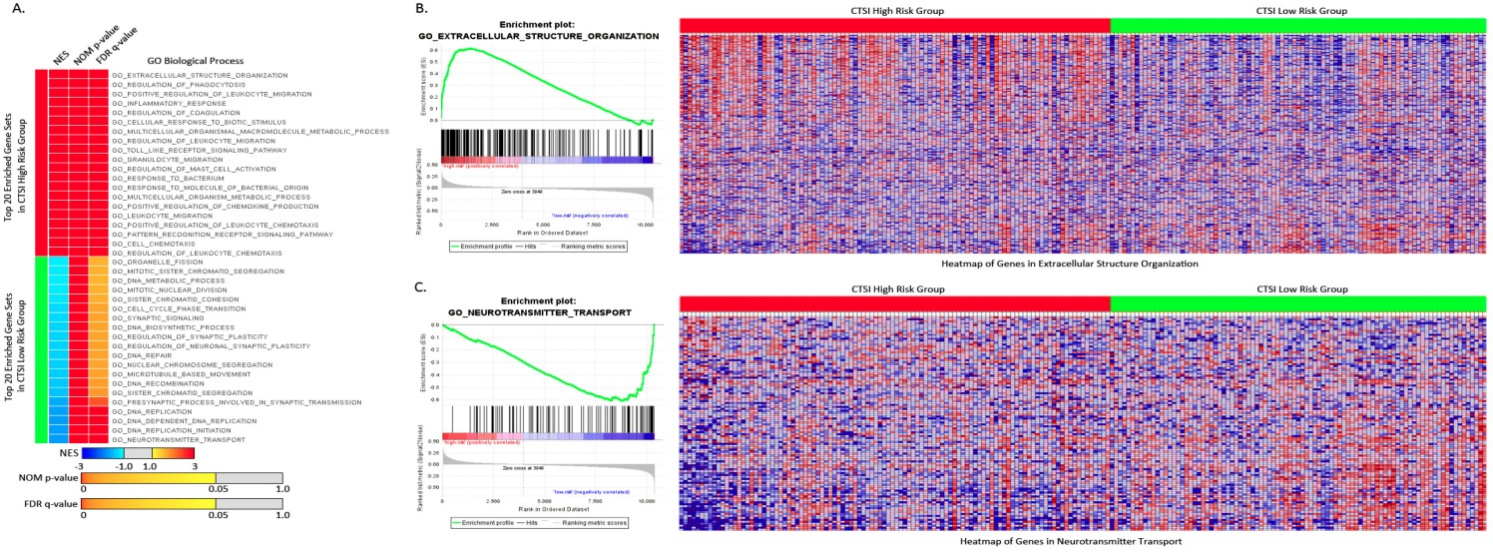
** Enriched gene sets of GO biological process based on both CTSI risk score phenotypes. A.** Top 20 up-regulated gene sets of GO biological process in both CTSI high-risk phenotype and CTSI low-risk phenotype. EXTRACELLULAR STRUCTURE ORGANIZATION was with the highest normalized enrichment scores (NES) in CTSI high-risk phenotype. NEUROTRANSMITTER TRANSPORT was the most enriched gene set in CTSI low-risk phenotype. Statistics as NES, nominal p-value (NOM p-value) and false discovery rate q-value (FDR q-value) were also shown. **B.** GSEA enriched profile and heatmap of enriched EXTRACELLULAR STRUCTURE ORGANIZATION in CTSI high-risk score phenotype.** C.** GSEA enriched profile and heatmap of enriched NEUROTRANSMITTER TRANSPORT in CTSI low-risk score phenotype.

For GO biological process, the top 20 enriched gene sets were shown in Figure 4A. The extracellular structure organization was most enriched process in CTSI high-risk group and neurotransmitter transport was most enriched process in CTSI low-risk group (Figure 4B and Figure 4C). Interestingly, CTSI high-risk phenotype show intense immunomodulatory including regulation of phagocytosis, inflammatory response, regulation of leukocyte migration, toll like receptor signaling pathway, granulocyte migration, regulation of mast cell activation, and so on (Supplementary Table S4). Besides inflammatory, CTSI high-risk phenotype also tend to closely communicate to environment such as extracellular structure organization, cellular response to biotic stimulus, multicellular organismal macromolecule metabolic process. Meanwhile, CTSI low-risk phenotype was found to related to neurobiological activity including neurotransmitter transport, presynaptic process involved in synaptic transmission, and cell proliferation including DNA biosynthetic process, DNA replication initiation, DNA repair, DNA recombination, microtubule based movement, sister chromatid segregation, cell cycle phase transition, and so on. The biological process of CTSI high- risk and low-risk phenotype reveal different function features, that is CTSI high-risk phenotype were prone to react to extracellular environment while low-risk phenotype tended to focus on cell activity especially proliferation. This can also be applied to GO cellular component and GO molecular function. As to component function, CTSI high-risk phenotype also tended to show high enrichment in function related to extracellular support structure, such as extracellular matrix component, basement membrane, external side of plasma membrane, protein complex involved in cell adhesion., while CTSI low-risk phenotype was more likely to show to intracellular structures such as condensed chromosome, chromosomal region, microtubule organizing center part. Referring to molecular function, CTSI high- risk phenotype was enriched in extracellular signal delivery such as integrin binding, cytokine receptor activity, growth factor binding, while low-risk phenotype was enriched intracellular activities such as microtubule motor activity, tubulin binding, DNA helicase activity (Supplementary Figure S5).

**Figure 5:**
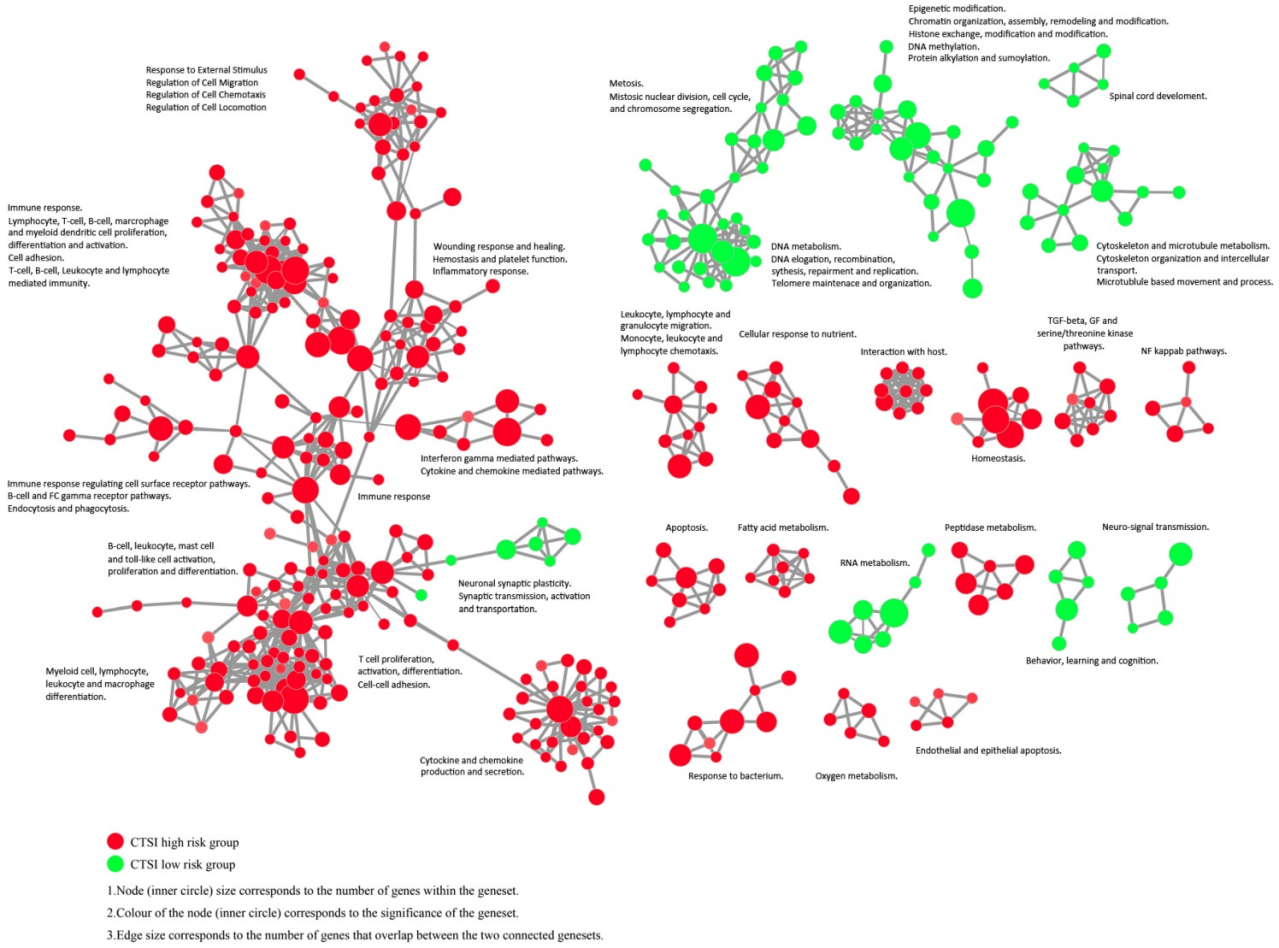
** Enrichment map contrasting both CTSI risk score phenotypes.** The gene set networks illustrated the results of GSEA targeted GO biological processes contrasting CTSI high-risk and CTSI low-risk phenotypes. Each node represents a gene set. Links between nodes represented the genes shared by both gene sets (filtered with p<0.05, FDR<0.05, Jaccard coefficient <0.95). The node color represented the strength and direction of enrichment (red gene sets were enriched in CTSI high-risk phenotype, green ones were enriched in CTSI low-risk phenotype). The figure was made by the Enrichment Map from Cytoscape 3.2.

The enrichment map revealed that CTSI high-risk phenotype were active to environment response which may contribute to immune escape while CTSI low-risk phenotype was more excited in cancer cell proliferation including mitosis, DNA and RNA metabolism, epigenetic modification, and cytoskeleton/microtubule metabolism (Figure 5 and Supplementary Figure S6).

## Discussion

As the most common primary intracranial malignancy, GBM is revealed as a molecularly heterogeneous disease. Recent studies through novel molecular platforms have provided molecular classifications of this tumor. In this study, based on transcriptome profile, we established a risk score model based on a 13-gene signature and validated its prognostic value by using differently published datasets and one retrospective clinical cohort from our local institution. Our findings of CTSI risk score provided a novel approach for prognosis evaluation of GBM patients and can serve as an important addition to the contemporary GBM prognosis evaluation system.

Recently, several gene signatures have been shown to be associated with the prognosis of glioma patients. Zhang et al. discovered that GPR85, SHOX2 and HMBOX1 had both diagnostic and prognostic values for patients with anaplastic glioma 24. Wang et al. reported a 3-gene signature (FPR3, IKBIP and S100A9) for prognostic evaluation of MGMTp-meth GBM patients 25. Compared with Wang's study, CTSI also provided good prognostic prediction of IDH-wt/MGMTp-unmeth patients. Consequently, it serves good complement to the 3-gene signature by Wang et al. to portrait the overall prognosis prediction map for GBM patients.

Out of the 13 signature genes, ATP6V1E1, EIF3D, ERCC1, GPNMB, MTDH, PCNA and NEDD9 have been reported to play crucial roles in various pathways and mechanism of glioma. Some of them were associated with prognosis 26-32. Other genes, such as CALCOCO2, RCAN2, ADI1, ECHS1, PLOD1 and VTI1B were associated with tumorigenesis or angiogenesis in some other cancers 33-35. Our study is the first one to report the involvement of those genes in GBM patients.

Gene set enrichment analysis revealed important genetic differences between the CTSI high-risk group and the CTSI low-risk group in our study. We found more than 1000 signaling pathways and biological processes that were differentially enriched in the two phenotypes. Gene sets related to cell cycle, mitosis, mRNA regulation, transcription, translation, DNA replication, DNA repair, neural biological processes and neural development were enriched in CTSI low-risk phenotype, while gene sets enriched in CTSI high-risk phenotype were mainly involved in immune responses, cell surface interactions, extracellular matrix, cell junction, cellular responses, apoptosis and energy metabolism. These genetic differences may offer some insights into the reason why GBM patients can have drastically different prognosis.

IDH mutation, MGMT promoter methylation and TERT promoter mutation are widely recognized as prognostic biomarkers in GBM patients. Patients with IDH mutation or wild-type TERT promoter have better outcomes compared with their counterparts 9,14. Patients with MGMT promoter methylation are more sensitive to temozolomide therapy 36. According to our analysis, CTSI risk score was unable to predict the prognosis of IDH-mut patients and MGMTp-meth patients. This indicates that the prognostic difference of CTSI risk score for overall GBM patients was majorly contributed by IDH-wt and MGMTp-unmeth patients. On the other hand, for GBM patients, TERT promoter mutation was predictive only in IDH-wt/MGMTp-unmeth subgroup. This finding agreed with another study focusing on TERT promoter mutation and MGMT promoter methylation for GBM patients 23. It suggests that the prognostic value of TERT promoter mutation is also majorly contributed by IDH-wt and MGMTp-unmeth patients. CTSI was a significant prognostic factor for patients with TERT promoter mutation. Based on these results, we suggest CTSI is a significant prognostic indicator for IDH-wt/MGMTp-unmeth GBM patients. And combined with TERT promoter mutation, CTSI is capable of further predicting the prognosis of GBM patients.

**Figure 6:**
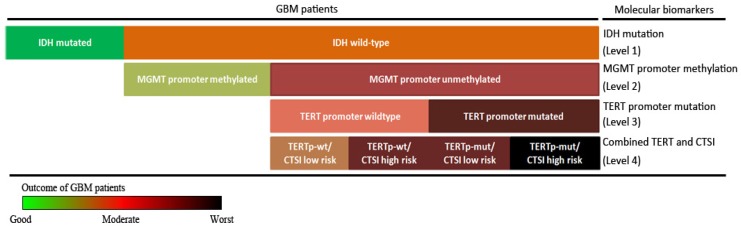
** Stepwise strategy for GBM prognosis evaluation.** The stepwise GBM prognosis evaluation system combined IDH mutation, MGMT promoter methylation, TERT promoter mutation and CTSI together. The color changed from green to black representing the patients' outcome from the good to the worst.

Finally, combining IDH mutation, TERT promoter mutation, MGMT promoter methylation with CTSI, we established a stepwise 4-level strategy for GBM prognosis evaluation (Figure 6). First, IDH mutation is the most important factor to divide GBM patients into those with good outcomes (IDH-mut) and bad outcomes (IDH-wt) (level 1). Second, for IDH-wt patients, MGMTp-meth patients have a better prognosis than MGMTp-unmeth patients (level 2). Third, TERT promoter mutation can predict outcome in MGMTp-unmeth patients, but not in MGMTp-meth patients (level 3). Finally, for IDH-wt/MGMTp-unmeth patients, CTSI is an important predictive factor and together with TERT promoter mutation, the prognosis of GBM patients can be more precisely predicted (level 4).

In conclusion, CTSI risk score is a predictable factor for IDH-wt/MGMTp-unmeth GBM patients. Based on CTSI, IDH mutation, TERT promoter mutation and MGMT promoter methylation, a 4-level stepwise prognosis evaluation system providing more precise outcome prediction for GBM patients was established.

## Materials and Methods

### Gene expression datasets and clinical data

GBM gene expression profiles and corresponding clinical data were obtained from three public databases: the TCGA dataset (http://cancergenome.nih.gov), the GEO GSE4271 dataset (https://www.ncbi.nlm.nih.gov/geo/query/acc.cgi?acc=GSE4271) and the CGGA dataset (http://cgga.org.cn). All these datasets were generated on Affymetrix platform HG-U133a.

Based on TCGA dataset, we selected 195 patients received post-operationally combined radio-chemotherapy to regain a new cohort, TCGA 195 patient cohort, as training set to identify the gene expression signature. This dataset is a subset of TCGA dataset, and the data was also generated on Affymetrix platform HG-U133a. On the other hand, the TCGA 529 patient cohort, GSE4271 dataset (54 patients) and CGGA dataset (144 patients) were included as validation sets.

### Microarray data processing

The Robust Multichip Average (RMA) algorithm was used for background adjustment 37. The probes (or probe sets) from Affymetrix HG-U133a were re-mapped to the human genome (GRCh38) using hgu133a.db (R/Bioconductor package). Multiple probes (or probe sets) mapping to the same gene were averaged using the mean values of those probes (or probe sets) to generate a single expression value (on the log2 scale). Two differential gene expression profiles from the TCGA dataset were created. Profile A was the differential gene expression profile between GBM and normal brain tissues. Profile B was generated as the following: 1. We used the median survival time as the cutoff point, and divided the 195 patient cohort into the radio-chemotherapy resistant group (n=85, median: 44.6 months) and the radio-chemotherapy sensitive group (n=110, median: 79.8 months). 2. By comparing gene expression patterns between the two groups, a differential gene expression profile was generated. As a result, Profile A contained 1349 genes and Profile B contained 1399 genes. Finally, we intersected Profile A with Profile B and a new data matrix containing 137 genes and 195 samples emerged. These 137 genes were GBM tissue-specific and associated with radio-chemotherapy sensitivity. The workflow of data processing was shown in Supplementary Figure S1.

### Risk scoring model

We performed univariable Cox regression analysis to evaluate the relationship between the expression levels of each of the 137 genes and the patient's overall survival (OS) time in the training dataset. Multivariable Cox regression analysis was performed on those genes in the training dataset, with OS as the dependent variable and other clinical information as the covariables. The genes significantly associated with survival (p<0.01) were used to create a risk scoring model for prognosis prediction. The risk scoring model, which we named Combined Therapy Sensitivity Index, CTSI, was defined as a linear combination of the expression values of the prognostic genes and the multivariable Cox regression coefficients as the weight. The risk score was calculated as previously described 24, 25, 38-41:


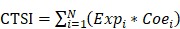
.

In this formula, N stands for the number of prognostic genes. *Exp_i_* is the relative expression level of gene*_i_*. *Coe_i_* is the estimated regression coefficient of *gene_i_* in the multivariable Cox regression analysis.

### Tissue samples, patients' information and follow-up

To further validate the CTSI risk scoring system, we collected 178 GBM surgical specimens from the tissue archive in our hospital. Those patients were admitted between Oct. 2008 - Nov. 2015. Age-matched normal brain tissues were obtained from patients with severe brain trauma. The expression of those 13 signature genes were quantified by real-time RT-PCR. Post-therapy MR images of the brain was performed to evaluate the response to the recurrence of tumors at 1-year intervals for follow-up. Data on death were defined as failure events for the overall survival rate. All follow-ups in this study ended in May 2016. The study was approved by the Committee on Medicine Ethics of Xiangya Hospital of Central South University. All patients were well informed and written informed consent was provided.

### RT-PCR and Quantitative real-time PCR

The RT-PCR and quantitative real-time PCR were performed as previously described 42. Briefly, total RNA isolation (Invitrogen), first strand cDNA synthesis (Fermentas) and qPCR (SYBR-Premix Ex TaqTM kit; Takara) were performed according to manufacturer's protocol. Thirty-five cycles were conducted on the ABI PRISM_ 7900 HT. Reported values were calculated using the 2-ΔΔCt method, normalized against endogenous GAPDH. Quantitative real-time PCR was conducted in triplicate for each sample. For data analysis, gene expression in normal brain tissue was used as reference to calculate relative expression level. Primer sequences were shown in Supplementary Table S1.

### IDH mutation, TERT promoter mutation and MGMT promoter methylation

Genomic DNA from 178 GBM tissue samples was isolated by using the QIAamp DNA mini kit (Qiagen) according to the manufacturer's instructions.

IDH1 and IDH2 mutation analysis were performed using previously described methods by pyrosequencing 43, 44. The regions spanning wild-type R132 of IDH1 and wild-type R172 of IDH2 were amplified by PCR. The PCR analysis was performed in 25 ul reaction volume, containing 0.2 uM each forward and reverse primer, 1×buffer, 2.0 mM dNTPs, 0.04 U of HotstartTaq (Qiagen), 1 mM MgCl_2_ and 2 ml of 10-20 ng DNA. The PCR conditions were as follows: 95°C for 9 minutes; 45 cycles of 95°C for 15 seconds, 56°C for 25 seconds, 72°C for 1minute (ABI PCR system 9700). DNA was purified and subjected to pyrosequencing on PyroMark Q96 ID System (QIAGEN). IDH mutated means that the subject was IDH1 or IDH2 mutated. All primers for IDH1 and IDH2 mutation analysis are listed in Supplementary Table S1b.

We used Sanger sequencing to determine the TERT promoter mutational status as described by Eckel-Passow JE and Lachance DH 14. Sequences covering C228T and C250T mutations in TERT promoter was amplified, which yield a 244-bp product. A total volume of 20 ul reaction mixture was prepared for PCR, consisting of 10-100ng DNA in solution, Platinum Taq DNA polymerase (1.5 unit), 0.2mM dNTPs, 0.25mM for each primer and 0.5X PCR Enhancer. The first PCR cycling conditions were set at 95°C for 2 minutes, 40 cycles at 95°C for 15 seconds, 62°C for 20 seconds and 72 °C for 10 minutes.1 uL of the amplified DNA from the first PCR was used for second PCR in a 20 uL reaction mixture, containing 0.5×PCR Enhancer, 10×dNTP mix that contained 1.5 mM dGTP, 0.5mM deaza-GTP (Sigma-Aldrich) and 0.5mM primers. The reaction cycling condition of was the same with the first PCR. Products of second PCR were purified and subjected to Sanger sequencing by using the BigDye Terminator cycle sequencing kit (Applied Biosystems) with the forward PCR primer as a sequencing primer. All primers are listed in Supplementary Table S1c.

We analyzed the methylation status of MGMT by bisulfite modification of the genomic DNA followed by pyrosequencing as previously described 45. We averaged the methylation values across the 16 CpG sites tested within the MGMT promoter. With an average methlyation above 10%, the samples were considered as MGMTp-meth. Primers for MGMT promoter methylation analysis are listed in Supplementary Table S1d.

### Gene set enrichment analysis

Gene Set Enrichment Analysis (GSEA) was used to identify associated signaling pathways between CTSI high-risk and CTSI low-risk groups. 6466 Annotated gene sets were downloaded from the molecular signatures database (MSigDB) C5 GO gene sets collection 46. Changes in gene expression and gene sets were evaluated using Permutation testing (1,000 permutations). The gene sets with a false discovery rate (FDR) < 0.05 were considered to be significantly enriched 46. The GSEA results were visualized using Cytoscape 3.2 and Enrichment Maps 47, 48.

### Statistical analysis

The receiver operating characteristic (ROC) curve was used to evaluate the prognostic performance of CTSI. Youden's index was calculated in the training dataset to identify the cutoff point of the CTSI score. The calculation formula of Youden's J statistic is shown as below:


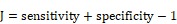


With the two right-hand quantities being sensitivity and specificity, the formula is:


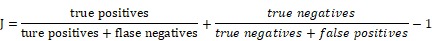


The GBM patients in the training and validation datasets were divided into the high-risk group and the low-risk group. The Kaplan-Meier method was used to estimate survival time for all the datasets and Xiangya GBM cohort. Multivariate Cox regression analysis was performed to test whether CTSI risk score was a significant predictor of survival after taking into account other important variables. The receiver operating characteristic curves was used to analyze the sensitivity and specificity of CTSI risk score in the TCGA 529 patient cohort dataset. Area under the curve (AUC) was calculated based on ROC. All analyses were performed using R/Bio-Conductor (version 3.2.2).

## Supplementary Material

Supplementary figures and tables.

## Figures and Tables

**Table 1:** Cox proportional hazard model for overall survival

		Univariate Cox regression Method: Enter				Multivariate Cox regression Method: Backward likewise			
		Co.ef	Std.Err	P value	HR (95% CI)	Co.ef	Std.Err	P value	HR (95% CI)
**TCGA 195 patients cohort**									
	**Age**	0.80	0.11	<0.001	1.33 (1.09-1.62)				
	**Gender**	0.10	0.10	0.34	2.33 (1.79-3.05)				
	**KPS**	-0.85	0.14	<0.001*	0.45 (0.36-0.56)	-0.46	0.26	0.081	0.63 (0.37-1.06)
	**Surgical Resection**	0.24	0.24	0.316	1.27 (0.78-2.02)				
	**G-CIMP**	-1.15	0.20	<0.001*	0.81 (0.70-0.95)	-1.12	0.21	<0.001*	0.80 (0.69-0.96)
	**Molecular subtype**	0.01	0.10	0.892	1.01 (0.83-1.24)				
	**CTSI risk score**	1.27	0.24	<0.001*	3.55 (2.23-5.64)	1.26	0.22	<0.001*	3.54 (2.26-5.46)
		No. of subject=161, No. of event=106, No. of censored=55, Log likelihood=835.008							
**TCGA 529 patients cohort**									
	**Age**	0.53	0.14	<0.001*	1.69 (1.29-2.21)	0.55	0.14	<0.001*	1.73 (1.33-2.26)
	**Gender**	-0.08	0.13	0.558	0.93 (0.72-1.19)				
	**KPS**	-3.34	0.15	0.027*	0.71 (0.53-0.96)	-0.34	0.15	0.021*	0.71 (0.53-0.95)
	**Surgical Resection**	0.14	0.17	0.398	1.15 (0.83-1.61)				
	**G-CIMP**	-1.15	0.26	<0.001*	0.32 (0.19-0.53)	-0.99	0.24	<0.001*	0.37 (0.23-0.59)
	**Molecular subtype**	0.07	0.06	0.024*	1.07 (0.95-1.21)				
	**Treatment modality**	-3.45	0.234	0.137	0.71 (0.45-1.12)	-0.39	0.23	0.084	0.67 (0.43-1.06)
	**CTSI risk score**	0.49	0.13	<0.001*	1.63 (1.27-2.09)	0.49	0.13	<0.001*	1.65 (1.29-2.11)
		No. of subject=369 , No. of event=279, No. of censored=90, Log likelihood=2722.843							
		
**GSE4271 dataset**									
	**Age**	1.77	1.58	0.263	5.89 (1.26-9.83)				
	**Gender**	-0.41	0.82	0.619	0.67 (0.13-3.31)				
	**Necrosis**	-0.09	1.19	0.939	0.91 (0.09-9.36)				
	**CTSI risk score**	2.28	1.22	0.048*	5.73 (1.18-9.31)	1.54	0.84	0.047*	4.65 (1.12-8.02)
		No. of subject=54, No. of event=42, No. of censored=12, Log likelihood=424.526							
**CGGA dataset**									
	**Recurrence**	0.41	0.22	0.059	1.50 (0.98-2.29)				
	**CTSI risk score**	0.99	0.24	<0.001*	2.69 (1.68-4.32)	0.94	0.34	<0.001*	2.56 (1.56-4.08)
		No. of subject=138, No. of event=92, No. of censored=46, Log likelihood=743.422							
**Xiangya GBM cohort**									
	**Age**	0.48	0.19	0.016*	1.61 (1.09-2.38)	0.48	0.19	0.016*	1.61 (1.09-2.38)
	**Gender**	-0.55	0.45	0.221	0.58 (0.24-1.39)				
	**KPS**	-0.48	0.21	0.021*	0.62 (0.41-0.93)	-0.48	0.21	0.021*	0.62 (0.41-0.93)
	**Surgical Resection**	0.01	0.32	0.970	1.01 (0.54-1.88)				
	**Treatment modality**	-0.82	0.22	<0.001*	0.44 (0.29-0.68)	-0.82	0.22	<0.001*	0.44 (0.29-0.67)
	**CTSI risk score**	0.52	0.19	0.008*	1.68 (1.14-2.46)	0.52	0.19	0.008*	1.68 (1.15-2.46)
	**TERT promoter** **mutation**	0.62	0.20	0.003*	1.85 (1.24-2.76)	0.61	0.20	0.003*	1.85 (1.24-2.75)
	**MGMT promoter** **methylation**	-0.98	0.24	<0.001*	0.38 (0.23-0.61)	-0.98	0.24	<0.001*	0.38 (0.23-0.61)
	**IDH mutation**	-1.98	0.62	0.002*	0.14 (0.04-0.47)	-1.97	0.62	0.001*	0.14 (0.04-0.47)
		No. of subject=178, No. of event=122, No. of censored=56, Log likelihood=1014.674							

Age: 0=Age<65 years, 1= Age>65 years. Gender: 0=Female, 1= Male. KPS: 0= KPS<70, 1=KPS>=70. Surgical resection: 0 = Others, 1= total resection. G-CIMP: 0=Non-C-CIMP, 1=G-CIMP Molecular subtype: 0= Non-proneural subtype. 1= Proneural subtype. Treatment modality: 0= Without chemotherapy or radiotherapy, 1= Chemotherapy with/or radiotherapy. Necrosis: 0= No necrosis, 1=Necrosis. Recurrence: 0= No recurrence, 1= Recurrence. CTSI risk score: 0= Low risk score group; 1= High risk score group. TERT promoter mutation: 0=Wildtype; 1=Mutated. MGMT promoter methylation: 0=Unmethylated; 1=Methylated. IDH mutation: 0=Wildtype; 1=Mutated. *,p<0.05 accepted as significance.

**Table 2:** ** Cox proportional hazard model for IDH wildtype, TERT promoter mutated and MGMT promoter unmethylated patients** (No. of subject=58, No. of event=44, No. of censored=14, Log likelihood=269.149).

		Univariate Cox regression Method: Enter				Multivariate Cox regression Method: Backward likewise			
		Co.ef	Std.Err	P value	HR (95% CI)	Co.ef	Std.Err	P value	HR (95% CI)
	**Age**	0.05	0.37	0.895	1.05 (0.51-2.18)				
	**Gender**	0.28	0.35	0.415	1.33 (0.67-2.66)				
	**KPS**	-0.01	0.33	0.969	0.98 (0.51-1.92)				
	**Surgical Resection**	-0.44	0.58	0.450	0.65 (0.21-1.99)				
	**Treatment modality**	-1.19	0.38	0.002*	0.30 (0.14-0.65)	-1.06	0.33	0.001*	0.35 (0.18-0.67)
	**CTSI risk score**	0.63	0.37	0.091	1.87 (0.90-3.86)	0.68	0.33	0.037*	1.98 (1.04-3.75)

Age: 0=Age<65 years, 1= Age>65 years. Gender: 0=Male; 1=Female. KPS: 0= KPS<70, 1=KPS>=70. Surgical resection: 0 = Others, 1= total resection. Treatment modality: 0= Without chemotherapy or radiotherapy, 1= Chemotherapy with radiotherapy. CTSI risk score: 0= low risk score group; 1= high risk score group. *, p<0.05 accepted as significance.

**Table 3:** ** Cox proportional hazard model for IDH wildtype, TERT promoter wildtype and MGMT promoter unmethylated patients** (No. of subject=67, No. of event=46, No. of censored=21, Log likelihood=293.099).

		Univariate Cox regression Method: Enter				Multivariate Cox regression Method: Backward likewise			
		Co.ef	Std.Err	P value	HR (95% CI)	Co.ef	Std.Err	P value	HR (95% CI)
	**Age**	0.58	0.33	0.079	0.40 (0.21-0.77)				
	**Gender**	-0.74	0.36	0.137	0.48 (0.24-1.06)				
	**KPS**	-0.89	0.34	0.008*	0.41 (0.21-0.79)	-0.91	0.33	0.006	0.40 (0.21-0.77)
	**Surgical Resection**	-0.47	0.54	0.381	0.63 (0.22-0.79)				
	**Treatment modality**	-1.34	0.41	0.001*	0.26 (0.12-0.59)	-1.25	0.40	0.002*	0.29 (0.13-0.63)
	**CTSI risk score**	0.84	0.37	0.022*	2.32 (1.13-4.76)	0.74	0.34	0.032*	2.09 (1.07-4.08)

Age: 0=Age<65 years, 1= Age>65 years. Gender: 0=Male; 1=Female. KPS: 0= KPS<70, 1=KPS>=70. Surgical resection: 0 = Others, 1= total resection. Treatment modality: 0= Without chemotherapy or radiotherapy, 1= Chemotherapy with radiotherapy. CTSI risk score: 0= low risk score group; 1= high risk score group. *, p<0.05 accepted as significance.

## Abbreviations

GBM: Glioblastoma
TCGA: The Cancer Genome Atlas
GEO: Gene Expression Omnibus
CGGA: Chinese Glioma Genome Atlas
GSEA: Gene set enrichment analysis
GO: Gene Ontology
CTSI: Combined Therapy Sensitivity Index
HR: Hazard Ratio
IDH-wt: IDH wild-type
IDH-mut: IDH-mutant
MGMTp-unmeth: MGMT promoter unmethylated
MGMTp-meth: MGMT promoter methylated
TERTp-mut: TERT promoter mutant
TERTp-wt: TERT promoter wild-type
G-CIMP: Glioma CpG island methylator phenotype
ROC: Receiver operating characteristic
AUC: Area under the curve
OS: Overall survival
RMA: Robust Multichip Average
MSigDB: Molecular signatures database
FDR: False discovery rate
