# Supplementary material for: Combined Therapy Sensitivity Index Based on a 13-Gene Signature Predicts Prognosis for IDH Wild-type and MGMT Promoter Unmethylated Glioblastoma Patients
Source: J Cancer. 2019 Aug 29;10(22):5536–48. doi: 10.7150/jca.30614 (PMC6775685; doi:10.7150/jca.30614)
Supplement: Supplementary file 1 — Supplementary figures and tables. [file jcav10p5536s1.pdf]

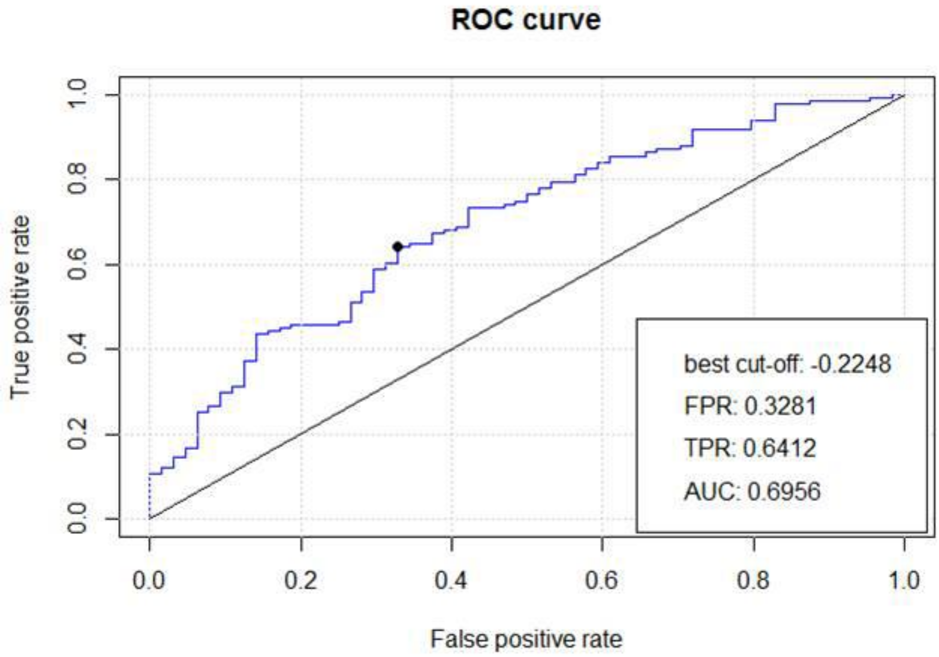

Figure S1. Receiver operating characteristic (ROC) curve for cutoff point determination. CTSI risk scores of TCGA dataset were ranked and the cutoff point was calculated by maximizing Youden index. -0.2248 was set as the cutoff point.

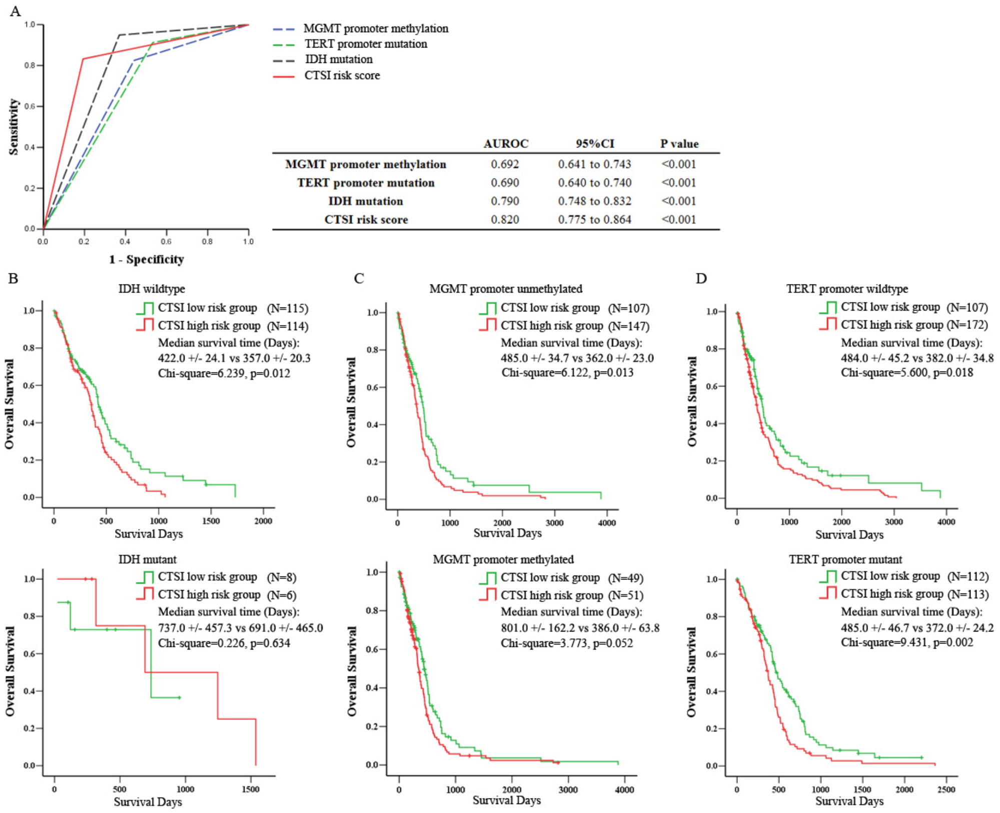

Figure S2.
A. Receiver operating characteristic (ROC) analysis of sensitivity and specificity of CTSI risk score model. MGMT promoter methylation, TERT promoter mutation and IDH mutation were assessed as references. The score performance was assessed by calculating the area under the ROC (AUROC). **B-D.** Survival of GBM patients in different CTSI risk groups stratified by IDH mutation, MGMT promoter methylation and TERT promoter mutation.

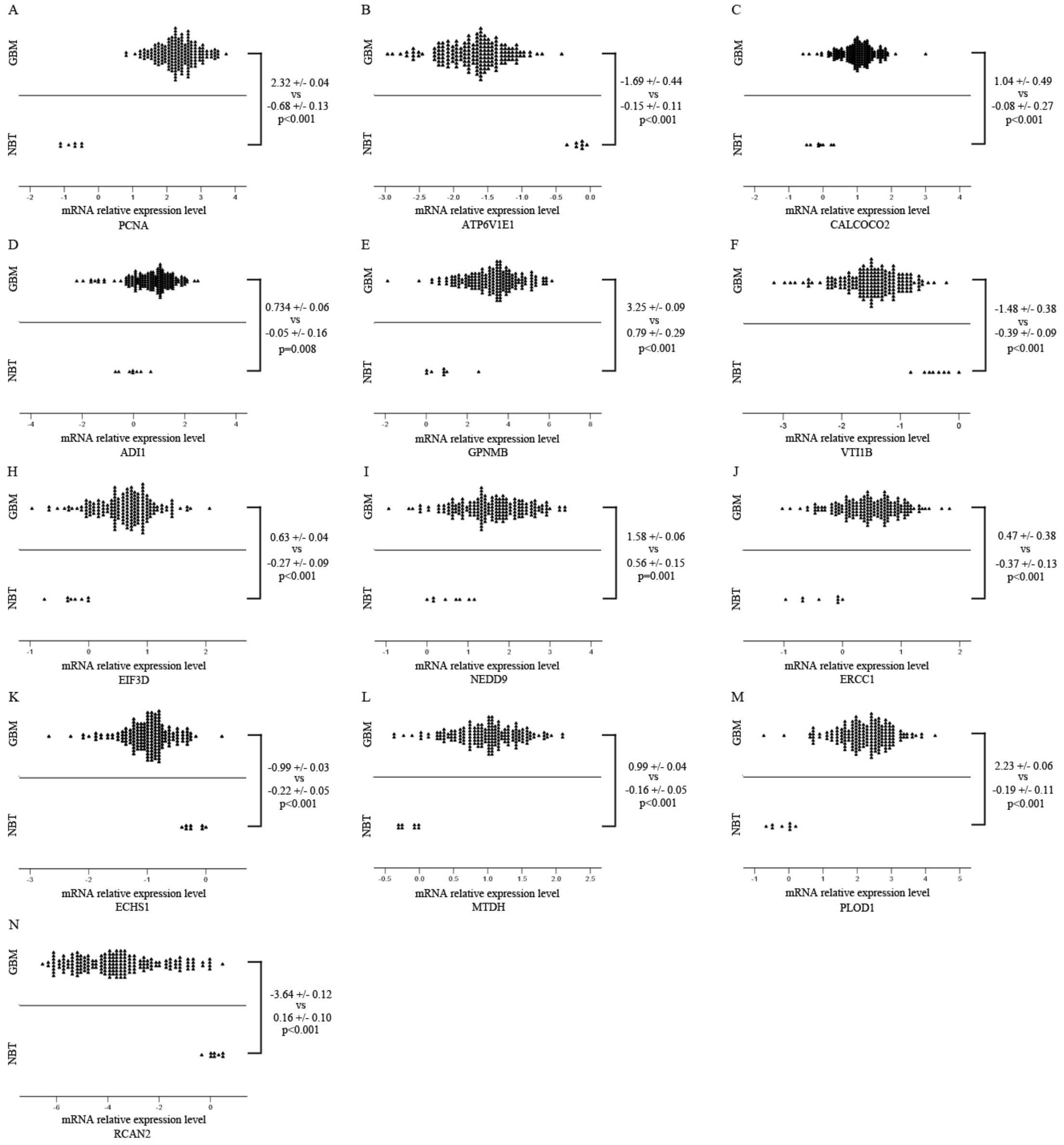

Figure S3.
Relative mRNA expression of the 13 signature genes of CTSI risk score model in Xiangya GBM cohort. Compared with normal brain tissue, PCNA, CALCOCO2, ADI1, GPNMB, MTDH, EIF3D, NEDD9, ERCC1 and PLOD1 were upregulated and ATP6V1E1, VTI1B, ECHS1 and CAN2 were down regulated in GBM patients.

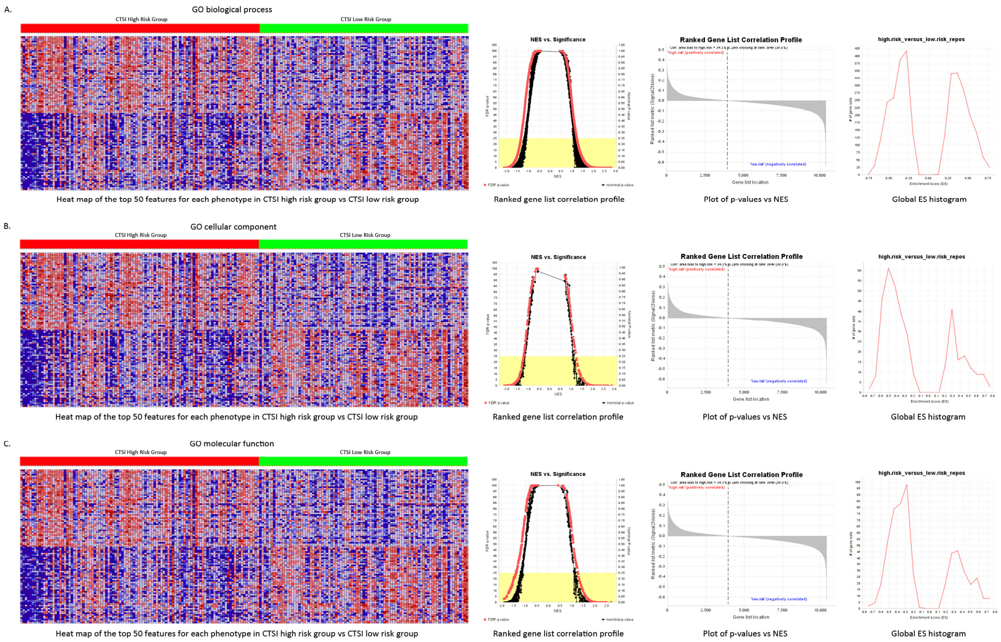

Figure S4.
The heatmap of top 50 feature for each CTSI phenotypes, the ranked gene list correlation profile, the plot of p-value vs NES and the gliobal ES histogram for GO biological process **(A)**, GO cellular component **(B)** and GO molecular function **(C)**.

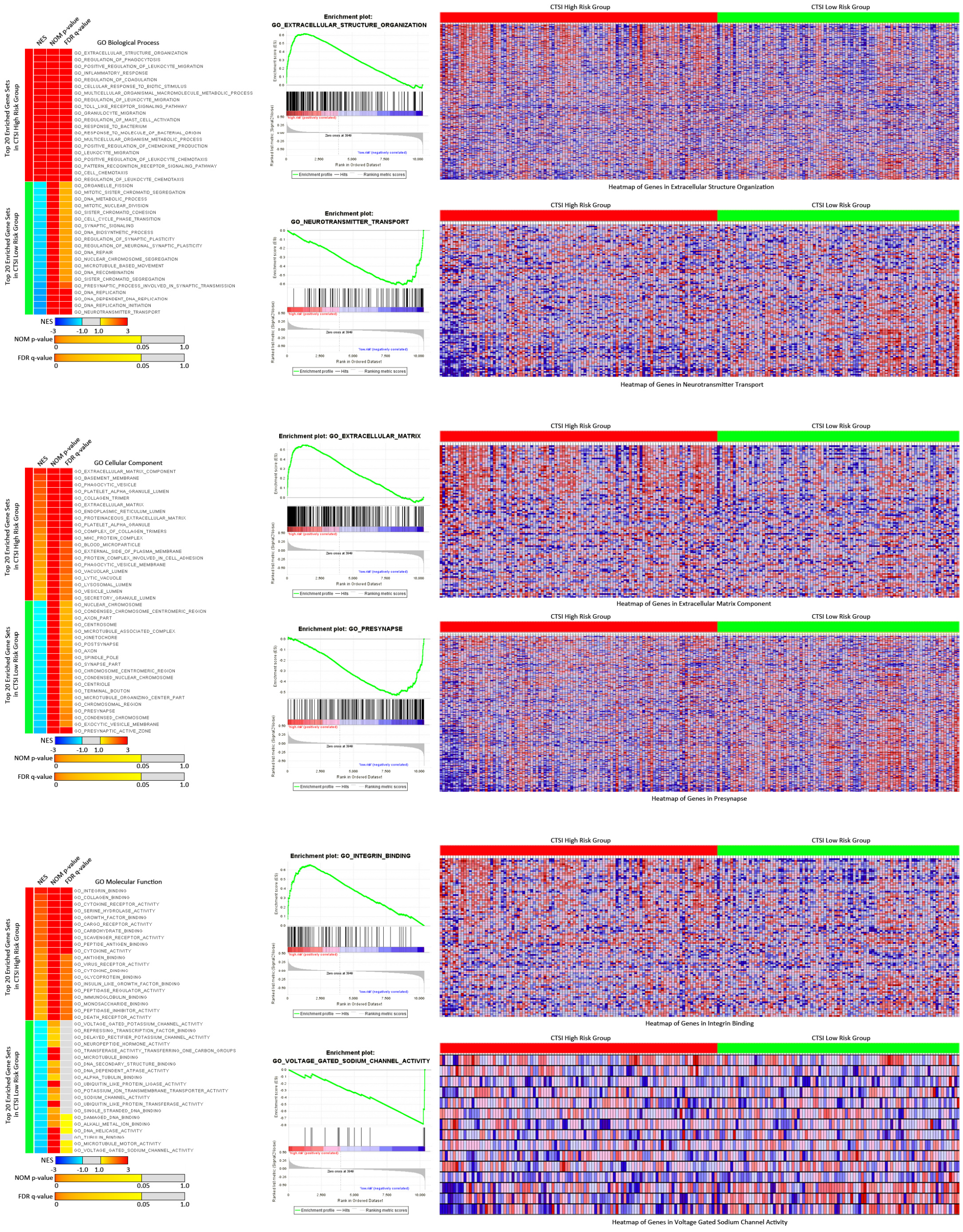

Figure S5.
Top 20 upregulated gene sets in both CTSI phenotypes, GSEA enriched profiles and heatmaps of gene set with highest NES in both CTSI high risk and low risk groups for GO biological process **(A)**, GO cellular component **(B)** and GO molecular function **(C).**

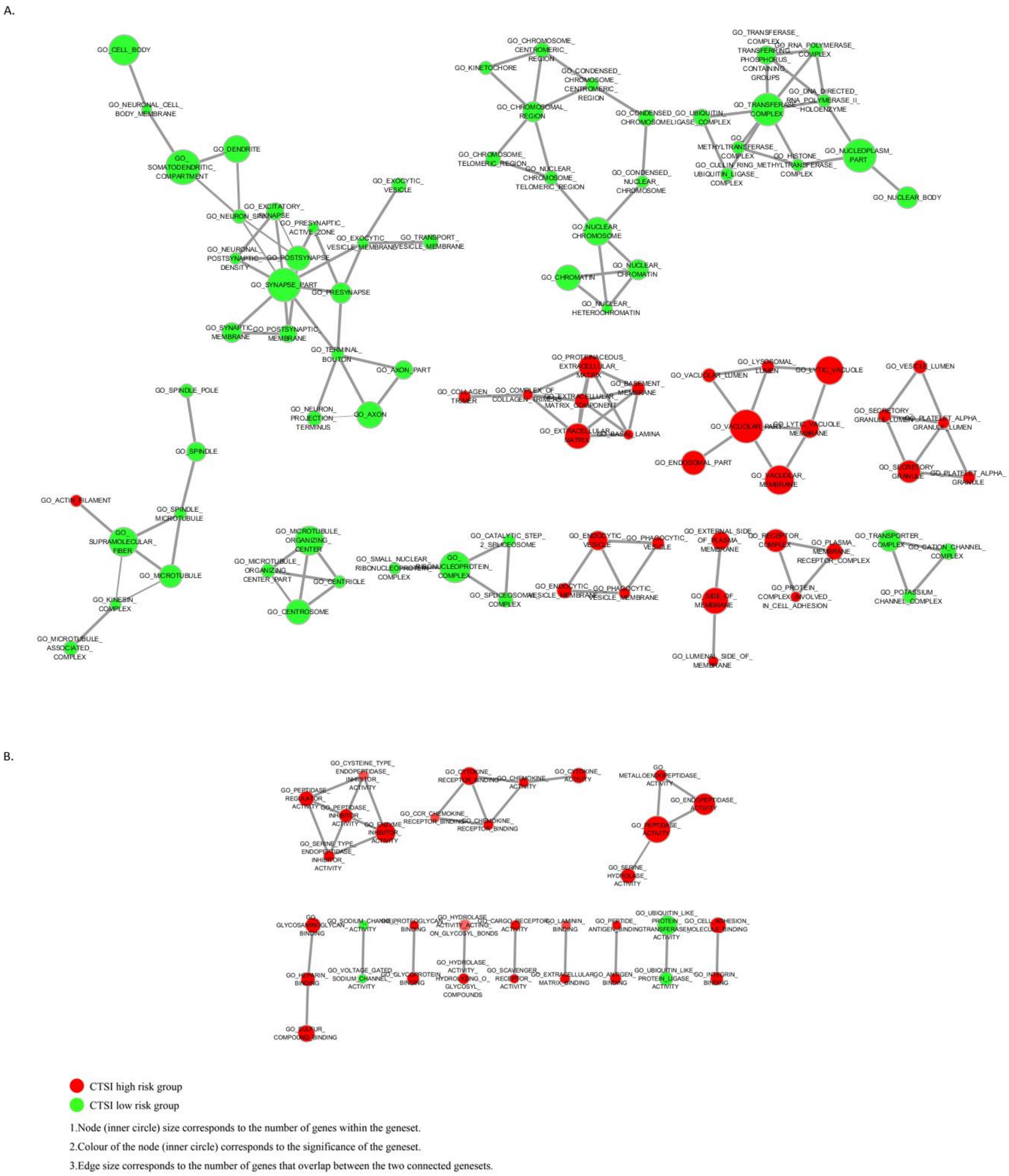

Figure S6.
Enrichment map contrasting both CTSI risk score phenotypes for GO cellular component **(A)** and GO molecular function **(B)**. Each node represents a gene set. Links between nodes represent the genes shared by both gen sets (filtered with $p<0.05$, $FDR<0.05$, Jaccard coefficient <0.95). The node color represents the strength and direction of enrichment (red gene sets are up-regulated in CTSI high risk phenotype, green ones are up-regulated in CTSI low risk phenotype). The figure was made by the Enrichment MAP from Cytoscape 3.2.

Table S1

a. Primers for quantitative real-time PCR

Gene	Forward Sequencing primer	Reverse Sequencing primer
**ADI1**	GCGGATCCATGGCGGCGACGGCACTGAGCA	ATAGGAGCTCTTAGATGAGGTCGTCCTCCTTG
**ATP6V1E1**	GATGGCTTTCATCGAACAAGAAG	GAAAGGTCGTCTTGTGCAAAC
**CALCOCO2**	TTAGCCCATTGGCCTGGGAGAGAT	TGAAGCTTGAGCTTGAGCCCATG
**ECHS1**	GCCTTTGCAGCTGGAGCCGA	GCAGGATT TCCGGCTGCCCA
**EIF3D**	CTGGAGGAGGGCAAATACCT	CTCGGTGGAAGGACAAACTC
**ERCC1**	GGGAATTTGGCGACGTAATTC	GCGGAGGCTGAGGAACAG
**GPNMB**	AGAGTCAAGCCCTGACTGGC	GAAGAGTGGGTTCCCAGTCA
**MTDH**	TGGCAAATGTGGCCAACA	TATTAGGTAACCGACCCCCTCTT
**NEDD9**	CCGCTCGAGATGTGGACAAGGAATCTTATGGC	CCGGAATTCAGAACGTTGCCATCTCCAGCAAAGA
**PCNA**	GATCGGATCCGTATGTTCGAGGCGCGCCTGGTC	GCTAGGATCCTAAGATCCTTCTTCATCCTCGATC
**PLOD1**	GAGCGGGAGTGGCACAAAT	GGAAGTGGTAGTTGTAGCGG
**RCAN2**	CCTGCAATGTTCACCAGTCTG	TCTGTCTCTGGGGTCTGGAC
**VTI1B**	CTCTTCTATGATTTCTGTACC	GAGGGATCCAATACCTTCTC

b. Primers for IDH mutation analysis

	Forward Sequencing primer	Reverse Sequencing primer
**IDH1**		
**Amplification**	ATATTCTGGGTGGCACGGTCTT	CCTTGCTTAATGGGTGTAGATACCA
**Sequencing**	CGGTCTTCAGAGAAGCCATT	
**IDH2**		
**Amplification**	TTCTGGTTGAAAGATGGCG	CAGGTCAGTGGATCCCCTC
**Sequencing**	ATGGCGGCTGCAGTGGG	

c. Primers for TERT promoter mutation analysis

	Forward Sequencing primer	Reverse Sequencing primer
**First PCR**	GCACAGACGCCCAGGACCGCGCT	TTCCCACGTGCGCAGCAGGACGCA
**Second PCR**	CAGGAAACAGCTATGACCATGATTACGGCACAGACGC CCAGGACCGCGCT	CGTTGTAAAACGACGGCCAGTGAATTGTTCCCACGTGCGCAG CAGGACGCA
**Sequencing**	CAGGAAACAGCTATGACCATGATTACG	

d. Primers for MGMT promoter methylation analysis

	Forward Sequencing primer	Reverse Sequencing primer
**Amplification**	GTTTYGGATATGTTGGGATAG	AAAACCACTCRAAACTACCAC
**Sequencing**	GATAGTTYGYGTTTTTAGAA	

Table S2. Detailed information for the 13 genes in CTSI model.

Coefficients of the 13 genes significantly associated with overall survival in the training set patients (n=195).

Gene symbol	Ensembl id	Cytoband	Co.ef	Z	P value
**ADI1**	ENSG00000182551	2p25.3	0.717	3.633	0.000
**ATP6V1E1**	ENSG00000131100	22pter-q11.2|22q11.1	-1.931	-4.649	3.34E-06
**CALCOCO2**	ENSG00000136436	17q21.32	-0.971	-3.586	0.000
**ECHS1**	ENSG00000127884	10q26.2-q26.3	1.517	4.121	3.78E-05
**EIF3D**	ENSG00000100353	22q13.1	1.839	5.021	5.13E-07
**ERCC1**	ENSG00000012061	19q13.2-q13.3	-0.950	-3.937	8.25E-05
**GPNMB**	ENSG00000136235	7p15	0.343	2.739	0.006
**MTDH**	ENSG00000147649	8q22.1	-1.085	-3.311	0.000
**NEDD9**	ENSG00000111859	6p25-p24	0.699	3.580	0.000
**PCNA**	ENSG00000132646	20pter-p12	-1.178	-5.234	1.66E-07
**PLOD1**	ENSG00000083444	1p36.22	0.933	4.0516	5.09E-05
**RCAN2**	ENSG00000172348	6p12.3	-0.258	-2.977	0.003
**VTI1B**	ENSG00000100568	14q24.1	0.874	2.670	0.007

Table S3.

Clinical and pathological characteristics of patients from Xiangya GBM cohort

**No. of patients**	178
**Gender**	
Male	102 (57.3%)
Female	76 (42.7%)
**Age**	
>65 years	71 (39.9%)
<65 years	107 (60.1%)
**KPS**	
<70	50 (32.0%)
>70	121 (68.0%)
**Resection**	
Total resection	161 (90.4%)
Others (Subtotal, Partial, Biopsy)	17 (9.6%)
**Treatment modality**	
Combined radio-chemotherapy	138 (78.0%)
Others (Radiotherapy alone, Chemotherapy alone, or Without chemotherapy or radiotherapy)	39 (22.0%)
**TERT promoter mutation**	
Wild-type	95 (53.4%)
Mutated	83 (46.6%)
**MGMT promoter methylation**	
Unmethylated	132 (74.2%)
Methylated	46 (25.8%)
**IDH mutation**	
Wildtype	167 (93.8%)
Mutated	11 (6.2%)
**For IDH wildtype**	
TERT promoter mutated-MGMT promoter unmethylated	58 (34.7%)
TERT promoter wildtype-MGMT promoter unmethylated	69 (41.3%)
TERT promoter mutated-MGMT promoter methylated	25 (15.0%)
TERT promoter wildtype-MGMT promoter methylated	15 (9.0%)
**CTSI risk score**	
Low risk score	87 (48.9%)
High risk score	91 (51.1%)

Table S4. Enriched pathways in GO biological process, celluar component, molecular function for CTSI high risk and low risk group

Enriched pathways in GO biological process for CTSI high risk group

NAME	GS<br>follow link to MSigDB	SIZE	ES	NES	NOM p-val	FDR q-val	FWER p-val	RANK AT MAX	LEADING EDGE
GO_EXTRACELLULAR_STRUCTURE_ORGANIZATION	GO_EXTRACELLULAR_STRUCTURE_ORGANIZATION	242	0.6197594	2.7972374	0	0	0	1348	tags=43%, list=13%, signal=49%
GO_REGULATION_OF_PHAGOCYTOSIS	GO_REGULATION_OF_PHAGOCYTOSIS	59	0.72397065	2.718413	0	0	0	772	tags=37%, list=7%, signal=40%
GO_POSITIVE_REGULATION_OF_LEUKOCYTE_MIGRATION	GO_POSITIVE_REGULATION_OF_LEUKOCYTE_MIGRATION	95	0.6591436	2.7076788	0	0	0	1053	tags=36%, list=10%, signal=39%
GO_INFLAMMATORY_RESPONSE	GO_INFLAMMATORY_RESPONSE	337	0.56646526	2.6900823	0	0	0	1602	tags=42%, list=15%, signal=48%
GO_REGULATION_OF_COAGULATION	GO_REGULATION_OF_COAGULATION	76	0.67656267	2.6786442	0	0	0	499	tags=25%, list=5%, signal=26%
GO_CELLULAR_RESPONSE_TO_BIOTIC_STIMULUS	GO_CELLULAR_RESPONSE_TO_BIOTIC_STIMULUS	132	0.62643594	2.6327913	0	0	0	1342	tags=42%, list=13%, signal=48%
GO_MULTICELLULAR_ORGANISMAL_MACROMOLECULE_METABOLIC_PROCESS	GO_MULTICELLULAR_ORGANISMAL_MACROMOLECULE_METABOLIC_PROCESS	67	0.68751717	2.6227794	0	0	0	826	tags=39%, list=8%, signal=42%
GO_REGULATION_OF_LEUKOCYTE_MIGRATION	GO_REGULATION_OF_LEUKOCYTE_MIGRATION	123	0.6050388	2.588253	0	0	0	1549	tags=43%, list=15%, signal=50%
GO_TOLL_LIKE_RECEPTOR_SIGNALING_PATHWAY	GO_TOLL_LIKE_RECEPTOR_SIGNALING_PATHWAY	70	0.67479634	2.586494	0	0	0	1538	tags=51%, list=15%, signal=60%
GO_GRANULOCYTE_MIGRATION	GO_GRANULOCYTE_MIGRATION	54	0.698096	2.5842927	0	0	0	1485	tags=48%, list=14%, signal=56%

GO_REGULATION_OF_MAST_CELL_ACTIVATION	GO_REGULATION_OF_MAST_CELL_ACTIVATION	34	0.7710577	2.568773	0	0	0	1344	tags=62%, list=13%, signal=71%
GO_RESPONSE_TO_BACTERIUM	GO_RESPONSE_TO_BACTERIUM	362	0.5301892	2.5420144	0	0	0	1502	tags=36%, list=14%, signal=41%
GO_RESPONSE_TO_MOLECULE_OF_BACTERIAL_ORIGIN	GO_RESPONSE_TO_MOLECULE_OF_BACTERIAL_ORIGIN	265	0.5504713	2.5363593	0	0	0	1538	tags=40%, list=15%, signal=46%
GO_MULTICELLULAR_ORGANISM_METABOLIC_PROCESS	GO_MULTICELLULAR_ORGANISM_METABOLIC_PROCESS	79	0.64036447	2.5290353	0	0	0	997	tags=35%, list=10%, signal=39%
GO_POSITIVE_REGULATION_OF_CHEMOKINE_PRODUCTION	GO_POSITIVE_REGULATION_OF_CHEMOKINE_PRODUCTION	41	0.72708976	2.5247896	0	0	0	1055	tags=37%, list=10%, signal=41%
GO_LEUKOCYTE_MIGRATION	GO_LEUKOCYTE_MIGRATION	216	0.55612653	2.5079126	0	0	0	1502	tags=36%, list=14%, signal=41%
GO_POSITIVE_REGULATION_OF_LEUKOCYTE_CHEMOTAXIS	GO_POSITIVE_REGULATION_OF_LEUKOCYTE_CHEMOTAXIS	72	0.63731736	2.495578	0	0	0	1053	tags=35%, list=10%, signal=38%
GO_PATTERN_RECOGNITION_RECEPTOR_SIGNALING_PATHWAY	GO_PATTERN_RECOGNITION_RECEPTOR_SIGNALING_PATHWAY	86	0.6207458	2.4952047	0	0	0	1538	tags=47%, list=15%, signal=54%
GO_CELL_CHEMOTAXIS	GO_CELL_CHEMOTAXIS	131	0.57508934	2.4944785	0	0	0	1502	tags=44%, list=14%, signal=50%
GO_REGULATION_OF_LEUKOCYTE_CHEMOTAXIS	GO_REGULATION_OF_LEUKOCYTE_CHEMOTAXIS	83	0.6386699	2.4942706	0	0	0	1549	tags=45%, list=15%, signal=52%
GO_REGULATION_OF_CELL_ADHESION_MEDIATED_BY_INTEGRIN	GO_REGULATION_OF_CELL_ADHESION_MEDIATED_BY_INTEGRIN	34	0.7695297	2.4906976	0	0	0	1048	tags=44%, list=10%, signal=49%
GO_LEUKOCYTE_CHEMOTAXIS	GO_LEUKOCYTE_CHEMOTAXIS	93	0.61215186	2.4743009	0	0	0	1502	tags=45%, list=14%, signal=52%
GO_ADAPTIVE_IMMUNE_RESPONSE	GO_ADAPTIVE_IMMUNE_RESPONSE	186	0.56937015	2.4638777	0	0	0	1471	tags=36%, list=14%, signal=41%

GO_REGULATION_OF_TUMOR_NECROSIS_FACTOR_SUPERFAMILY_CYTOKINE_PRODUCTION	GO_REGULATION_OF_TUMOR_NECROSIS_FACTOR_SUPERFAMILY_CYTOKINE_PRODUCTION	73	0.62358725	2.4627237	0	0	0	1244	tags=42%, list=12%, signal=48%
GO_LEUKOCYTE_CELL_CELL_ADHESION	GO_LEUKOCYTE_CELL_CELL_ADHESION	208	0.54136777	2.442229	0	0	0	1487	tags=35%, list=14%, signal=40%
GO_REGULATION_OF_MAST_CELL_DEGRANULATION	GO_REGULATION_OF_MAST_CELL_DEGRANULATION	27	0.7774333	2.4325702	0	0	0	1225	tags=59%, list=12%, signal=67%
GO_PHAGOCYTOSIS	GO_PHAGOCYTOSIS	132	0.5674785	2.4264112	0	0	0	1469	tags=39%, list=14%, signal=45%
GO_INTEGRIN_MEDIATED_SIGNALING_PATHWAY	GO_INTEGRIN_MEDIATED_SIGNALING_PATHWAY	69	0.6582893	2.4205039	0	0	0	1099	tags=41%, list=11%, signal=45%
GO_POSITIVE_REGULATION_OF_CHEMOTAXIS	GO_POSITIVE_REGULATION_OF_CHEMOTAXIS	105	0.57835275	2.4201713	0	0	0	1485	tags=38%, list=14%, signal=44%
GO_NEGATIVE_REGULATION_OF_TUMOR_NECROSIS_FACTOR_SUPERFAMILY_CYTOKINE_PRODUCTION	GO_NEGATIVE_REGULATION_OF_TUMOR_NECROSIS_FACTOR_SUPERFAMILY_CYTOKINE_PRODUCTION	29	0.7520658	2.420029	0	0	0	1244	tags=52%, list=12%, signal=59%
GO_PLATELET_DEGRANULATION	GO_PLATELET_DEGRANULATION	89	0.60376984	2.4164376	0	0	0	1176	tags=36%, list=11%, signal=40%
GO_REGULATION_OF_MAST_CELL_ACTIVATION_INVOLVED_IN_IMMUNE_RESPONSE	GO_REGULATION_OF_MAST_CELL_ACTIVATION_INVOLVED_IN_IMMUNE_RESPONSE	27	0.7774332	2.4108524	0	0	0	1225	tags=59%, list=12%, signal=67%
GO_POSITIVE_REGULATION_OF_PHAGOCYTOSIS	GO_POSITIVE_REGULATION_OF_PHAGOCYTOSIS	39	0.7152593	2.406105	0	0	0	772	tags=41%, list=7%, signal=44%
GO_REGULATION_OF_INTERLEUKIN_6_PRODUCTION	GO_REGULATION_OF_INTERLEUKIN_6_PRODUCTION	72	0.6123379	2.3987062	0	0	0	1297	tags=40%, list=12%, signal=46%
GO_REGULATION_OF_CHEMOTAXIS	GO_REGULATION_OF_CHEMOTAXIS	152	0.55861056	2.3949757	0	0	0	1741	tags=41%, list=17%, signal=49%

GO_DEFENSE_RESPONSE_TO_BACTERIUM	GO_DEFENSE_RESPONSE_TO_BACTERIUM	125	0.5671345	2.391989	0	0	0	1216	tags=31%, list=12%, signal=35%
GO_POSITIVE_REGULATION_OF_COAGULATION	GO_POSITIVE_REGULATION_OF_COAGULATION	22	0.80215716	2.3906176	0	0	0	706	tags=36%, list=7%, signal=39%
GO_LIPOPOLYSACCHARIDE_MEDIATED_SIGNALING_PATHWAY	GO_LIPOPOLYSACCHARIDE_MEDIATED_SIGNALING_PATHWAY	29	0.7362728	2.3855233	0	0	0	1766	tags=62%, list=17%, signal=75%
GO_COLLAGEN_FIBRIL_ORGANIZATION	GO_COLLAGEN_FIBRIL_ORGANIZATION	31	0.7458733	2.3824	0	0	0	604	tags=48%, list=6%, signal=51%
GO_REGULATION_OF_INFLAMMATORY_RESPONSE	GO_REGULATION_OF_INFLAMMATORY_RESPONSE	223	0.52706194	2.3812006	0	0	0	1341	tags=35%, list=13%, signal=40%
GO_REGULATION_OF_LEUKOCYTE_DEGRANULATION	GO_REGULATION_OF_LEUKOCYTE_DEGRANULATION	36	0.7053962	2.3790536	0	0	0	1053	tags=50%, list=10%, signal=55%
GO_POSITIVE_REGULATION_OF_LOCOMOTION	GO_POSITIVE_REGULATION_OF_LOCOMOTION	341	0.49634296	2.3774683	0	0	0	1196	tags=29%, list=12%, signal=32%
GO_POSITIVE_REGULATION_OF_RESPONSE_TO_WOUNDING	GO_POSITIVE_REGULATION_OF_RESPONSE_TO_WOUNDING	127	0.5544876	2.3769767	0	0	0	1281	tags=37%, list=12%, signal=42%
GO_ADAPTIVE_IMMUNE_RESPONSE_BASED_ON_SOMATIC_RECOMBINATION_OF_IMMUNE_RECEPTORS_BUILT_FROM_IMMUNOGLOBULIN_SUPERFAMILY_DOMAINS	GO_ADAPTIVE_IMMUNE_RESPONSE_BASED_ON_SOMATIC_RECOMBINATION_OF_IMMUNE_RECEPTORS_BUILT_FROM_IMMUNOGLOBULIN_SUPERFAMILY_DOMAINS	97	0.5848604	2.3685708	0	0	0	1309	tags=39%, list=13%, signal=44%
GO_T_CELL_ACTIVATION_INVOLVED_IN_IMMUNE_RESPONSE	GO_T_CELL_ACTIVATION_INVOLVED_IN_IMMUNE_RESPONSE	41	0.6924995	2.364977	0	0	0	1200	tags=39%, list=12%, signal=44%
GO_ACUTE_INFLAMMATORY_RESPONSE	GO_ACUTE_INFLAMMATORY_RESPONSE	59	0.6350087	2.3647265	0	0	0	983	tags=31%, list=9%, signal=34%
GO_FIBRINOLYSIS	GO_FIBRINOLYSIS	21	0.8084645	2.3639586	0	0	0	306	tags=24%, list=3%, signal=24%

GO_POSITIVE_REGULATION_OF_ENDOCYTOSIS	GO_POSITIVE_REGULATION_OF_ENDOCYTOSIS	91	0.5837353	2.3586059	0	4.1E-05	0.001	1003	tags=30%, list=10%, signal=33%
GO_POSITIVE_REGULATION_OF_RESPONSE_TO_EXTERNAL_STIMULUS	GO_POSITIVE_REGULATION_OF_RESPONSE_TO_EXTERNAL_STIMULUS	236	0.5154959	2.3580823	0	4E-05	0.001	1434	tags=33%, list=14%, signal=37%
GO_POSITIVE_REGULATION_OF_INTERLEUKIN_6_PRODUCTION	GO_POSITIVE_REGULATION_OF_INTERLEUKIN_6_PRODUCTION	51	0.64666945	2.3456554	0	3.9E-05	0.001	1297	tags=41%, list=12%, signal=47%
GO_POSITIVE_REGULATION_OF_CYTOKINE_PRODUCTION	GO_POSITIVE_REGULATION_OF_CYTOKINE_PRODUCTION	285	0.50872856	2.3414803	0	3.9E-05	0.001	1808	tags=41%, list=17%, signal=48%
GO_POSITIVE_REGULATION_OF_INFLAMMATORY_RESPONSE	GO_POSITIVE_REGULATION_OF_INFLAMMATORY_RESPONSE	88	0.58955026	2.3412275	0	3.8E-05	0.001	1281	tags=41%, list=12%, signal=46%
GO_REGULATION_OF_ALPHA_BETA_T_CELL_ACTIVATION	GO_REGULATION_OF_ALPHA_BETA_T_CELL_ACTIVATION	55	0.62508166	2.3397155	0	3.7E-05	0.001	1801	tags=51%, list=17%, signal=61%
GO_WOUND_HEALING	GO_WOUND_HEALING	380	0.48971093	2.3382955	0	3.6E-05	0.001	1259	tags=28%, list=12%, signal=31%
GO_MYELOID_LEUKOCYTE_ACTIVATION	GO_MYELOID_LEUKOCYTE_ACTIVATION	78	0.59405345	2.3317032	0	3.6E-05	0.001	1200	tags=46%, list=12%, signal=52%
GO_NEGATIVE_REGULATION_OF_COAGULATION	GO_NEGATIVE_REGULATION_OF_COAGULATION	44	0.65310526	2.3307161	0	3.5E-05	0.001	494	tags=20%, list=5%, signal=21%
GO_POSITIVE_REGULATION_OF_CYTOKINE_BIOSYNTHETIC_PROCESS	GO_POSITIVE_REGULATION_OF_CYTOKINE_BIOSYNTHETIC_PROCESS	49	0.63744265	2.329695	0	3.5E-05	0.001	1580	tags=47%, list=15%, signal=55%
GO_SINGLE_ORGANISM_CELL_ADHESION	GO_SINGLE_ORGANISM_CELL_ADHESION	361	0.48048782	2.3224294	0	3.4E-05	0.001	1277	tags=30%, list=12%, signal=34%
GO_CELLULAR_DEFENSE_RESPONSE	GO_CELLULAR_DEFENSE_RESPONSE	47	0.6421891	2.3210382	0	3.3E-05	0.001	1053	tags=32%, list=10%, signal=35%
GO_POSITIVE_REGULATION_OF_T_CELL_PROLIFERATION	GO_POSITIVE_REGULATION_OF_T_CELL_PROLIFERATION	75	0.6094748	2.3198955	0	3.3E-05	0.001	1806	tags=49%, list=17%, signal=59%

GO_ENTRY_INTO_HOST_CELL	GO_ENTRY_INTO_HOST_CELL	72	0.59163356	2.3183198	0	3.2E-05	0.001	1042	tags=39%, list=10%, signal=43%
GO_REGULATION_OF_RESPONSE_TO_WOUNDING	GO_REGULATION_OF_RESPONSE_TO_WOUNDING	321	0.49395907	2.3158817	0	3.2E-05	0.001	1313	tags=31%, list=13%, signal=34%
GO_PROTEIN_ACTIVATION_CASCADE	GO_PROTEIN_ACTIVATION_CASCADE	63	0.6150966	2.3116972	0	3.1E-05	0.001	1302	tags=30%, list=13%, signal=34%
GO_MOVEMENT_IN_ENVIRONMENT_OF_OTHER_ORGANISM_INVOLVED_IN_SYMBIOTIC_INTERACTION	GO_MOVEMENT_IN_ENVIRONMENT_OF_OTHER_ORGANISM_INVOLVED_IN_SYMBIOTIC_INTERACTION	72	0.59163356	2.3074582	0	3.1E-05	0.001	1042	tags=39%, list=10%, signal=43%
GO_HETEROTYPIC_CELL_CELL_ADHESION	GO_HETEROTYPIC_CELL_CELL_ADHESION	22	0.7602233	2.29784	0	3E-05	0.001	986	tags=45%, list=9%, signal=50%
GO_POSITIVE_REGULATION_OF_CELL_ACTIVATION	GO_POSITIVE_REGULATION_OF_CELL_ACTIVATION	229	0.5068906	2.296989	0	3E-05	0.001	1470	tags=39%, list=14%, signal=44%
GO_POSITIVE_REGULATION_OF_VASCULAR_ENDOTHELIAL_GROWTH_FACTOR_PRODUCTION	GO_POSITIVE_REGULATION_OF_VASCULAR_ENDOTHELIAL_GROWTH_FACTOR_PRODUCTION	22	0.76487714	2.2964408	0	2.9E-05	0.001	1186	tags=50%, list=11%, signal=56%
GO_MYELOID_LEUKOCYTE_MIGRATION	GO_MYELOID_LEUKOCYTE_MIGRATION	78	0.5907295	2.2952237	0	2.9E-05	0.001	1281	tags=37%, list=12%, signal=42%
GO_REGULATION_OF_ANTIGEN_RECEPTOR_MEDIATED_SIGNALING_PATHWAY	GO_REGULATION_OF_ANTIGEN_RECEPTOR_MEDIATED_SIGNALING_PATHWAY	28	0.7271891	2.2806652	0	2.9E-05	0.001	1932	tags=57%, list=19%, signal=70%
GO_ENTRY_INTO_HOST	GO_ENTRY_INTO_HOST	72	0.5916336	2.2794685	0	2.8E-05	0.001	1042	tags=39%, list=10%, signal=43%
GO_ACTIVATION_OF_IMMUNE_RESPONSE	GO_ACTIVATION_OF_IMMUNE_RESPONSE	318	0.48382598	2.273525	0	8.4E-05	0.003	1313	tags=29%, list=13%, signal=32%
GO_MOVEMENT_IN_HOST_ENVIRONMENT	GO_MOVEMENT_IN_HOST_ENVIRONMENT	72	0.59163356	2.2730513	0	8.3E-05	0.003	1042	tags=39%, list=10%, signal=43%

GO_B_CELL_RECEPTOR_SIGNALING_PATHWAY	GO_B_CELL_RECEPTOR_SIGNALING_PATHWAY	32	0.6872468	2.2710915	0	8.2E-05	0.003	1615	tags=47%, list=16%, signal=55%
GO_POSITIVE_REGULATION_OF_IMMUNE_RESPONSE	GO_POSITIVE_REGULATION_OF_IMMUNE_RESPONSE	416	0.46871942	2.2692204	0	8.1E-05	0.003	1479	tags=31%, list=14%, signal=35%
GO_POSITIVE_REGULATION_OF_TUMOR_NECROSIS_FACTOR_SUPERFAMILY_CYTOKINE_PRODUCTION	GO_POSITIVE_REGULATION_OF_TUMOR_NECROSIS_FACTOR_SUPERFAMILY_CYTOKINE_PRODUCTION	45	0.625249	2.2678277	0	0.00011	0.004	1055	tags=40%, list=10%, signal=44%
GO_RESPONSE_TO_LIPOPROTEIN_PARTICLE	GO_RESPONSE_TO_LIPOPROTEIN_PARTICLE	17	0.8047726	2.2661269	0	0.00011	0.004	986	tags=65%, list=9%, signal=71%
GO_POSITIVE_REGULATION_OF_WOUND_HEALING	GO_POSITIVE_REGULATION_OF_WOUND_HEALING	40	0.6544528	2.2656484	0	0.0001	0.004	1238	tags=40%, list=12%, signal=45%
GO_POSITIVE_REGULATION_OF_IMMUNE_EFFECTOR_PROCESS	GO_POSITIVE_REGULATION_OF_IMMUNE_EFFECTOR_PROCESS	122	0.5476035	2.264911	0	0.0001	0.004	1446	tags=39%, list=14%, signal=44%
GO_EXTRACELLULAR_MATRIX_DISASSEMBLY	GO_EXTRACELLULAR_MATRIX_DISASSEMBLY	57	0.61575377	2.2622137	0	0.0001	0.004	1643	tags=56%, list=16%, signal=66%
GO_REGULATION_OF_PROTEIN_MATURATION	GO_REGULATION_OF_PROTEIN_MATURATION	60	0.58975464	2.2598095	0	0.0001	0.004	1272	tags=37%, list=12%, signal=42%
GO_ENTRY_INTO_OTHER_ORGANISM_INVOLVED_IN_SYMBIOTIC_INTERACTION	GO_ENTRY_INTO_OTHER_ORGANISM_INVOLVED_IN_SYMBIOTIC_INTERACTION	72	0.5916338	2.258367	0	9.9E-05	0.004	1042	tags=39%, list=10%, signal=43%
GO_ENTRY_INTO_CELL_OF_OTHER_ORGANISM_INVOLVED_IN_SYMBIOTIC_INTERACTION	GO_ENTRY_INTO_CELL_OF_OTHER_ORGANISM_INVOLVED_IN_SYMBIOTIC_INTERACTION	72	0.59163374	2.2561758	0	9.8E-05	0.004	1042	tags=39%, list=10%, signal=43%
GO_NEGATIVE_REGULATION_OF_IMMUNE_RESPONSE	GO_NEGATIVE_REGULATION_OF_IMMUNE_RESPONSE	93	0.55551296	2.2501426	0	9.6E-05	0.004	1451	tags=45%, list=14%, signal=52%

GO_POSITIVE_REGULATION_OF_LYMPHOCYTE_MIGRATION	GO_POSITIVE_REGULATION_OF_LYMPHOCYTE_MIGRATION	22	0.72875684	2.2495718	0	9.5E-05	0.004	1281	tags=41%, list=12%, signal=47%
GO_REGULATION_OF_T_CELL_PROLIFERATION	GO_REGULATION_OF_T_CELL_PROLIFERATION	113	0.5355838	2.2473593	0	9.4E-05	0.004	1806	tags=43%, list=17%, signal=52%
GO_EPIBOLY	GO_EPIBOLY	17	0.7928673	2.2437677	0	9.3E-05	0.004	792	tags=35%, list=8%, signal=38%
GO_POSITIVE_REGULATION_OF_LEUKOCYTE_PROLIFERATION	GO_POSITIVE_REGULATION_OF_LEUKOCYTE_PROLIFERATION	108	0.53418237	2.241646	0	9.2E-05	0.004	1469	tags=39%, list=14%, signal=45%
GO_REGULATION_OF_CHEMOKINE_PRODUCTION	GO_REGULATION_OF_CHEMOKINE_PRODUCTION	53	0.61802256	2.2412488	0	9.1E-05	0.004	560	tags=25%, list=5%, signal=26%
GO_REGULATION_OF_LEUKOCYTE_MEDIATED_IMMUNITY	GO_REGULATION_OF_LEUKOCYTE_MEDIATED_IMMUNITY	123	0.5346018	2.2406852	0	9E-05	0.004	1446	tags=40%, list=14%, signal=46%
GO_MYD88_DEPENDENT_TOLL_LIKE_RECEPTOR_SIGNALING_PATHWAY	GO_MYD88_DEPENDENT_TOLL_LIKE_RECEPTOR_SIGNALING_PATHWAY	23	0.73424464	2.2399337	0	8.9E-05	0.004	1453	tags=61%, list=14%, signal=71%
GO_POSITIVE_REGULATION_OF_ALPHA_BETA_T_CELL_ACTIVATION	GO_POSITIVE_REGULATION_OF_ALPHA_BETA_T_CELL_ACTIVATION	41	0.6363784	2.233881	0	0.00011	0.005	1470	tags=49%, list=14%, signal=57%
GO_PEPTIDE_CROSS_LINKING	GO_PEPTIDE_CROSS_LINKING	25	0.7206709	2.2334385	0	0.00011	0.005	1093	tags=36%, list=11%, signal=40%
GO_VIRAL_ENTRY_INTO_HOST_CELL	GO_VIRAL_ENTRY_INTO_HOST_CELL	72	0.5916336	2.2325437	0	0.00011	0.005	1042	tags=39%, list=10%, signal=43%
GO_REGULATION_OF_CELL_ACTIVATION	GO_REGULATION_OF_CELL_ACTIVATION	352	0.46831527	2.2292671	0	0.00011	0.005	1470	tags=35%, list=14%, signal=39%
GO_MACROPHAGE_ACTIVATION	GO_MACROPHAGE_ACTIVATION	28	0.7171836	2.2238636	0	0.00013	0.006	1326	tags=46%, list=13%, signal=53%
GO_REGULATION_OF_WOUND_HEALING	GO_REGULATION_OF_WOUND_HEALING	107	0.5429789	2.2207716	0	0.00013	0.006	499	tags=21%, list=5%, signal=22%
GO_CELLULAR_RESPONSE_TO_VITAMIN	GO_CELLULAR_RESPONSE_TO_VITAMIN	25	0.7147926	2.2182794	0	0.00012	0.006	698	tags=44%, list=7%, signal=47%

GO_ANGIOGENESIS	GO_ANGIOGENESIS	237	0.48000613	2.2142618	0	0.00012	0.006	1048	tags=28%, list=10%, signal=30%
GO_INNATE_IMMUNE_RESPONSE	GO_INNATE_IMMUNE_RESPONSE	397	0.4672536	2.2119393	0	0.00014	0.007	1539	tags=33%, list=15%, signal=37%
GO_REGULATION_OF_CYTOKINE_SECRETION	GO_REGULATION_OF_CYTOKINE_SECRETION	108	0.5271384	2.211258	0	0.00014	0.007	1244	tags=34%, list=12%, signal=39%
GO_COMPLEMENT_ACTIVATION	GO_COMPLEMENT_ACTIVATION	42	0.6369704	2.2093482	0	0.00016	0.008	667	tags=26%, list=6%, signal=28%
GO_REGULATION_OF_ACUTE_INFLAMMATORY_RESPONSE	GO_REGULATION_OF_ACUTE_INFLAMMATORY_RESPONSE	62	0.5910844	2.2018023	0	0.00018	0.009	1239	tags=40%, list=12%, signal=46%
GO_POSITIVE_REGULATION_OF_DEFENSE_RESPONSE	GO_POSITIVE_REGULATION_OF_DEFENSE_RESPONSE	285	0.47315252	2.2008598	0	0.00018	0.009	1313	tags=32%, list=13%, signal=35%
GO_CELL_MATRIX_ADHESION	GO_CELL_MATRIX_ADHESION	92	0.5491795	2.1997728	0	0.00017	0.009	1099	tags=29%, list=11%, signal=33%
GO_RESPONSE_TO_WOUNDING	GO_RESPONSE_TO_WOUNDING	454	0.4593799	2.1896794	0	0.00017	0.009	1259	tags=28%, list=12%, signal=30%
GO_ACUTE_PHASE_RESPONSE	GO_ACUTE_PHASE_RESPONSE	35	0.65536755	2.1895978	0	0.00017	0.009	983	tags=31%, list=9%, signal=35%
GO_REGULATION_OF_ALPHA_BETA_T_CELL_PROLIFERATION	GO_REGULATION_OF_ALPHA_BETA_T_CELL_PROLIFERATION	19	0.7567467	2.188365	0	0.00017	0.009	1750	tags=79%, list=17%, signal=95%
GO_POSITIVE_REGULATION_OF_CELL_CELL_ADHESION	GO_POSITIVE_REGULATION_OF_CELL_CELL_ADHESION	192	0.49367532	2.1880007	0	0.00017	0.009	1806	tags=43%, list=17%, signal=51%
GO_REGULATION_OF_VASCULATURE_DEVELOPMENT	GO_REGULATION_OF_VASCULATURE_DEVELOPMENT	193	0.48298043	2.1855	0	0.00017	0.009	1129	tags=26%, list=11%, signal=29%
GO_CELLULAR_RESPONSE_TO_NUTRIENT	GO_CELLULAR_RESPONSE_TO_NUTRIENT	36	0.6506602	2.1827881	0	0.00017	0.009	698	tags=33%, list=7%, signal=36%
GO_REGULATION_OF_INTERLEUKIN_8_PRODUCTION	GO_REGULATION_OF_INTERLEUKIN_8_PRODUCTION	46	0.605272	2.1819558	0	0.00016	0.009	1621	tags=39%, list=16%, signal=46%

GO_REGULATION_OF_VASCULAR_ENDOTHELIAL_GROWTH_FACTOR_PRODUCTION	GO_REGULATION_OF_VASCULAR_ENDOTHELIAL_GROWTH_FACTOR_PRODUCTION	27	0.6971863	2.1815047	0	0.00016	0.009	1186	tags=52%, list=11%, signal=58%
GO_POSITIVE_REGULATION_OF_MYELOID_LEUKOCYTE_MEDIATED_IMMUNITY	GO_POSITIVE_REGULATION_OF_MYELOID_LEUKOCYTE_MEDIATED_IMMUNITY	15	0.7937508	2.1798823	0	0.00016	0.009	405	tags=40%, list=4%, signal=42%
GO_POSITIVE_REGULATION_OF_VASOCONSTRICTION	GO_POSITIVE_REGULATION_OF_VASOCONSTRICTION	33	0.664958	2.1791239	0	0.00016	0.009	779	tags=30%, list=8%, signal=33%
GO_REGULATION_OF_LEUKOCYTE_PROLIFERATION	GO_REGULATION_OF_LEUKOCYTE_PROLIFERATION	160	0.4984756	2.1766517	0	0.00018	0.01	1766	tags=41%, list=17%, signal=49%
GO_POSITIVE_REGULATION_OF_LEUKOCYTE_DEGRANULATION	GO_POSITIVE_REGULATION_OF_LEUKOCYTE_DEGRANULATION	17	0.7673027	2.1758385	0	0.00017	0.01	505	tags=41%, list=5%, signal=43%
GO_T_CELL_MEDIATED_IMMUNITY	GO_T_CELL_MEDIATED_IMMUNITY	20	0.73654854	2.1732574	0	0.00017	0.01	1123	tags=50%, list=11%, signal=56%
GO_LYMPHOCYTE_MEDIATED_IMMUNITY	GO_LYMPHOCYTE_MEDIATED_IMMUNITY	85	0.54499125	2.170621	0	0.00017	0.01	1123	tags=32%, list=11%, signal=35%
GO_CELL_ACTIVATION	GO_CELL_ACTIVATION	451	0.4450407	2.167212	0	0.00019	0.011	1244	tags=29%, list=12%, signal=31%
GO_POSITIVE_REGULATION_OF_MAST_CELL_ACTIVATION	GO_POSITIVE_REGULATION_OF_MAST_CELL_ACTIVATION	15	0.7947616	2.166713	0	0.00018	0.011	1289	tags=60%, list=12%, signal=68%
GO_REGULATION_OF_HOMOTYPIC_CELL_CELL_ADHESION	GO_REGULATION_OF_HOMOTYPIC_CELL_CELL_ADHESION	235	0.46588778	2.1656888	0	0.00018	0.011	1470	tags=34%, list=14%, signal=38%
GO_REGULATION_OF_LYMPHOCYTE_MIGRATION	GO_REGULATION_OF_LYMPHOCYTE_MIGRATION	29	0.6628991	2.1612294	0	0.00023	0.014	1281	tags=48%, list=12%, signal=55%

GO_REGULATION_OF_MONOCYTE_CHEMOTAXIS	GO_REGULATION_OF_MONOCYTE_CHEMOTAXIS	19	0.75310415	2.1606784	0	0.00023	0.014	1543	tags=58%, list=15%, signal=68%
GO_LEUKOCYTE_ACTIVATION	GO_LEUKOCYTE_ACTIVATION	327	0.45559162	2.1587334	0	0.00025	0.015	1244	tags=30%, list=12%, signal=33%
GO_POSITIVE_REGULATION_OF_VASCULATURE_DEVELOPMENT	GO_POSITIVE_REGULATION_OF_VASCULATURE_DEVELOPMENT	110	0.53565776	2.1574223	0	0.00024	0.015	823	tags=24%, list=8%, signal=25%
GO_REGULATION_OF_GRANULOCYTE_CHEMOTAXIS	GO_REGULATION_OF_GRANULOCYTE_CHEMOTAXIS	35	0.64735585	2.1561441	0	0.00024	0.015	1549	tags=46%, list=15%, signal=54%
GO_NEGATIVE_REGULATION_OF_CHEMOTAXIS	GO_NEGATIVE_REGULATION_OF_CHEMOTAXIS	41	0.6253803	2.1537404	0	0.00026	0.016	1543	tags=41%, list=15%, signal=49%
GO_NEGATIVE_REGULATION_OF_PEPTIDASE_ACTIVITY	GO_NEGATIVE_REGULATION_OF_PEPTIDASE_ACTIVITY	167	0.48081896	2.1529694	0	0.00025	0.016	1383	tags=26%, list=13%, signal=30%
GO_RESPONSE_TO_VITAMIN_D	GO_RESPONSE_TO_VITAMIN_D	31	0.6599159	2.1526575	0	0.00027	0.017	986	tags=45%, list=9%, signal=50%
GO_POSITIVE_REGULATION_OF_LEUKOCYTE_DIFFERENTIATION	GO_POSITIVE_REGULATION_OF_LEUKOCYTE_DIFFERENTIATION	102	0.5264198	2.1519513	0	0.00027	0.017	1518	tags=39%, list=15%, signal=45%
GO_DENDRITIC_CELL_DIFFERENTIATION	GO_DENDRITIC_CELL_DIFFERENTIATION	28	0.66966647	2.1488695	0	0.00028	0.018	1200	tags=46%, list=12%, signal=52%
GO_POSITIVE_REGULATION_OF_ALPHA_BETA_T_CELL_DIFFERENTIATION	GO_POSITIVE_REGULATION_OF_ALPHA_BETA_T_CELL_DIFFERENTIATION	29	0.677718	2.1399245	0	0.00034	0.022	1470	tags=45%, list=14%, signal=52%
GO_REGULATION_OF_CYTOKINE_PRODUCTION	GO_REGULATION_OF_CYTOKINE_PRODUCTION	421	0.45112142	2.1251278	0	0.00049	0.032	1634	tags=35%, list=16%, signal=39%
GO_POSITIVE_REGULATION_OF_CELL_ADHESION	GO_POSITIVE_REGULATION_OF_CELL_ADHESION	290	0.45990336	2.1248436	0	0.00049	0.032	1806	tags=38%, list=17%, signal=45%

GO_POSITIVE_REGULATION_OF_ALPHA_BETA_T_CELL_PROLIFERATION	GO_POSITIVE_REGULATION_OF_ALPHA_BETA_T_CELL_PROLIFERATION	16	0.7790375	2.1243577	0.003086	0.00048	0.032	1750	tags=81%, list=17%, signal=98%
GO_REGULATION_OF_INTERLEUKIN_12_PRODUCTION	GO_REGULATION_OF_INTERLEUKIN_12_PRODUCTION	42	0.60900116	2.1233563	0	0.00049	0.033	1664	tags=50%, list=16%, signal=59%
GO_APOPTOTIC_CELL_CLEARANCE	GO_APOPTOTIC_CELL_CLEARANCE	23	0.71506625	2.123227	0	0.0005	0.034	1451	tags=57%, list=14%, signal=66%
GO_REGULATION_OF_SMOOTH_MUSCLE_CELL_MIGRATION	GO_REGULATION_OF_SMOOTH_MUSCLE_CELL_MIGRATION	44	0.60267836	2.1227596	0	0.0005	0.034	1634	tags=50%, list=16%, signal=59%
GO_LEUKOTRIENE_METABOLIC_PROCESS	GO_LEUKOTRIENE_METABOLIC_PROCESS	20	0.6983309	2.1210415	0	0.0005	0.034	893	tags=35%, list=9%, signal=38%
GO_REGULATION_OF_T_CELL_RECEPTOR_SIGNALING_PATHWAY	GO_REGULATION_OF_T_CELL_RECEPTOR_SIGNALING_PATHWAY	23	0.70185596	2.1192791	0	0.00054	0.037	1932	tags=52%, list=19%, signal=64%
GO_REGULATION_OF_CYTOKINE_BIOSYNTHETIC_PROCESS	GO_REGULATION_OF_CYTOKINE_BIOSYNTHETIC_PROCESS	77	0.54093546	2.116443	0	0.00053	0.037	1801	tags=45%, list=17%, signal=55%
GO_NEGATIVE_REGULATION_OF_CELL_ACTIVATION	GO_NEGATIVE_REGULATION_OF_CELL_ACTIVATION	117	0.50338566	2.1143894	0	0.00054	0.038	1338	tags=35%, list=13%, signal=40%
GO_IMMUNE_EFFECTOR_PROCESS	GO_IMMUNE_EFFECTOR_PROCESS	338	0.44792587	2.114352	0	0.00054	0.038	1539	tags=33%, list=15%, signal=38%
GO_HEMOSTASIS	GO_HEMOSTASIS	254	0.4577253	2.1097412	0	0.00055	0.039	1342	tags=26%, list=13%, signal=29%
GO_REGULATION_OF_MACROPHAGE_CHEMOTAXIS	GO_REGULATION_OF_MACROPHAGE_CHEMOTAXIS	15	0.7797989	2.1085353	0	0.00056	0.04	969	tags=47%, list=9%, signal=51%
GO_ACTIVATION_OF_INNATE_IMMUNE_RESPONSE	GO_ACTIVATION_OF_INNATE_IMMUNE_RESPONSE	163	0.48214787	2.107137	0	0.00058	0.042	1313	tags=32%, list=13%, signal=36%

GO_REGULATION_OF_ALPHA_BETA_T_CELL_DIFFERENTIATION	GO_REGULATION_OF_ALPHA_BETA_T_CELL_DIFFERENTIATION	36	0.6216449	2.1031013	0	0.00062	0.045	1470	tags=36%, list=14%, signal=42%
GO_CYTOKINE_PRODUCTION	GO_CYTOKINE_PRODUCTION	95	0.52034515	2.1030314	0	0.00062	0.045	1055	tags=33%, list=10%, signal=36%
GO_REGULATION_OF_INTERFERON_GAMMA_PRODUCTION	GO_REGULATION_OF_INTERFERON_GAMMA_PRODUCTION	66	0.5502917	2.100044	0	0.00061	0.045	1862	tags=50%, list=18%, signal=61%
GO_VASCULATURE_DEVELOPMENT	GO_VASCULATURE_DEVELOPMENT	377	0.44324976	2.0964072	0	0.00061	0.045	1048	tags=24%, list=10%, signal=26%
GO_REGULATION_OF_LEUKOCYTE_DIFFERENTIATION	GO_REGULATION_OF_LEUKOCYTE_DIFFERENTIATION	174	0.46948442	2.0956023	0	0.00061	0.045	1539	tags=36%, list=15%, signal=42%
GO_REGULATION_OF_HUMORAL_IMMUNE_RESPONSE	GO_REGULATION_OF_HUMORAL_IMMUNE_RESPONSE	40	0.61629766	2.0942512	0	0.0006	0.045	686	tags=28%, list=7%, signal=29%
GO_MEMBRANE_RAFT_ORGANIZATION	GO_MEMBRANE_RAFT_ORGANIZATION	16	0.7599371	2.093992	0	0.0006	0.045	1350	tags=56%, list=13%, signal=65%
GO_CHEMOKINE_MEDIATED_SIGNALING_PATHWAY	GO_CHEMOKINE_MEDIATED_SIGNALING_PATHWAY	52	0.5720279	2.0924165	0	0.00062	0.047	2209	tags=52%, list=21%, signal=66%
GO_REGULATION_OF_PROTEIN_ACTIVATION_CASCADE	GO_REGULATION_OF_PROTEIN_ACTIVATION_CASCADE	30	0.6532235	2.092332	0	0.00062	0.047	946	tags=37%, list=9%, signal=40%
GO_PHAGOCYTOSIS_ENGULFMENT	GO_PHAGOCYTOSIS_ENGULFMENT	20	0.7091888	2.091396	0	0.00061	0.047	1265	tags=50%, list=12%, signal=57%
GO_T_CELL_SELECTION	GO_T_CELL_SELECTION	31	0.65923023	2.0897307	0	0.00062	0.048	1862	tags=48%, list=18%, signal=59%
GO_ALPHA_BETA_T_CELL_ACTIVATION	GO_ALPHA_BETA_T_CELL_ACTIVATION	43	0.5868452	2.0884695	0	0.00063	0.049	1236	tags=40%, list=12%, signal=45%
GO_POSITIVE_REGULATION_OF_NEUTROPHIL_MIGRATION	GO_POSITIVE_REGULATION_OF_NEUTROPHIL_MIGRATION	24	0.69288397	2.0817792	0	0.00069	0.054	828	tags=42%, list=8%, signal=45%

GO_REGULATION_OF_METANEPHROS_DEVELOPMENT	GO_REGULATION_OF_METANEPHROS_DEVELOPMENT	20	0.7111652	2.0743575	0	0.0008	0.062	2151	tags=55%, list=21%, signal=69%
GO_LEUKOCYTE_MEDIATED_IMMUNITY	GO_LEUKOCYTE_MEDIATED_IMMUNITY	119	0.4978913	2.0732563	0	0.00082	0.064	1176	tags=33%, list=11%, signal=37%
GO_POSITIVE_REGULATION_OF_CELL_ADHESION_MEDIATED_BY_INTEGRIN	GO_POSITIVE_REGULATION_OF_CELL_ADHESION_MEDIATED_BY_INTEGRIN	15	0.7486103	2.0702882	0	0.00083	0.065	941	tags=33%, list=9%, signal=37%
GO_POSITIVE_REGULATION_OF_HEMOPOIESIS	GO_POSITIVE_REGULATION_OF_HEMOPOIESIS	125	0.4893641	2.0695865	0	0.00084	0.066	1533	tags=36%, list=15%, signal=42%
GO_MONOCYTE_CHEMOTAXIS	GO_MONOCYTE_CHEMOTAXIS	30	0.63269407	2.0689979	0	0.00085	0.068	2163	tags=50%, list=21%, signal=63%
GO_O_GLYCAN_PROCESSING	GO_O_GLYCAN_PROCESSING	33	0.6078198	2.0688589	0	0.00085	0.068	2047	tags=48%, list=20%, signal=60%
GO_POSITIVE_REGULATION_OF_REACTIVE_OXYGEN_SPECIES_METABOLIC_PROCESS	GO_POSITIVE_REGULATION_OF_REACTIVE_OXYGEN_SPECIES_METABOLIC_PROCESS	74	0.536023	2.0683744	0	0.00084	0.068	1288	tags=34%, list=12%, signal=38%
GO_REGULATION_OF_ENDOTHELIAL_CELL_APOPTOTIC_PROCESS	GO_REGULATION_OF_ENDOTHELIAL_CELL_APOPTOTIC_PROCESS	36	0.6061927	2.0682073	0	0.00084	0.068	781	tags=28%, list=8%, signal=30%
GO_REACTIVE_OXYGEN_SPECIES_BIOSYNTHETIC_PROCESS	GO_REACTIVE_OXYGEN_SPECIES_BIOSYNTHETIC_PROCESS	22	0.69525474	2.0631518	0	0.00092	0.075	1984	tags=59%, list=19%, signal=73%
GO_POSITIVE_REGULATION_OF_LYMPHOCYTE_DIFFERENTIATION	GO_POSITIVE_REGULATION_OF_LYMPHOCYTE_DIFFERENTIATION	63	0.54057485	2.059476	0	0.00097	0.08	1470	tags=44%, list=14%, signal=51%
GO_REGULATION_OF_CELL_CELL_ADHESION	GO_REGULATION_OF_CELL_CELL_ADHESION	292	0.4424357	2.0582917	0	0.00098	0.081	1487	tags=32%, list=14%, signal=36%
GO_REGULATION_OF_NEUTROPHIL_MIGRATION	GO_REGULATION_OF_NEUTROPHIL_MIGRATION	28	0.6577534	2.0577343	0	0.00099	0.082	1549	tags=50%, list=15%, signal=59%

GO_REGULATION_OF_B_CELL_PROLIFERATION	GO_REGULATION_OF_B_CELL_PROLIFERATION	42	0.59457684	2.055882	0	0.00101	0.084	1756	tags=40%, list=17%, signal=49%
GO_REGULATION_OF_IMMUNE_EFFECTOR_PROCESS	GO_REGULATION_OF_IMMUNE_EFFECTOR_PROCESS	316	0.43707108	2.0540385	0	0.00104	0.087	1446	tags=31%, list=14%, signal=35%
GO_MATERNAL_PROCESS_INVOLVED_IN_FEMALE_PREGNANCY	GO_MATERNAL_PROCESS_INVOLVED_IN_FEMALE_PREGNANCY	50	0.5545938	2.052933	0	0.00107	0.09	1762	tags=46%, list=17%, signal=55%
GO_BLOOD_VESSEL_MORPHOGENESIS	GO_BLOOD_VESSEL_MORPHOGENESIS	299	0.4380764	2.0505476	0	0.00108	0.092	1048	tags=26%, list=10%, signal=28%
GO_REGULATION_OF_B_CELL_ACTIVATION	GO_REGULATION_OF_B_CELL_ACTIVATION	81	0.5219614	2.049887	0	0.00109	0.093	1469	tags=36%, list=14%, signal=41%
GO_FATTY_ACID_DERIVATIVE_METABOLIC_PROCESS	GO_FATTY_ACID_DERIVATIVE_METABOLIC_PROCESS	65	0.5305207	2.046292	0	0.0012	0.102	1078	tags=28%, list=10%, signal=31%
GO_LYMPHOCYTE_ACTIVATION	GO_LYMPHOCYTE_ACTIVATION	271	0.43813032	2.046089	0	0.00119	0.102	1244	tags=28%, list=12%, signal=31%
GO_MYELOID_DENDRITIC_CELL_ACTIVATION	GO_MYELOID_DENDRITIC_CELL_ACTIVATION	20	0.7054088	2.0442927	0	0.00118	0.102	1440	tags=50%, list=14%, signal=58%
GO_CELL_SUBSTRATE_ADHESION	GO_CELL_SUBSTRATE_ADHESION	126	0.48512205	2.0421004	0	0.00122	0.104	1220	tags=33%, list=12%, signal=36%
GO_CELL_ACTIVATION_INVOLVED_IN_IMMUNE_RESPONSE	GO_CELL_ACTIVATION_INVOLVED_IN_IMMUNE_RESPONSE	102	0.4989541	2.0413234	0	0.00123	0.105	1234	tags=36%, list=12%, signal=41%
GO_REGULATION_OF_REACTIVE_OXYGEN_SPECIES_METABOLIC_PROCESS	GO_REGULATION_OF_REACTIVE_OXYGEN_SPECIES_METABOLIC_PROCESS	125	0.48778406	2.0407119	0	0.00123	0.106	1288	tags=30%, list=12%, signal=34%
GO_NEGATIVE_REGULATION_OF_IMMUNE_SYSTEM_PROCESS	GO_NEGATIVE_REGULATION_OF_IMMUNE_SYSTEM_PROCESS	271	0.43602344	2.0352323	0	0.00133	0.115	1543	tags=34%, list=15%, signal=39%
GO_RESPONSE_TO_OXYGEN_LEVELS	GO_RESPONSE_TO_OXYGEN_LEVELS	265	0.43220618	2.0332267	0	0.00134	0.117	1035	tags=25%, list=10%, signal=27%

GO_LEUKOCYTE_PROLIFERATION	GO_LEUKOCYTE_PROLIFERATION	68	0.53965485	2.0302742	0	0.0014	0.121	1342	tags=32%, list=13%, signal=37%
GO_ORGAN_REGENERATION	GO_ORGAN_REGENERATION	73	0.51824313	2.0218549	0	0.00158	0.135	801	tags=27%, list=8%, signal=29%
GO_REGULATION_OF_EPITHELIAL_CELL_APOPTOTIC_PROCESS	GO_REGULATION_OF_EPITHELIAL_CELL_APOPTOTIC_PROCESS	51	0.55140644	2.0203776	0	0.00162	0.139	1216	tags=33%, list=12%, signal=38%
GO_CYTOKINE_MEDIATED_SIGNALING_PATHWAY	GO_CYTOKINE_MEDIATED_SIGNALING_PATHWAY	343	0.42731607	2.017115	0	0.00167	0.143	1602	tags=32%, list=15%, signal=37%
GO_ICOSANOID_METABOLIC_PROCESS	GO_ICOSANOID_METABOLIC_PROCESS	65	0.5305207	2.0169513	0	0.00166	0.143	1078	tags=28%, list=10%, signal=31%
GO_NEGATIVE_REGULATION_OF_ANTIGEN_RECEPTOR_MEDIATED_SIGNALING_PATHWAY	GO_NEGATIVE_REGULATION_OF_ANTIGEN_RECEPTOR_MEDIATED_SIGNALING_PATHWAY	16	0.71719307	2.0108993	0	0.00181	0.154	1620	tags=50%, list=16%, signal=59%
GO_MEMBRANE_INVAGINATION	GO_MEMBRANE_INVAGINATION	26	0.63408	2.0079992	0	0.00184	0.157	1394	tags=42%, list=13%, signal=49%
GO_CELLULAR_RESPONSE_TO_AMINO_ACID_STIMULUS	GO_CELLULAR_RESPONSE_TO_AMINO_ACID_STIMULUS	38	0.60308576	2.004998	0	0.00194	0.165	1061	tags=39%, list=10%, signal=44%
GO_POSITIVE_REGULATION_OF_INTERLEUKIN_8_PRODUCTION	GO_POSITIVE_REGULATION_OF_INTERLEUKIN_8_PRODUCTION	36	0.6177364	2.003689	0	0.00197	0.167	1055	tags=31%, list=10%, signal=34%
GO_REGULATION_OF_ENDOCYTOSIS	GO_REGULATION_OF_ENDOCYTOSIS	156	0.45839006	2.0022507	0	0.00205	0.175	1451	tags=33%, list=14%, signal=38%
GO_LYMPHOCYTE_ACTIVATION_INVOLVED_IN_IMMUNE_RESPONSE	GO_LYMPHOCYTE_ACTIVATION_INVOLVED_IN_IMMUNE_RESPONSE	69	0.5258441	2.0010285	0	0.0021	0.179	1200	tags=32%, list=12%, signal=36%
GO_FIBRIL_ORGANIZATION	GO_FIBRIL_ORGANIZATION	17	0.70852023	2.0006478	0	0.00211	0.181	431	tags=29%, list=4%, signal=31%
GO_POSITIVE_REGULATION_OF_NF_KAPPAB_IMPORT_INTO_NUCLEUS	GO_POSITIVE_REGULATION_OF_NF_KAPPAB_IMPORT_INTO_NUCLEUS	21	0.6815643	2.0003834	0.003205	0.0021	0.181	1175	tags=48%, list=11%, signal=54%

GO_INTERACTION_WITH_HOST	GO_INTERACTION_WITH_HOST	112	0.48254266	1.9995843	0	0.0021	0.182	1053	tags=31%, list=10%, signal=34%
GO_REGULATION_OF_CYTOKINE_PRODUCTION_INVOLVED_IN_IMMUNE_RESPONSE	GO_REGULATION_OF_CYTOKINE_PRODUCTION_INVOLVED_IN_IMMUNE_RESPONSE	47	0.5633877	1.9988483	0	0.00209	0.183	2005	tags=51%, list=19%, signal=63%
GO_UNSATURATED_FATTY_ACID_METABOLIC_PROCESS	GO_UNSATURATED_FATTY_ACID_METABOLIC_PROCESS	76	0.5073387	1.9988468	0	0.00208	0.183	893	tags=24%, list=9%, signal=26%
GO_POSITIVE_REGULATION_OF_INTERFERON_GAMMA_PRODUCTION	GO_POSITIVE_REGULATION_OF_INTERFERON_GAMMA_PRODUCTION	46	0.5681669	1.9971799	0	0.00213	0.188	1440	tags=48%, list=14%, signal=55%
GO_ENDODERM_FORMATION	GO_ENDODERM_FORMATION	39	0.5824765	1.9947864	0	0.00224	0.198	1462	tags=49%, list=14%, signal=56%
GO_REGULATION_OF_NEUTROPHIL_CHEMOTAXIS	GO_REGULATION_OF_NEUTROPHIL_CHEMOTAXIS	23	0.6583686	1.9935744	0.003155	0.00227	0.203	1862	tags=61%, list=18%, signal=74%
GO_NEGATIVE_REGULATION_OF_CELL_CELL_ADHESION	GO_NEGATIVE_REGULATION_OF_CELL_CELL_ADHESION	103	0.479227	1.9920374	0	0.00226	0.203	1330	tags=30%, list=13%, signal=34%
GO_REGULATION_OF_NITRIC_OXIDE_BIOSYNTHETIC_PROCESS	GO_REGULATION_OF_NITRIC_OXIDE_BIOSYNTHETIC_PROCESS	46	0.5606892	1.9920207	0	0.00225	0.203	1250	tags=37%, list=12%, signal=42%
GO_REGULATION_OF_MONONUCLEAR_CELL_MIGRATION	GO_REGULATION_OF_MONONUCLEAR_CELL_MIGRATION	15	0.7481987	1.9913723	0	0.00226	0.205	969	tags=40%, list=9%, signal=44%
GO_REGULATION_OF_ERK1_AND_ERK2_CASCADE	GO_REGULATION_OF_ERK1_AND_ERK2_CASCADE	177	0.45111233	1.9894997	0	0.00226	0.206	1451	tags=33%, list=14%, signal=38%
GO_PLATELET_ACTIVATION	GO_PLATELET_ACTIVATION	124	0.47148085	1.9882607	0	0.0023	0.209	1342	tags=30%, list=13%, signal=34%

GO_REGULATION_OF_MULTICELLULAR_ORGANISMAL_METABOLIC_PROCESS	GO_REGULATION_OF_MULTICELLULAR_ORGANISMAL_METABOLIC_PROCESS	31	0.6048383	1.9874163	0	0.00232	0.211	1811	tags=52%, list=17%, signal=62%
GO_RESPONSE_TO_INTERFERON_GAMMA	GO_RESPONSE_TO_INTERFERON_GAMMA	104	0.47162917	1.9851116	0	0.00234	0.214	1741	tags=41%, list=17%, signal=49%
GO_ICOSANOID_BIOSYNTHETIC_PROCESS	GO_ICOSANOID_BIOSYNTHETIC_PROCESS	30	0.618519	1.9830528	0	0.00242	0.221	474	tags=30%, list=5%, signal=31%
GO_REGULATION_OF_BODY_FLUID_LEVELS	GO_REGULATION_OF_BODY_FLUID_LEVELS	402	0.4211726	1.981815	0	0.00245	0.223	1342	tags=23%, list=13%, signal=26%
GO_POSITIVE_REGULATION_OF_INNATE_IMMUNE_RESPONSE	GO_POSITIVE_REGULATION_OF_INNATE_IMMUNE_RESPONSE	195	0.43933252	1.9806007	0	0.00247	0.226	1313	tags=30%, list=13%, signal=33%
GO_POSITIVE_REGULATION_OF_CYTOKINE_SECRETION	GO_POSITIVE_REGULATION_OF_CYTOKINE_SECRETION	73	0.51484936	1.9786412	0	0.00251	0.23	1053	tags=30%, list=10%, signal=33%
GO_LEUKOCYTE_DIFFERENTIATION	GO_LEUKOCYTE_DIFFERENTIATION	234	0.42724657	1.975007	0	0.00272	0.25	1290	tags=29%, list=12%, signal=32%
GO_RESPONSE_TO_FLUID_SHEAR_STRESS	GO_RESPONSE_TO_FLUID_SHEAR_STRESS	32	0.5983521	1.9675058	0	0.003	0.269	706	tags=28%, list=7%, signal=30%
GO_REGULATION_OF_PEPTIDASE_ACTIVITY	GO_REGULATION_OF_PEPTIDASE_ACTIVITY	285	0.42544544	1.9651701	0	0.0031	0.276	1164	tags=22%, list=11%, signal=24%
GO_FATTY_ACID_DERIVATIVE_BIOSYNTHETIC_PROCESS	GO_FATTY_ACID_DERIVATIVE_BIOSYNTHETIC_PROCESS	30	0.6185188	1.964738	0	0.0031	0.277	474	tags=30%, list=5%, signal=31%
GO_CELLULAR_EXTRAVASATION	GO_CELLULAR_EXTRAVASATION	21	0.6607251	1.9589635	0	0.00336	0.295	1048	tags=38%, list=10%, signal=42%
GO_MRNA_TRANSCRIPTION	GO_MRNA_TRANSCRIPTION	17	0.69459647	1.9578713	0.003086	0.0034	0.301	2224	tags=59%, list=21%, signal=75%
GO_EMBRYO_IMPLANTATION	GO_EMBRYO_IMPLANTATION	32	0.580351	1.9572437	0	0.00345	0.306	1193	tags=38%, list=11%, signal=42%

GO_NEGATIVE_REGULATION_OF_PEPTIDYL_TYROSINE_PHOSPHORYLATION	GO_NEGATIVE_REGULATION_OF_PEPTIDYL_TYROSINE_PHOSPHORYLATION	28	0.61427677	1.9572134	0	0.00344	0.307	908	tags=36%, list=9%, signal=39%
GO_MYELOID_DENDRITIC_CELL_DIFFERENTIATION	GO_MYELOID_DENDRITIC_CELL_DIFFERENTIATION	15	0.71919066	1.9570048	0	0.00342	0.307	1200	tags=47%, list=12%, signal=53%
GO_REGULATION_OF_CELL_ADHESION	GO_REGULATION_OF_CELL_ADHESION	486	0.41528857	1.956107	0	0.00345	0.311	1491	tags=30%, list=14%, signal=34%
GO_POSITIVE_REGULATION_OF_SMOOTH_MUSCLE_CELL_MIGRATION	GO_POSITIVE_REGULATION_OF_SMOOTH_MUSCLE_CELL_MIGRATION	25	0.6281074	1.9533316	0	0.00357	0.324	1196	tags=36%, list=12%, signal=41%
GO_CELL_CELL_ADHESION	GO_CELL_CELL_ADHESION	437	0.40196812	1.952332	0	0.0036	0.329	1289	tags=26%, list=12%, signal=28%
GO_REGULATION_OF_PRODUCTION_OF_MOLECULAR_MEDIATOR_OF_IMMUNE_RESPONSE	GO_REGULATION_OF_PRODUCTION_OF_MOLECULAR_MEDIATOR_OF_IMMUNE_RESPONSE	83	0.48960966	1.9520384	0	0.00362	0.33	1801	tags=40%, list=17%, signal=48%
GO_PARTURITION	GO_PARTURITION	17	0.6893065	1.9457134	0.003106	0.00389	0.352	828	tags=35%, list=8%, signal=38%
GO_TAXIS	GO_TAXIS	350	0.4142069	1.9450771	0	0.00392	0.355	1502	tags=30%, list=14%, signal=34%
GO_BRANCHING_INVOLVED_IN_SALIVARY_GLAND_MORPHOGENESIS	GO_BRANCHING_INVOLVED_IN_SALIVARY_GLAND_MORPHOGENESIS	15	0.7097984	1.9444948	0	0.00395	0.36	1603	tags=53%, list=15%, signal=63%
GO_T_CELL_DIFFERENTIATION	GO_T_CELL_DIFFERENTIATION	101	0.47208375	1.9443594	0	0.00394	0.361	1244	tags=31%, list=12%, signal=35%
GO_T_CELL_DIFFERENTIATION_INVOLVED_IN_IMMUNE_RESPONSE	GO_T_CELL_DIFFERENTIATION_INVOLVED_IN_IMMUNE_RESPONSE	20	0.67630017	1.941891	0	0.00406	0.373	1200	tags=45%, list=12%, signal=51%
GO_REGULATION_OF_MACROPHAGE_DERIVED_FOAM_CELL_DIFFERENTIATION	GO_REGULATION_OF_MACROPHAGE_DERIVED_FOAM_CELL_DIFFERENTIATION	27	0.6141072	1.9414265	0	0.00407	0.375	1313	tags=48%, list=13%, signal=55%

GO_UNSATURATED_FATTY_ACID_BIOSYNTHETIC_PROCESS	GO_UNSATURATED_FATTY_ACID_BIOSYNTHETIC_PROCESS	40	0.5799682	1.9403728	0	0.0041	0.377	474	tags=25%, list=5%, signal=26%
GO_T_CELL_PROLIFERATION	GO_T_CELL_PROLIFERATION	29	0.61783373	1.9339614	0	0.00441	0.404	1236	tags=41%, list=12%, signal=47%
GO_CELL_REDOX_HOMEOSTASIS	GO_CELL_REDOX_HOMEOSTASIS	39	0.56012213	1.9307202	0	0.00453	0.413	1343	tags=38%, list=13%, signal=44%
GO_POSITIVE_REGULATION_OF_TISSUE_REMODELING	GO_POSITIVE_REGULATION_OF_TISSUE_REMODELING	21	0.65934086	1.9301527	0	0.00455	0.415	1751	tags=43%, list=17%, signal=51%
GO_REGULATION_OF_INTERLEUKIN_1_PRODUCTION	GO_REGULATION_OF_INTERLEUKIN_1_PRODUCTION	39	0.5629625	1.9289968	0	0.00458	0.419	1290	tags=44%, list=12%, signal=50%
GO_HUMORAL_IMMUNE_RESPONSE	GO_HUMORAL_IMMUNE_RESPONSE	117	0.4706873	1.9288085	0	0.00458	0.42	1470	tags=26%, list=14%, signal=31%
GO_REGULATION_OF_ADAPTIVE_IMMUNE_RESPONSE	GO_REGULATION_OF_ADAPTIVE_IMMUNE_RESPONSE	99	0.46057913	1.9253997	0	0.00473	0.428	1539	tags=37%, list=15%, signal=43%
GO_REGULATION_OF_INTERLEUKIN_10_PRODUCTION	GO_REGULATION_OF_INTERLEUKIN_10_PRODUCTION	28	0.6045189	1.9229702	0.003509	0.00487	0.439	2064	tags=57%, list=20%, signal=71%
GO_CYTOKINE_SECRETION	GO_CYTOKINE_SECRETION	28	0.5963902	1.9184287	0	0.00507	0.455	1203	tags=36%, list=12%, signal=40%
GO_LEUKOCYTE_MEDIATED_CYTOTOXICITY	GO_LEUKOCYTE_MEDIATED_CYTOTOXICITY	23	0.63711697	1.914255	0.007067	0.00524	0.466	1502	tags=52%, list=14%, signal=61%
GO_REGULATION_OF_TYPE_2_IMMUNE_RESPONSE	GO_REGULATION_OF_TYPE_2_IMMUNE_RESPONSE	19	0.6740277	1.9142481	0.003145	0.00523	0.467	1992	tags=68%, list=19%, signal=85%
GO_REGULATION_OF_REACTIVE_OXYGEN_SPECIES_BIOSYNTHETIC_PROCESS	GO_REGULATION_OF_REACTIVE_OXYGEN_SPECIES_BIOSYNTHETIC_PROCESS	55	0.5298948	1.9139818	0	0.00525	0.469	1250	tags=36%, list=12%, signal=41%
GO_RESPONSE_TO_PROTOZOAN	GO_RESPONSE_TO_PROTOZOAN	18	0.6826713	1.9134822	0	0.00524	0.471	502	tags=33%, list=5%, signal=35%

GO_REGULATION_OF_HEMOPOIESIS	GO_REGULATION_OF_HEMOPOIESIS	223	0.42248622	1.9082447	0	0.00564	0.499	1539	tags=31%, list=15%, signal=36%
GO_NEGATIVE_REGULATION_OF_EXTRINSIC_APOPTOTIC_SIGNALING_PATHWAY_VIA_DEATH_DOMAIN_RECEPTORS	GO_NEGATIVE_REGULATION_OF_EXTRINSIC_APOPTOTIC_SIGNALING_PATHWAY_VIA_DEATH_DOMAIN_RECEPTORS	29	0.5965674	1.9075885	0	0.00564	0.502	1980	tags=55%, list=19%, signal=68%
GO_NEGATIVE_REGULATION_OF_INTERFERON_GAMMA_PRODUCTION	GO_NEGATIVE_REGULATION_OF_INTERFERON_GAMMA_PRODUCTION	22	0.62933594	1.9008818	0.006601	0.00607	0.529	2397	tags=64%, list=23%, signal=83%
GO_LYMPHOCYTE_COSTIMULATION	GO_LYMPHOCYTE_COSTIMULATION	58	0.5182162	1.8986917	0	0.0062	0.536	1853	tags=48%, list=18%, signal=58%
GO_REGULATION_OF_VASOCONSTRICTION	GO_REGULATION_OF_VASOCONSTRICTION	59	0.50622386	1.8952547	0	0.00645	0.549	779	tags=22%, list=8%, signal=24%
GO_ENDOCYTOSIS	GO_ENDOCYTOSIS	365	0.4025428	1.8910726	0	0.00674	0.564	1142	tags=25%, list=11%, signal=27%
GO_NEUTROPHIL_MEDIATED_IMMUNITY	GO_NEUTROPHIL_MEDIATED_IMMUNITY	18	0.65060943	1.8888398	0.006309	0.00685	0.572	1686	tags=56%, list=16%, signal=66%
GO_CYTOKINE_METABOLIC_PROCESS	GO_CYTOKINE_METABOLIC_PROCESS	15	0.70441794	1.8876666	0.003021	0.00694	0.577	1750	tags=53%, list=17%, signal=64%
GO_CELLULAR_RESPONSE_TO_CYTOKINE_STIMULUS	GO_CELLULAR_RESPONSE_TO_CYTOKINE_STIMULUS	457	0.39625576	1.8837861	0	0.00717	0.586	1546	tags=30%, list=15%, signal=33%
GO_CHOLESTEROL_EFFLUX	GO_CHOLESTEROL_EFFLUX	22	0.6229031	1.8828588	0	0.00719	0.589	747	tags=32%, list=7%, signal=34%
GO_B_CELL_PROLIFERATION	GO_B_CELL_PROLIFERATION	27	0.6038981	1.8807176	0.003322	0.00734	0.601	1342	tags=30%, list=13%, signal=34%
GO_B_CELL_MEDIATED_IMMUNITY	GO_B_CELL_MEDIATED_IMMUNITY	54	0.52430576	1.8806661	0	0.00731	0.601	946	tags=30%, list=9%, signal=32%
GO_RECEPTOR_MEDIATED_ENDOCYTOSIS	GO_RECEPTOR_MEDIATED_ENDOCYTOSIS	159	0.4293022	1.8787652	0	0.00739	0.607	986	tags=23%, list=9%, signal=25%
GO_REGULATION_OF_INNATE_IMMUNE_RESPONSE	GO_REGULATION_OF_INNATE_IMMUNE_RESPONSE	271	0.40260452	1.8742859	0	0.00782	0.625	1546	tags=31%, list=15%, signal=35%

GO_NEGATIVE_REGULATION_OF_LEUKOCYTE_MEDIATED_IMMUNITY	GO_NEGATIVE_REGULATION_OF_LEUKOCYTE_MEDIATED_IMMUNITY	38	0.5554307	1.87396	0	0.00781	0.626	1446	tags=47%, list=14%, signal=55%
GO_OVULATION	GO_OVULATION	15	0.6968249	1.873664	0.006289	0.00779	0.626	417	tags=40%, list=4%, signal=42%
GO_REGULATION_OF_PLASMA_LIPOPROTEIN_PARTICLE_LEVELS	GO_REGULATION_OF_PLASMA_LIPOPROTEIN_PARTICLE_LEVELS	37	0.5543328	1.8720995	0.003861	0.0079	0.631	1026	tags=38%, list=10%, signal=42%
GO_LYMPHOCYTE_MIGRATION	GO_LYMPHOCYTE_MIGRATION	34	0.5638995	1.8712443	0	0.00798	0.634	2472	tags=56%, list=24%, signal=73%
GO_CELLULAR_RESPONSE_TO_EXTERNAL_STIMULUS	GO_CELLULAR_RESPONSE_TO_EXTERNAL_STIMULUS	213	0.40793324	1.8705626	0	0.00802	0.635	1066	tags=29%, list=10%, signal=31%
GO_DEFENSE_RESPONSE_TO_OTHER_ORGANISM	GO_DEFENSE_RESPONSE_TO_OTHER_ORGANISM	306	0.40154228	1.8703909	0	0.00801	0.635	1539	tags=30%, list=15%, signal=34%
GO_POSITIVE_REGULATION_OF_LEUKOCYTE_MEDIATED_IMMUNITY	GO_POSITIVE_REGULATION_OF_LEUKOCYTE_MEDIATED_IMMUNITY	66	0.49851984	1.8702931	0	0.00799	0.635	1801	tags=41%, list=17%, signal=49%
GO_REGULATION_OF_MACROPHAGE_ACTIVATION	GO_REGULATION_OF_MACROPHAGE_ACTIVATION	15	0.69646627	1.8683352	0.002915	0.00815	0.644	1434	tags=40%, list=14%, signal=46%
GO_REGULATION_OF_CELLULAR_EXTRAVASATION	GO_REGULATION_OF_CELLULAR_EXTRAVASATION	19	0.64893955	1.8665304	0.00304	0.00824	0.647	580	tags=37%, list=6%, signal=39%
GO_POSITIVE_REGULATION_OF_PRODUCTION_OF_MOLECULAR_MEDIATOR_OF_IMMUNE_RESPONSE	GO_POSITIVE_REGULATION_OF_PRODUCTION_OF_MOLECULAR_MEDIATOR_OF_IMMUNE_RESPONSE	51	0.5149654	1.8665172	0	0.00821	0.647	1801	tags=45%, list=17%, signal=54%
GO_POSITIVE_REGULATION_OF_B_CELL_ACTIVATION	GO_POSITIVE_REGULATION_OF_B_CELL_ACTIVATION	53	0.51523733	1.8658532	0	0.00824	0.651	1469	tags=34%, list=14%, signal=39%

GO_NEGATIVE_REGULATION_OF_LOCOMOTION	GO_NEGATIVE_REGULATION_OF_LOCOMOTION	208	0.41229677	1.8656226	0	0.00822	0.652	1648	tags=31%, list=16%, signal=36%
GO_NEGATIVE_REGULATION_OF_ALPHA_BETA_T_CELL_ACTIVATION	GO_NEGATIVE_REGULATION_OF_ALPHA_BETA_T_CELL_ACTIVATION	20	0.62642884	1.8626012	0	0.00847	0.668	1330	tags=40%, list=13%, signal=46%
GO_ALPHA_BETA_T_CELL_DIFFERENTIATION	GO_ALPHA_BETA_T_CELL_DIFFERENTIATION	35	0.5520638	1.8625861	0.003509	0.00844	0.668	1200	tags=34%, list=12%, signal=39%
GO_NEGATIVE_REGULATION_OF_RESPONSE_TO_CYTOKINE_STIMULUS	GO_NEGATIVE_REGULATION_OF_RESPONSE_TO_CYTOKINE_STIMULUS	30	0.5735441	1.8622134	0.003448	0.00843	0.668	1341	tags=43%, list=13%, signal=50%
GO_RESPONSE_TO_HYPEROXIA	GO_RESPONSE_TO_HYPEROXIA	22	0.61550206	1.8609276	0	0.00856	0.674	1224	tags=41%, list=12%, signal=46%
GO_PLASMA_LIPOPROTEIN_PARTICLE_CLEARANCE	GO_PLASMA_LIPOPROTEIN_PARTICLE_CLEARANCE	18	0.6663442	1.8560036	0.002994	0.00893	0.694	948	tags=39%, list=9%, signal=43%
GO_REGULATION_OF_EXTRINSIC_APOPTOTIC_SIGNALING_PATHWAY_VIA_DEATH_DOMAIN_RECEPTORS	GO_REGULATION_OF_EXTRINSIC_APOPTOTIC_SIGNALING_PATHWAY_VIA_DEATH_DOMAIN_RECEPTORS	46	0.52353495	1.854086	0	0.00903	0.7	1066	tags=26%, list=10%, signal=29%
GO_MULTI_MULTICELLULAR_ORGANISM_PROCESS	GO_MULTI_MULTICELLULAR_ORGANISM_PROCESS	183	0.41958153	1.8519957	0	0.00923	0.708	1073	tags=22%, list=10%, signal=24%
GO_RESPONSE_TO_FUNGUS	GO_RESPONSE_TO_FUNGUS	35	0.5422391	1.8518177	0	0.00921	0.71	634	tags=31%, list=6%, signal=33%
GO_POSITIVE_REGULATION_OF_INTERLEUKIN_10_PRODUCTION	GO_POSITIVE_REGULATION_OF_INTERLEUKIN_10_PRODUCTION	19	0.64513546	1.8514907	0	0.00919	0.711	2064	tags=63%, list=20%, signal=79%
GO_OSTEOCLAST_DIFFERENTIATION	GO_OSTEOCLAST_DIFFERENTIATION	25	0.6103864	1.8511422	0.002985	0.00921	0.714	1527	tags=36%, list=15%, signal=42%
GO_REGULATION_OF_T_CELL_MIGRATION	GO_REGULATION_OF_T_CELL_MIGRATION	20	0.6242963	1.8498411	0	0.00927	0.721	1053	tags=40%, list=10%, signal=44%
GO_PROTEIN_TRIMERIZATION	GO_PROTEIN_TRIMERIZATION	27	0.57734853	1.847022	0	0.00956	0.73	483	tags=19%, list=5%, signal=19%

GO_POSITIVE_REGULATION_OF_ACUTE_INFLAMMATORY_RESPONSE	GO_POSITIVE_REGULATION_OF_ACUTE_INFLAMMATORY_RESPONSE	23	0.60436183	1.8436056	0.006369	0.00979	0.74	1238	tags=52%, list=12%, signal=59%
GO_REGULATION_OF_PLATELET_ACTIVATION	GO_REGULATION_OF_PLATELET_ACTIVATION	28	0.58257437	1.8435858	0	0.00975	0.74	1055	tags=32%, list=10%, signal=36%
GO_REGULATION_OF_LIPOPOLYSACCHARIDE_MEDIATED_SIGNALING_PATHWAY	GO_REGULATION_OF_LIPOPOLYSACCHARIDE_MEDIATED_SIGNALING_PATHWAY	15	0.6802014	1.8423098	0	0.00983	0.743	588	tags=40%, list=6%, signal=42%
GO_NEGATIVE_REGULATION_OF_IMMUNE_EFFECTOR_PROCESS	GO_NEGATIVE_REGULATION_OF_IMMUNE_EFFECTOR_PROCESS	81	0.47041616	1.8418078	0	0.00983	0.744	1243	tags=37%, list=12%, signal=42%
GO_POSITIVE_REGULATION_OF_REACTIVE_OXYGEN_SPECIES_BIOSYNTHETIC_PROCESS	GO_POSITIVE_REGULATION_OF_REACTIVE_OXYGEN_SPECIES_BIOSYNTHETIC_PROCESS	42	0.53483546	1.8409646	0	0.00987	0.747	786	tags=29%, list=8%, signal=31%
GO_ACTIN_CYTOSKELETON_REORGANIZATION	GO_ACTIN_CYTOSKELETON_REORGANIZATION	40	0.53342646	1.8400611	0	0.00991	0.749	1466	tags=40%, list=14%, signal=46%
GO_LYMPHOCYTE_CHEMOTAXIS	GO_LYMPHOCYTE_CHEMOTAXIS	26	0.5768873	1.8384221	0	0.01007	0.755	1741	tags=46%, list=17%, signal=55%
GO_REGULATION_OF_TOLL_LIKE_RECEPTOR_SIGNALING_PATHWAY	GO_REGULATION_OF_TOLL_LIKE_RECEPTOR_SIGNALING_PATHWAY	33	0.55499685	1.8365306	0	0.01018	0.762	1539	tags=45%, list=15%, signal=53%
GO_REGULATION_OF_MEMBRANE_PROTEIN_ECTODOMAIN_PROTEOLYSIS	GO_REGULATION_OF_MEMBRANE_PROTEIN_ECTODOMAIN_PROTEOLYSIS	18	0.6417774	1.8349911	0	0.01036	0.768	1216	tags=44%, list=12%, signal=50%
GO_AMINOGLYCAN_CATABOLIC_PROCESS	GO_AMINOGLYCAN_CATABOLIC_PROCESS	50	0.5153948	1.8318797	0.003953	0.0106	0.773	935	tags=38%, list=9%, signal=42%
GO_DECIDUALIZATION	GO_DECIDUALIZATION	18	0.6435916	1.8318483	0.003185	0.01057	0.773	652	tags=33%, list=6%, signal=36%
GO_HYALURONAN_METABOLIC_PROCESS	GO_HYALURONAN_METABOLIC_PROCESS	26	0.5727606	1.8254764	0	0.01137	0.8	1808	tags=50%, list=17%, signal=60%

GO_NEGATIVE_REGULATION_OF_WOUND_HEALING	GO_NEGATIVE_REGULATION_OF_WOUND_HEALING	53	0.50026524	1.8253433	0	0.01136	0.801	494	tags=17%, list=5%, signal=18%
GO_REGULATION_OF_RESPONSE_TO_CYTOKINE_STIMULUS	GO_REGULATION_OF_RESPONSE_TO_CYTOKINE_STIMULUS	106	0.4401809	1.8229258	0	0.01161	0.817	1486	tags=31%, list=14%, signal=36%
GO_RESPONSE_TO_HEAT	GO_RESPONSE_TO_HEAT	68	0.4751022	1.8217111	0	0.01173	0.82	1113	tags=28%, list=11%, signal=31%
GO_EPITHELIAL_CELL_MORPHOGENESIS	GO_EPITHELIAL_CELL_MORPHOGENESIS	35	0.54356724	1.8204798	0	0.0118	0.823	1143	tags=34%, list=11%, signal=38%
GO_REGULATION_OF_PEPTIDYL_TYROSINE_PHOSPHORYLATION	GO_REGULATION_OF_PEPTIDYL_TYROSINE_PHOSPHORYLATION	172	0.42033574	1.8201339	0	0.01177	0.823	1246	tags=29%, list=12%, signal=32%
GO_REGULATION_OF_EXTRINSIC_APOPTOTIC_SIGNALING_PATHWAY	GO_REGULATION_OF_EXTRINSIC_APOPTOTIC_SIGNALING_PATHWAY	128	0.42535406	1.8200561	0	0.01174	0.823	1073	tags=23%, list=10%, signal=25%
GO_POSITIVE_T_CELL_SELECTION	GO_POSITIVE_T_CELL_SELECTION	17	0.6625905	1.8198873	0	0.0117	0.824	745	tags=29%, list=7%, signal=32%
GO_REGULATION_OF_LYMPHOCYTE_DIFFERENTIATION	GO_REGULATION_OF_LYMPHOCYTE_DIFFERENTIATION	100	0.44463834	1.819151	0	0.01173	0.825	1470	tags=35%, list=14%, signal=40%
GO_HOMOTYPIC_CELL_CELL_ADHESION	GO_HOMOTYPIC_CELL_CELL_ADHESION	42	0.5109988	1.817341	0	0.01199	0.831	1099	tags=31%, list=11%, signal=34%
GO_REGULATION_OF_SYSTEMIC_ARTERIAL_BLOOD_PRESSURE_MEDIATED_BY_A_CHEMICAL_SIGNAL	GO_REGULATION_OF_SYSTEMIC_ARTERIAL_BLOOD_PRESSURE_MEDIATED_BY_A_CHEMICAL_SIGNAL	42	0.519753	1.8173337	0.003534	0.01195	0.831	2557	tags=45%, list=25%, signal=60%
GO_HYDROGEN_PEROXIDE_METABOLIC_PROCESS	GO_HYDROGEN_PEROXIDE_METABOLIC_PROCESS	23	0.6033069	1.8169962	0	0.01193	0.833	1247	tags=30%, list=12%, signal=35%
GO_DEFENSE_RESPONSE_TO_GRAM_NEGATIVE_BACTERIUM	GO_DEFENSE_RESPONSE_TO_GRAM_NEGATIVE_BACTERIUM	33	0.562359	1.8149345	0	0.01213	0.84	560	tags=30%, list=5%, signal=32%

GO_CD4_POSITIVE_ALPHA_BETA_T_CELL_ACTIVATION	GO_CD4_POSITIVE_ALPHA_BETA_T_CELL_ACTIVATION	25	0.5877195	1.813486	0.006757	0.01228	0.843	1200	tags=40%, list=12%, signal=45%
GO_THYMIC_T_CELL_SELECTION	GO_THYMIC_T_CELL_SELECTION	18	0.63220745	1.8124764	0.003077	0.01237	0.845	2212	tags=50%, list=21%, signal=63%
GO_MEMBRANE_LIPID_CATABOLIC_PROCESS	GO_MEMBRANE_LIPID_CATABOLIC_PROCESS	18	0.63146466	1.8116028	0.003067	0.01241	0.845	1075	tags=44%, list=10%, signal=49%
GO_PLATELET_AGGREGATION	GO_PLATELET_AGGREGATION	33	0.53680074	1.8114945	0.003344	0.0124	0.845	1099	tags=36%, list=11%, signal=41%
GO_NEGATIVE_REGULATION_OF_HYDROLASE_ACTIVITY	GO_NEGATIVE_REGULATION_OF_HYDROLASE_ACTIVITY	263	0.394038	1.8111821	0	0.01238	0.845	1290	tags=23%, list=12%, signal=25%
GO_RESPONSE_TO_MAGNESIUM_ION	GO_RESPONSE_TO_MAGNESIUM_ION	19	0.62993914	1.8109969	0.003086	0.01235	0.845	230	tags=21%, list=2%, signal=21%
GO_PEPTIDE_CATABOLIC_PROCESS	GO_PEPTIDE_CATABOLIC_PROCESS	17	0.6465153	1.8102928	0	0.01241	0.847	1920	tags=53%, list=18%, signal=65%
GO_NEGATIVE_REGULATION_OF_CELL_ADHESION	GO_NEGATIVE_REGULATION_OF_CELL_ADHESION	177	0.41372305	1.8084728	0	0.01255	0.856	1244	tags=27%, list=12%, signal=30%
GO_REGULATION_OF_T_CELL_DIFFERENTIATION	GO_REGULATION_OF_T_CELL_DIFFERENTIATION	82	0.45591137	1.8061147	0	0.01276	0.862	1801	tags=39%, list=17%, signal=47%
GO_HUMORAL_IMMUNE_RESPONSE_MEDIATED_BY_CIRCULATING_IMMUNOGLOBULIN	GO_HUMORAL_IMMUNE_RESPONSE_MEDIATED_BY_CIRCULATING_IMMUNOGLOBULIN	31	0.5642894	1.8058053	0.007168	0.01275	0.865	946	tags=29%, list=9%, signal=32%
GO_NECROPTOTIC_PROCESS	GO_NECROPTOTIC_PROCESS	16	0.65867394	1.8057426	0.012121	0.01272	0.866	1055	tags=31%, list=10%, signal=35%
GO_NEGATIVE_REGULATION_OF_HOMOTYPIC_CELL_CELL_ADHESION	GO_NEGATIVE_REGULATION_OF_HOMOTYPIC_CELL_CELL_ADHESION	75	0.46230993	1.8048271	0	0.01278	0.871	1539	tags=33%, list=15%, signal=39%
GO_REGULATION_OF_T_CELL_MEDIATED_IMMUNITY	GO_REGULATION_OF_T_CELL_MEDIATED_IMMUNITY	40	0.53652793	1.8040466	0.003636	0.01283	0.874	2064	tags=45%, list=20%, signal=56%

GO_RESPONSE_TO_MECHANICAL_STIMULUS	GO_RESPONSE_TO_MECHANICAL_STIMULUS	173	0.408911	1.8015223	0	0.01303	0.877	1356	tags=34%, list=13%, signal=39%
GO_NEGATIVE_REGULATION_OF_RESPONSE_TO_WOUNDING	GO_NEGATIVE_REGULATION_OF_RESPONSE_TO_WOUNDING	125	0.43271512	1.8005856	0	0.01315	0.883	1847	tags=35%, list=18%, signal=42%
GO_SUBSTRATE_ADHESION_DEPENDENT_CELL_SPREADING	GO_SUBSTRATE_ADHESION_DEPENDENT_CELL_SPREADING	27	0.58888036	1.7979298	0.006969	0.0134	0.889	781	tags=37%, list=8%, signal=40%
GO_RESPONSE_TO_CAMP	GO_RESPONSE_TO_CAMP	89	0.4472861	1.797781	0.004762	0.01337	0.889	725	tags=18%, list=7%, signal=19%
GO_RECEPTOR_INTERNALIZATION	GO_RECEPTOR_INTERNALIZATION	44	0.513074	1.7948328	0	0.01379	0.897	1288	tags=32%, list=12%, signal=36%
GO_NEGATIVE_REGULATION_OF_EPITHELIAL_CELL_APOPTOTIC_PROCESS	GO_NEGATIVE_REGULATION_OF_EPITHELIAL_CELL_APOPTOTIC_PROCESS	31	0.55627245	1.7947983	0.003311	0.01375	0.897	1216	tags=35%, list=12%, signal=40%
GO_REGULATION_OF_CD4_POSITIVE_ALPHA_BETA_T_CELL_ACTIVATION	GO_REGULATION_OF_CD4_POSITIVE_ALPHA_BETA_T_CELL_ACTIVATION	29	0.5581438	1.7945064	0	0.01374	0.897	2139	tags=52%, list=21%, signal=65%
GO_RESPONSE_TO_INCREASED_OXYGEN_LEVELS	GO_RESPONSE_TO_INCREASED_OXYGEN_LEVELS	22	0.61550206	1.7937412	0.006579	0.01378	0.899	1224	tags=41%, list=12%, signal=46%
GO_CELL_SUBSTRATE_JUNCTION_ASSEMBLY	GO_CELL_SUBSTRATE_JUNCTION_ASSEMBLY	35	0.54295653	1.792587	0	0.01387	0.901	2185	tags=54%, list=21%, signal=69%
GO_REGULATION_OF_ALCOHOL_BIOSYNTHETIC_PROCESS	GO_REGULATION_OF_ALCOHOL_BIOSYNTHETIC_PROCESS	40	0.52827513	1.7924701	0	0.01383	0.901	1487	tags=38%, list=14%, signal=44%
GO_REGULATION_OF_NF_KAPPAB_IMPORT_INTO_NUCLEUS	GO_REGULATION_OF_NF_KAPPAB_IMPORT_INTO_NUCLEUS	38	0.5206977	1.7924231	0	0.01379	0.901	1216	tags=37%, list=12%, signal=42%
GO_CELLULAR_TRANSITION_METAL_ION_HOMEOSTASIS	GO_CELLULAR_TRANSITION_METAL_ION_HOMEOSTASIS	52	0.49442077	1.7909819	0	0.01391	0.904	1059	tags=25%, list=10%, signal=28%

GO_RESPONSE_TO_VITAMIN	GO_RESPONSE_TO_VITAMIN	95	0.44399872	1.7880708	0	0.01425	0.908	1236	tags=31%, list=12%, signal=34%
GO_POSITIVE_REGULATION_OF_PROTEIN_KINASE_B_SIGNALING	GO_POSITIVE_REGULATION_OF_PROTEIN_KINASE_B_SIGNALING	66	0.4700333	1.7868156	0.004505	0.01438	0.91	1343	tags=32%, list=13%, signal=36%
GO_RESPONSE_TO_PROGESTERONE	GO_RESPONSE_TO_PROGESTERONE	39	0.53148615	1.7855089	0	0.01446	0.913	658	tags=28%, list=6%, signal=30%
GO_POSITIVE_REGULATION_OF_I_KAPPAB_KINASE_NF_KAPPAB_SIGNALING	GO_POSITIVE_REGULATION_OF_I_KAPPAB_KINASE_NF_KAPPAB_SIGNALING	136	0.41748402	1.7823745	0	0.01484	0.921	1453	tags=29%, list=14%, signal=33%
GO_ENDODERM_DEVELOPMENT	GO_ENDODERM_DEVELOPMENT	56	0.46748155	1.7792131	0.004348	0.01526	0.923	1462	tags=41%, list=14%, signal=48%
GO_NEGATIVE_REGULATION_OF_PROTEIN_MATURATION	GO_NEGATIVE_REGULATION_OF_PROTEIN_MATURATION	27	0.55299455	1.7770838	0.013746	0.01556	0.928	946	tags=33%, list=9%, signal=37%
GO_POSITIVE_REGULATION_OF_ERK1_AND_ERK2_CASCADE	GO_POSITIVE_REGULATION_OF_ERK1_AND_ERK2_CASCADE	128	0.41502962	1.7765955	0	0.01557	0.93	1451	tags=30%, list=14%, signal=34%
GO_NEGATIVE_REGULATION_OF_LEUKOCYTE_PROLIFERATION	GO_NEGATIVE_REGULATION_OF_LEUKOCYTE_PROLIFERATION	53	0.48947567	1.7760397	0.004367	0.01564	0.932	1751	tags=42%, list=17%, signal=50%
GO_RESPONSE_TO_ORGANOPHOSPHORUS	GO_RESPONSE_TO_ORGANOPHOSPHORUS	115	0.4262972	1.7727299	0	0.01608	0.939	735	tags=18%, list=7%, signal=19%
GO_REGULATION_OF_ANTIGEN_PROCESSING_AND_PRESENTATION	GO_REGULATION_OF_ANTIGEN_PROCESSING_AND_PRESENTATION	19	0.62462497	1.7718941	0.003135	0.01621	0.939	1434	tags=47%, list=14%, signal=55%
GO_CELLULAR_RESPONSE_TO_ACID_CHEMICAL	GO_CELLULAR_RESPONSE_TO_ACID_CHEMICAL	136	0.40716684	1.7702852	0	0.01646	0.943	1061	tags=24%, list=10%, signal=27%
GO_LYSOSOME_ORGANIZATION	GO_LYSOSOME_ORGANIZATION	32	0.53531814	1.7687734	0.003367	0.01661	0.943	1182	tags=34%, list=11%, signal=39%
GO_N_GLYCAN_PROCESSING	GO_N_GLYCAN_PROCESSING	17	0.623855	1.7684329	0.00578	0.0166	0.945	762	tags=35%, list=7%, signal=38%

GO_NEGATIVE_REGULATION_OF_ENDOTHELIAL_CELL_APOPTOTIC_PROCESS	GO_NEGATIVE_REGULATION_OF_ENDOTHELIAL_CELL_APOPTOTIC_PROCESS	25	0.5816519	1.7663511	0.003279	0.01683	0.949	1216	tags=36%, list=12%, signal=41%
GO_POSITIVE_REGULATION_OF_B_CELL_PROLIFERATION	GO_POSITIVE_REGULATION_OF_B_CELL_PROLIFERATION	28	0.5624224	1.7644793	0.007092	0.01708	0.95	1469	tags=32%, list=14%, signal=37%
GO_CELLULAR_RESPONSE_TO_INTERLEUKIN_1	GO_CELLULAR_RESPONSE_TO_INTERLEUKIN_1	65	0.46104306	1.7635528	0	0.01713	0.952	1836	tags=37%, list=18%, signal=45%
GO_PHAGOSOME_MATURATION	GO_PHAGOSOME_MATURATION	25	0.54598045	1.7619292	0	0.01728	0.955	1182	tags=32%, list=11%, signal=36%
GO_ARACHIDONIC_ACID_METABOLIC_PROCESS	GO_ARACHIDONIC_ACID_METABOLIC_PROCESS	38	0.51255286	1.7607127	0.008333	0.0174	0.956	474	tags=21%, list=5%, signal=22%
GO_FORMATION_OF_PRIMARY_GERM_LAYER	GO_FORMATION_OF_PRIMARY_GERM_LAYER	88	0.4484699	1.7602441	0	0.01745	0.956	1621	tags=39%, list=16%, signal=45%
GO_REGULATION_OF_MYOBLAST_FUSION	GO_REGULATION_OF_MYOBLAST_FUSION	17	0.6335281	1.7601756	0.006494	0.01742	0.956	770	tags=29%, list=7%, signal=32%
GO_EMBRYONIC_PLACENTA_MORPHOGENESIS	GO_EMBRYONIC_PLACENTA_MORPHOGENESIS	19	0.61140966	1.7591059	0.006734	0.01753	0.957	1288	tags=42%, list=12%, signal=48%
GO_POSITIVE_REGULATION_OF_STAT_CASCADE	GO_POSITIVE_REGULATION_OF_STAT_CASCADE	61	0.46654215	1.7579285	0.00813	0.01768	0.958	1342	tags=31%, list=13%, signal=36%
GO_CELLULAR_RESPONSE_TO_MECHANICAL_STIMULUS	GO_CELLULAR_RESPONSE_TO_MECHANICAL_STIMULUS	73	0.4594158	1.756709	0	0.01777	0.958	1342	tags=41%, list=13%, signal=47%
GO_CELLULAR_RESPONSE_TO_EXTRACELLULAR_STIMULUS	GO_CELLULAR_RESPONSE_TO_EXTRACELLULAR_STIMULUS	144	0.40934855	1.7566758	0	0.01772	0.958	1061	tags=26%, list=10%, signal=29%
GO_ENDODERMAL_CELL_DIFFERENTIATION	GO_ENDODERMAL_CELL_DIFFERENTIATION	32	0.54018706	1.7556756	0.008065	0.01787	0.96	1462	tags=47%, list=14%, signal=54%
GO_POSITIVE_REGULATION_OF_INTERLEUKIN_12_PRODUCTION	GO_POSITIVE_REGULATION_OF_INTERLEUKIN_12_PRODUCTION	28	0.54260325	1.7555146	0.003717	0.01785	0.96	2223	tags=57%, list=21%, signal=73%
GO_I_KAPPAB_KINASE_NF_KAPPAB_SIGNALING	GO_I_KAPPAB_KINASE_NF_KAPPAB_SIGNALING	48	0.49202046	1.7548121	0.003937	0.01794	0.96	1538	tags=35%, list=15%, signal=41%

GO_POSITIVE_REGULATION_OF_TRANSCRIPTION_FACTOR_IMPORT_INTO_NUCLEUS	GO_POSITIVE_REGULATION_OF_TRANSCRIPTION_FACTOR_IMPORT_INTO_NUCLEUS	40	0.51355207	1.7542794	0.00823	0.01796	0.96	1314	tags=38%, list=13%, signal=43%
GO_LIPID_STORAGE	GO_LIPID_STORAGE	17	0.6237085	1.7541796	0.015873	0.01793	0.96	803	tags=35%, list=8%, signal=38%
GO_NEGATIVE_REGULATION_OF_PROTEIN_PROCESSING	GO_NEGATIVE_REGULATION_OF_PROTEIN_PROCESSING	27	0.5529948	1.7532496	0.009494	0.018	0.961	946	tags=33%, list=9%, signal=37%
GO_CELLULAR_RESPONSE_TO_CARBOHYDRATE_STIMULUS	GO_CELLULAR_RESPONSE_TO_CARBOHYDRATE_STIMULUS	63	0.46517846	1.7525004	0	0.01805	0.961	889	tags=29%, list=9%, signal=31%
GO_PEPTIDYL_TYROSINE_AUTOPHOSPHORYLATION	GO_PEPTIDYL_TYROSINE_AUTOPHOSPHORYLATION	36	0.51605725	1.7514352	0.003546	0.01813	0.963	978	tags=25%, list=9%, signal=28%
GO_REGULATION_OF_INSULIN_LIKE_GROWTH_FACTOR_RECEPTOR_SIGNALING_PATHWAY	GO_REGULATION_OF_INSULIN_LIKE_GROWTH_FACTOR_RECEPTOR_SIGNALING_PATHWAY	20	0.5999502	1.7509766	0.003185	0.01817	0.963	1095	tags=30%, list=11%, signal=33%
GO_POSITIVE_REGULATION_OF_ADAPTIVE_IMMUNE_RESPONSE	GO_POSITIVE_REGULATION_OF_ADAPTIVE_IMMUNE_RESPONSE	58	0.472513	1.75087	0	0.01812	0.963	1801	tags=43%, list=17%, signal=52%
GO_SEMAPHORIN_PLEXIN_SIGNALING_PATHWAY	GO_SEMAPHORIN_PLEXIN_SIGNALING_PATHWAY	33	0.53147674	1.750562	0	0.01812	0.963	1603	tags=36%, list=15%, signal=43%
GO_CELLULAR_RESPONSE_TO_KETONE	GO_CELLULAR_RESPONSE_TO_KETONE	59	0.4757856	1.7499714	0.014706	0.01816	0.963	734	tags=24%, list=7%, signal=25%
GO_NEGATIVE_REGULATION_OF_LEUKOCYTE_APOPTOTIC_PROCESS	GO_NEGATIVE_REGULATION_OF_LEUKOCYTE_APOPTOTIC_PROCESS	33	0.53829557	1.7492709	0.007576	0.01821	0.963	2005	tags=52%, list=19%, signal=64%
GO_NEGATIVE_REGULATION_OF_LIPID_STORAGE	GO_NEGATIVE_REGULATION_OF_LIPID_STORAGE	17	0.645683	1.7468628	0.003268	0.01864	0.966	1296	tags=41%, list=12%, signal=47%

GO_LYMPHOCYTE_DIFFERENTIATION	GO_LYMPHOCYTE_DIFFERENTIATION	165	0.39603147	1.7454734	0	0.0188	0.966	1244	tags=26%, list=12%, signal=29%
GO_POSITIVE_REGULATION_OF_EPITHELIAL_CELL_APOPTOTIC_PROCESS	GO_POSITIVE_REGULATION_OF_EPITHELIAL_CELL_APOPTOTIC_PROCESS	20	0.58946514	1.7454325	0.003497	0.01875	0.966	412	tags=25%, list=4%, signal=26%
GO_REGULATION_OF_LYMPHOCYTE_CHEMOTAXIS	GO_REGULATION_OF_LYMPHOCYTE_CHEMOTAXIS	15	0.63436097	1.7432715	0.011142	0.01907	0.968	1741	tags=40%, list=17%, signal=48%
GO_REGULATION_OF_CELLULAR_RESPONSE_TO_TRANSFORMING_GROWTH_FACTOR_BETA_STIMULUS	GO_REGULATION_OF_CELLULAR_RESPONSE_TO_TRANSFORMING_GROWTH_FACTOR_BETA_STIMULUS	74	0.44942102	1.7431144	0	0.01906	0.968	1187	tags=27%, list=11%, signal=30%
GO_POSITIVE_REGULATION_OF_CD4_POSITIVE_ALPHA_BETA_T_CELL_ACTIVATION	GO_POSITIVE_REGULATION_OF_CD4_POSITIVE_ALPHA_BETA_T_CELL_ACTIVATION	21	0.60514325	1.7428503	0.006536	0.01907	0.968	1470	tags=48%, list=14%, signal=55%
GO_NEGATIVE_REGULATION_OF_ENDOTHELIAL_CELL_PROLIFERATION	GO_NEGATIVE_REGULATION_OF_ENDOTHELIAL_CELL_PROLIFERATION	26	0.55735534	1.7418374	0.011494	0.01922	0.968	681	tags=23%, list=7%, signal=25%
GO_REGULATION_OF_MACROPHAGE_DIFFERENTIATION	GO_REGULATION_OF_MACROPHAGE_DIFFERENTIATION	17	0.64404297	1.7408425	0	0.01934	0.968	969	tags=41%, list=9%, signal=45%
GO_RESPONSE_TO_PURINE_CONTAINING_COMPOUND	GO_RESPONSE_TO_PURINE_CONTAINING_COMPOUND	134	0.40891704	1.7396646	0	0.01951	0.971	735	tags=19%, list=7%, signal=20%
GO_ENDOTHELIAL_CELL_MIGRATION	GO_ENDOTHELIAL_CELL_MIGRATION	50	0.48236957	1.7392019	0.004464	0.01951	0.971	1141	tags=32%, list=11%, signal=36%
GO_REGULATION_OF_T_HELPER_CELL_DIFFERENTIATION	GO_REGULATION_OF_T_HELPER_CELL_DIFFERENTIATION	19	0.60351676	1.7385368	0.00639	0.01955	0.973	825	tags=26%, list=8%, signal=29%

GO_NEGATIVE_REGULATION_OF_CYTOKINE_PRODUCTION	GO_NEGATIVE_REGULATION_OF_CYTOKINE_PRODUCTION	146	0.4071111	1.7381731	0	0.01955	0.974	1330	tags=27%, list=13%, signal=31%
GO_RESPONSE_TO_NUTRIENT	GO_RESPONSE_TO_NUTRIENT	177	0.39713866	1.7378871	0	0.01957	0.974	1241	tags=25%, list=12%, signal=28%
GO_REGULATION_OF_LEUKOCYTE_APOPTOTIC_PROCESS	GO_REGULATION_OF_LEUKOCYTE_APOPTOTIC_PROCESS	57	0.47231272	1.7366937	0.004505	0.01972	0.978	1862	tags=42%, list=18%, signal=51%
GO_REGULATION_OF_NECROTIC_CELL_DEATH	GO_REGULATION_OF_NECROTIC_CELL_DEATH	23	0.58817977	1.7363869	0.009967	0.01972	0.978	1954	tags=48%, list=19%, signal=59%
GO_POSITIVE_REGULATION_OF_EXTRINSIC_APOPTOTIC_SIGNALING_PATHWAY	GO_POSITIVE_REGULATION_OF_EXTRINSIC_APOPTOTIC_SIGNALING_PATHWAY	47	0.48739955	1.7361224	0	0.0197	0.978	801	tags=23%, list=8%, signal=25%
GO_PROTEIN_DEGLYCOSYLATION	GO_PROTEIN_DEGLYCOSYLATION	17	0.6262094	1.7357008	0.010067	0.01974	0.978	1627	tags=47%, list=16%, signal=56%
GO_NEGATIVE_REGULATION_OF_EXTRINSIC_APOPTOTIC_SIGNALING_PATHWAY	GO_NEGATIVE_REGULATION_OF_EXTRINSIC_APOPTOTIC_SIGNALING_PATHWAY	81	0.44328722	1.7337106	0	0.01997	0.98	1073	tags=25%, list=10%, signal=27%
GO_REGULATION_OF_TYROSINE_PHOSPHORYLATION_OF_STAT_PROTEIN	GO_REGULATION_OF_TYROSINE_PHOSPHORYLATION_OF_STAT_PROTEIN	55	0.47601283	1.7291704	0	0.02083	0.984	1342	tags=35%, list=13%, signal=39%
GO_SUBSTRATE_DEPENDENT_CELL_MIGRATION	GO_SUBSTRATE_DEPENDENT_CELL_MIGRATION	23	0.5717612	1.7272708	0.018182	0.0211	0.986	1427	tags=39%, list=14%, signal=45%
GO_MYELOID_LEUKOCYTE_DIFFERENTIATION	GO_MYELOID_LEUKOCYTE_DIFFERENTIATION	77	0.44136724	1.7260529	0	0.02132	0.988	1290	tags=31%, list=12%, signal=35%
GO_NEGATIVE_REGULATION_OF_CYTOKINE_PRODUCTION_INVOLVED_IN_IMMUNE_RESPONSE	GO_NEGATIVE_REGULATION_OF_CYTOKINE_PRODUCTION_INVOLVED_IN_IMMUNE_RESPONSE	20	0.5806063	1.7251768	0.009524	0.02148	0.99	2005	tags=50%, list=19%, signal=62%

GO_PROSTAGLANDIN_METABOLIC_PROCESS	GO_PROSTAGLANDIN_METABOLIC_PROCESS	20	0.59294	1.7243587	0.006944	0.02161	0.99	426	tags=30%, list=4%, signal=31%
GO_PARASYMPATHETIC_NERVOUS_SYSTEM_DEVELOPMENT	GO_PARASYMPATHETIC_NERVOUS_SYSTEM_DEVELOPMENT	15	0.6358863	1.7234187	0.003125	0.0217	0.99	2242	tags=53%, list=22%, signal=68%
GO_NEGATIVE_REGULATION_OF_ERK1_AND_ERK2_CASCADE	GO_NEGATIVE_REGULATION_OF_ERK1_AND_ERK2_CASCADE	38	0.505112	1.7223842	0.012	0.02184	0.99	1178	tags=39%, list=11%, signal=44%
GO_NEGATIVE_REGULATION_OF_SMOOTH_MUSCLE_CELL_MIGRATION	GO_NEGATIVE_REGULATION_OF_SMOOTH_MUSCLE_CELL_MIGRATION	16	0.6188013	1.7221961	0.006667	0.02181	0.99	1341	tags=56%, list=13%, signal=64%
GO_POSITIVE_REGULATION_OF_JAK_STAT_CASCADE	GO_POSITIVE_REGULATION_OF_JAK_STAT_CASCADE	61	0.46654215	1.7204124	0	0.02214	0.991	1342	tags=31%, list=13%, signal=36%
GO_POSITIVE_REGULATION_OF_LIPID_CATABOLIC_PROCESS	GO_POSITIVE_REGULATION_OF_LIPID_CATABOLIC_PROCESS	21	0.5787721	1.7180244	0	0.02252	0.992	1133	tags=29%, list=11%, signal=32%
GO_NEGATIVE_REGULATION_OF_RESPONSE_TO_EXTERNAL_STIMULUS	GO_NEGATIVE_REGULATION_OF_RESPONSE_TO_EXTERNAL_STIMULUS	214	0.38327038	1.716362	0	0.02285	0.994	1496	tags=26%, list=14%, signal=30%
GO_REGENERATION	GO_REGENERATION	137	0.40032938	1.7142804	0	0.02327	0.994	1208	tags=28%, list=12%, signal=31%
GO_OLIGOSACCHARIDE_METABOLIC_PROCESS	GO_OLIGOSACCHARIDE_METABOLIC_PROCESS	50	0.47857946	1.712196	0	0.02364	0.994	1209	tags=34%, list=12%, signal=38%
GO_REGULATION_OF_SYSTEMIC_ARTERIAL_BLOOD_PRESSURE_BY_RENIN_ANGIOTENSIN	GO_REGULATION_OF_SYSTEMIC_ARTERIAL_BLOOD_PRESSURE_BY_RENIN_ANGIOTENSIN	21	0.57011926	1.7115602	0.016949	0.02367	0.995	2466	tags=57%, list=24%, signal=75%
GO_RESPONSE_TO_INTERLEUKIN_1	GO_RESPONSE_TO_INTERLEUKIN_1	88	0.42622203	1.7109257	0.005128	0.02375	0.995	1762	tags=34%, list=17%, signal=41%
GO_ODONTOGENESIS	GO_ODONTOGENESIS	82	0.43011415	1.7108356	0	0.02369	0.995	839	tags=22%, list=8%, signal=24%

GO_CELLULAR_RESPONSE_TO_FLUID_SHEAR_STRESS	GO_CELLULAR_RESPONSE_TO_FLUID_SHEAR_STRESS	18	0.6050163	1.710617	0.009288	0.02366	0.995	298	tags=17%, list=3%, signal=17%
GO_REGULATION_OF_I_KAPPAB_KINASE_NF_KAPPAB_SIGNALING	GO_REGULATION_OF_I_KAPPAB_KINASE_NF_KAPPAB_SIGNALING	176	0.38282335	1.7091894	0	0.02384	0.995	1216	tags=25%, list=12%, signal=28%
GO_BONE_RESORPTION	GO_BONE_RESORPTION	18	0.59363186	1.7090442	0.006349	0.02381	0.995	389	tags=22%, list=4%, signal=23%
GO_VITAMIN_TRANSPORT	GO_VITAMIN_TRANSPORT	26	0.5420724	1.7088606	0.003289	0.02379	0.995	947	tags=23%, list=9%, signal=25%
GO_LYTIC_VACUOLE_ORGANIZATION	GO_LYTIC_VACUOLE_ORGANIZATION	32	0.53531814	1.7069455	0.003497	0.02419	0.995	1182	tags=34%, list=11%, signal=39%
GO_CELLULAR_RESPONSE_TO_HEAT	GO_CELLULAR_RESPONSE_TO_HEAT	24	0.5538368	1.7050471	0	0.02457	0.996	328	tags=25%, list=3%, signal=26%
GO_REGULATION_OF_CARBOHYDRATE_BIOSYNTHETIC_PROCESS	GO_REGULATION_OF_CARBOHYDRATE_BIOSYNTHETIC_PROCESS	53	0.47277802	1.703485	0	0.02484	0.996	1440	tags=32%, list=14%, signal=37%
GO_TISSUE_REMODELING	GO_TISSUE_REMODELING	72	0.4478503	1.7034419	0	0.02479	0.996	1093	tags=28%, list=11%, signal=31%
GO_REGULATION_OF_TRANSFORMING_GROWTH_FACTOR_BETA_RECEPTOR_SIGNALING_PATHWAY	GO_REGULATION_OF_TRANSFORMING_GROWTH_FACTOR_BETA_RECEPTOR_SIGNALING_PATHWAY	74	0.44942084	1.7031195	0	0.02481	0.996	1187	tags=27%, list=11%, signal=30%
GO_POSITIVE_REGULATION_OF_MYELOID_LEUKOCYTE_DIFFERENTIATION	GO_POSITIVE_REGULATION_OF_MYELOID_LEUKOCYTE_DIFFERENTIATION	38	0.5025463	1.702581	0.003247	0.02487	0.996	2240	tags=45%, list=22%, signal=57%
GO_ESTROUS_CYCLE	GO_ESTROUS_CYCLE	18	0.6140273	1.7016536	0.009404	0.02499	0.996	825	tags=22%, list=8%, signal=24%
GO_PROSTANOID_METABOLIC_PROCESS	GO_PROSTANOID_METABOLIC_PROCESS	20	0.59294	1.7014788	0.018462	0.025	0.996	426	tags=30%, list=4%, signal=31%

GO_REGULATION_OF_PRI_MIRNA_TRANSCRIPTION_FROM_RNA_POLYMERASE_II_PROMOTER	GO_REGULATION_OF_PRI_MIRNA_TRANSCRIPTION_FROM_RNA_POLYMERASE_II_PROMOTER	16	0.5978768	1.7011116	0.009615	0.02505	0.996	1762	tags=56%, list=17%, signal=68%
GO_EXCRETION	GO_EXCRETION	39	0.5057974	1.6989546	0	0.02542	0.996	2791	tags=46%, list=27%, signal=63%
GO_NECROTIC_CELL_DEATH	GO_NECROTIC_CELL_DEATH	22	0.55423886	1.6986269	0.016026	0.02543	0.996	1980	tags=41%, list=19%, signal=50%
GO_REGULATION_OF_CELL_SUBSTRATE_ADHESION	GO_REGULATION_OF_CELL_SUBSTRATE_ADHESION	132	0.40405303	1.6986265	0	0.02537	0.996	1689	tags=28%, list=16%, signal=33%
GO_NEGATIVE_REGULATION_OF_EMBRYONIC_DEVELOPMENT	GO_NEGATIVE_REGULATION_OF_EMBRYONIC_DEVELOPMENT	22	0.5643392	1.6973294	0.00365	0.0256	0.996	656	tags=27%, list=6%, signal=29%
GO_REACTIVE_OXYGEN_SPECIES_METABOLIC_PROCESS	GO_REACTIVE_OXYGEN_SPECIES_METABOLIC_PROCESS	75	0.4436665	1.6923732	0	0.0266	0.996	1247	tags=28%, list=12%, signal=32%
GO_REGULATION_OF_SYSTEMIC_ARTERIAL_BLOOD_PRESSURE_BY_HORMONE	GO_REGULATION_OF_SYSTEMIC_ARTERIAL_BLOOD_PRESSURE_BY_HORMONE	33	0.5192192	1.6900826	0.007326	0.02707	0.997	2557	tags=48%, list=25%, signal=64%
GO_RESPONSE_TO_KETONE	GO_RESPONSE_TO_KETONE	148	0.38731885	1.6898296	0	0.02709	0.997	861	tags=21%, list=8%, signal=23%
GO_RUFFLE_ORGANIZATION	GO_RUFFLE_ORGANIZATION	16	0.60057306	1.6887826	0.012232	0.02729	0.997	2275	tags=63%, list=22%, signal=80%
GO_RESPONSE_TO_CORTICOSTEROID	GO_RESPONSE_TO_CORTICOSTEROID	149	0.3887187	1.687818	0	0.02742	0.997	1073	tags=21%, list=10%, signal=24%
GO_CELLULAR_RESPONSE_TO_LIPID	GO_CELLULAR_RESPONSE_TO_LIPID	358	0.3521531	1.6859789	0	0.02775	0.997	1313	tags=23%, list=13%, signal=25%
GO_REGULATION_OF_SMOOTH_MUSCLE_CONTRACTION	GO_REGULATION_OF_SMOOTH_MUSCLE_CONTRACTION	51	0.4589343	1.6859542	0.004367	0.02768	0.997	405	tags=16%, list=4%, signal=16%

GO_REGULATION_OF_T_HELPER_1_TYPE_IMMUNE_RESPONSE	GO_REGULATION_OF_T_HELPER_1_TYPE_IMMUNE_RESPONSE	15	0.61698574	1.6856626	0.018927	0.02769	0.997	2286	tags=73%, list=22%, signal=94%
GO_POSITIVE_REGULATION_OF_CYTOKINE_PRODUCTION_INVOLVED_IN_IMMUNE_RESPONSE	GO_POSITIVE_REGULATION_OF_CYTOKINE_PRODUCTION_INVOLVED_IN_IMMUNE_RESPONSE	23	0.5557679	1.68472	0.02069	0.0278	0.997	1930	tags=57%, list=19%, signal=69%
GO_REGULATION_OF_LIPID_STORAGE	GO_REGULATION_OF_LIPID_STORAGE	31	0.5154398	1.6792063	0.016779	0.02898	0.997	1313	tags=35%, list=13%, signal=40%
GO_NEGATIVE_REGULATION_OF_PRODUCTION_OF_MOLECULAR_MEDIATOR_OF_IMMUNE_RESPONSE	GO_NEGATIVE_REGULATION_OF_PRODUCTION_OF_MOLECULAR_MEDIATOR_OF_IMMUNE_RESPONSE	25	0.5367748	1.6777933	0.009709	0.02922	0.997	590	tags=24%, list=6%, signal=25%
GO_NEGATIVE_REGULATION_OF_CELLULAR_RESPONSE_TO_GROWTH_FACTOR_STIMULUS	GO_NEGATIVE_REGULATION_OF_CELLULAR_RESPONSE_TO_GROWTH_FACTOR_STIMULUS	92	0.4136812	1.6763481	0	0.0295	0.997	999	tags=24%, list=10%, signal=26%
GO_RESPONSE_TO_TUMOR_NECROSIS_FACTOR	GO_RESPONSE_TO_TUMOR_NECROSIS_FACTOR	175	0.3830613	1.6756045	0	0.02964	0.998	1741	tags=25%, list=17%, signal=30%
GO_T_HELPER_1_TYPE_IMMUNE_RESPONSE	GO_T_HELPER_1_TYPE_IMMUNE_RESPONSE	15	0.614007	1.6736765	0.028125	0.02993	0.999	1203	tags=53%, list=12%, signal=60%
GO_NEGATIVE_REGULATION_OF_B_CELL_ACTIVATION	GO_NEGATIVE_REGULATION_OF_B_CELL_ACTIVATION	25	0.5457958	1.6735976	0.00678	0.02987	0.999	1338	tags=40%, list=13%, signal=46%
GO_CELLULAR_RESPONSE_TO_INTERFERON_GAMMA	GO_CELLULAR_RESPONSE_TO_INTERFERON_GAMMA	88	0.4217117	1.6707654	0	0.03042	0.999	1853	tags=40%, list=18%, signal=48%
GO_NEGATIVE_REGULATION_OF_CELLULAR_RESPONSE_TO_TRANSFORMING_GROWTH_FACTOR_BETA_STIMULUS	GO_NEGATIVE_REGULATION_OF_CELLULAR_RESPONSE_TO_TRANSFORMING_GROWTH_FACTOR_BETA_STIMULUS	49	0.4608222	1.6696042	0	0.03065	0.999	1113	tags=27%, list=11%, signal=30%

GO_REGULATION_OF_SYNCYTIUM_FORMATION_BY_PLASMA_MEMBRANE_FUSION	GO_REGULATION_OF_SYNCYTIUM_FORMATION_BY_PLASMA_MEMBRANE_FUSION	21	0.56919795	1.6695964	0.0301	0.03059	0.999	2224	tags=57%, list=21%, signal=73%
GO_POSITIVE_REGULATION_OF_MYELOID_CELL_DIFFERENTIATION	GO_POSITIVE_REGULATION_OF_MYELOID_CELL_DIFFERENTIATION	62	0.44596514	1.6695845	0.004082	0.03052	0.999	2139	tags=40%, list=21%, signal=50%
GO_ERK1_AND_ERK2_CASCADE	GO_ERK1_AND_ERK2_CASCADE	18	0.5865059	1.666089	0.022663	0.03132	0.999	1123	tags=33%, list=11%, signal=37%
GO_REGULATION_OF_VIRAL_ENTRY_INTO_HOST_CELL	GO_REGULATION_OF_VIRAL_ENTRY_INTO_HOST_CELL	26	0.5243357	1.6652173	0.010169	0.03147	0.999	1302	tags=38%, list=13%, signal=44%
GO_TISSUE_MIGRATION	GO_TISSUE_MIGRATION	68	0.4366657	1.6651568	0.004274	0.03141	0.999	1193	tags=31%, list=11%, signal=35%
GO_POSITIVE_REGULATION_OF_NF_KAPPAB_TRANSCRIPTION_FACTOR_ACTIVITY	GO_POSITIVE_REGULATION_OF_NF_KAPPAB_TRANSCRIPTION_FACTOR_ACTIVITY	103	0.40015987	1.6644936	0	0.03151	0.999	1453	tags=26%, list=14%, signal=30%
GO_REGULATION_OF_MYELOID_LEUKOCYTE_DIFFERENTIATION	GO_REGULATION_OF_MYELOID_LEUKOCYTE_DIFFERENTIATION	80	0.42754668	1.6628599	0	0.03193	0.999	1055	tags=25%, list=10%, signal=28%
GO_REGULATION_OF_LYMPHOCYTE_MEDIATED_IMMUNITY	GO_REGULATION_OF_LYMPHOCYTE_MEDIATED_IMMUNITY	88	0.4200413	1.6620592	0.005556	0.03205	0.999	1446	tags=33%, list=14%, signal=38%
GO_RESPONSE_TO_CARBOHYDRATE	GO_RESPONSE_TO_CARBOHYDRATE	145	0.386918	1.6595461	0	0.03251	0.999	1193	tags=25%, list=11%, signal=28%
GO_POSITIVE_REGULATION_OF_TYROSINE_PHOSPHORYLATION_OF_STAT3_PROTEIN	GO_POSITIVE_REGULATION_OF_TYROSINE_PHOSPHORYLATION_OF_STAT3_PROTEIN	28	0.5243087	1.6575677	0.027027	0.03286	0.999	1236	tags=43%, list=12%, signal=49%
GO_RESPONSE_TO_HYDROGEN_PEROXIDE	GO_RESPONSE_TO_HYDROGEN_PEROXIDE	96	0.40246692	1.6570848	0.005128	0.0329	0.999	1224	tags=27%, list=12%, signal=30%

GO_NEGATIVE_REGULATION_OF_CYSTEINE_TYPE_ENDOPEPTIDASE_ACTIVITY	GO_NEGATIVE_REGULATION_OF_CYSTEINE_TYPE_ENDOPEPTIDASE_ACTIVITY	68	0.435326	1.6561046	0	0.0331	0.999	1290	tags=25%, list=12%, signal=28%
GO_RESPONSE_TO_ATP	GO_RESPONSE_TO_ATP	24	0.5443071	1.6555792	0.010067	0.03316	0.999	1609	tags=38%, list=15%, signal=44%
GO_REGULATION_OF_FIBROBLAST_GROWTH_FACTOR_RECEPTOR_SIGNALING_PATHWAY	GO_REGULATION_OF_FIBROBLAST_GROWTH_FACTOR_RECEPTOR_SIGNALING_PATHWAY	17	0.5799653	1.6551821	0.033435	0.03321	0.999	656	tags=24%, list=6%, signal=25%
GO_REGULATION_OF_LEUKOCYTE_MEDIATED_CYTOTOXICITY	GO_REGULATION_OF_LEUKOCYTE_MEDIATED_CYTOTOXICITY	37	0.4960295	1.6548774	0.003891	0.0332	0.999	1446	tags=38%, list=14%, signal=44%
GO_RECEPTOR_METABOLIC_PROCESS	GO_RECEPTOR_METABOLIC_PROCESS	65	0.4332334	1.6545732	0	0.0332	0.999	1394	tags=31%, list=13%, signal=35%
GO_LUNG_ALVEOLUS_DEVELOPMENT	GO_LUNG_ALVEOLUS_DEVELOPMENT	28	0.5167523	1.6539547	0.015528	0.03331	0.999	754	tags=29%, list=7%, signal=31%
GO_STEROL_TRANSPORT	GO_STEROL_TRANSPORT	41	0.48165497	1.6511483	0.003413	0.03395	0.999	948	tags=27%, list=9%, signal=29%
GO_NEGATIVE_REGULATION_OF_TRANSFORMING_GROWTH_FACTOR_BETA_RECEPTOR_SIGNALING_PATHWAY	GO_NEGATIVE_REGULATION_OF_TRANSFORMING_GROWTH_FACTOR_BETA_RECEPTOR_SIGNALING_PATHWAY	49	0.46082225	1.6508545	0.004032	0.03396	0.999	1113	tags=27%, list=11%, signal=30%
GO_BIOMINERAL_TISSUE_DEVELOPMENT	GO_BIOMINERAL_TISSUE_DEVELOPMENT	51	0.45121548	1.6490406	0.008475	0.03437	0.999	1578	tags=35%, list=15%, signal=41%
GO_ORGAN_OR_TISSUE_SPECIFIC_IMMUNE_RESPONSE	GO_ORGAN_OR_TISSUE_SPECIFIC_IMMUNE_RESPONSE	19	0.56126034	1.6490277	0.017341	0.0343	0.999	989	tags=21%, list=10%, signal=23%
GO_POSITIVE_REGULATION_OF_IMMUNOGLOBULIN_PRODUCTION	GO_POSITIVE_REGULATION_OF_IMMUNOGLOBULIN_PRODUCTION	26	0.5296152	1.6482874	0.013699	0.0344	0.999	1751	tags=46%, list=17%, signal=55%

GO_REGULATION_OF_SUPEROXIDE_METABOLIC_PROCESS	GO_REGULATION_OF_SUPEROXIDE_METABOLIC_PROCESS	20	0.5690696	1.6481793	0.00678	0.03436	0.999	743	tags=35%, list=7%, signal=38%
GO_DEFENSE_RESPONSE_TO_GRAM_POSITIVE_BACTERIUM	GO_DEFENSE_RESPONSE_TO_GRAM_POSITIVE_BACTERIUM	48	0.4649066	1.6472958	0.003968	0.03454	0.999	989	tags=27%, list=10%, signal=30%
GO_LIPID_LOCALIZATION	GO_LIPID_LOCALIZATION	186	0.36631566	1.6447301	0	0.03519	0.999	1227	tags=24%, list=12%, signal=26%
GO_RESPONSE_TO_TRANSFORMING_GROWTH_FACTOR_BETA	GO_RESPONSE_TO_TRANSFORMING_GROWTH_FACTOR_BETA	126	0.39369482	1.6443541	0	0.03522	0.999	809	tags=22%, list=8%, signal=24%
GO_FC_GAMMA_RECEPTOR_SIGNALING_PATHWAY	GO_FC_GAMMA_RECEPTOR_SIGNALING_PATHWAY	64	0.42877424	1.643677	0	0.03534	0.999	1082	tags=23%, list=10%, signal=26%
GO_CELLULAR_HOMEOSTASIS	GO_CELLULAR_HOMEOSTASIS	496	0.34064212	1.6409857	0	0.03599	0.999	1381	tags=23%, list=13%, signal=25%
GO_MYELOID_CELL_DIFFERENTIATION	GO_MYELOID_CELL_DIFFERENTIATION	153	0.3784012	1.6399692	0	0.03618	0.999	1290	tags=26%, list=12%, signal=29%
GO_NEGATIVE_REGULATION_OF_PROTEOLYSIS	GO_NEGATIVE_REGULATION_OF_PROTEOLYSIS	232	0.35551247	1.6398543	0	0.03613	0.999	1383	tags=22%, list=13%, signal=25%
GO_NEGATIVE_REGULATION_OF_ANION_TRANSPORT	GO_NEGATIVE_REGULATION_OF_ANION_TRANSPORT	29	0.5148664	1.6382102	0.016129	0.03659	0.999	889	tags=21%, list=9%, signal=23%
GO_REGULATION_OF_INTERFERON_BETA_PRODUCTION	GO_REGULATION_OF_INTERFERON_BETA_PRODUCTION	35	0.48021758	1.6371069	0.003597	0.03683	0.999	1678	tags=46%, list=16%, signal=54%
GO_NEGATIVE_REGULATION_OF_INFLAMMATORY_RESPONSE	GO_NEGATIVE_REGULATION_OF_INFLAMMATORY_RESPONSE	75	0.4179415	1.6364708	0	0.03694	0.999	1847	tags=37%, list=18%, signal=45%
GO_CARTILAGE_DEVELOPMENT_INVOLVED_IN_ENDOCHONDRAL_BONE_MORPHOGENESIS	GO_CARTILAGE_DEVELOPMENT_INVOLVED_IN_ENDOCHONDRAL_BONE_MORPHOGENESIS	17	0.58764607	1.6364233	0.022951	0.03687	0.999	775	tags=24%, list=7%, signal=25%

GO_REGULATION_OF_CELL_KILLING	GO_REGULATION_OF_CELL_KILLING	47	0.45710358	1.636374	0.004065	0.0368	0.999	1446	tags=34%, list=14%, signal=39%
GO_AGING	GO_AGING	217	0.36031434	1.631527	0	0.03815	1	1236	tags=24%, list=12%, signal=27%
GO_REGULATION_OF_TUMOR_NECROSIS_FACTOR_MEDIATED_SIGNALING_PATHWAY	GO_REGULATION_OF_TUMOR_NECROSIS_FACTOR_MEDIATED_SIGNALING_PATHWAY	39	0.49053788	1.6297485	0.007326	0.03862	1	936	tags=26%, list=9%, signal=28%
GO_REGULATION_OF_B_CELL_DIFFERENTIATION	GO_REGULATION_OF_B_CELL_DIFFERENTIATION	16	0.5916438	1.629726	0.015924	0.03855	1	585	tags=38%, list=6%, signal=40%
GO_RESPONSE_TO_FATTY_ACID	GO_RESPONSE_TO_FATTY_ACID	72	0.42827454	1.6280197	0.00905	0.03901	1	701	tags=24%, list=7%, signal=25%
GO_POSITIVE_REGULATION_OF_SMOOTH_MUSCLE_CONTRACTION	GO_POSITIVE_REGULATION_OF_SMOOTH_MUSCLE_CONTRACTION	25	0.51154315	1.6279426	0.019868	0.03893	1	405	tags=20%, list=4%, signal=21%
GO_REGULATION_OF_INTERLEUKIN_2_PRODUCTION	GO_REGULATION_OF_INTERLEUKIN_2_PRODUCTION	36	0.47652647	1.6277325	0.006969	0.03893	1	1930	tags=47%, list=19%, signal=58%
GO_POSITIVE_REGULATION_OF_SMOOTH_MUSCLE_CELL_PROLIFERATION	GO_POSITIVE_REGULATION_OF_SMOOTH_MUSCLE_CELL_PROLIFERATION	50	0.44185233	1.6262068	0.004762	0.03931	1	1169	tags=30%, list=11%, signal=34%
GO_GLAND_MORPHOGENESIS	GO_GLAND_MORPHOGENESIS	83	0.41290742	1.6254358	0	0.0395	1	1017	tags=27%, list=10%, signal=29%
GO_MORPHOGENESIS_OF_A_BRANCHING_STRUCTURE	GO_MORPHOGENESIS_OF_A_BRANCHING_STRUCTURE	135	0.37751582	1.6231617	0	0.04017	1	1603	tags=35%, list=15%, signal=41%
GO_HYDROGEN_PEROXIDE_CATABOLIC_PROCESS	GO_HYDROGEN_PEROXIDE_CATABOLIC_PROCESS	15	0.61044073	1.6230924	0.019355	0.0401	1	1247	tags=33%, list=12%, signal=38%
GO_REGULATION_OF_INTERFERON_ALPHA_PRODUCTION	GO_REGULATION_OF_INTERFERON_ALPHA_PRODUCTION	16	0.5959292	1.6202502	0.033537	0.04083	1	1678	tags=56%, list=16%, signal=67%

GO_NEGATIVE_REGULATION_OF_STEROID_METABOLIC_PROCESS	GO_NEGATIVE_REGULATION_OF_STEROID_METABOLIC_PROCESS	18	0.57337695	1.6189946	0.031153	0.0411	1	1487	tags=33%, list=14%, signal=39%
GO_PLACENTA_DEVELOPMENT	GO_PLACENTA_DEVELOPMENT	113	0.39054027	1.6187265	0.005348	0.0411	1	1338	tags=26%, list=13%, signal=29%
GO_AMINO_SUGAR_METABOLIC_PROCESS	GO_AMINO_SUGAR_METABOLIC_PROCESS	32	0.4977534	1.6183501	0.017606	0.04114	1	1467	tags=34%, list=14%, signal=40%
GO_NEGATIVE_REGULATION_OF_T_CELL_PROLIFERATION	GO_NEGATIVE_REGULATION_OF_T_CELL_PROLIFERATION	39	0.47607896	1.6181309	0.01087	0.04114	1	1216	tags=31%, list=12%, signal=35%
GO_VASCULAR_ENDOTHELIAL_GROWTH_FACTOR_RECEPTOR_SIGNALING_PATHWAY	GO_VASCULAR_ENDOTHELIAL_GROWTH_FACTOR_RECEPTOR_SIGNALING_PATHWAY	64	0.42380834	1.6179386	0.004032	0.04116	1	776	tags=22%, list=7%, signal=23%
GO_DIVALENT_INORGANIC_CATION_HOMEOSTASIS	GO_DIVALENT_INORGANIC_CATION_HOMEOSTASIS	268	0.34523356	1.6132743	0	0.04251	1	1381	tags=22%, list=13%, signal=25%
GO_REGULATION_OF_IMMUNOGLOBULIN_SECRETION	GO_REGULATION_OF_IMMUNOGLOBULIN_SECRETION	15	0.60030013	1.6123401	0.031746	0.04272	1	2240	tags=53%, list=22%, signal=68%
GO_REGULATION_OF_GASTRULATION	GO_REGULATION_OF_GASTRULATION	23	0.53521675	1.611721	0.016234	0.04279	1	583	tags=22%, list=6%, signal=23%
GO_CELLULAR_RESPONSE_TO_PROSTAGLANDIN_STIMULUS	GO_CELLULAR_RESPONSE_TO_PROSTAGLANDIN_STIMULUS	22	0.52579755	1.6067005	0.029508	0.04423	1	685	tags=27%, list=7%, signal=29%
GO_REGULATION_OF_TYPE_I_INTERFERON_PRODUCTION	GO_REGULATION_OF_TYPE_I_INTERFERON_PRODUCTION	86	0.39759278	1.604591	0	0.04488	1	1553	tags=35%, list=15%, signal=41%
GO_POSITIVE_REGULATION_OF_ALCOHOL_BIOSYNTHETIC_PROCESS	GO_POSITIVE_REGULATION_OF_ALCOHOL_BIOSYNTHETIC_PROCESS	21	0.53094906	1.6033844	0.012579	0.04514	1	1487	tags=38%, list=14%, signal=44%

GO_POSITIVE_REGULATION_OF_IMMUNOGLOBULIN_MEDIATED_IMMUNE_RESPONSE	GO_POSITIVE_REGULATION_OF_IMMUNOGLOBULIN_MEDIATED_IMMUNE_RESPONSE	23	0.52433366	1.6015294	0.022951	0.0457	1	1751	tags=48%, list=17%, signal=57%
GO_MAINTENANCE_OF_LOCATION	GO_MAINTENANCE_OF_LOCATION	101	0.39417338	1.6004452	0	0.04597	1	1250	tags=28%, list=12%, signal=31%
GO_TRANSFORMING_GROWTH_FACTOR_BETA_RECEPTOR_SIGNALING_PATHWAY	GO_TRANSFORMING_GROWTH_FACTOR_BETA_RECEPTOR_SIGNALING_PATHWAY	83	0.40224978	1.600288	0.004831	0.04591	1	986	tags=25%, list=9%, signal=28%
GO_NEGATIVE_REGULATION_OF_BIOMINERAL_TISSUE_DEVELOPMENT	GO_NEGATIVE_REGULATION_OF_BIOMINERAL_TISSUE_DEVELOPMENT	15	0.60113573	1.592929	0.023392	0.0484	1	823	tags=33%, list=8%, signal=36%
GO_NEGATIVE_REGULATION_OF_VASCULATURE_DEVELOPMENT	GO_NEGATIVE_REGULATION_OF_VASCULATURE_DEVELOPMENT	67	0.42723736	1.5927978	0.004831	0.04836	1	1646	tags=31%, list=16%, signal=37%
GO_CELLULAR_CHEMICAL_HOMEOSTASIS	GO_CELLULAR_CHEMICAL_HOMEOSTASIS	431	0.33347744	1.592359	0	0.04843	1	1381	tags=22%, list=13%, signal=25%
GO_POSITIVE_REGULATION_OF_B_CELL_MEDIATED_IMMUNITY	GO_POSITIVE_REGULATION_OF_B_CELL_MEDIATED_IMMUNITY	23	0.52433354	1.5910277	0.022436	0.04881	1	1751	tags=48%, list=17%, signal=57%
GO_TUMOR_NECROSIS_FACTOR_MEDIATED_SIGNALING_PATHWAY	GO_TUMOR_NECROSIS_FACTOR_MEDIATED_SIGNALING_PATHWAY	95	0.40166202	1.5892123	0.009524	0.04936	1	1954	tags=26%, list=19%, signal=32%
GO_NEGATIVE_REGULATION_OF_BMP_SIGNALING_PATHWAY	GO_NEGATIVE_REGULATION_OF_BMP_SIGNALING_PATHWAY	29	0.48940304	1.5889904	0.013559	0.04934	1	1394	tags=38%, list=13%, signal=44%
GO_TRANSITION_METAL_ION_HOMEOSTASIS	GO_TRANSITION_METAL_ION_HOMEOSTASIS	73	0.41867065	1.588344	0.004587	0.04943	1	1059	tags=21%, list=10%, signal=23%
GO_POSITIVE_REGULATION_OF_MAPK_CASCADE	GO_POSITIVE_REGULATION_OF_MAPK_CASCADE	361	0.32954842	1.587431	0	0.04973	1	1281	tags=22%, list=12%, signal=25%
GO_REGULATION_OF_BONE_RESORPTION	GO_REGULATION_OF_BONE_RESORPTION	25	0.507684	1.5860901	0.029032	0.05012	1	1578	tags=36%, list=15%, signal=42%

GO_CARBOHYDRATE_DERIVATIVE_CATABOLIC_PROCESS	GO_CARBOHYDRATE_DERIVATIVE_CATABOLIC_PROCESS	129	0.3708597	1.5850432	0	0.05035	1	1137	tags=27%, list=11%, signal=30%
GO_OSSIFICATION	GO_OSSIFICATION	199	0.3520282	1.5839599	0	0.05061	1	1247	tags=25%, list=12%, signal=27%
GO_BONE_GROWTH	GO_BONE_GROWTH	17	0.5730573	1.5828578	0.028213	0.05094	1	1348	tags=35%, list=13%, signal=40%
GO_POSITIVE_REGULATION_OF_BLOOD_VESSEL_ENDOTHELIAL_CELL_MIGRATION	GO_POSITIVE_REGULATION_OF_BLOOD_VESSEL_ENDOTHELIAL_CELL_MIGRATION	22	0.5273797	1.5821688	0.023729	0.05109	1	581	tags=23%, list=6%, signal=24%
GO_CELLULAR_RESPONSE_TO_PROSTAGLANDIN_E_STIMULUS	GO_CELLULAR_RESPONSE_TO_PROSTAGLANDIN_E_STIMULUS	17	0.5502616	1.5815631	0.031949	0.05121	1	1196	tags=35%, list=12%, signal=40%
GO_ACYLGLYCEROL_HOMEOSTASIS	GO_ACYLGLYCEROL_HOMEOSTASIS	20	0.5413969	1.5811257	0.029221	0.05127	1	1355	tags=25%, list=13%, signal=29%
GO_HEAD_MORPHOGENESIS	GO_HEAD_MORPHOGENESIS	28	0.49709144	1.5800109	0.034091	0.05157	1	955	tags=32%, list=9%, signal=35%
GO_MONOAMINE_TRANSPORT	GO_MONOAMINE_TRANSPORT	19	0.56671405	1.5797638	0.031847	0.05157	1	175	tags=11%, list=2%, signal=11%
GO_RESPONSE_TO_TEMPERATURE_STIMULUS	GO_RESPONSE_TO_TEMPERATURE_STIMULUS	116	0.38014084	1.5780457	0.006579	0.05206	1	1149	tags=25%, list=11%, signal=28%
GO_REGULATION_OF_TYROSINE_PHOSPHORYLATION_OF_STAT3_PROTEIN	GO_REGULATION_OF_TYROSINE_PHOSPHORYLATION_OF_STAT3_PROTEIN	34	0.4688268	1.577917	0.02214	0.05203	1	1236	tags=38%, list=12%, signal=43%
GO_REGULATION_OF_ACTIVATED_T_CELL_PROLIFERATION	GO_REGULATION_OF_ACTIVATED_T_CELL_PROLIFERATION	29	0.4954019	1.5777016	0.017857	0.05202	1	1116	tags=28%, list=11%, signal=31%
GO_BONE_REMODELING	GO_BONE_REMODELING	30	0.48422718	1.577031	0.017483	0.05214	1	876	tags=27%, list=8%, signal=29%
GO_CELLULAR_RESPONSE_TO_OXYGEN_LEVELS	GO_CELLULAR_RESPONSE_TO_OXYGEN_LEVELS	116	0.3773564	1.5742608	0	0.05294	1	1035	tags=22%, list=10%, signal=24%

GO_KERATAN_SULFATE_METABOLIC_PROCESS	GO_KERATAN_SULFATE_METABOLIC_PROCESS	27	0.5024103	1.5720558	0.027397	0.05371	1	647	tags=26%, list=6%, signal=28%
GO_POSITIVE_REGULATION_OF_ACTIVATED_T_CELL_PROLIFERATION	GO_POSITIVE_REGULATION_OF_ACTIVATED_T_CELL_PROLIFERATION	21	0.54365087	1.5716207	0.024691	0.05375	1	2831	tags=67%, list=27%, signal=91%
GO_POSITIVE_REGULATION_OF_MYOTUBE_DIFFERENTIATION	GO_POSITIVE_REGULATION_OF_MYOTUBE_DIFFERENTIATION	24	0.5109406	1.5694968	0.029801	0.05448	1	333	tags=21%, list=3%, signal=21%
GO_INTERFERON_GAMMA_MEDIATED_SIGNALING_PATHWAY	GO_INTERFERON_GAMMA_MEDIATED_SIGNALING_PATHWAY	55	0.42924115	1.5694788	0.004367	0.05438	1	1853	tags=45%, list=18%, signal=55%
GO_POSITIVE_REGULATION_OF_TYPE_I_INTERFERON_PRODUCTION	GO_POSITIVE_REGULATION_OF_TYPE_I_INTERFERON_PRODUCTION	57	0.41965675	1.5693748	0.008658	0.05433	1	1553	tags=39%, list=15%, signal=45%
GO_POSITIVE_REGULATION_OF_BLOOD_PRESSURE	GO_POSITIVE_REGULATION_OF_BLOOD_PRESSURE	34	0.47685498	1.5684305	0.03125	0.05463	1	1241	tags=21%, list=12%, signal=23%
GO_REGULATION_OF_APOPTOTIC_SIGNALING_PATHWAY	GO_REGULATION_OF_APOPTOTIC_SIGNALING_PATHWAY	284	0.3367652	1.5663025	0	0.0554	1	1073	tags=18%, list=10%, signal=19%
GO_ENDOTHELIAL_CELL_DEVELOPMENT	GO_ENDOTHELIAL_CELL_DEVELOPMENT	38	0.46930218	1.5659314	0.015748	0.05542	1	734	tags=26%, list=7%, signal=28%
GO_NATURAL_KILLER_CELL_ACTIVATION	GO_NATURAL_KILLER_CELL_ACTIVATION	37	0.4553697	1.5658475	0.011152	0.05534	1	1440	tags=35%, list=14%, signal=41%
GO_REGULATION_OF_TRANSMEMBRANE_RECEPTOR_PROTEIN_SERINE_THREONINE_KINASE_SIGNALING_PATHWAY	GO_REGULATION_OF_TRANSMEMBRANE_RECEPTOR_PROTEIN_SERINE_THREONINE_KINASE_SIGNALING_PATHWAY	155	0.3557425	1.5646178	0	0.05574	1	1233	tags=23%, list=12%, signal=26%
GO_REGULATION_OF_ACTIN_CYTOSKELETON_REORGANIZATION	GO_REGULATION_OF_ACTIN_CYTOSKELETON_REORGANIZATION	26	0.5020336	1.5626789	0.021739	0.05635	1	978	tags=38%, list=9%, signal=42%

GO_RESPONSE_TO_REACTIVE_OXYGEN_SPECIES	GO_RESPONSE_TO_REACTIVE_OXYGEN_SPECIES	167	0.35887352	1.5605927	0	0.05707	1	1377	tags=26%, list=13%, signal=29%
GO_NEGATIVE_REGULATION_OF_AXON_GUIDANCE	GO_NEGATIVE_REGULATION_OF_AXON_GUIDANCE	23	0.50829595	1.5599053	0.030864	0.05725	1	1496	tags=30%, list=14%, signal=35%
GO_POSITIVE_REGULATION_OF_CELL_SUBSTRATE_ADHESION	GO_POSITIVE_REGULATION_OF_CELL_SUBSTRATE_ADHESION	71	0.39512742	1.559406	0	0.05734	1	1649	tags=30%, list=16%, signal=35%
GO_IMMUNE_RESPONSE_REGULATING_CELL_SURFACE_RECEPTOR_SIGNALING_PATHWAY	GO_IMMUNE_RESPONSE_REGULATING_CELL_SURFACE_RECEPTOR_SIGNALING_PATHWAY	239	0.3378022	1.5587791	0	0.05752	1	1364	tags=22%, list=13%, signal=25%
GO_PROTEIN_KINASE_B_SIGNALING	GO_PROTEIN_KINASE_B_SIGNALING	28	0.47664225	1.5585654	0.024306	0.05747	1	1377	tags=43%, list=13%, signal=49%
GO_REGULATION_OF_CELLULAR_RESPONSE_TO_GROWTH_FACTOR_STIMULUS	GO_REGULATION_OF_CELLULAR_RESPONSE_TO_GROWTH_FACTOR_STIMULUS	166	0.35102963	1.5574576	0	0.05785	1	1113	tags=21%, list=11%, signal=23%
GO_POSITIVE_REGULATION_OF_INTERLEUKIN_2_PRODUCTION	GO_POSITIVE_REGULATION_OF_INTERLEUKIN_2_PRODUCTION	26	0.50050604	1.5573541	0.032787	0.05779	1	1470	tags=46%, list=14%, signal=54%
GO_REGULATION_OF_LIPID_BIOSYNTHETIC_PROCESS	GO_REGULATION_OF_LIPID_BIOSYNTHETIC_PROCESS	95	0.3825233	1.5567535	0	0.05797	1	1355	tags=26%, list=13%, signal=30%
GO_NEGATIVE_REGULATION_OF_TRANSMEMBRANE_RECEPTOR_PROTEIN_SERINE_THREONINE_KINASE_SIGNALING_PATHWAY	GO_NEGATIVE_REGULATION_OF_TRANSMEMBRANE_RECEPTOR_PROTEIN_SERINE_THREONINE_KINASE_SIGNALING_PATHWAY	73	0.4038186	1.5566936	0	0.0579	1	1233	tags=26%, list=12%, signal=29%
GO_MATERNAL_PLACENTA_DEVELOPMENT	GO_MATERNAL_PLACENTA_DEVELOPMENT	26	0.52518046	1.5566598	0.033133	0.05781	1	1736	tags=42%, list=17%, signal=51%

GO_POSITIVE_REGULATION_OF_PROTEIN_IMPORT	GO_POSITIVE_REGULATION_OF_PROTEIN_IMPORT	78	0.39489982	1.5563744	0	0.05784	1	664	tags=22%, list=6%, signal=23%
GO_TRIGLYCERIDE_HOMEOSTASIS	GO_TRIGLYCERIDE_HOMEOSTASIS	20	0.541397	1.555418	0.038585	0.05807	1	1355	tags=25%, list=13%, signal=29%
GO_PROSTAGLANDIN_BIOSYNTHETIC_PROCESS	GO_PROSTAGLANDIN_BIOSYNTHETIC_PROCESS	15	0.5637362	1.5540144	0.040881	0.0585	1	253	tags=27%, list=2%, signal=27%
GO_ENDOTHELIAL_CELL_DIFFERENTIATION	GO_ENDOTHELIAL_CELL_DIFFERENTIATION	57	0.4096686	1.5539309	0	0.05842	1	734	tags=23%, list=7%, signal=24%
GO_NEGATIVE_REGULATION_OF_LEUKOCYTE_DIFFERENTIATION	GO_NEGATIVE_REGULATION_OF_LEUKOCYTE_DIFFERENTIATION	61	0.4163641	1.551555	0.004292	0.05925	1	1085	tags=26%, list=10%, signal=29%
GO_RESPONSE_TO_COPPER_ION	GO_RESPONSE_TO_COPPER_ION	26	0.5062762	1.5507191	0.02439	0.05944	1	616	tags=27%, list=6%, signal=29%
GO_POSITIVE_REGULATION_OF_BLOOD_CIRCULATION	GO_POSITIVE_REGULATION_OF_BLOOD_CIRCULATION	86	0.3791429	1.5502092	0.010753	0.05959	1	779	tags=19%, list=8%, signal=20%
GO_NEGATIVE_REGULATION_OF_APOPTOTIC_SIGNALING_PATHWAY	GO_NEGATIVE_REGULATION_OF_APOPTOTIC_SIGNALING_PATHWAY	156	0.35083959	1.5499963	0	0.05959	1	1073	tags=19%, list=10%, signal=21%
GO_REGULATION_OF_SMOOTH_MUSCLE_CELL_PROLIFERATION	GO_REGULATION_OF_SMOOTH_MUSCLE_CELL_PROLIFERATION	87	0.38444677	1.5496454	0.009302	0.05959	1	1236	tags=28%, list=12%, signal=31%
GO_POSITIVE_REGULATION_OF_PEPTIDYL_TYROSINE_PHOSPHORYLATION	GO_POSITIVE_REGULATION_OF_PEPTIDYL_TYROSINE_PHOSPHORYLATION	132	0.36230212	1.5491134	0	0.05969	1	1246	tags=27%, list=12%, signal=30%
GO_SMOOTH_MUSCLE_TISSUE_DEVELOPMENT	GO_SMOOTH_MUSCLE_TISSUE_DEVELOPMENT	15	0.58373153	1.5484499	0.029412	0.05987	1	1328	tags=47%, list=13%, signal=53%
GO_AMINOGLYCAN_METABOLIC_PROCESS	GO_AMINOGLYCAN_METABOLIC_PROCESS	118	0.37461975	1.5472095	0	0.06027	1	935	tags=25%, list=9%, signal=27%
GO_REGULATION_OF_INTERLEUKIN_4_PRODUCTION	GO_REGULATION_OF_INTERLEUKIN_4_PRODUCTION	18	0.5504841	1.5469128	0.028662	0.06034	1	1801	tags=50%, list=17%, signal=60%

GO_POSITIVE_REGULATION_OF_OSTEOCLAST_DIFFERENTIATION	GO_POSITIVE_REGULATION_OF_OSTEOCLAST_DIFFERENTIATION	17	0.55352974	1.5468137	0.04127	0.06026	1	2139	tags=53%, list=21%, signal=67%
GO_POSITIVE_REGULATION_OF_APOPTOTIC_SIGNALING_PATHWAY	GO_POSITIVE_REGULATION_OF_APOPTOTIC_SIGNALING_PATHWAY	141	0.36169198	1.5466559	0	0.06023	1	1142	tags=20%, list=11%, signal=22%
GO_ANTIGEN_PROCESSING_AND_PRESENTATION_OF_EXOGENOUS_PEPTIDE_ANTIGEN_VIA_MHC_CLASS_I	GO_ANTIGEN_PROCESSING_AND_PRESENTATION_OF_EXOGENOUS_PEPTIDE_ANTIGEN_VIA_MHC_CLASS_I	56	0.42049152	1.5460297	0.004444	0.06038	1	914	tags=18%, list=9%, signal=19%
GO_POLYOL_BIOSYNTHETIC_PROCESS	GO_POLYOL_BIOSYNTHETIC_PROCESS	18	0.53373235	1.5450997	0.03871	0.06066	1	1133	tags=28%, list=11%, signal=31%
GO_REGULATION_OF_MYELOID_CELL_DIFFERENTIATION	GO_REGULATION_OF_MYELOID_CELL_DIFFERENTIATION	130	0.3683716	1.5447614	0	0.06069	1	969	tags=21%, list=9%, signal=23%
GO_INTESTINAL_ABSORPTION	GO_INTESTINAL_ABSORPTION	20	0.5369834	1.5446056	0.030488	0.06067	1	2524	tags=45%, list=24%, signal=59%
GO_NEGATIVE_REGULATION_OF_ENDOCYTOSIS	GO_NEGATIVE_REGULATION_OF_ENDOCYTOSIS	30	0.47708485	1.5444521	0.027304	0.06065	1	1311	tags=33%, list=13%, signal=38%
GO_REGULATION_OF_CARTILAGE_DEVELOPMENT	GO_REGULATION_OF_CARTILAGE_DEVELOPMENT	49	0.43549037	1.543314	0.016064	0.06106	1	1222	tags=22%, list=12%, signal=25%
GO_RESPONSE_TO_IMMOBILIZATION_STRESS	GO_RESPONSE_TO_IMMOBILIZATION_STRESS	18	0.5320965	1.542625	0.03869	0.06124	1	1512	tags=33%, list=15%, signal=39%
GO_RESPONSE_TO_EXOGENOUS_DSRNA	GO_RESPONSE_TO_EXOGENOUS_DSRNA	28	0.498545	1.5400269	0.024465	0.0622	1	1426	tags=29%, list=14%, signal=33%
GO_NEGATIVE_REGULATION_OF_ADAPTIVE_IMMUNE_RESPONSE	GO_NEGATIVE_REGULATION_OF_ADAPTIVE_IMMUNE_RESPONSE	31	0.48096037	1.5398952	0.017794	0.06215	1	617	tags=23%, list=6%, signal=24%
GO_REGULATION_OF_LIPID_CATABOLIC_PROCESS	GO_REGULATION_OF_LIPID_CATABOLIC_PROCESS	41	0.44820294	1.5389411	0.018868	0.06251	1	1326	tags=27%, list=13%, signal=31%

GO_NEGATIVE_REGULATION_OF_PLATELET_ACTIVATION	GO_NEGATIVE_REGULATION_OF_PLATELET_ACTIVATION	15	0.57206947	1.536665	0.042135	0.06342	1	494	tags=27%, list=5%, signal=28%
GO_RESPONSE_TO_ACID_CHEMICAL	GO_RESPONSE_TO_ACID_CHEMICAL	258	0.34201038	1.5356221	0	0.06378	1	1161	tags=21%, list=11%, signal=23%
GO_NEGATIVE_REGULATION_OF_CELL_SUBSTRATE_ADHESION	GO_NEGATIVE_REGULATION_OF_CELL_SUBSTRATE_ADHESION	45	0.44558844	1.5350564	0.011111	0.06387	1	674	tags=18%, list=6%, signal=19%
GO_REGULATION_OF_T_CELL_MEDIATED_CYTOTOXICITY	GO_REGULATION_OF_T_CELL_MEDIATED_CYTOTOXICITY	18	0.55234516	1.5348405	0.045161	0.06386	1	914	tags=28%, list=9%, signal=30%
GO_ARTERY_DEVELOPMENT	GO_ARTERY_DEVELOPMENT	63	0.40288058	1.5345688	0.017241	0.06386	1	754	tags=19%, list=7%, signal=20%
GO_POSITIVE_REGULATION_OF_ENDOTHELIAL_CELL_MIGRATION	GO_POSITIVE_REGULATION_OF_ENDOTHELIAL_CELL_MIGRATION	56	0.4213768	1.5333794	0.026316	0.06433	1	581	tags=14%, list=6%, signal=15%
GO_POSITIVE_REGULATION_OF_TRANSFORMING_GROWTH_FACTOR_BETA_RECEPTOR_SIGNALING_PATHWAY	GO_POSITIVE_REGULATION_OF_TRANSFORMING_GROWTH_FACTOR_BETA_RECEPTOR_SIGNALING_PATHWAY	20	0.5223634	1.5322592	0.031153	0.06481	1	1187	tags=35%, list=11%, signal=39%
GO_MYOBLAST_FUSION	GO_MYOBLAST_FUSION	16	0.5582985	1.5315474	0.050898	0.06498	1	620	tags=25%, list=6%, signal=27%
GO_REGULATION_OF_FATTY_ACID_TRANSPORT	GO_REGULATION_OF_FATTY_ACID_TRANSPORT	24	0.50966936	1.5274228	0.045752	0.06682	1	889	tags=21%, list=9%, signal=23%
GO_BONE_MINERALIZATION	GO_BONE_MINERALIZATION	28	0.47524384	1.5251634	0.017007	0.06775	1	1518	tags=43%, list=15%, signal=50%
GO_MYD88_INDEPENDENT_TOLL_LIKE_RECEPTOR_SIGNALING_PATHWAY	GO_MYD88_INDEPENDENT_TOLL_LIKE_RECEPTOR_SIGNALING_PATHWAY	29	0.47058886	1.5250443	0.01773	0.06769	1	1471	tags=24%, list=14%, signal=28%
GO_PROSTANOID_BIOSYNTHETIC_PROCESS	GO_PROSTANOID_BIOSYNTHETIC_PROCESS	15	0.56373614	1.5232621	0.037618	0.06849	1	253	tags=27%, list=2%, signal=27%

GO_RESPONSE_TO_EXTRACELLULAR_STIMULUS	GO_RESPONSE_TO_EXTRACELLULAR_STIMULUS	364	0.31488043	1.5216839	0	0.06908	1	1241	tags=21%, list=12%, signal=23%
GO_REGULATION_OF_INTERLEUKIN_1_BETA_PRODUCTION	GO_REGULATION_OF_INTERLEUKIN_1_BETA_PRODUCTION	31	0.475983	1.5213863	0.021277	0.06914	1	1609	tags=42%, list=15%, signal=49%
GO_REGULATION_OF_T_CELL_CYTOKINE_PRODUCTION	GO_REGULATION_OF_T_CELL_CYTOKINE_PRODUCTION	17	0.5398706	1.5204228	0.049689	0.06941	1	1992	tags=53%, list=19%, signal=65%
GO_NEGATIVE_REGULATION_OF_DEFENSE_RESPONSE	GO_NEGATIVE_REGULATION_OF_DEFENSE_RESPONSE	109	0.37446824	1.5203142	0	0.06934	1	1847	tags=39%, list=18%, signal=46%
GO_REGULATION_OF_ENDOTHELIAL_CELL_PROLIFERATION	GO_REGULATION_OF_ENDOTHELIAL_CELL_PROLIFERATION	79	0.39551473	1.520238	0.010638	0.06924	1	940	tags=20%, list=9%, signal=22%
GO_POSITIVE_REGULATION_OF_EPITHELIAL_CELL_PROLIFERATION	GO_POSITIVE_REGULATION_OF_EPITHELIAL_CELL_PROLIFERATION	119	0.3657814	1.5201323	0.007576	0.06916	1	975	tags=21%, list=9%, signal=23%
GO_PROTEIN_SECRETION	GO_PROTEIN_SECRETION	83	0.3932644	1.5177693	0.015385	0.07012	1	508	tags=16%, list=5%, signal=16%
GO_VIRAL_LIFE_CYCLE	GO_VIRAL_LIFE_CYCLE	202	0.32908222	1.5161139	0	0.07075	1	1042	tags=16%, list=10%, signal=17%
GO_NEGATIVE_REGULATION_OF_LIPID_TRANSPORT	GO_NEGATIVE_REGULATION_OF_LIPID_TRANSPORT	21	0.51367724	1.515853	0.034722	0.07077	1	2331	tags=43%, list=22%, signal=55%
GO_REGULATION_OF_CYSTEINE_TYPE_ENDOPEPTIDASE_ACTIVITY	GO_REGULATION_OF_CYSTEINE_TYPE_ENDOPEPTIDASE_ACTIVITY	167	0.3472216	1.5157117	0	0.0707	1	1142	tags=19%, list=11%, signal=21%
GO_PATTERNING_OF_BLOOD_VESSELS	GO_PATTERNING_OF_BLOOD_VESSELS	26	0.48908475	1.5152365	0.038869	0.07081	1	828	tags=27%, list=8%, signal=29%

GO_TRANSMEMBRANE_RECEPTOR_PROTEIN_SERINE_THREONINE_KINASE_SIGNALING_PATHWAY	GO_TRANSMEMBRANE_RECEPTOR_PROTEIN_SERINE_THREONINE_KINASE_SIGNALING_PATHWAY	156	0.34533706	1.5125943	0	0.07191	1	1577	tags=28%, list=15%, signal=33%
GO_POSITIVE_REGULATION_OF_ENDOTHELIAL_CELL_PROLIFERATION	GO_POSITIVE_REGULATION_OF_ENDOTHELIAL_CELL_PROLIFERATION	56	0.40547392	1.5112278	0.008403	0.07244	1	940	tags=23%, list=9%, signal=25%
GO_MYELOID_CELL_HOMEOSTASIS	GO_MYELOID_CELL_HOMEOSTASIS	76	0.38527256	1.5097764	0.010204	0.07307	1	1112	tags=22%, list=11%, signal=25%
GO_DETOXIFICATION	GO_DETOXIFICATION	48	0.433589	1.5096881	0.004016	0.07299	1	1886	tags=35%, list=18%, signal=43%
GO_REGULATION_OF_CHOLESTEROL_TRANSPORT	GO_REGULATION_OF_CHOLESTEROL_TRANSPORT	31	0.4688449	1.5085771	0.034483	0.07347	1	1313	tags=32%, list=13%, signal=37%
GO_REGULATION_OF_IMMUNOGLOBULIN_PRODUCTION	GO_REGULATION_OF_IMMUNOGLOBULIN_PRODUCTION	40	0.43923974	1.5071757	0.007143	0.07396	1	1751	tags=38%, list=17%, signal=45%
GO_REGULATION_OF_CELL_SHAPE	GO_REGULATION_OF_CELL_SHAPE	94	0.3708901	1.5046686	0.00578	0.07505	1	1281	tags=32%, list=12%, signal=36%
GO_REGULATION_OF_CYSTEINE_TYPE_ENDOPEPTIDASE_ACTIVITY_INVOLVED_IN_APOPTOTIC_SIGNALING_PATHWAY	GO_REGULATION_OF_CYSTEINE_TYPE_ENDOPEPTIDASE_ACTIVITY_INVOLVED_IN_APOPTOTIC_SIGNALING_PATHWAY	20	0.51669383	1.504653	0.037931	0.07493	1	2154	tags=60%, list=21%, signal=76%
GO_SALIVARY_GLAND_DEVELOPMENT	GO_SALIVARY_GLAND_DEVELOPMENT	31	0.45926473	1.5034764	0.013559	0.07535	1	1017	tags=29%, list=10%, signal=32%
GO_REGULATION_OF_LIPASE_ACTIVITY	GO_REGULATION_OF_LIPASE_ACTIVITY	68	0.39568067	1.5014251	0.023474	0.07627	1	978	tags=22%, list=9%, signal=24%
GO_POSITIVE_REGULATION_OF_CELLULAR_RESPONSE_TO_TRANSFORMING_GROWTH_FACTOR_BETA_STIMULUS	GO_POSITIVE_REGULATION_OF_CELLULAR_RESPONSE_TO_TRANSFORMING_GROWTH_FACTOR_BETA_STIMULUS	20	0.5223633	1.5010095	0.031949	0.07636	1	1187	tags=35%, list=11%, signal=39%

GO_POSITIVE_REGULATION_OF_CALCIUM_MEDIATED_SIGNALING	GO_POSITIVE_REGULATION_OF_CALCIUM_MEDIATED_SIGNALING	28	0.48079893	1.5008483	0.051447	0.07631	1	1369	tags=32%, list=13%, signal=37%
GO_PROTEIN_HETEROOLIGOMERIZATION	GO_PROTEIN_HETEROOLIGOMERIZATION	81	0.37165624	1.500227	0.026316	0.07649	1	1538	tags=22%, list=15%, signal=26%
GO_NEGATIVE_REGULATION_OF_CELL_MATRIX_ADHESION	GO_NEGATIVE_REGULATION_OF_CELL_MATRIX_ADHESION	25	0.48224062	1.4993793	0.047771	0.07687	1	279	tags=16%, list=3%, signal=16%
GO_NEGATIVE_REGULATION_OF_ENDOTHELIAL_CELL_MIGRATION	GO_NEGATIVE_REGULATION_OF_ENDOTHELIAL_CELL_MIGRATION	34	0.4476066	1.4990529	0.027972	0.07693	1	1857	tags=35%, list=18%, signal=43%
GO_ENDOCRINE_PROCESS	GO_ENDOCRINE_PROCESS	41	0.4385475	1.4987307	0.02439	0.07697	1	2324	tags=39%, list=22%, signal=50%
GO_POSITIVE_REGULATION_OF_RECEPTOR_MEDIATED_ENDOCYTOSIS	GO_POSITIVE_REGULATION_OF_RECEPTOR_MEDIATED_ENDOCYTOSIS	37	0.43677086	1.4980841	0.032967	0.07717	1	1330	tags=24%, list=13%, signal=28%
GO_REGULATION_OF_MUSCLE_CONTRACTION	GO_REGULATION_OF_MUSCLE_CONTRACTION	126	0.34645432	1.4974777	0.006579	0.07737	1	1269	tags=20%, list=12%, signal=22%
GO_POSITIVE_REGULATION_OF_DNA_TEMPLATED_TRANSCRIPTION_INITIATION	GO_POSITIVE_REGULATION_OF_DNA_TEMPLATED_TRANSCRIPTION_INITIATION	21	0.51031554	1.4960303	0.048232	0.07799	1	534	tags=24%, list=5%, signal=25%
GO_REGULATION_OF_PLASMA_MEMBRANE_ORGANIZATION	GO_REGULATION_OF_PLASMA_MEMBRANE_ORGANIZATION	53	0.40796146	1.4954971	0.008065	0.07816	1	1834	tags=38%, list=18%, signal=46%
GO_CELLULAR_GLUCOSE_HOMEOSTASIS	GO_CELLULAR_GLUCOSE_HOMEOSTASIS	63	0.39760387	1.495275	0.017857	0.07812	1	889	tags=25%, list=9%, signal=28%
GO_REGULATION_OF_FIBROBLAST_MIGRATION	GO_REGULATION_OF_FIBROBLAST_MIGRATION	21	0.5105352	1.4950325	0.048276	0.07809	1	1736	tags=38%, list=17%, signal=46%
GO_SKIN_DEVELOPMENT	GO_SKIN_DEVELOPMENT	137	0.3461912	1.4946607	0	0.07815	1	1180	tags=19%, list=11%, signal=21%
GO_REGULATION_OF_STEROL_TRANSPORT	GO_REGULATION_OF_STEROL_TRANSPORT	31	0.46884483	1.4930676	0.035088	0.07888	1	1313	tags=32%, list=13%, signal=37%

GO_EPITHELIAL_CELL_DEVELOPMENT	GO_EPITHELIAL_CELL_DEVELOPMENT	148	0.34542	1.4900137	0.00813	0.08046	1	1182	tags=22%, list=11%, signal=24%
GO_POSITIVE_REGULATION_OF_MUSCLE_CONTRACTION	GO_POSITIVE_REGULATION_OF_MUSCLE_CONTRACTION	38	0.4299372	1.4898108	0.024911	0.08043	1	779	tags=24%, list=8%, signal=26%
GO_BLOOD_COAGULATION_FIBRIN_CLOT_FORMATION	GO_BLOOD_COAGULATION_FIBRIN_CLOT_FORMATION	23	0.48971805	1.4890544	0.046205	0.08072	1	1238	tags=22%, list=12%, signal=25%
GO_NEGATIVE_REGULATION_OF_LEUKOCYTE_MIGRATION	GO_NEGATIVE_REGULATION_OF_LEUKOCYTE_MIGRATION	23	0.48591274	1.48821	0.054422	0.08105	1	763	tags=30%, list=7%, signal=33%
GO_REGULATION_OF_ACTIN_FILAMENT_BASED_PROCESS	GO_REGULATION_OF_ACTIN_FILAMENT_BASED_PROCESS	230	0.32478604	1.4878397	0	0.08109	1	1667	tags=30%, list=16%, signal=35%
GO_POSITIVE_REGULATION_OF_REPRODUCTIVE_PROCESS	GO_POSITIVE_REGULATION_OF_REPRODUCTIVE_PROCESS	45	0.42299724	1.4874927	0.015686	0.08115	1	2022	tags=40%, list=19%, signal=49%
GO_POSITIVE_REGULATION_OF_T_CELL_MEDIATED_IMMUNITY	GO_POSITIVE_REGULATION_OF_T_CELL_MEDIATED_IMMUNITY	24	0.48310357	1.4860644	0.017241	0.08172	1	2283	tags=50%, list=22%, signal=64%
GO_PHAGOSOME_ACIDIFICATION	GO_PHAGOSOME_ACIDIFICATION	18	0.5233664	1.485405	0.054441	0.08197	1	310	tags=17%, list=3%, signal=17%
GO_NEGATIVE_REGULATION_OF_LYMPHOCYTE_MEDIATED_IMMUNITY	GO_NEGATIVE_REGULATION_OF_LYMPHOCYTE_MEDIATED_IMMUNITY	28	0.46231282	1.4845926	0.030612	0.08235	1	1446	tags=39%, list=14%, signal=46%
GO_REGULATION_OF_ENDOTHELIAL_CELL_MIGRATION	GO_REGULATION_OF_ENDOTHELIAL_CELL_MIGRATION	96	0.3642446	1.4844029	0.010582	0.0823	1	1885	tags=31%, list=18%, signal=38%
GO_MESENCHYME_MORPHOGENESIS	GO_MESENCHYME_MORPHOGENESIS	32	0.459336	1.4829416	0.039007	0.08297	1	754	tags=28%, list=7%, signal=30%
GO_BONE_MORPHOGENESIS	GO_BONE_MORPHOGENESIS	63	0.3936938	1.4811963	0.017857	0.08381	1	857	tags=19%, list=8%, signal=21%
GO_NEGATIVE_REGULATION_OF_ANOIKIS	GO_NEGATIVE_REGULATION_OF_ANOIKIS	17	0.5201043	1.4811325	0.050336	0.08371	1	1004	tags=35%, list=10%, signal=39%

GO_REGULATION_OF_INTRINSIC_APOPTOTIC_SIGNALING_PATHWAY	GO_REGULATION_OF_INTRINSIC_APOPTOTIC_SIGNALING_PATHWAY	114	0.35094935	1.4787934	0.018987	0.08495	1	908	tags=18%, list=9%, signal=19%
GO_REGULATION_OF_OSTEOCLAST_DIFFERENTIATION	GO_REGULATION_OF_OSTEOCLAST_DIFFERENTIATION	45	0.4207967	1.4786029	0.023438	0.08489	1	1055	tags=24%, list=10%, signal=27%
GO_SUPEROXIDE_METABOLIC_PROCESS	GO_SUPEROXIDE_METABOLIC_PROCESS	22	0.49497122	1.4779979	0.042042	0.0851	1	743	tags=18%, list=7%, signal=20%
GO_REGULATION_OF_VESICLE_MEDIATED_TRANSPORT	GO_REGULATION_OF_VESICLE_MEDIATED_TRANSPORT	333	0.309183	1.477122	0	0.08545	1	1415	tags=26%, list=14%, signal=30%
GO_PROTEIN_O_LINKED_GLYCOSYLATION	GO_PROTEIN_O_LINKED_GLYCOSYLATION	60	0.396772	1.4758929	0.025424	0.08604	1	2093	tags=37%, list=20%, signal=46%
GO_MONOSACCHARIDE_TRANSPORT	GO_MONOSACCHARIDE_TRANSPORT	38	0.43545434	1.4757831	0.02847	0.08599	1	1193	tags=24%, list=11%, signal=27%
GO_REGULATED_EXOCYTOSIS	GO_REGULATED_EXOCYTOSIS	161	0.3446124	1.4751825	0	0.08612	1	1190	tags=27%, list=11%, signal=30%
GO_RESPONSE_TO_OXIDATIVE_STRESS	GO_RESPONSE_TO_OXIDATIVE_STRESS	285	0.31590182	1.4744276	0	0.08641	1	1343	tags=22%, list=13%, signal=25%
GO_REGULATION_OF_ODONTOGENESIS	GO_REGULATION_OF_ODONTOGENESIS	18	0.5225969	1.4737933	0.052117	0.0866	1	1475	tags=39%, list=14%, signal=45%
GO_LUNG_MORPHOGENESIS	GO_LUNG_MORPHOGENESIS	34	0.43882406	1.4724433	0.039146	0.0872	1	1435	tags=32%, list=14%, signal=37%
GO_RESPONSE_TO_AMPHETAMINE	GO_RESPONSE_TO_AMPHETAMINE	24	0.4803546	1.4719561	0.048611	0.08733	1	156	tags=8%, list=2%, signal=8%
GO_REGULATION_OF_INTERLEUKIN_17_PRODUCTION	GO_REGULATION_OF_INTERLEUKIN_17_PRODUCTION	17	0.525127	1.4680798	0.069767	0.08937	1	1297	tags=41%, list=12%, signal=47%
GO_REGULATION_OF_CALCIUM_MEDIATED_SIGNALING	GO_REGULATION_OF_CALCIUM_MEDIATED_SIGNALING	55	0.39623645	1.4678601	0.036036	0.08936	1	1369	tags=25%, list=13%, signal=29%
GO_RESPONSE_TO_INTERFERON_ALPHA	GO_RESPONSE_TO_INTERFERON_ALPHA	20	0.5051582	1.4674145	0.043771	0.08949	1	1275	tags=40%, list=12%, signal=46%

GO_REGULATION_OF_SYNAPTIC_TRANSMISSION_DOPAMINERGIC	GO_REGULATION_OF_SYNAPTIC_TRANSMISSION_DOPAMINERGIC	15	0.5324787	1.4656478	0.067742	0.09037	1	2466	tags=40%, list=24%, signal=52%
GO_ION_HOMEOSTASIS	GO_ION_HOMEOSTASIS	433	0.31208262	1.465105	0	0.09049	1	1399	tags=21%, list=13%, signal=23%
GO_AORTA_DEVELOPMENT	GO_AORTA_DEVELOPMENT	35	0.43470356	1.4633511	0.04059	0.09139	1	591	tags=20%, list=6%, signal=21%
GO_RESPONSE_TO_AMINE	GO_RESPONSE_TO_AMINE	39	0.42519698	1.4627118	0.024561	0.09163	1	353	tags=13%, list=3%, signal=13%
GO_MYELOID_CELL_ACTIVATION_INVOLVED_IN_IMMUNE_RESPONSE	GO_MYELOID_CELL_ACTIVATION_INVOLVED_IN_IMMUNE_RESPONSE	34	0.43083435	1.4622393	0.037594	0.09179	1	1176	tags=41%, list=11%, signal=46%
GO_REGULATION_OF_CYTOSOLIC_CALCIUM_ION_CONCENTRATION	GO_REGULATION_OF_CYTOSOLIC_CALCIUM_ION_CONCENTRATION	168	0.3343697	1.4592028	0	0.09342	1	1381	tags=23%, list=13%, signal=26%
GO_REGULATION_OF_SYSTEMIC_ARTERIAL_BLOOD_PRESSURE	GO_REGULATION_OF_SYSTEMIC_ARTERIAL_BLOOD_PRESSURE	75	0.3745199	1.4582647	0.017467	0.09382	1	1256	tags=17%, list=12%, signal=20%
GO_POSITIVE_REGULATION_OF_MULTICELLULAR_ORGANISMAL_METABOLIC_PROCESS	GO_POSITIVE_REGULATION_OF_MULTICELLULAR_ORGANISMAL_METABOLIC_PROCESS	19	0.49162018	1.4572598	0.054422	0.09428	1	1989	tags=47%, list=19%, signal=58%
GO_PH_REDUCTION	GO_PH_REDUCTION	28	0.45719084	1.4569763	0.052795	0.09433	1	653	tags=18%, list=6%, signal=19%
GO_POSITIVE_REGULATION_OF_TRANSFORMING_GROWTH_FACTOR_BETA_PRODUCTION	GO_POSITIVE_REGULATION_OF_TRANSFORMING_GROWTH_FACTOR_BETA_PRODUCTION	16	0.51998264	1.455493	0.075908	0.09512	1	1038	tags=31%, list=10%, signal=35%
GO_POSITIVE_REGULATION_OF_PROTEIN_SECRETION	GO_POSITIVE_REGULATION_OF_PROTEIN_SECRETION	161	0.3351197	1.455005	0	0.0952	1	1073	tags=21%, list=10%, signal=23%
GO_REGULATION_OF_AXON_GUIDANCE	GO_REGULATION_OF_AXON_GUIDANCE	33	0.43564782	1.4546791	0.034843	0.09527	1	1603	tags=30%, list=15%, signal=36%

GO_POSITIVE_REGULATION_OF_PROTEIN_LOCALIZATION_TO_NUCLEUS	GO_POSITIVE_REGULATION_OF_PROTEIN_LOCALIZATION_TO_NUCLEUS	100	0.35606572	1.4516852	0.021277	0.09697	1	664	tags=18%, list=6%, signal=19%
GO_REGULATION_OF_CELL_MATRIX_ADHESION	GO_REGULATION_OF_CELL_MATRIX_ADHESION	71	0.3757963	1.4506767	0.013953	0.09739	1	431	tags=10%, list=4%, signal=10%
GO_RESPONSE_TO_AMMONIUM_ION	GO_RESPONSE_TO_AMMONIUM_ION	40	0.4252402	1.4506289	0.03413	0.09726	1	1068	tags=23%, list=10%, signal=25%
GO_RESPONSE_TO_INTERLEUKIN_4	GO_RESPONSE_TO_INTERLEUKIN_4	26	0.46341753	1.4499745	0.058065	0.09754	1	2005	tags=50%, list=19%, signal=62%
GO_REGULATION_OF_DEFENSE_RESPONSE_TO_VIRUS_BY_VIRUS	GO_REGULATION_OF_DEFENSE_RESPONSE_TO_VIRUS_BY_VIRUS	23	0.48883763	1.4495604	0.06689	0.09764	1	569	tags=17%, list=5%, signal=18%
GO_POSITIVE_REGULATION_OF_ACTIN_FILAMENT_POLYMERIZATION	GO_POSITIVE_REGULATION_OF_ACTIN_FILAMENT_POLYMERIZATION	47	0.41005695	1.4494768	0.021818	0.09753	1	2299	tags=43%, list=22%, signal=54%
GO_REGULATION_OF_PROTEIN_KINASE_B_SIGNALING	GO_REGULATION_OF_PROTEIN_KINASE_B_SIGNALING	97	0.3579407	1.4485105	0.011111	0.09795	1	1905	tags=34%, list=18%, signal=41%
GO_MORPHOGENESIS_OF_AN_EPITHELIAL_SHEET	GO_MORPHOGENESIS_OF_AN_EPITHELIAL_SHEET	32	0.443406	1.4481418	0.044674	0.09804	1	422	tags=16%, list=4%, signal=16%
GO_BODY_MORPHOGENESIS	GO_BODY_MORPHOGENESIS	36	0.44046697	1.4463419	0.041985	0.09904	1	955	tags=25%, list=9%, signal=27%
GO_POSITIVE_REGULATION_OF_INTERLEUKIN_1_PRODUCTION	GO_POSITIVE_REGULATION_OF_INTERLEUKIN_1_PRODUCTION	24	0.46680242	1.4463239	0.047022	0.0989	1	1609	tags=46%, list=15%, signal=54%
GO_EPITHELIAL_TUBE_BRANCHING_INVOLVED_IN_LUNG_MORPHOGENESIS	GO_EPITHELIAL_TUBE_BRANCHING_INVOLVED_IN_LUNG_MORPHOGENESIS	18	0.5162593	1.4445462	0.080997	0.1	1	1997	tags=50%, list=19%, signal=62%

GO_REGULATION_OF_PATHWAY_RESTRICTED_SMAD_PROTEIN_PHOSPHORYLATION	GO_REGULATION_OF_PATHWAY_RESTRICTED_SMAD_PROTEIN_PHOSPHORYLATION	48	0.40523365	1.4445127	0.026718	0.09986	1	1330	tags=29%, list=13%, signal=33%
**Enriched pathways in GO biological process for CTSI low risk group**									
**NAME**	**GS<br>follow link to MSigDB**	**SIZE**	**ES**	**NES**	**NOM p-val**	**FDR q-val**	**FWER p-val**	**RANK AT MAX**	**LEADING EDGE**
GO_NEUROTRANSMITTER_TRANSPORT	GO_NEUROTRANSMITTER_TRANSPORT	110	-0.60994875	-2.1117325	0	0	0	1575	tags=35%, list=15%, signal=41%
GO_DNA_REPLICATION_INITIATION	GO_DNA_REPLICATION_INITIATION	21	-0.7975868	-2.110074	0	0	0	1488	tags=81%, list=14%, signal=94%
GO_DNA_DEPENDENT_DNA_REPLICATION	GO_DNA_DEPENDENT_DNA_REPLICATION	68	-0.6499204	-2.1073723	0	0	0	1707	tags=57%, list=16%, signal=68%
GO_DNA_REPLICATION	GO_DNA_REPLICATION	142	-0.5889851	-2.0987017	0	0	0	2181	tags=51%, list=21%, signal=64%
GO_PRESYNAPTIC_PROCESS_INVOLVED_IN_SYNAPTIC_TRANSMISSION	GO_PRESYNAPTIC_PROCESS_INVOLVED_IN_SYNAPTIC_TRANSMISSION	77	-0.627585	-2.0732167	0	0.00014	0.001	2159	tags=45%, list=21%, signal=57%
GO_SISTER_CHROMATID_SEGREGATION	GO_SISTER_CHROMATID_SEGREGATION	115	-0.58055043	-2.0193012	0	0.00135	0.012	3520	tags=72%, list=34%, signal=108%
GO_DNA_RECOMBINATION	GO_DNA_RECOMBINATION	134	-0.55498147	-2.007337	0	0.00163	0.017	2905	tags=60%, list=28%, signal=82%
GO_MICROTUBULE_BASED_MOVEMENT	GO_MICROTUBULE_BASED_MOVEMENT	118	-0.5618505	-1.9980433	0	0.00185	0.022	2359	tags=43%, list=23%, signal=55%
GO_NUCLEAR_CHROMOSOME_SEGREGATION	GO_NUCLEAR_CHROMOSOME_SEGREGATION	137	-0.55641353	-1.9891839	0	0.00202	0.027	3520	tags=65%, list=34%, signal=97%
GO_DNA_REPAIR	GO_DNA_REPAIR	315	-0.5232194	-1.9845372	0	0.00208	0.031	3457	tags=60%, list=33%, signal=87%
GO_REGULATION_OF_NEURONAL_SYNAPTIC_PLASTICITY	GO_REGULATION_OF_NEURONAL_SYNAPTIC_PLASTICITY	38	-0.6888381	-1.9839463	0	0.00189	0.031	2032	tags=50%, list=20%, signal=62%

GO_REGULATION_OF_SYNAPTIC_PLASTICITY	GO_REGULATION_OF_SYNAPTIC_PLASTICITY	113	-0.5603532	-1.9819453	0	0.0019	0.034	2087	tags=41%, list=20%, signal=50%
GO_DNA_BIOSYNTHETIC_PROCESS	GO_DNA_BIOSYNTHETIC_PROCESS	87	-0.5784222	-1.9774947	0	0.00191	0.037	2120	tags=48%, list=20%, signal=60%
GO_SYNAPTIC_SIGNALING	GO_SYNAPTIC_SIGNALING	330	-0.5161648	-1.9717891	0	0.00192	0.04	1618	tags=25%, list=16%, signal=28%
GO_CELL_CYCLE_PHASE_TRANSITION	GO_CELL_CYCLE_PHASE_TRANSITION	195	-0.5285128	-1.966958	0	0.00193	0.043	3013	tags=55%, list=29%, signal=77%
GO_SISTER_CHROMATID_COHESION	GO_SISTER_CHROMATID_COHESION	67	-0.59386736	-1.9368262	0	0.00286	0.067	3520	tags=76%, list=34%, signal=114%
GO_MITOTIC_NUCLEAR_DIVISION	GO_MITOTIC_NUCLEAR_DIVISION	230	-0.5121585	-1.927917	0	0.00325	0.08	2831	tags=50%, list=27%, signal=67%
GO_DNA_METABOLIC_PROCESS	GO_DNA_METABOLIC_PROCESS	482	-0.49058723	-1.92547	0	0.00329	0.085	3457	tags=54%, list=33%, signal=78%
GO_MITOTIC_SISTER_CHROMATID_SEGREGATION	GO_MITOTIC_SISTER_CHROMATID_SEGREGATION	64	-0.59597254	-1.92419	0	0.00322	0.088	3445	tags=75%, list=33%, signal=112%
GO_ORGANELLE_FISSION	GO_ORGANELLE_FISSION	300	-0.5050365	-1.92116	0	0.00323	0.092	3035	tags=50%, list=29%, signal=68%
GO_DNA_STRAND_ELONGATION	GO_DNA_STRAND_ELONGATION	28	-0.68906146	-1.9131372	0	0.00391	0.116	1371	tags=57%, list=13%, signal=66%
GO_CHROMOSOME_SEGREGATION	GO_CHROMOSOME_SEGREGATION	161	-0.5261674	-1.908785	0	0.00385	0.12	3619	tags=63%, list=35%, signal=96%
GO_DOUBLE_STRAND_BREAK_REPAIR	GO_DOUBLE_STRAND_BREAK_REPAIR	100	-0.55252594	-1.9080225	0	0.00374	0.122	2905	tags=55%, list=28%, signal=76%
GO_GLUTAMATE_SECRETION	GO_GLUTAMATE_SECRETION	27	-0.6968788	-1.9063313	0	0.00381	0.13	1653	tags=44%, list=16%, signal=53%
GO_DNA_SYNTHESIS_INVOLVED_IN_DNA_REPAIR	GO_DNA_SYNTHESIS_INVOLVED_IN_DNA_REPAIR	58	-0.5923501	-1.9044933	0	0.00385	0.137	2120	tags=50%, list=20%, signal=62%
GO_NEURON_FATE_SPECIFICATION	GO_NEURON_FATE_SPECIFICATION	20	-0.74736977	-1.9030776	0	0.0038	0.14	344	tags=30%, list=3%, signal=31%

GO_REGULATION_OF_NEUROTRANSMITTER_LEVELS	GO_REGULATION_OF_NEUROTRANSMITTER_LEVELS	135	-0.53523743	-1.8861632	0	0.00493	0.183	2078	tags=36%, list=20%, signal=44%
GO_DNA_STRAND_ELONGATION_INVOLVED_IN_DNA_REPLICATION	GO_DNA_STRAND_ELONGATION_INVOLVED_IN_DNA_REPLICATION	23	-0.7138768	-1.8782423	0.001379	0.00567	0.213	1371	tags=65%, list=13%, signal=75%
GO_DNA_CONFORMATION_CHANGE	GO_DNA_CONFORMATION_CHANGE	173	-0.5102415	-1.8726115	0	0.00599	0.23	3233	tags=50%, list=31%, signal=71%
GO_REGULATION_OF_AMINO_ACID_TRANSPORT	GO_REGULATION_OF_AMINO_ACID_TRANSPORT	24	-0.6909995	-1.860886	0	0.00749	0.288	581	tags=25%, list=6%, signal=26%
GO_CALCIUM_ION_REGULATED_EXOCYTOSIS	GO_CALCIUM_ION_REGULATED_EXOCYTOSIS	44	-0.6133337	-1.850378	0	0.00918	0.347	1575	tags=39%, list=15%, signal=45%
GO_REGULATION_OF_SYNAPSE_STRUCTURE_OR_ACTIVITY	GO_REGULATION_OF_SYNAPSE_STRUCTURE_OR_ACTIVITY	170	-0.5011971	-1.839157	0	0.01102	0.41	1898	tags=36%, list=18%, signal=43%
GO_MICROTUBULE_BASED_PROCESS	GO_MICROTUBULE_BASED_PROCESS	305	-0.48066422	-1.8381642	0	0.01081	0.416	2444	tags=40%, list=24%, signal=50%
GO_CELL_DIVISION	GO_CELL_DIVISION	294	-0.48031303	-1.8379864	0	0.01049	0.416	2586	tags=42%, list=25%, signal=54%
GO_MRNA_PROCESSING	GO_MRNA_PROCESSING	239	-0.48620018	-1.8294452	0	0.01173	0.461	4028	tags=62%, list=39%, signal=100%
GO_CELL_CYCLE_G2_M_PHASE_TRANSITION	GO_CELL_CYCLE_G2_M_PHASE_TRANSITION	105	-0.5315964	-1.8275075	0	0.01174	0.469	3619	tags=63%, list=35%, signal=96%
GO_SYNAPTIC_VESICLE_CYCLE	GO_SYNAPTIC_VESICLE_CYCLE	50	-0.59206975	-1.8262213	0	0.01175	0.478	1575	tags=34%, list=15%, signal=40%
GO_RNA_SPLICING_VIA_TRANSESTERIFICATION_REACTIONS	GO_RNA_SPLICING_VIA_TRANSESTERIFICATION_REACTIONS	156	-0.5036617	-1.8153831	0	0.01361	0.536	4060	tags=67%, list=39%, signal=109%
GO_SYNAPSE_ORGANIZATION	GO_SYNAPSE_ORGANIZATION	110	-0.51663953	-1.8141208	0	0.01359	0.54	1612	tags=32%, list=16%, signal=37%
GO_SIGNAL_RELEASE	GO_SIGNAL_RELEASE	120	-0.51439536	-1.8099134	0	0.01436	0.576	2442	tags=43%, list=24%, signal=55%

GO_PROTEIN_DNA_COMPLEX_SUBUNIT_ORGANIZATION	GO_PROTEIN_DNA_COMPLEX_SUBUNIT_ORGANIZATION	149	-0.5071431	-1.8086677	0	0.01432	0.583	3216	tags=50%, list=31%, signal=72%
GO_ASSOCIATIVE_LEARNING	GO_ASSOCIATIVE_LEARNING	58	-0.5739669	-1.8064489	0	0.0145	0.599	670	tags=19%, list=6%, signal=20%
GO_NUCLEOTIDE_EXCISION_REPAIR	GO_NUCLEOTIDE_EXCISION_REPAIR	96	-0.52482784	-1.805416	0	0.01441	0.604	3598	tags=61%, list=35%, signal=93%
GO_CHROMATIN_MODIFICATION	GO_CHROMATIN_MODIFICATION	335	-0.46779242	-1.8020967	0	0.0152	0.632	3520	tags=51%, list=34%, signal=75%
GO_CHROMATIN_ORGANIZATION	GO_CHROMATIN_ORGANIZATION	406	-0.46407315	-1.8006163	0	0.01526	0.646	3553	tags=50%, list=34%, signal=73%
GO_CELL_DIFFERENTIATION_IN_SPINAL_CORD	GO_CELL_DIFFERENTIATION_IN_SPINAL_CORD	31	-0.6329525	-1.8002409	0	0.015	0.647	1158	tags=29%, list=11%, signal=33%
GO_MICROTUBULE_ORGANIZING_CENTER_ORGANIZATION	GO_MICROTUBULE_ORGANIZING_CENTER_ORGANIZATION	55	-0.5718734	-1.7991631	0.001297	0.01503	0.655	2797	tags=60%, list=27%, signal=82%
GO_ACIDIC_AMINO_ACID_TRANSPORT	GO_ACIDIC_AMINO_ACID_TRANSPORT	19	-0.7065923	-1.7988905	0.001435	0.01483	0.659	1689	tags=47%, list=16%, signal=56%
GO_RNA_SPLICING	GO_RNA_SPLICING	201	-0.48656645	-1.7984214	0	0.01465	0.661	4110	tags=65%, list=40%, signal=106%
GO_SPINAL_CORD_MOTOR_NEURON_DIFFERENTIATION	GO_SPINAL_CORD_MOTOR_NEURON_DIFFERENTIATION	21	-0.6969785	-1.7959515	0	0.0151	0.677	853	tags=33%, list=8%, signal=36%
GO_SPINAL_CORD_DEVELOPMENT	GO_SPINAL_CORD_DEVELOPMENT	74	-0.54172814	-1.7958883	0	0.0148	0.677	1212	tags=26%, list=12%, signal=29%
GO_PEPTIDYL_LYSINE_MODIFICATION	GO_PEPTIDYL_LYSINE_MODIFICATION	208	-0.4799206	-1.7949861	0	0.0148	0.685	3814	tags=62%, list=37%, signal=96%
GO_MEIOSIS_I	GO_MEIOSIS_I	48	-0.585975	-1.7936548	0	0.01485	0.694	3013	tags=54%, list=29%, signal=76%
GO_MICROTUBULE_CYTOSKELETON_ORGANIZATION	GO_MICROTUBULE_CYTOSKELETON_ORGANIZATION	206	-0.48360333	-1.7931057	0	0.01473	0.698	2622	tags=44%, list=25%, signal=57%

GO_SERINE_FAMILY_AMINO_ACID_METABOLIC_PROCESS	GO_SERINE_FAMILY_AMINO_ACID_METABOLIC_PROCESS	29	-0.64937	-1.792068	0	0.01467	0.706	2035	tags=45%, list=20%, signal=56%
GO_LEARNING	GO_LEARNING	108	-0.5142497	-1.7856746	0	0.01582	0.737	1250	tags=27%, list=12%, signal=30%
GO_NEURONAL_ACTION_POTENTIAL	GO_NEURONAL_ACTION_POTENTIAL	24	-0.6653064	-1.7853104	0	0.01564	0.738	125	tags=13%, list=1%, signal=13%
GO_ESTABLISHMENT_OF_LOCALIZATION_BY_MOVEMENT_ALONG_MICROTUBULE	GO_ESTABLISHMENT_OF_LOCALIZATION_BY_MOVEMENT_ALONG_MICROTUBULE	62	-0.55830026	-1.7820535	0	0.01631	0.761	2359	tags=45%, list=23%, signal=58%
GO_MITOTIC_CYTOKINESIS	GO_MITOTIC_CYTOKINESIS	23	-0.66297346	-1.7794988	0.001481	0.01666	0.776	2829	tags=70%, list=27%, signal=95%
GO_REGULATION_OF_LONG_TERM_NEURONAL_SYNAPTIC_PLASTICITY	GO_REGULATION_OF_LONG_TERM_NEURONAL_SYNAPTIC_PLASTICITY	19	-0.69937354	-1.7772403	0	0.01702	0.787	2032	tags=53%, list=20%, signal=65%
GO_CILIUM_ORGANIZATION	GO_CILIUM_ORGANIZATION	91	-0.5197329	-1.7733922	0	0.01775	0.805	2338	tags=44%, list=23%, signal=56%
GO_ADULT_BEHAVIOR	GO_ADULT_BEHAVIOR	111	-0.5050152	-1.7728698	0	0.01764	0.806	1163	tags=24%, list=11%, signal=27%
GO_NEUROPEPTIDE_SIGNALING_PATHWAY	GO_NEUROPEPTIDE_SIGNALING_PATHWAY	61	-0.5548348	-1.7700727	0.002621	0.01815	0.822	1062	tags=21%, list=10%, signal=24%
GO_MITOTIC_RECOMBINATION	GO_MITOTIC_RECOMBINATION	35	-0.6120607	-1.7677435	0	0.01854	0.831	2287	tags=63%, list=22%, signal=80%
GO_PHOTORECEPTOR_CELL_MAINTENANCE	GO_PHOTORECEPTOR_CELL_MAINTENANCE	20	-0.6740725	-1.7675155	0.002954	0.01834	0.835	1292	tags=30%, list=12%, signal=34%
GO_REGULATION_OF_MICROTUBULE_BASED_PROCESS	GO_REGULATION_OF_MICROTUBULE_BASED_PROCESS	164	-0.48464823	-1.76536	0	0.01865	0.843	1723	tags=35%, list=17%, signal=42%
GO_MEMBRANE_DEPOLARIZATION_DURING_ACTION_POTENTIAL	GO_MEMBRANE_DEPOLARIZATION_DURING_ACTION_POTENTIAL	31	-0.61923844	-1.7652961	0.001406	0.01838	0.843	786	tags=16%, list=8%, signal=17%
GO_CYTOSKELETON_DEPENDENT_CYTOKINESIS	GO_CYTOSKELETON_DEPENDENT_CYTOKINESIS	28	-0.6344146	-1.7651484	0.001451	0.01812	0.843	3138	tags=68%, list=30%, signal=97%

GO_CENTROSOME_CYCLE	GO_CENTROSOME_CYCLE	33	-0.61982924	-1.7623767	0	0.01855	0.852	2797	tags=73%, list=27%, signal=99%
GO_MODULATION_OF_SYNAPTIC_TRANSMISSION	GO_MODULATION_OF_SYNAPTIC_TRANSMISSION	243	-0.46861193	-1.7603544	0	0.01888	0.861	1351	tags=25%, list=13%, signal=28%
GO_DNA_GEOMETRIC_CHANGE	GO_DNA_GEOMETRIC_CHANGE	66	-0.53600085	-1.7551646	0	0.02009	0.888	3181	tags=53%, list=31%, signal=76%
GO_POSTSYNAPTIC_MEMBRANE_ORGANIZATION	GO_POSTSYNAPTIC_MEMBRANE_ORGANIZATION	22	-0.6675326	-1.7529126	0.00142	0.02055	0.895	2078	tags=50%, list=20%, signal=62%
GO_DNA_PACKAGING	GO_DNA_PACKAGING	109	-0.5064335	-1.7529007	0	0.02027	0.895	3282	tags=50%, list=32%, signal=73%
GO_COVALENT_CHROMATIN_MODIFICATION	GO_COVALENT_CHROMATIN_MODIFICATION	219	-0.4688557	-1.7519892	0	0.02018	0.901	3442	tags=53%, list=33%, signal=77%
GO_OLIGODENDROCYTE_DEVELOPMENT	GO_OLIGODENDROCYTE_DEVELOPMENT	27	-0.6429396	-1.7510072	0	0.02019	0.904	555	tags=30%, list=5%, signal=31%
GO_MEIOTIC_CELL_CYCLE	GO_MEIOTIC_CELL_CYCLE	107	-0.5019751	-1.7475973	0	0.02079	0.912	3422	tags=51%, list=33%, signal=76%
GO_REGULATION_OF_CALCIUM_ION_DEPENDENT_EXOCYTOSIS	GO_REGULATION_OF_CALCIUM_ION_DEPENDENT_EXOCYTOSIS	46	-0.5730305	-1.7419864	0	0.02231	0.93	2071	tags=39%, list=20%, signal=49%
GO_CENTROSOME_DUPLICATION	GO_CENTROSOME_DUPLICATION	22	-0.6627423	-1.7414756	0.004348	0.02221	0.931	2797	tags=82%, list=27%, signal=112%
GO_CENTROMERE_COMPLEX_ASSEMBLY	GO_CENTROMERE_COMPLEX_ASSEMBLY	23	-0.66435564	-1.740985	0.004304	0.02201	0.931	2547	tags=61%, list=25%, signal=80%
GO_CELL_CYCLE_G1_S_PHASE_TRANSITION	GO_CELL_CYCLE_G1_S_PHASE_TRANSITION	85	-0.52083206	-1.7408187	0.001271	0.02178	0.932	3000	tags=56%, list=29%, signal=79%
GO_MITOTIC_SPINDLE_ORGANIZATION	GO_MITOTIC_SPINDLE_ORGANIZATION	51	-0.5623098	-1.7375348	0	0.02253	0.938	1661	tags=45%, list=16%, signal=53%
GO_PROTEIN_LOCALIZATION_TO_CHROMOSOME	GO_PROTEIN_LOCALIZATION_TO_CHROMOSOME	29	-0.6150204	-1.7374325	0.001404	0.0223	0.938	1951	tags=52%, list=19%, signal=64%

GO_DEOXYRIBONUCLEOTIDE_METABOLIC_PROCESS	GO_DEOXYRIBONUCLEOTIDE_METABOLIC_PROCESS	26	-0.63209677	-1.7371285	0.001389	0.02212	0.938	2424	tags=62%, list=23%, signal=80%
GO_NEURON_FATE_COMMITMENT	GO_NEURON_FATE_COMMITMENT	44	-0.5748696	-1.7356397	0.002789	0.02246	0.941	2748	tags=43%, list=26%, signal=58%
GO_RECOMBINATIONAL_REPAIR	GO_RECOMBINATIONAL_REPAIR	44	-0.5734423	-1.7354108	0.001377	0.02227	0.942	2901	tags=64%, list=28%, signal=88%
GO_OLIGODENDROCYTE_DIFFERENTIATION	GO_OLIGODENDROCYTE_DIFFERENTIATION	46	-0.57045037	-1.7342339	0	0.02248	0.944	1756	tags=37%, list=17%, signal=44%
GO_REGULATION_OF_SYNAPTIC_VESICLE_TRANSPORT	GO_REGULATION_OF_SYNAPTIC_VESICLE_TRANSPORT	23	-0.6581119	-1.7331682	0.001408	0.0226	0.948	1909	tags=48%, list=18%, signal=58%
GO_G1_S_TRANSITION_OF_MITOTIC_CELL_CYCLE	GO_G1_S_TRANSITION_OF_MITOTIC_CELL_CYCLE	85	-0.520832	-1.7328017	0	0.02249	0.95	3000	tags=56%, list=29%, signal=79%
GO_REGULATION_OF_DNA_DEPENDENT_DNA_REPLICATION	GO_REGULATION_OF_DNA_DEPENDENT_DNA_REPLICATION	30	-0.61727065	-1.7314986	0.002817	0.02268	0.956	3396	tags=80%, list=33%, signal=119%
GO_HIPPOCAMPUS_DEVELOPMENT	GO_HIPPOCAMPUS_DEVELOPMENT	58	-0.5437901	-1.7314935	0.001276	0.02243	0.956	1846	tags=29%, list=18%, signal=35%
GO_SPINDLE_CHECKPOINT	GO_SPINDLE_CHECKPOINT	20	-0.67064404	-1.7288446	0.004673	0.02288	0.962	2332	tags=65%, list=22%, signal=84%
GO_RNA_PROCESSING	GO_RNA_PROCESSING	453	-0.44244117	-1.7268481	0	0.02322	0.965	3925	tags=54%, list=38%, signal=83%
GO_PEPTIDYL_LYSINE_METHYLATION	GO_PEPTIDYL_LYSINE_METHYLATION	43	-0.57587016	-1.7226262	0	0.0243	0.968	2641	tags=51%, list=25%, signal=68%
GO_NON_RECOMBINATIONAL_REPAIR	GO_NON_RECOMBINATIONAL_REPAIR	46	-0.5622888	-1.719077	0.002714	0.02521	0.971	3661	tags=65%, list=35%, signal=100%
GO_HISTONE_EXCHANGE	GO_HISTONE_EXCHANGE	27	-0.62410027	-1.7165142	0.00141	0.02603	0.977	2547	tags=56%, list=25%, signal=73%
GO_POSITIVE_REGULATION_OF_AMINE_TRANSPORT	GO_POSITIVE_REGULATION_OF_AMINE_TRANSPORT	31	-0.60519207	-1.7151887	0.005706	0.02624	0.978	581	tags=23%, list=6%, signal=24%

GO_VENTRAL_SPINAL_CORD_DEVELOPMENT	GO_VENTRAL_SPINAL_CORD_DEVELOPMENT	29	-0.61721164	-1.7144866	0.004219	0.02623	0.978	853	tags=28%, list=8%, signal=30%
GO_REGULATION_OF_DENDRITIC_SPINE_MORPHOGENESIS	GO_REGULATION_OF_DENDRITIC_SPINE_MORPHOGENESIS	22	-0.6467332	-1.7144009	0.007102	0.02601	0.979	1618	tags=50%, list=16%, signal=59%
GO_PHOTORECEPTOR_CELL_DIFFERENTIATION	GO_PHOTORECEPTOR_CELL_DIFFERENTIATION	37	-0.583189	-1.7087897	0.004202	0.02796	0.986	463	tags=19%, list=4%, signal=20%
GO_REGULATION_OF_CHROMOSOME_SEGREGATION	GO_REGULATION_OF_CHROMOSOME_SEGREGATION	61	-0.5358365	-1.707866	0.00266	0.02807	0.987	2332	tags=49%, list=22%, signal=63%
GO_EYE_PHOTORECEPTOR_CELL_DIFFERENTIATION	GO_EYE_PHOTORECEPTOR_CELL_DIFFERENTIATION	31	-0.60750455	-1.7053093	0.001439	0.02892	0.989	463	tags=19%, list=4%, signal=20%
GO_LOCOMOTORY_BEHAVIOR	GO_LOCOMOTORY_BEHAVIOR	139	-0.48230326	-1.7035348	0	0.02941	0.991	1311	tags=22%, list=13%, signal=25%
GO_MEIOTIC_CELL_CYCLE_PROCESS	GO_MEIOTIC_CELL_CYCLE_PROCESS	88	-0.5042811	-1.7011739	0.001225	0.03015	0.992	2905	tags=45%, list=28%, signal=63%
GO_TELOMERE_MAINTENANCE_VIA_RECOMBINATION	GO_TELOMERE_MAINTENANCE_VIA_RECOMBINATION	29	-0.61199206	-1.6992509	0.004249	0.03065	0.994	2120	tags=62%, list=20%, signal=78%
GO_NEUROTRANSMITTER_UPTAKE	GO_NEUROTRANSMITTER_UPTAKE	15	-0.6875614	-1.696881	0.002915	0.03134	0.994	259	tags=20%, list=2%, signal=20%
GO_REGULATION_OF_POSTSYNAPTIC_MEMBRANE_POTENTIAL	GO_REGULATION_OF_POSTSYNAPTIC_MEMBRANE_POTENTIAL	44	-0.5671721	-1.693491	0.002706	0.03247	0.995	1909	tags=27%, list=18%, signal=33%
GO_WNT_SIGNALING_PATHWAY_CALCIUM_MODULATING_PATHWAY	GO_WNT_SIGNALING_PATHWAY_CALCIUM_MODULATING_PATHWAY	29	-0.59067327	-1.6906465	0	0.03342	0.997	1975	tags=41%, list=19%, signal=51%
GO_SYNAPTIC_VESICLE_LOCALIZATION	GO_SYNAPTIC_VESICLE_LOCALIZATION	59	-0.5380518	-1.6846192	0	0.0359	0.998	1575	tags=31%, list=15%, signal=36%
GO_HISTONE_METHYLATION	GO_HISTONE_METHYLATION	56	-0.53318083	-1.6837946	0.001295	0.03599	0.998	3363	tags=57%, list=32%, signal=84%
GO_PROTEIN_ALKYLATION	GO_PROTEIN_ALKYLATION	72	-0.5049704	-1.6801455	0.001304	0.0375	0.998	3363	tags=54%, list=32%, signal=80%

GO_CELL_CYCLE_CHECKPOINT	GO_CELL_CYCLE_CHECKPOINT	146	-0.4657936	-1.6799858	0	0.03723	0.998	3599	tags=54%, list=35%, signal=82%
GO_SODIUM_ION_TRANSMEMBRANE_TRANSPORT	GO_SODIUM_ION_TRANSMEMBRANE_TRANSPORT	60	-0.51898974	-1.679355	0.002538	0.03718	0.998	425	tags=13%, list=4%, signal=14%
GO_CYTOSKELETON_DEPENDENT_INTRACELLULAR_TRANSPORT	GO_CYTOSKELETON_DEPENDENT_INTRACELLULAR_TRANSPORT	74	-0.51316226	-1.6784394	0	0.03725	0.998	2359	tags=41%, list=23%, signal=52%
GO_CILIUM_MORPHOGENESIS	GO_CILIUM_MORPHOGENESIS	96	-0.4915833	-1.6776	0	0.03735	0.998	2338	tags=43%, list=23%, signal=55%
GO_TRANSCRIPTION_COUPLED_NUCLEOTIDE_EXCISION_REPAIR	GO_TRANSCRIPTION_COUPLED_NUCLEOTIDE_EXCISION_REPAIR	67	-0.5196799	-1.6769449	0.001337	0.03736	0.998	3989	tags=69%, list=38%, signal=111%
GO_NEGATIVE_REGULATION_OF_LYASE_ACTIVITY	GO_NEGATIVE_REGULATION_OF_LYASE_ACTIVITY	24	-0.62373316	-1.6764512	0.004237	0.03727	0.998	1812	tags=33%, list=17%, signal=40%
GO_DICARBOXYLIC_ACID_TRANSPORT	GO_DICARBOXYLIC_ACID_TRANSPORT	60	-0.5227084	-1.6749281	0.001282	0.03756	0.998	1689	tags=32%, list=16%, signal=38%
GO_TELOMERE_ORGANIZATION	GO_TELOMERE_ORGANIZATION	68	-0.509877	-1.6725433	0	0.03841	0.998	2314	tags=41%, list=22%, signal=53%
GO_BASE_EXCISION_REPAIR	GO_BASE_EXCISION_REPAIR	34	-0.58254915	-1.6662319	0.002721	0.04113	0.998	3649	tags=76%, list=35%, signal=118%
GO_LIMBIC_SYSTEM_DEVELOPMENT	GO_LIMBIC_SYSTEM_DEVELOPMENT	82	-0.49891135	-1.6653504	0	0.04123	0.998	655	tags=17%, list=6%, signal=18%
GO_DENDRITE_MORPHOGENESIS	GO_DENDRITE_MORPHOGENESIS	35	-0.55795735	-1.6637566	0.002699	0.04173	0.999	1767	tags=37%, list=17%, signal=45%
GO_DORSAL_VENTRAL_AXIS_SPECIFICATION	GO_DORSAL_VENTRAL_AXIS_SPECIFICATION	18	-0.6589688	-1.6633127	0.005822	0.04162	0.999	1471	tags=44%, list=14%, signal=52%
GO_GAMMA_AMINOBUTYRIC_ACID_SIGNALING_PATHWAY	GO_GAMMA_AMINOBUTYRIC_ACID_SIGNALING_PATHWAY	20	-0.6498352	-1.6631439	0.002813	0.04141	0.999	810	tags=30%, list=8%, signal=32%
GO_RECIPROCAL_MEIOTIC_RECOMBINATION	GO_RECIPROCAL_MEIOTIC_RECOMBINATION	25	-0.60781056	-1.6599327	0.002747	0.04283	1	2905	tags=60%, list=28%, signal=83%

GO_CHROMATIN_ASSEMBLY_OR_DISASSEMBLY	GO_CHROMATIN_ASSEMBLY_OR_DISASSEMBLY	102	-0.47951147	-1.6573409	0.001222	0.04387	1	3369	tags=48%, list=32%, signal=70%
GO_STRAND_DISPLACEMENT	GO_STRAND_DISPLACEMENT	20	-0.63182944	-1.6568863	0.011544	0.04378	1	2110	tags=60%, list=20%, signal=75%
GO_DNA_TEMPLATED_TRANSCRIPTION_TERMINATION	GO_DNA_TEMPLATED_TRANSCRIPTION_TERMINATION	64	-0.5128919	-1.6560317	0.002567	0.04391	1	3863	tags=72%, list=37%, signal=114%
GO_DENDRITIC_SPINE_DEVELOPMENT	GO_DENDRITIC_SPINE_DEVELOPMENT	16	-0.6747406	-1.6542792	0.008785	0.04466	1	2952	tags=69%, list=28%, signal=96%
GO_POSTREPLICATION_REPAIR	GO_POSTREPLICATION_REPAIR	39	-0.5697792	-1.654049	0	0.04448	1	2478	tags=54%, list=24%, signal=70%
GO_TRNA_PROCESSING	GO_TRNA_PROCESSING	56	-0.518464	-1.6528107	0.001323	0.04487	1	4235	tags=73%, list=41%, signal=123%
GO_HISTONE_H4_ACETYLATION	GO_HISTONE_H4_ACETYLATION	27	-0.61212516	-1.6525458	0.008487	0.04467	1	3574	tags=81%, list=34%, signal=124%
GO_PROTEIN_METHYLATION	GO_PROTEIN_METHYLATION	72	-0.5049704	-1.6524839	0.001232	0.04435	1	3363	tags=54%, list=32%, signal=80%
GO_CENTRAL_NERVOUS_SYSTEM_NEURON_DIFFERENTIATION	GO_CENTRAL_NERVOUS_SYSTEM_NEURON_DIFFERENTIATION	118	-0.47096172	-1.6516094	0	0.04453	1	1051	tags=23%, list=10%, signal=25%
GO_CELLULAR_PROTEIN_COMPLEX_DISASSEMBLY	GO_CELLULAR_PROTEIN_COMPLEX_DISASSEMBLY	75	-0.50020695	-1.6510571	0.0025	0.0446	1	3916	tags=65%, list=38%, signal=104%
GO_RECIPROCAL_DNA_RECOMBINATION	GO_RECIPROCAL_DNA_RECOMBINATION	25	-0.60781056	-1.6502768	0.008811	0.04468	1	2905	tags=60%, list=28%, signal=83%
GO_CELLULAR_MACROMOLECULAR_COMPLEX_ASSEMBLY	GO_CELLULAR_MACROMOLECULAR_COMPLEX_ASSEMBLY	455	-0.42465997	-1.6499248	0	0.04455	1	3350	tags=44%, list=32%, signal=62%
GO_DNA_REPLICATION_INDEPENDENT_NUCLEOSOME_ASSEMBLY	GO_DNA_REPLICATION_INDEPENDENT_NUCLEOSOME_ASSEMBLY	26	-0.60584265	-1.6472608	0.008772	0.04574	1	2547	tags=50%, list=25%, signal=66%
GO_RIBONUCLEOPROTEIN_COMPLEX_LOCALIZATION	GO_RIBONUCLEOPROTEIN_COMPLEX_LOCALIZATION	73	-0.49880198	-1.6458391	0	0.04623	1	3944	tags=67%, list=38%, signal=107%

GO_CHROMATIN_REMODELING	GO_CHROMATIN_REMODELING	91	-0.48551896	-1.6447123	0	0.04655	1	3216	tags=49%, list=31%, signal=71%
GO_REGULATION_OF_CELL_CYCLE_G2_M_PHASE_TRANSITION	GO_REGULATION_OF_CELL_CYCLE_G2_M_PHASE_TRANSITION	40	-0.5574257	-1.6445636	0.00277	0.04627	1	2086	tags=40%, list=20%, signal=50%
GO_POSITIVE_REGULATION_OF_DENDRITE_MORPHOGENESIS	GO_POSITIVE_REGULATION_OF_DENDRITE_MORPHOGENESIS	22	-0.62603533	-1.6429238	0.005848	0.04686	1	946	tags=32%, list=9%, signal=35%
GO_TERMINATION_OF_RNA_POLYMERASE_II_TRANSCRIPTION	GO_TERMINATION_OF_RNA_POLYMERASE_II_TRANSCRIPTION	35	-0.56719726	-1.642415	0.002721	0.04685	1	3863	tags=77%, list=37%, signal=122%
GO_DNA_REPLICATION_INDEPENDENT_NUCLEOSOME_ORGANIZATION	GO_DNA_REPLICATION_INDEPENDENT_NUCLEOSOME_ORGANIZATION	26	-0.60584265	-1.641523	0.004243	0.04701	1	2547	tags=50%, list=25%, signal=66%
GO_MITOCHONDRIAL_TRANSLATION	GO_MITOCHONDRIAL_TRANSLATION	60	-0.51352626	-1.6385816	0.003807	0.04832	1	3916	tags=68%, list=38%, signal=109%
GO_CHROMOSOME_ORGANIZATION_INVOLVED_IN_MEIOTIC_CELL_CYCLE	GO_CHROMOSOME_ORGANIZATION_INVOLVED_IN_MEIOTIC_CELL_CYCLE	24	-0.6149021	-1.6384907	0.011445	0.04802	1	2141	tags=46%, list=21%, signal=58%
GO_POSITIVE_REGULATION_OF_CYTOKINESIS	GO_POSITIVE_REGULATION_OF_CYTOKINESIS	25	-0.6154734	-1.6369774	0.005772	0.04847	1	1661	tags=44%, list=16%, signal=52%
GO_REGULATION_OF_CYTOKINESIS	GO_REGULATION_OF_CYTOKINESIS	43	-0.5356775	-1.6357064	0.001316	0.04887	1	2169	tags=44%, list=21%, signal=56%
GO_REGULATION_OF_MICROTUBULE_POLYMERIZATION_OR_DEPOLYMERIZATION	GO_REGULATION_OF_MICROTUBULE_POLYMERIZATION_OR_DEPOLYMERIZATION	121	-0.4622777	-1.6344668	0.002407	0.04927	1	2558	tags=45%, list=25%, signal=59%
GO_REGULATION_OF_SYNAPTIC_VESICLE_EXOCYTOSIS	GO_REGULATION_OF_SYNAPTIC_VESICLE_EXOCYTOSIS	15	-0.67909116	-1.6325991	0.007704	0.05007	1	1909	tags=53%, list=18%, signal=65%
GO_RIBONUCLEOPROTEIN_COMPLEX_SUBUNIT_ORGANIZATION	GO_RIBONUCLEOPROTEIN_COMPLEX_SUBUNIT_ORGANIZATION	110	-0.46648744	-1.629322	0.00235	0.05182	1	3399	tags=50%, list=33%, signal=74%

GO_ISOPRENOID_BIOSYNTHETIC_PROCESS	GO_ISOPRENOID_BIOSYNTHETIC_PROCESS	19	-0.62920946	-1.6267852	0.008837	0.05302	1	1040	tags=32%, list=10%, signal=35%
GO_MEIOTIC_CHROMOSOME_SEGREGATION	GO_MEIOTIC_CHROMOSOME_SEGREGATION	30	-0.57740694	-1.6261411	0.004196	0.05315	1	2141	tags=40%, list=21%, signal=50%
GO_QUINONE_METABOLIC_PROCESS	GO_QUINONE_METABOLIC_PROCESS	23	-0.6146844	-1.6258069	0.007278	0.05299	1	2208	tags=48%, list=21%, signal=61%
GO_POSITIVE_REGULATION_OF_DENDRITIC_SPINE_DEVELOPMENT	GO_POSITIVE_REGULATION_OF_DENDRITIC_SPINE_DEVELOPMENT	23	-0.61643267	-1.6247624	0.007062	0.05319	1	2451	tags=61%, list=24%, signal=79%
GO_POSITIVE_REGULATION_OF_DENDRITE_DEVELOPMENT	GO_POSITIVE_REGULATION_OF_DENDRITE_DEVELOPMENT	43	-0.55168724	-1.6233658	0.001366	0.05379	1	2451	tags=49%, list=24%, signal=64%
GO_TRNA_METABOLIC_PROCESS	GO_TRNA_METABOLIC_PROCESS	93	-0.47690874	-1.6219015	0.001225	0.0545	1	3980	tags=57%, list=38%, signal=92%
GO_DNA_TEMPLATED_TRANSCRIPTION_ELONGATION	GO_DNA_TEMPLATED_TRANSCRIPTION_ELONGATION	79	-0.4867381	-1.6196926	0.002503	0.05577	1	3335	tags=58%, list=32%, signal=85%
GO_PROTEIN_SUMOYLATION	GO_PROTEIN_SUMOYLATION	92	-0.48062354	-1.6196039	0	0.05545	1	3085	tags=51%, list=30%, signal=72%
GO_VISUAL_BEHAVIOR	GO_VISUAL_BEHAVIOR	38	-0.5586793	-1.6188737	0.01257	0.05561	1	670	tags=24%, list=6%, signal=25%
GO_LUNG_EPITHELIUM_DEVELOPMENT	GO_LUNG_EPITHELIUM_DEVELOPMENT	25	-0.60672283	-1.6182945	0.004286	0.05558	1	1044	tags=24%, list=10%, signal=27%
GO_REGULATION_OF_VOLTAGE_GATED_CALCIUM_CHANNEL_ACTIVITY	GO_REGULATION_OF_VOLTAGE_GATED_CALCIUM_CHANNEL_ACTIVITY	21	-0.62341905	-1.6155974	0.010234	0.057	1	1927	tags=43%, list=19%, signal=53%
GO_CELLULAR_RESPIRATION	GO_CELLULAR_RESPIRATION	111	-0.4586585	-1.6147743	0.001235	0.05721	1	4398	tags=66%, list=42%, signal=113%
GO_ERROR_PRONE_TRANSLESION_SYNTHESIS	GO_ERROR_PRONE_TRANSLESION_SYNTHESIS	16	-0.652694	-1.6129389	0.017804	0.05809	1	2068	tags=56%, list=20%, signal=70%
GO_CELLULAR_RESPONSE_TO_DNA_DAMAGE_STIMULUS	GO_CELLULAR_RESPONSE_TO_DNA_DAMAGE_STIMULUS	481	-0.40988	-1.61154	0	0.05872	1	3583	tags=54%, list=35%, signal=78%

GO_MAINTENANCE_OF_CELL_NUMBER	GO_MAINTENANCE_OF_CELL_NUMBER	93	-0.47294602	-1.6103203	0.002424	0.05921	1	1665	tags=30%, list=16%, signal=36%
GO_ADULT_WALKING_BEHAVIOR	GO_ADULT_WALKING_BEHAVIOR	27	-0.5808315	-1.6089625	0.012483	0.05979	1	1311	tags=33%, list=13%, signal=38%
GO_SUBPALLIUM_DEVELOPMENT	GO_SUBPALLIUM_DEVELOPMENT	17	-0.6435706	-1.6088872	0.003035	0.05947	1	1361	tags=41%, list=13%, signal=47%
GO_INTERMEDIATE_FILAMENT_BASED_PROCESS	GO_INTERMEDIATE_FILAMENT_BASED_PROCESS	30	-0.5702822	-1.608363	0.011111	0.05947	1	272	tags=10%, list=3%, signal=10%
GO_RIBONUCLEOPROTEIN_COMPLEX_BIOGENESIS	GO_RIBONUCLEOPROTEIN_COMPLEX_BIOGENESIS	244	-0.4248997	-1.6050134	0	0.06151	1	4142	tags=55%, list=40%, signal=89%
GO_POSITIVE_REGULATION_OF_NEURON_APOPTOTIC_PROCESS	GO_POSITIVE_REGULATION_OF_NEURON_APOPTOTIC_PROCESS	39	-0.5498509	-1.603822	0.004144	0.06205	1	1976	tags=36%, list=19%, signal=44%
GO_DEOXYRIBOSE_PHOSPHATE_CATABOLIC_PROCESS	GO_DEOXYRIBOSE_PHOSPHATE_CATABOLIC_PROCESS	16	-0.6584142	-1.6029452	0.007246	0.06229	1	2424	tags=69%, list=23%, signal=90%
GO_NEURON_NEURON_SYNAPTIC_TRANSMISSION	GO_NEURON_NEURON_SYNAPTIC_TRANSMISSION	51	-0.516402	-1.6028851	0.005312	0.06197	1	1613	tags=25%, list=16%, signal=30%
GO_ANTEROGRADE_AXONAL_TRANSPORT	GO_ANTEROGRADE_AXONAL_TRANSPORT	15	-0.66754496	-1.602763	0.008982	0.0617	1	320	tags=27%, list=3%, signal=27%
GO_INTERSTRAND_CROSS_LINK_REPAIR	GO_INTERSTRAND_CROSS_LINK_REPAIR	22	-0.603628	-1.6027247	0.017956	0.06137	1	2807	tags=55%, list=27%, signal=75%
GO_DENDRITE_DEVELOPMENT	GO_DENDRITE_DEVELOPMENT	63	-0.5005726	-1.6014869	0.005168	0.06188	1	2129	tags=35%, list=21%, signal=44%
GO_NEGATIVE_REGULATION_OF_CELL_DIVISION	GO_NEGATIVE_REGULATION_OF_CELL_DIVISION	41	-0.53656465	-1.601252	0.006831	0.06168	1	2332	tags=54%, list=22%, signal=69%
GO_SOMITOGENESIS	GO_SOMITOGENESIS	45	-0.53010625	-1.6010273	0.003958	0.06152	1	1471	tags=31%, list=14%, signal=36%
GO_HISTONE_H3_K4_METHYLATION	GO_HISTONE_H3_K4_METHYLATION	18	-0.6381033	-1.6007184	0.016058	0.06139	1	2641	tags=67%, list=25%, signal=89%
GO_REGULATION_OF_DENDRITIC_SPINE_DEVELOPMENT	GO_REGULATION_OF_DENDRITIC_SPINE_DEVELOPMENT	38	-0.5494153	-1.6003993	0.010855	0.06124	1	2451	tags=50%, list=24%, signal=65%

GO_SPLICEOSOMAL_COMPLEX_ASSEMBLY	GO_SPLICEOSOMAL_COMPLEX_ASSEMBLY	32	-0.56712765	-1.5985243	0.007032	0.06225	1	4060	tags=84%, list=39%, signal=138%
GO_REGULATION_OF_AMINE_TRANSPORT	GO_REGULATION_OF_AMINE_TRANSPORT	67	-0.48560953	-1.597733	0.005168	0.06241	1	797	tags=16%, list=8%, signal=18%
GO_AXIS_SPECIFICATION	GO_AXIS_SPECIFICATION	65	-0.49334514	-1.5965761	0.003778	0.06294	1	1264	tags=28%, list=12%, signal=31%
GO_LUNG_CELL_DIFFERENTIATION	GO_LUNG_CELL_DIFFERENTIATION	19	-0.6236855	-1.5962033	0.012839	0.0628	1	1038	tags=26%, list=10%, signal=29%
GO_CEREBRAL_CORTEX_DEVELOPMENT	GO_CEREBRAL_CORTEX_DEVELOPMENT	82	-0.47643447	-1.5928777	0.005083	0.06479	1	1612	tags=35%, list=16%, signal=42%
GO_NEGATIVE_REGULATION_OF_CYTOSKELETON_ORGANIZATION	GO_NEGATIVE_REGULATION_OF_CYTOSKELETON_ORGANIZATION	156	-0.4417434	-1.5913161	0.001163	0.06557	1	1681	tags=31%, list=16%, signal=36%
GO_NEGATIVE_REGULATION_OF_MITOTIC_NUCLEAR_DIVISION	GO_NEGATIVE_REGULATION_OF_MITOTIC_NUCLEAR_DIVISION	25	-0.58861643	-1.5908382	0.008633	0.06554	1	3382	tags=68%, list=33%, signal=101%
GO_MRNA_METABOLIC_PROCESS	GO_MRNA_METABOLIC_PROCESS	340	-0.41631258	-1.5892729	0	0.06622	1	4206	tags=56%, list=41%, signal=92%
GO_NUCLEAR_TRANSCRIBED_MRNA_CATABOLIC_PROCESS_EXONUCLEOLYTIC	GO_NUCLEAR_TRANSCRIBED_MRNA_CATABOLIC_PROCESS_EXONUCLEOLYTIC	23	-0.60231286	-1.5846121	0.022378	0.06934	1	3399	tags=78%, list=33%, signal=116%
GO_PHOTORECEPTOR_CELL_DEVELOPMENT	GO_PHOTORECEPTOR_CELL_DEVELOPMENT	28	-0.57265437	-1.5824138	0.015299	0.0708	1	1553	tags=29%, list=15%, signal=34%
GO_MITOTIC_CELL_CYCLE_CHECKPOINT	GO_MITOTIC_CELL_CYCLE_CHECKPOINT	110	-0.4562341	-1.5820655	0.003628	0.07066	1	3599	tags=55%, list=35%, signal=84%
GO_SPERMATID_DIFFERENTIATION	GO_SPERMATID_DIFFERENTIATION	69	-0.4894847	-1.581853	0.005	0.07044	1	2584	tags=33%, list=25%, signal=44%
GO_TRANSMISSION_OF_NERVE_IMPULSE	GO_TRANSMISSION_OF_NERVE_IMPULSE	44	-0.52525246	-1.5816356	0.007979	0.07025	1	345	tags=11%, list=3%, signal=12%
GO_PROTEIN_UBIQUITINATION	GO_PROTEIN_UBIQUITINATION	365	-0.41308215	-1.5803006	0	0.07089	1	3785	tags=51%, list=36%, signal=77%
GO_ATP_DEPENDENT_CHROMATIN_REMODELING	GO_ATP_DEPENDENT_CHROMATIN_REMODELING	43	-0.5234484	-1.5802718	0.009396	0.07055	1	2861	tags=49%, list=28%, signal=67%

GO_INTRACILIARY_TRANSPORT	GO_INTRACILIARY_TRANSPORT	17	-0.6317007	-1.5802215	0.01506	0.07022	1	1934	tags=53%, list=19%, signal=65%
GO_NEGATIVE_REGULATION_OF_PROTEIN_COMPLEX_DISASSEMBLY	GO_NEGATIVE_REGULATION_OF_PROTEIN_COMPLEX_DISASSEMBLY	121	-0.4507171	-1.5802085	0.001163	0.06988	1	1681	tags=31%, list=16%, signal=37%
GO_DNA_DAMAGE_RESPONSE_DETECTION_OF_DNA_DAMAGE	GO_DNA_DAMAGE_RESPONSE_DETECTION_OF_DNA_DAMAGE	33	-0.5493929	-1.5797125	0.008219	0.06988	1	3153	tags=61%, list=30%, signal=87%
GO_SPLICEOSOMAL_SNRNP_ASSEMBLY	GO_SPLICEOSOMAL_SNRNP_ASSEMBLY	27	-0.566563	-1.5796468	0.017021	0.06954	1	3922	tags=78%, list=38%, signal=125%
GO_TRNA_MODIFICATION	GO_TRNA_MODIFICATION	26	-0.57603407	-1.5790755	0.018258	0.06964	1	2316	tags=50%, list=22%, signal=64%
GO_POTASSIUM_ION_TRANSPORT	GO_POTASSIUM_ION_TRANSPORT	111	-0.45847195	-1.578774	0.003727	0.06951	1	425	tags=11%, list=4%, signal=11%
GO_PROTEIN_K63_LINKED_UBIQUITINATION	GO_PROTEIN_K63_LINKED_UBIQUITINATION	25	-0.5695201	-1.5778575	0.028329	0.0699	1	3922	tags=76%, list=38%, signal=122%
GO_PALLIUM_DEVELOPMENT	GO_PALLIUM_DEVELOPMENT	117	-0.45124885	-1.5775812	0	0.06974	1	1513	tags=27%, list=15%, signal=32%
GO_RESPONSE_TO_LIGHT_STIMULUS	GO_RESPONSE_TO_LIGHT_STIMULUS	215	-0.4279161	-1.5772958	0	0.06959	1	2464	tags=34%, list=24%, signal=44%
GO_NEGATIVE_REGULATION_OF_CHROMOSOME_SEGREGATION	GO_NEGATIVE_REGULATION_OF_CHROMOSOME_SEGREGATION	23	-0.59061635	-1.5757673	0.025899	0.07045	1	2332	tags=52%, list=22%, signal=67%
GO_DORSAL_VENTRAL_PATTERN_FORMATION	GO_DORSAL_VENTRAL_PATTERN_FORMATION	63	-0.49281394	-1.574515	0.008997	0.07098	1	1903	tags=30%, list=18%, signal=37%
GO_POSITIVE_REGULATION_OF_DNA_REPAIR	GO_POSITIVE_REGULATION_OF_DNA_REPAIR	27	-0.5723477	-1.5743278	0.019774	0.07076	1	2299	tags=52%, list=22%, signal=66%
GO_ORGANELLE_ASSEMBLY	GO_ORGANELLE_ASSEMBLY	287	-0.4120791	-1.57421	0	0.0705	1	2256	tags=32%, list=22%, signal=40%
GO_WALKING_BEHAVIOR	GO_WALKING_BEHAVIOR	27	-0.5808315	-1.5740579	0.015342	0.07028	1	1311	tags=33%, list=13%, signal=38%
GO_PEPTIDYL_LYSINE_TRIMETHYLATION	GO_PEPTIDYL_LYSINE_TRIMETHYLATION	15	-0.65559405	-1.5736382	0.017964	0.07024	1	2347	tags=60%, list=23%, signal=77%

GO_TRANSLATIONAL_TERMINATION	GO_TRANSLATIONAL_TERMINATION	55	-0.49791154	-1.5732192	0.005222	0.07024	1	3916	tags=65%, list=38%, signal=105%
GO_REGULATION_OF_DENDRITE_DEVELOPMENT	GO_REGULATION_OF_DENDRITE_DEVELOPMENT	88	-0.4674597	-1.572358	0.001245	0.07049	1	2298	tags=38%, list=22%, signal=48%
GO_NEUROBLAST_PROLIFERATION	GO_NEUROBLAST_PROLIFERATION	21	-0.6109028	-1.5723361	0.010014	0.07017	1	1473	tags=33%, list=14%, signal=39%
GO_POSITIVE_REGULATION_OF_HISTONE_METHYLATION	GO_POSITIVE_REGULATION_OF_HISTONE_METHYLATION	25	-0.576031	-1.5706892	0.011905	0.07106	1	1875	tags=44%, list=18%, signal=54%
GO_AXO_DENDRITIC_TRANSPORT	GO_AXO_DENDRITIC_TRANSPORT	23	-0.58078307	-1.5706778	0.02329	0.07074	1	593	tags=22%, list=6%, signal=23%
GO_NEGATIVE_REGULATION_OF_CHROMOSOME_ORGANIZATION	GO_NEGATIVE_REGULATION_OF_CHROMOSOME_ORGANIZATION	72	-0.47826144	-1.5704683	0.004926	0.07061	1	2332	tags=42%, list=22%, signal=53%
GO_NEGATIVE_REGULATION_OF_GLIAL_CELL_DIFFERENTIATION	GO_NEGATIVE_REGULATION_OF_GLIAL_CELL_DIFFERENTIATION	21	-0.59561026	-1.5695215	0.021307	0.07104	1	2200	tags=48%, list=21%, signal=60%
GO_POSITIVE_REGULATION_OF_NEUROBLAST_PROLIFERATION	GO_POSITIVE_REGULATION_OF_NEUROBLAST_PROLIFERATION	18	-0.62004447	-1.5693625	0.021116	0.07085	1	2223	tags=50%, list=21%, signal=64%
GO_REGULATION_OF_APPETITE	GO_REGULATION_OF_APPETITE	18	-0.62225425	-1.5666313	0.019971	0.07263	1	270	tags=17%, list=3%, signal=17%
GO_SPINDLE_ASSEMBLY	GO_SPINDLE_ASSEMBLY	45	-0.5149316	-1.5661942	0.009434	0.07268	1	1009	tags=33%, list=10%, signal=37%
GO_REGULATION_OF_DNA_REPAIR	GO_REGULATION_OF_DNA_REPAIR	52	-0.5047503	-1.5655161	0.011811	0.07298	1	2400	tags=50%, list=23%, signal=65%
GO_PROTEIN_TRANSPORT_ALONG_MICROTUBULE	GO_PROTEIN_TRANSPORT_ALONG_MICROTUBULE	17	-0.63170075	-1.5643785	0.012748	0.07351	1	1934	tags=53%, list=19%, signal=65%
GO_NEURAL_NUCLEUS_DEVELOPMENT	GO_NEURAL_NUCLEUS_DEVELOPMENT	52	-0.49627176	-1.5629122	0.009321	0.07441	1	2059	tags=37%, list=20%, signal=45%
GO_METHYLATION	GO_METHYLATION	145	-0.43528607	-1.5625511	0.001172	0.07436	1	3405	tags=48%, list=33%, signal=71%

GO_CELLULAR_COMPONENT_ASSEMBLY_INVOLVED_IN_MORPHOGENESIS	GO_CELLULAR_COMPONENT_ASSEMBLY_INVOLVED_IN_MORPHOGENESIS	140	-0.43243083	-1.5610461	0	0.07527	1	2254	tags=33%, list=22%, signal=41%
GO_ORGANELLE_TRANSPORT_ALONG_MICROTUBULE	GO_ORGANELLE_TRANSPORT_ALONG_MICROTUBULE	38	-0.5367758	-1.560902	0.011236	0.07504	1	2359	tags=42%, list=23%, signal=54%
GO_POSITIVE_REGULATION_OF_EXCITATORY_POSTSYNAPTIC_POTENTIAL	GO_POSITIVE_REGULATION_OF_EXCITATORY_POSTSYNAPTIC_POTENTIAL	15	-0.63864005	-1.5607611	0.018182	0.07481	1	1767	tags=40%, list=17%, signal=48%
GO_COCHLEA_DEVELOPMENT	GO_COCHLEA_DEVELOPMENT	30	-0.5551764	-1.5600777	0.021097	0.07501	1	3530	tags=60%, list=34%, signal=91%
GO_ACID_SECRETION	GO_ACID_SECRETION	54	-0.49363837	-1.5598079	0.010485	0.07488	1	1444	tags=28%, list=14%, signal=32%
GO_REGULATION_OF_DENDRITE_MORPHOGENESIS	GO_REGULATION_OF_DENDRITE_MORPHOGENESIS	57	-0.49144426	-1.557404	0.006631	0.07655	1	1618	tags=28%, list=16%, signal=33%
GO_TRANSLESION_SYNTHESIS	GO_TRANSLESION_SYNTHESIS	32	-0.5446537	-1.5545963	0.023843	0.0785	1	2120	tags=44%, list=20%, signal=55%
GO_MICROTUBULE_CYTOSKELETON_ORGANIZATION_INVOLVED_IN_MITOSIS	GO_MICROTUBULE_CYTOSKELETON_ORGANIZATION_INVOLVED_IN_MITOSIS	30	-0.55422217	-1.5543216	0.017167	0.07839	1	1009	tags=40%, list=10%, signal=44%
GO_HISTONE_H3_DEACETYLATION	GO_HISTONE_H3_DEACETYLATION	17	-0.6216845	-1.5543175	0.024781	0.07806	1	1915	tags=47%, list=18%, signal=58%
GO_OXIDATIVE_PHOSPHORYLATION	GO_OXIDATIVE_PHOSPHORYLATION	62	-0.4849639	-1.5529023	0.013977	0.07896	1	4160	tags=69%, list=40%, signal=115%
GO_MITOTIC_SPINDLE_ASSEMBLY	GO_MITOTIC_SPINDLE_ASSEMBLY	30	-0.55422217	-1.5523162	0.021157	0.07921	1	1009	tags=40%, list=10%, signal=44%
GO_SINGLE_ORGANISM_BEHAVIOR	GO_SINGLE_ORGANISM_BEHAVIOR	305	-0.4044665	-1.5521257	0	0.07904	1	1311	tags=22%, list=13%, signal=24%
GO_REGULATION_OF_TRANSCRIPTION_INVOLVED_IN_G1_S_TRANSITION_OF_MITOTIC_CELL_CYCLE	GO_REGULATION_OF_TRANSCRIPTION_INVOLVED_IN_G1_S_TRANSITION_OF_MITOTIC_CELL_CYCLE	19	-0.6085179	-1.5506685	0.015805	0.07988	1	2612	tags=58%, list=25%, signal=77%
GO_ACTION_POTENTIAL	GO_ACTION_POTENTIAL	80	-0.47255304	-1.5498375	0.001235	0.08018	1	2427	tags=28%, list=23%, signal=36%

GO_SYNAPSE_ASSEMBLY	GO_SYNAPSE_ASSEMBLY	48	-0.51121795	-1.5475407	0.006748	0.08182	1	1603	tags=31%, list=15%, signal=37%
GO_GLUTAMATE_RECEPTOR_SIGNALING_PATHWAY	GO_GLUTAMATE_RECEPTOR_SIGNALING_PATHWAY	36	-0.53281945	-1.5452992	0.013908	0.08339	1	1422	tags=25%, list=14%, signal=29%
GO_METENCEPHALON_DEVELOPMENT	GO_METENCEPHALON_DEVELOPMENT	80	-0.46003702	-1.541762	0.003802	0.08603	1	847	tags=19%, list=8%, signal=20%
GO_NEGATIVE_REGULATION_OF_CELL_CYCLE_PROCESS	GO_NEGATIVE_REGULATION_OF_CELL_CYCLE_PROCESS	158	-0.42837757	-1.5409067	0	0.08643	1	3574	tags=53%, list=34%, signal=80%
GO_PROTEIN_ACETYLATION	GO_PROTEIN_ACETYLATION	67	-0.48178402	-1.5400099	0.007712	0.08684	1	3610	tags=61%, list=35%, signal=93%
GO_MACROMOLECULAR_COMPLEX_DISASSEMBLY	GO_MACROMOLECULAR_COMPLEX_DISASSEMBLY	121	-0.4338417	-1.5381767	0.002375	0.08801	1	3003	tags=47%, list=29%, signal=66%
GO_MRNA_3_END_PROCESSING	GO_MRNA_3_END_PROCESSING	40	-0.52320206	-1.5378832	0.012195	0.08789	1	3863	tags=70%, list=37%, signal=111%
GO_RNA_3_END_PROCESSING	GO_RNA_3_END_PROCESSING	53	-0.48893884	-1.5364592	0.009186	0.08879	1	3642	tags=66%, list=35%, signal=101%
GO_SOMITE_DEVELOPMENT	GO_SOMITE_DEVELOPMENT	57	-0.4908884	-1.5357207	0.008895	0.08901	1	1471	tags=28%, list=14%, signal=33%
GO_ELECTRON_TRANSPORT_CHAIN	GO_ELECTRON_TRANSPORT_CHAIN	71	-0.47240165	-1.5352015	0.001285	0.08917	1	4398	tags=66%, list=42%, signal=114%
GO_MULTICELLULAR_ORGANISM_AGING	GO_MULTICELLULAR_ORGANISM_AGING	21	-0.5853353	-1.5311694	0.03003	0.09241	1	2850	tags=48%, list=27%, signal=65%
GO_CELL_FATE_SPECIFICATION	GO_CELL_FATE_SPECIFICATION	50	-0.49874878	-1.5311673	0.014845	0.09204	1	1859	tags=28%, list=18%, signal=34%
GO_PROTEIN_MONOUBIQUITINATION	GO_PROTEIN_MONOUBIQUITINATION	39	-0.5154649	-1.5305051	0.020134	0.09229	1	3332	tags=51%, list=32%, signal=75%
GO_RNA_MODIFICATION	GO_RNA_MODIFICATION	50	-0.5010832	-1.5286969	0.011811	0.09357	1	3431	tags=50%, list=33%, signal=74%
GO_CHROMOSOME_LOCALIZATION	GO_CHROMOSOME_LOCALIZATION	41	-0.508794	-1.5271643	0.014379	0.09448	1	3445	tags=63%, list=33%, signal=95%
GO_CELL_PART_MORPHOGENESIS	GO_CELL_PART_MORPHOGENESIS	400	-0.39492986	-1.527067	0	0.09419	1	2156	tags=30%, list=21%, signal=37%

GO_REGULATION_OF_CENTROSOME_CYCLE	GO_REGULATION_OF_CENTROSOME_CYCLE	28	-0.55408096	-1.5261506	0.035014	0.09466	1	3554	tags=75%, list=34%, signal=114%
GO_TRANSCRIPTION_ELONGATION_FROM_RNA_POLYMERASE_II_PROMOTER	GO_TRANSCRIPTION_ELONGATION_FROM_RNA_POLYMERASE_II_PROMOTER	67	-0.46356562	-1.525826	0.013995	0.09458	1	3335	tags=57%, list=32%, signal=83%
GO_RETINA_LAYER_FORMATION	GO_RETINA_LAYER_FORMATION	16	-0.6193618	-1.5256975	0.039941	0.09432	1	115	tags=19%, list=1%, signal=19%
GO_PROTEIN_AUTOUBIQUITINATION	GO_PROTEIN_AUTOUBIQUITINATION	32	-0.5363118	-1.5250055	0.026685	0.09454	1	3696	tags=63%, list=36%, signal=97%
GO_RETROGRADE_VESICLE_MEDIATED_TRANSPORT_GOLGI_TO_ER	GO_RETROGRADE_VESICLE_MEDIATED_TRANSPORT_GOLGI_TO_ER	59	-0.48107588	-1.5245761	0.017038	0.09459	1	2943	tags=49%, list=28%, signal=68%
GO_SOMATIC_STEM_CELL_POPULATION_MAINTENANCE	GO_SOMATIC_STEM_CELL_POPULATION_MAINTENANCE	53	-0.48966947	-1.5235417	0.014628	0.09523	1	1665	tags=28%, list=16%, signal=34%
GO_REGULATION_OF_DNA_TEMPLATED_TRANSCRIPTION_ELONGATION	GO_REGULATION_OF_DNA_TEMPLATED_TRANSCRIPTION_ELONGATION	32	-0.53675145	-1.5212253	0.020604	0.09719	1	3245	tags=56%, list=31%, signal=82%
GO_NUCLEOTIDE_EXCISION_REPAIR_DNA_INCISION	GO_NUCLEOTIDE_EXCISION_REPAIR_DNA_INCISION	35	-0.5223326	-1.5205051	0.020243	0.09748	1	3762	tags=66%, list=36%, signal=103%
GO_TELOMERE_CAPPING	GO_TELOMERE_CAPPING	19	-0.5927795	-1.5182794	0.019915	0.09939	1	3089	tags=58%, list=30%, signal=82%
GO_PROTEIN_ACYLATION	GO_PROTEIN_ACYLATION	83	-0.44813782	-1.5181379	0.002516	0.09913	1	3610	tags=57%, list=35%, signal=86%
GO_COGNITION	GO_COGNITION	199	-0.4095646	-1.5177865	0	0.09912	1	1513	tags=25%, list=15%, signal=28%
GO_MACROMOLECULE_METHYLATION	GO_MACROMOLECULE_METHYLATION	109	-0.4368389	-1.5174454	0.004884	0.09914	1	3376	tags=46%, list=33%, signal=67%
GO_PROTEIN_UBIQUITINATION_INVOLVED_IN_UBIQUITIN_DEPENDENT_PROTEIN_CATABOLIC_PROCESS	GO_PROTEIN_UBIQUITINATION_INVOLVED_IN_UBIQUITIN_DEPENDENT_PROTEIN_CATABOLIC_PROCESS	89	-0.44668183	-1.5172479	0.007417	0.09894	1	4322	tags=63%, list=42%, signal=107%

GO_CYTOKINESIS	GO_CYTOKINESIS	58	-0.47847846	-1.5171226	0.019763	0.09866	1	2829	tags=47%, list=27%, signal=64%
GO_MACROMOLECULE_DEACYLATION	GO_MACROMOLECULE_DEACYLATION	48	-0.49462318	-1.5152595	0.017568	0.09997	1	3763	tags=52%, list=36%, signal=81%

Enriched pathways in GO cellular component for CTSI high risk group

NAME	GS<br>follow link to MSigDB	SIZE	ES	NES	NOM p-val	FDR q-val	FWER p-val	RANK AT MAX	LEADING EDGE
GO_EXTRACELLULAR_MATRIX_COMPONENT	GO_EXTRACELLULAR_MATRIX_COMPONENT	99	0.707726	2.881564	0	0	0	742	tags=39%, list=7%, signal=42%
GO_BASEMENT_MEMBRANE	GO_BASEMENT_MEMBRANE	72	0.70496	2.699865	0	0	0	742	tags=40%, list=7%, signal=43%
GO_PHAGOCYTIC_VESICLE	GO_PHAGOCYTIC_VESICLE	61	0.697483	2.602737	0	0	0	1203	tags=48%, list=12%, signal=53%
GO_PLATELET_ALPHA_GRANULE_LUMEN	GO_PLATELET_ALPHA_GRANULE_LUMEN	46	0.716614	2.587456	0	0	0	1176	tags=39%, list=11%, signal=44%
GO_COLLAGEN_TRIMER	GO_COLLAGEN_TRIMER	58	0.688238	2.542815	0	0	0	997	tags=45%, list=10%, signal=49%
GO_EXTRACELLULAR_MATRIX	GO_EXTRACELLULAR_MATRIX	305	0.548234	2.5296	0	0	0	1487	tags=36%, list=14%, signal=41%
GO_ENDOPLASMIC_RETICULUM_LUMEN	GO_ENDOPLASMIC_RETICULUM_LUMEN	141	0.584284	2.52635	0	0	0	1516	tags=40%, list=15%, signal=46%
GO_PROTEINACEOUS_EXTRACELLULAR_MATRIX	GO_PROTEINACEOUS_EXTRACELLULAR_MATRIX	252	0.546634	2.525686	0	0	0	1351	tags=35%, list=13%, signal=39%
GO_PLATELET_ALPHA_GRANULE	GO_PLATELET_ALPHA_GRANULE	62	0.675832	2.506731	0	0	0	1176	tags=37%, list=11%, signal=42%
GO_COMPLEX_OF_COLLAGEN_TRIMERS	GO_COMPLEX_OF_COLLAGEN_TRIMERS	22	0.778333	2.371068	0	0	0	997	tags=55%, list=10%, signal=60%

GO_MHC_PROTEIN_COMPLEX	GO_MHC_PROTEIN_COMPLEX	19	0.805836	2.319073	0	0	0	1963	tags=89%, list=19%, signal=110%
GO_BLOOD_MICROPARTICLE	GO_BLOOD_MICROPARTICLE	87	0.572547	2.299567	0	0.000102	0.001	1187	tags=25%, list=11%, signal=28%
GO_EXTERNAL_SIDE_OF_PLASMA_MEMBRANE	GO_EXTERNAL_SIDE_OF_PLASMA_MEMBRANE	171	0.519633	2.295062	0	9.38E-05	0.001	1508	tags=36%, list=15%, signal=42%
GO_PROTEIN_COMPLEX_INVOLVED_IN_CELL_ADHESION	GO_PROTEIN_COMPLEX_INVOLVED_IN_CELL_ADHESION	25	0.719364	2.277527	0	8.71E-05	0.001	986	tags=48%, list=9%, signal=53%
GO_PHAGOCYTIC_VESICLE_MEMBRANE	GO_PHAGOCYTIC_VESICLE_MEMBRANE	39	0.651351	2.241917	0	0.000187	0.002	1203	tags=46%, list=12%, signal=52%
GO_VACUOLAR_LUMEN	GO_VACUOLAR_LUMEN	87	0.550819	2.217039	0	0.000175	0.002	1161	tags=45%, list=11%, signal=50%
GO_LYTIC_VACUOLE	GO_LYTIC_VACUOLE	355	0.462718	2.199801	0	0.00033	0.004	1182	tags=31%, list=11%, signal=34%
GO_LYSOSOMAL_LUMEN	GO_LYSOSOMAL_LUMEN	68	0.584545	2.199422	0	0.000312	0.004	1161	tags=51%, list=11%, signal=58%
GO_VESICLE_LUMEN	GO_VESICLE_LUMEN	86	0.540315	2.159337	0	0.000373	0.005	1176	tags=28%, list=11%, signal=31%
GO_SECRETORY_GRANULE_LUMEN	GO_SECRETORY_GRANULE_LUMEN	70	0.571696	2.156179	0	0.000354	0.005	1176	tags=31%, list=11%, signal=35%
GO_CELL_SUBSTRATE_JUNCTION	GO_CELL_SUBSTRATE_JUNCTION	311	0.458082	2.119395	0	0.000686	0.01	1509	tags=33%, list=15%, signal=37%
GO_PODOSOME	GO_PODOSOME	16	0.753465	2.084873	0	0.000847	0.013	1443	tags=63%, list=14%, signal=72%

GO_ANCHORING_JUNCTION	GO_ANCHORING_JUNCTION	371	0.430987	2.044441	0	0.001397	0.022	1509	tags=31%, list=15%, signal=35%
GO_BASAL_LAMINA	GO_BASAL_LAMINA	17	0.721166	2.033004	0	0.001462	0.024	1943	tags=65%, list=19%, signal=79%
GO_LYTIC_VACUOLE_MEMBRANE	GO_LYTIC_VACUOLE_MEMBRANE	170	0.458162	1.995645	0	0.002153	0.037	1934	tags=40%, list=19%, signal=48%
GO_MEMBRANE_MICRODOMAIN	GO_MEMBRANE_MICRODOMAIN	239	0.421524	1.943997	0	0.003446	0.061	1029	tags=26%, list=10%, signal=28%
GO_ENDOCYTIC_VESICLE_MEMBRANE	GO_ENDOCYTIC_VESICLE_MEMBRANE	110	0.454495	1.909562	0	0.004379	0.079	1203	tags=35%, list=12%, signal=39%
GO_ENDOCYTIC_VESICLE	GO_ENDOCYTIC_VESICLE	189	0.426055	1.906363	0	0.004273	0.08	1321	tags=32%, list=13%, signal=36%
GO_PIGMENT_GRANULE	GO_PIGMENT_GRANULE	93	0.476157	1.890173	0	0.005196	0.1	1190	tags=37%, list=11%, signal=41%
GO_IMMUNOLOGICAL_SYNAPSE	GO_IMMUNOLOGICAL_SYNAPSE	26	0.613672	1.884398	0.003484	0.005392	0.106	1766	tags=54%, list=17%, signal=65%
GO_ACTIN_FILAMENT	GO_ACTIN_FILAMENT	51	0.504731	1.829656	0	0.008309	0.161	1525	tags=45%, list=15%, signal=53%
GO_SIDE_OF_MEMBRANE	GO_SIDE_OF_MEMBRANE	320	0.389404	1.820798	0	0.008651	0.17	1508	tags=29%, list=15%, signal=33%
GO_LUMENAL_SIDE_OF_MEMBRANE	GO_LUMENAL_SIDE_OF_MEMBRANE	19	0.627354	1.796747	0	0.010142	0.204	2160	tags=74%, list=21%, signal=93%
GO_VACUOLAR_PART	GO_VACUOLAR_PART	450	0.364855	1.775407	0	0.011936	0.242	1532	tags=29%, list=15%, signal=32%

GO_ENDOSOME_LUMEN	GO_ENDOSOME_LUMEN	20	0.601301	1.774339	0.006369	0.01178	0.246	1123	tags=35%, list=11%, signal=39%
GO_PLASMA_MEMBRANE_RECEPTOR_COMPLEX	GO_PLASMA_MEMBRANE_RECEPTOR_COMPLEX	138	0.403196	1.77146	0	0.011589	0.249	1342	tags=30%, list=13%, signal=34%
GO_ACTIN_FILAMENT_BUNDLE	GO_ACTIN_FILAMENT_BUNDLE	42	0.506875	1.760529	0	0.012692	0.273	1566	tags=40%, list=15%, signal=47%
GO_SECRETORY_GRANULE	GO_SECRETORY_GRANULE	252	0.391594	1.734298	0	0.015404	0.325	1241	tags=25%, list=12%, signal=28%
GO_ER_TO_GOLGI_TRANSPORT_VESICLE	GO_ER_TO_GOLGI_TRANSPORT_VESICLE	52	0.46765	1.727821	0.004587	0.016227	0.345	1963	tags=50%, list=19%, signal=61%
GO_T_CELL_RECEPTOR_COMPLEX	GO_T_CELL_RECEPTOR_COMPLEX	17	0.614989	1.718646	0.009317	0.017238	0.369	2255	tags=53%, list=22%, signal=68%
GO_ANCHORED_COMPONENT_OF_PLASMA_MEMBRANE	GO_ANCHORED_COMPONENT_OF_PLASMA_MEMBRANE	22	0.55619	1.644549	0.021672	0.029596	0.559	758	tags=27%, list=7%, signal=29%
GO_ROUGH_ENDOPLASMIC_RETICULUM_MEMBRANE	GO_ROUGH_ENDOPLASMIC_RETICULUM_MEMBRANE	15	0.576389	1.58783	0.025157	0.044183	0.714	428	tags=20%, list=4%, signal=21%
GO_RUFFLE_MEMBRANE	GO_RUFFLE_MEMBRANE	59	0.426473	1.583652	0.008734	0.044559	0.726	1315	tags=29%, list=13%, signal=33%
GO_ENDOPLASMIC_RETICULUM_GOLGI_INTERMEDIATE_COMPARTMENT	GO_ENDOPLASMIC_RETICULUM_GOLGI_INTERMEDIATE_COMPARTMENT	71	0.410873	1.577179	0.00495	0.045733	0.741	1462	tags=37%, list=14%, signal=42%
GO_RUFFLE	GO_RUFFLE	110	0.384242	1.574126	0	0.045889	0.757	1315	tags=29%, list=13%, signal=33%

GO_ENDOPLASMIC_RETICULUM_GOLGI_INTERMEDIATE_COMPARTMENT_MEMBRANE	GO_ENDOPLASMIC_RETICULUM_GOLGI_INTERMEDIATE_COMPARTMENT_MEMBRANE	43	0.449536	1.5534	0.020492	0.051674	0.791	1462	tags=35%, list=14%, signal=40%
GO_CELL_CELL_ADHERENS_JUNCTION	GO_CELL_CELL_ADHERENS_JUNCTION	43	0.444615	1.533308	0.022814	0.058172	0.838	1452	tags=30%, list=14%, signal=35%
GO_ENDOSOMAL_PART	GO_ENDOSOMAL_PART	281	0.323223	1.522072	0	0.061362	0.854	1516	tags=26%, list=15%, signal=29%
GO_KERATIN_FILAMENT	GO_KERATIN_FILAMENT	18	0.536418	1.518777	0.036923	0.061311	0.861	3465	tags=83%, list=33%, signal=125%
GO_VACUOLAR_MEMBRANE	GO_VACUOLAR_MEMBRANE	372	0.315928	1.512695	0	0.06266	0.874	1532	tags=25%, list=15%, signal=29%
GO_ACTOMYOSIN	GO_ACTOMYOSIN	47	0.424072	1.504978	0.011905	0.064728	0.889	1566	tags=36%, list=15%, signal=42%
GO_LATE_ENDOSOME	GO_LATE_ENDOSOME	130	0.347869	1.455511	0	0.087063	0.966	1123	tags=22%, list=11%, signal=24%
GO_LIPID_PARTICLE	GO_LIPID_PARTICLE	39	0.421151	1.438654	0.048193	0.094993	0.977	1646	tags=28%, list=16%, signal=33%
GO_PROTEIN_LIPID_COMPLEX	GO_PROTEIN_LIPID_COMPLEX	29	0.44373	1.433725	0.054608	0.096271	0.978	1026	tags=28%, list=10%, signal=31%
GO_RECEPTOR_COMPLEX	GO_RECEPTOR_COMPLEX	256	0.306606	1.427166	0	0.097903	0.983	1342	tags=24%, list=13%, signal=27%
GO_COSTAMERE	GO_COSTAMERE	16	0.52365	1.425354	0.087227	0.097309	0.984	1507	tags=38%, list=15%, signal=44%

Enriched pathways in GO cellular component for CTSI low risk group

NAME	GS<br>follow link to MSigDB	SIZE	ES	NES	NOM p-val	FDR q-val	FWER p-val	RANK AT MAX	LEADING EDGE
GO_PRESYNAPTIC_ACTIVE_ZONE	GO_PRESYNAPTIC_ACTIVE_ZONE	24	-0.777	-2.09009	0	0	0	1283	tags=67%, list=12%, signal=76%
GO_EXOCYTIC_VESICLE_MEMBRANE	GO_EXOCYTIC_VESICLE_MEMBRANE	37	-0.68213	-2.00436	0	0.000864	0.002	2159	tags=65%, list=21%, signal=82%
GO_CONDENSED_CHROMOSOME	GO_CONDENSED_CHROMOSOME	123	-0.56717	-1.99251	0	0.000576	0.002	3455	tags=68%, list=33%, signal=101%
GO_PRESYNAPSE	GO_PRESYNAPSE	209	-0.53077	-1.97142	0	0.000866	0.004	2089	tags=37%, list=20%, signal=45%
GO_CHROMOSOMAL_REGION	GO_CHROMOSOMAL_REGION	204	-0.52875	-1.94672	0	0.001566	0.009	3453	tags=58%, list=33%, signal=86%
GO_MICROTUBULE_ORGANIZING_CENTER_PART	GO_MICROTUBULE_ORGANIZING_CENTER_PART	82	-0.57149	-1.91795	0	0.002019	0.014	2906	tags=59%, list=28%, signal=81%
GO_TERMINAL_BOUTON	GO_TERMINAL_BOUTON	53	-0.61326	-1.91465	0	0.00173	0.014	1909	tags=36%, list=18%, signal=44%
GO_CENTRIOLE	GO_CENTRIOLE	57	-0.59841	-1.91408	0	0.001514	0.014	2829	tags=65%, list=27%, signal=89%
GO_CONDENSED_NUCLEAR_CHROMOSOME	GO_CONDENSED_NUCLEAR_CHROMOSOME	57	-0.60194	-1.91064	0	0.001539	0.016	3453	tags=67%, list=33%, signal=99%

GO_CHROMOSOME_CENTROMERIC_REGION	GO_CHROMOSOME_CENTROMERIC_REGION	106	-0.54614	-1.90057	0	0.001469	0.017	3553	tags=67%, list=34%, signal=101%
GO_SYNAPSE_PART	GO_SYNAPSE_PART	450	-0.48804	-1.89913	0	0.001336	0.017	2089	tags=32%, list=20%, signal=38%
GO_SPINDLE_POLE	GO_SPINDLE_POLE	78	-0.56649	-1.89201	0	0.001294	0.018	3413	tags=69%, list=33%, signal=102%
GO_AXON	GO_AXON	319	-0.4907	-1.8721	0	0.002063	0.031	2477	tags=38%, list=24%, signal=48%
GO_POSTSYNAPSE	GO_POSTSYNAPSE	283	-0.48033	-1.84301	0	0.003075	0.048	1767	tags=27%, list=17%, signal=31%
GO_KINETOCHORE	GO_KINETOCHORE	70	-0.56225	-1.83718	0	0.003102	0.052	3313	tags=66%, list=32%, signal=96%
GO_MICROTUBULE_ASSOCIATED_COMPLEX	GO_MICROTUBULE_ASSOCIATED_COMPLEX	94	-0.53949	-1.82814	0	0.003335	0.059	2422	tags=44%, list=23%, signal=56%
GO_AXON_PART	GO_AXON_PART	170	-0.50193	-1.82179	0	0.003442	0.065	1927	tags=32%, list=19%, signal=39%
GO_CENTROSOME	GO_CENTROSOME	290	-0.47537	-1.82177	0	0.003251	0.065	3260	tags=50%, list=31%, signal=71%
GO_CONDENSED_CHROMOSOME_CENTROMERIC_REGION	GO_CONDENSED_CHROMOSOME_CENTROMERIC_REGION	59	-0.57232	-1.81659	0	0.003531	0.075	2054	tags=51%, list=20%, signal=63%
GO_NUCLEAR_CHROMOSOME	GO_NUCLEAR_CHROMOSOME	350	-0.47072	-1.81635	0	0.003485	0.078	3453	tags=52%, list=33%, signal=76%
GO_UBIQUITIN_LIGASE_COMPLEX	GO_UBIQUITIN_LIGASE_COMPLEX	138	-0.50791	-1.8132	0	0.003485	0.082	3780	tags=60%, list=36%, signal=93%

GO_TRANSFERASE_COMPLEX	GO_TRANSFERASE_COMPLEX	431	-0.46438	-1.80676	0	0.003879	0.095	3640	tags=56%, list=35%, signal=82%
GO_CULLIN_RING_UBIQUITIN_LIGASE_COMPLEX	GO_CULLIN_RING_UBIQUITIN_LIGASE_COMPLEX	73	-0.55184	-1.8054	0	0.003786	0.097	2999	tags=56%, list=29%, signal=78%
GO_MICROTUBULE_ORGANIZING_CENTER	GO_MICROTUBULE_ORGANIZING_CENTER	363	-0.46969	-1.80061	0	0.003773	0.1	3318	tags=51%, list=32%, signal=72%
GO_NUCLEAR_HETEROCHROMATIN	GO_NUCLEAR_HETEROCHROMATIN	19	-0.69221	-1.78774	0.001511	0.004178	0.114	2692	tags=79%, list=26%, signal=106%
GO_SPLICEOSOMAL_COMPLEX	GO_SPLICEOSOMAL_COMPLEX	97	-0.52024	-1.78529	0	0.004147	0.117	3965	tags=64%, list=38%, signal=102%
GO_NEURONAL_CELL_BODY_MEMBRANE	GO_NEURONAL_CELL_BODY_MEMBRANE	15	-0.74118	-1.78093	0.004511	0.004252	0.124	137	tags=27%, list=1%, signal=27%
GO_METHYLTRANSFERASE_COMPLEX	GO_METHYLTRANSFERASE_COMPLEX	65	-0.54543	-1.77541	0	0.004561	0.136	3616	tags=66%, list=35%, signal=101%
GO_CHROMOSOME_TELOMERIC_REGION	GO_CHROMOSOME_TELOMERIC_REGION	100	-0.51246	-1.77068	0	0.004784	0.147	3422	tags=52%, list=33%, signal=77%
GO_REPLICATION_FORK	GO_REPLICATION_FORK	43	-0.5886	-1.76651	0.002646	0.004939	0.158	2161	tags=56%, list=21%, signal=70%
GO_NUCLEOPLASM_PART	GO_NUCLEOPLASM_PART	443	-0.45039	-1.76569	0	0.004893	0.161	3642	tags=53%, list=35%, signal=78%
GO_EXOCYTIC_VESICLE	GO_EXOCYTIC_VESICLE	95	-0.51344	-1.76197	0	0.005142	0.173	2408	tags=42%, list=23%, signal=54%
GO_NEURON_SPINE	GO_NEURON_SPINE	86	-0.52144	-1.76089	0	0.005013	0.173	1024	tags=23%, list=10%, signal=26%

GO_SPINDLE	GO_SPINDLE	182	-0.48085	-1.75595	0	0.005194	0.185	3248	tags=55%, list=31%, signal=79%
GO_SMALL_NUCLEAR_RIBONUCLEOPROTEIN_COMPLEX	GO_SMALL_NUCLEAR_RIBONUCLEOPROTEIN_COMPLEX	43	-0.58433	-1.74547	0.001374	0.005977	0.22	3350	tags=67%, list=32%, signal=99%
GO_NEURONAL_POSTSYNAPTIC_DENSITY	GO_NEURONAL_POSTSYNAPTIC_DENSITY	46	-0.56954	-1.74287	0.001348	0.00609	0.23	2017	tags=43%, list=19%, signal=54%
GO_EXCITATORY_SYNAPSE	GO_EXCITATORY_SYNAPSE	147	-0.48401	-1.73425	0	0.006459	0.246	1944	tags=35%, list=19%, signal=42%
GO_SWI_SNF_SUPERFAMILY_TYPE_COMPLEX	GO_SWI_SNF_SUPERFAMILY_TYPE_COMPLEX	51	-0.55703	-1.72938	0	0.006694	0.261	2784	tags=61%, list=27%, signal=83%
GO_RNA_POLYMERASE_COMPLEX	GO_RNA_POLYMERASE_COMPLEX	83	-0.51899	-1.72768	0	0.006763	0.269	3104	tags=53%, list=30%, signal=75%
GO_CATALYTIC_STEP_2_SPLICEOSOME	GO_CATALYTIC_STEP_2_SPLICEOSOME	50	-0.55791	-1.72146	0.001351	0.007227	0.288	3475	tags=60%, list=33%, signal=90%
GO_NUCLEAR_CHROMOSOME_TELOMERIC_REGION	GO_NUCLEAR_CHROMOSOME_TELOMERIC_REGION	81	-0.51428	-1.72128	0	0.007073	0.289	3422	tags=52%, list=33%, signal=77%
GO_HISTONE_METHYLTRANSFERASE_COMPLEX	GO_HISTONE_METHYLTRANSFERASE_COMPLEX	52	-0.55054	-1.71931	0.003901	0.007171	0.299	2983	tags=56%, list=29%, signal=78%
GO_KINESIN_COMPLEX	GO_KINESIN_COMPLEX	34	-0.60322	-1.71611	0.001404	0.007363	0.311	2852	tags=65%, list=27%, signal=89%
GO_DENDRITE	GO_DENDRITE	329	-0.44483	-1.71564	0	0.007275	0.313	1119	tags=21%, list=11%, signal=22%
GO_SITE_OF_POLARIZED_GROWTH	GO_SITE_OF_POLARIZED_GROWTH	106	-0.4849	-1.68415	0	0.010888	0.44	2089	tags=37%, list=20%, signal=46%

GO_NEURON_PROJECTION_TERMINUS	GO_NEURON_PROJECTION_TERMINUS	103	-0.49219	-1.68267	0	0.010838	0.443	1927	tags=28%, list=19%, signal=34%
GO_MICROTUBULE	GO_MICROTUBULE	253	-0.44575	-1.68123	0	0.010789	0.45	2858	tags=41%, list=28%, signal=55%
GO_INTERCELLULAR_BRIDGE	GO_INTERCELLULAR_BRIDGE	26	-0.61188	-1.67626	0.002886	0.011099	0.467	2935	tags=58%, list=28%, signal=80%
GO_MITOTIC_SPINDLE	GO_MITOTIC_SPINDLE	40	-0.55398	-1.66811	0.005587	0.012431	0.511	2558	tags=52%, list=25%, signal=69%
GO_DNA_REPAIR_COMPLEX	GO_DNA_REPAIR_COMPLEX	27	-0.60804	-1.66534	0.005634	0.012576	0.521	2905	tags=63%, list=28%, signal=87%
GO_CILIARY_PART	GO_CILIARY_PART	155	-0.45999	-1.65393	0	0.014398	0.579	2394	tags=32%, list=23%, signal=40%
GO_DNA_DIRECTED_RNA_POLYMERASE_II_HOLOENZYME	GO_DNA_DIRECTED_RNA_POLYMERASE_II_HOLOENZYME	68	-0.50507	-1.64831	0.002653	0.015226	0.601	4286	tags=69%, list=41%, signal=117%
GO_SPINDLE_MIDZONE	GO_SPINDLE_MIDZONE	21	-0.63041	-1.64415	0.005698	0.015912	0.619	1828	tags=57%, list=18%, signal=69%
GO_PROTEIN_DNA_COMPLEX	GO_PROTEIN_DNA_COMPLEX	107	-0.4749	-1.64335	0	0.015665	0.62	2161	tags=31%, list=21%, signal=39%
GO_ACETYLTRANSFERASE_COMPLEX	GO_ACETYLTRANSFERASE_COMPLEX	48	-0.53887	-1.64001	0.006605	0.015897	0.629	3683	tags=75%, list=35%, signal=116%
GO_PCG_PROTEIN_COMPLEX	GO_PCG_PROTEIN_COMPLEX	35	-0.56571	-1.63854	0.001362	0.015891	0.637	2144	tags=43%, list=21%, signal=54%
GO_CELL_PROJECTION_CYTOPLASM	GO_CELL_PROJECTION_CYTOPLASM	33	-0.57484	-1.63759	0.004225	0.015824	0.643	1767	tags=30%, list=17%, signal=36%

GO_CARBOXY_TERMINAL_DOMAIN_PROTEIN_KINASE_COMPLEX	GO_CARBOXY_TERMINAL_DOMAIN_PROTEIN_KINASE_COMPLEX	17	-0.65634	-1.62333	0.019316	0.018417	0.708	2404	tags=59%, list=23%, signal=76%
GO_SOMATODENDRITIC_COMPARTMENT	GO_SOMATODENDRITIC_COMPARTMENT	486	-0.41182	-1.62209	0	0.018367	0.712	2089	tags=28%, list=20%, signal=33%
GO_SPLICEOSOMAL_TRI_SNRNP_COMPLEX	GO_SPLICEOSOMAL_TRI_SNRNP_COMPLEX	23	-0.61137	-1.62074	0.009777	0.018247	0.715	3350	tags=78%, list=32%, signal=115%
GO_CENTRIOLAR_SATELLITE	GO_CENTRIOLAR_SATELLITE	15	-0.67391	-1.62038	0.016845	0.01799	0.715	2321	tags=67%, list=22%, signal=86%
GO_HETEROCHROMATIN	GO_HETEROCHROMATIN	42	-0.53778	-1.61835	0.009358	0.018115	0.72	2447	tags=48%, list=24%, signal=62%
GO_CATION_CHANNEL_COMPLEX	GO_CATION_CHANNEL_COMPLEX	120	-0.45807	-1.61595	0	0.018412	0.736	1341	tags=17%, list=13%, signal=19%
GO_TRANSPORT_VESICLE_MEMBRANE	GO_TRANSPORT_VESICLE_MEMBRANE	104	-0.46731	-1.61396	0	0.018608	0.74	1979	tags=35%, list=19%, signal=42%
GO_SPINDLE_MICROTUBULE	GO_SPINDLE_MICROTUBULE	45	-0.5311	-1.60497	0.001318	0.020662	0.783	2494	tags=53%, list=24%, signal=70%
GO_CILIARY_TIP	GO_CILIARY_TIP	26	-0.59181	-1.5917	0.011747	0.023897	0.841	2166	tags=46%, list=21%, signal=58%
GO_SEX_CHROMOSOME	GO_SEX_CHROMOSOME	22	-0.60563	-1.5896	0.012894	0.024102	0.845	2960	tags=64%, list=29%, signal=89%
GO_SPERM_FLAGELLUM	GO_SPERM_FLAGELLUM	30	-0.55588	-1.58084	0.021798	0.026195	0.875	1408	tags=30%, list=14%, signal=35%
GO_RIBONUCLEOPROTEIN_COMPLEX	GO_RIBONUCLEOPROTEIN_COMPLEX	402	-0.40812	-1.57592	0	0.027415	0.893	3965	tags=53%, list=38%, signal=83%

GO_MAST_CELL_GRANULE	GO_MAST_CELL_GRANULE	17	-0.62599	-1.56724	0.027536	0.03009	0.912	177	tags=18%, list=2%, signal=18%
GO_CUL3_RING_UBIQUITIN_LIGASE_COMPLEX	GO_CUL3_RING_UBIQUITIN_LIGASE_COMPLEX	31	-0.55228	-1.56645	0.019048	0.029931	0.916	3391	tags=68%, list=33%, signal=100%
GO_SYNAPTIC_MEMBRANE	GO_SYNAPTIC_MEMBRANE	193	-0.42543	-1.56322	0	0.030502	0.92	2089	tags=29%, list=20%, signal=36%
GO_NUCLEAR_TRANSCRIPTION_FACTOR_COMPLEX	GO_NUCLEAR_TRANSCRIPTION_FACTOR_COMPLEX	105	-0.45173	-1.5612	0.006053	0.030675	0.923	2612	tags=42%, list=25%, signal=55%
GO_CILIARY_BASAL_BODY	GO_CILIARY_BASAL_BODY	42	-0.52404	-1.56112	0.014986	0.030295	0.923	2338	tags=48%, list=23%, signal=61%
GO_CHROMATIN	GO_CHROMATIN	300	-0.40628	-1.5573	0	0.031071	0.93	3626	tags=49%, list=35%, signal=73%
GO_MITOCHONDRIAL_MATRIX	GO_MITOCHONDRIAL_MATRIX	283	-0.4093	-1.55377	0	0.031934	0.94	3684	tags=49%, list=35%, signal=74%
GO_AXONAL_GROWTH_CONE	GO_AXONAL_GROWTH_CONE	18	-0.61204	-1.55245	0.027066	0.031908	0.942	2451	tags=67%, list=24%, signal=87%
GO_U2_SNRNP	GO_U2_SNRNP	15	-0.6393	-1.54869	0.024531	0.032597	0.949	2722	tags=60%, list=26%, signal=81%
GO_TRANSCRIPTIONAL_REPRESSOR_COMPLEX	GO_TRANSCRIPTIONAL_REPRESSOR_COMPLEX	51	-0.50121	-1.54729	0.01738	0.032585	0.95	2494	tags=39%, list=24%, signal=51%
GO_CELL_BODY	GO_CELL_BODY	394	-0.39893	-1.54701	0	0.032265	0.95	2089	tags=27%, list=20%, signal=32%
GO_POTASSIUM_CHANNEL_COMPLEX	GO_POTASSIUM_CHANNEL_COMPLEX	68	-0.47917	-1.54697	0.003778	0.031866	0.95	1255	tags=18%, list=12%, signal=20%

GO_PRESYNAPTIC_MEMBRANE	GO_PRESYNAPTIC_MEMBRANE	41	-0.52068	-1.5426	0.021739	0.0329	0.956	1216	tags=32%, list=12%, signal=36%
GO_TRANSFERASE_COMPLEX_TRANSFERRING_PHOSPHORUS_CONTAINING_GROUPS	GO_TRANSFERASE_COMPLEX_TRANSFERRING_PHOSPHORUS_CONTAINING_GROUPS	170	-0.41974	-1.53334	0.002315	0.035911	0.964	2545	tags=39%, list=25%, signal=51%
GO_AXON_CYTOPLASM	GO_AXON_CYTOPLASM	21	-0.58337	-1.53081	0.019802	0.036232	0.965	2341	tags=43%, list=23%, signal=55%
GO_SCF_UBIQUITIN_LIGASE_COMPLEX	GO_SCF_UBIQUITIN_LIGASE_COMPLEX	18	-0.62069	-1.53061	0.020772	0.035946	0.965	3576	tags=67%, list=34%, signal=102%
GO_MITOCHONDRIAL_PROTEIN_COMPLEX	GO_MITOCHONDRIAL_PROTEIN_COMPLEX	91	-0.44818	-1.5273	0.00875	0.036894	0.97	5036	tags=79%, list=48%, signal=152%
GO_CILIUM	GO_CILIUM	240	-0.40483	-1.52605	0.001095	0.036881	0.97	2458	tags=30%, list=24%, signal=38%
GO_U4_U6_X_U5_TRI_SNRNP_COMPLEX	GO_U4_U6_X_U5_TRI_SNRNP_COMPLEX	18	-0.60724	-1.52591	0.020498	0.036511	0.97	3166	tags=72%, list=30%, signal=104%
GO_INNER_MITOCHONDRIAL_MEMBRANE_PROTEIN_COMPLEX	GO_INNER_MITOCHONDRIAL_MEMBRANE_PROTEIN_COMPLEX	70	-0.46305	-1.51616	0.021711	0.039639	0.977	5036	tags=81%, list=48%, signal=157%
GO_U12_TYPE_SPLICEOSOMAL_COMPLEX	GO_U12_TYPE_SPLICEOSOMAL_COMPLEX	17	-0.60714	-1.51497	0.026906	0.039674	0.978	3350	tags=71%, list=32%, signal=104%
GO_MOTILE_CILIUM	GO_MOTILE_CILIUM	53	-0.47802	-1.51361	0.011704	0.039739	0.979	1408	tags=26%, list=14%, signal=30%
GO_HETEROTRIMERIC_G_PROTEIN_COMPLEX	GO_HETEROTRIMERIC_G_PROTEIN_COMPLEX	28	-0.5497	-1.50968	0.024896	0.040791	0.983	628	tags=21%, list=6%, signal=23%

GO_POSTSYNAPTIC_MEMBRANE	GO_POSTSYNAPTIC_MEMBRANE	150	-0.42231	-1.50614	0.001188	0.041885	0.986	2631	tags=31%, list=25%, signal=41%
GO_PRESPLICEOSOME	GO_PRESPLICEOSOME	16	-0.61281	-1.50577	0.030043	0.041604	0.986	2469	tags=56%, list=24%, signal=74%
GO_PERIKARYON	GO_PERIKARYON	81	-0.45565	-1.50366	0.006211	0.042037	0.986	1696	tags=30%, list=16%, signal=35%
GO_HISTONE_DEACETYLASE_COMPLEX	GO_HISTONE_DEACETYLASE_COMPLEX	44	-0.49219	-1.50041	0.016064	0.042871	0.986	2834	tags=41%, list=27%, signal=56%
GO_SYNAPTONEMAL_COMPLEX	GO_SYNAPTONEMAL_COMPLEX	20	-0.58316	-1.49993	0.036179	0.042625	0.987	1589	tags=30%, list=15%, signal=35%
GO_TRANSCRIPTION_FACTOR_TFIID_COMPLEX	GO_TRANSCRIPTION_FACTOR_TFIID_COMPLEX	19	-0.59085	-1.49986	0.041727	0.042226	0.987	2364	tags=53%, list=23%, signal=68%
GO_RESPIRATORY_CHAIN	GO_RESPIRATORY_CHAIN	59	-0.46526	-1.49208	0.023591	0.045041	0.992	4841	tags=78%, list=47%, signal=145%
GO_NUCLEAR_PERIPHERY	GO_NUCLEAR_PERIPHERY	92	-0.43691	-1.492	0.011166	0.044625	0.992	2803	tags=42%, list=27%, signal=58%
GO_NUCLEOID	GO_NUCLEOID	33	-0.51585	-1.49174	0.038028	0.044276	0.993	3640	tags=61%, list=35%, signal=93%
GO_MITOCHONDRIAL_MEMBRANE_PART	GO_MITOCHONDRIAL_MEMBRANE_PART	108	-0.42663	-1.48341	0.009615	0.047541	0.994	4841	tags=73%, list=47%, signal=136%
GO_RNA_POLYMERASE_II_TRANSCRIPTION_FACTOR_COMPLEX	GO_RNA_POLYMERASE_II_TRANSCRIPTION_FACTOR_COMPLEX	84	-0.4446	-1.48172	0.01233	0.047844	0.994	2721	tags=44%, list=26%, signal=59%
GO_MLL1_2_COMPLEX	GO_MLL1_2_COMPLEX	21	-0.57027	-1.4809	0.041018	0.047715	0.994	3616	tags=71%, list=35%, signal=109%

GO_ORGANELLAR_RIBOSOME	GO_ORGANELLAR_RIBOSOME	46	-0.48802	-1.48053	0.027397	0.047455	0.995	3916	tags=65%, list=38%, signal=104%
GO_DYNEIN_COMPLEX	GO_DYNEIN_COMPLEX	28	-0.53175	-1.47935	0.031294	0.047704	0.995	2229	tags=39%, list=21%, signal=50%
GO_NUCLEAR_REPLICATION_FORK	GO_NUCLEAR_REPLICATION_FORK	27	-0.54019	-1.47892	0.030345	0.047467	0.995	3598	tags=70%, list=35%, signal=107%
GO_INO80_TYPE_COMPLEX	GO_INO80_TYPE_COMPLEX	16	-0.61388	-1.47527	0.039074	0.048559	0.997	3974	tags=94%, list=38%, signal=152%
GO_PHOTORECEPTOR_INNER_SEGMENT	GO_PHOTORECEPTOR_INNER_SEGMENT	29	-0.52667	-1.47312	0.05007	0.049067	0.997	1114	tags=21%, list=11%, signal=23%
GO_NUCLEAR_CHROMATIN	GO_NUCLEAR_CHROMATIN	204	-0.39778	-1.47155	0.00444	0.049285	0.997	3001	tags=41%, list=29%, signal=57%
GO_MYELIN_SHEATH	GO_MYELIN_SHEATH	134	-0.41208	-1.46987	0.005952	0.049601	0.997	1597	tags=22%, list=15%, signal=26%
GO_NUCLEAR_BODY	GO_NUCLEAR_BODY	231	-0.39501	-1.46867	0.001098	0.049793	0.998	3662	tags=48%, list=35%, signal=73%
GO_PHOTORECEPTOR_CONNECTING_CILIUM	GO_PHOTORECEPTOR_CONNECTING_CILIUM	23	-0.55497	-1.46697	0.038012	0.050319	0.998	2166	tags=43%, list=21%, signal=55%
GO_CYCLIN_DEPENDENT_PROTEIN_KINASE_HOLOENZYME_COMPLEX	GO_CYCLIN_DEPENDENT_PROTEIN_KINASE_HOLOENZYME_COMPLEX	22	-0.55959	-1.46563	0.049708	0.050605	0.999	2304	tags=59%, list=22%, signal=76%
GO_INTRACILIARY_TRANSPORT_PARTICLE	GO_INTRACILIARY_TRANSPORT_PARTICLE	17	-0.59585	-1.46356	0.057225	0.05114	0.999	2166	tags=53%, list=21%, signal=67%

GO_DNA_PACKAGING_COMPLEX	GO_DNA_PACKAGING_COMPLEX	56	-0.46754	-1.46291	0.034346	0.051032	0.999	1984	tags=27%, list=19%, signal=33%
GO_CYTOPLASMIC_MICROTUBULE	GO_CYTOPLASMIC_MICROTUBULE	40	-0.49044	-1.46002	0.030544	0.052018	0.999	3231	tags=45%, list=31%, signal=65%
GO_PRIMARY_CILIUM	GO_PRIMARY_CILIUM	118	-0.4128	-1.4507	0.006053	0.056049	0.999	3302	tags=42%, list=32%, signal=60%
GO_NEURON_PROJECTION_MEMBRANE	GO_NEURON_PROJECTION_MEMBRANE	27	-0.52603	-1.44799	0.050068	0.057073	0.999	1613	tags=30%, list=16%, signal=35%
GO_ORGANELLE_INNER_MEMBRANE	GO_ORGANELLE_INNER_MEMBRANE	330	-0.3776	-1.44781	0	0.056727	0.999	3934	tags=50%, list=38%, signal=78%
GO_NUCLEOLAR_PART	GO_NUCLEOLAR_PART	38	-0.47871	-1.43318	0.052703	0.064077	0.999	3363	tags=53%, list=32%, signal=78%
GO_TRANSPORTER_COMPLEX	GO_TRANSPORTER_COMPLEX	229	-0.38088	-1.43075	0.004425	0.064752	1	2354	tags=23%, list=23%, signal=29%
GO_CLATHRIN_COATED_VESICLE_MEMBRANE	GO_CLATHRIN_COATED_VESICLE_MEMBRANE	57	-0.45332	-1.42682	0.034574	0.066232	1	976	tags=19%, list=9%, signal=21%
GO_SNARE_COMPLEX	GO_SNARE_COMPLEX	40	-0.46701	-1.40781	0.043776	0.076937	1	2450	tags=40%, list=24%, signal=52%
GO_NUCLEAR_EUCHROMATIN	GO_NUCLEAR_EUCHROMATIN	17	-0.56397	-1.40064	0.093514	0.080878	1	1675	tags=41%, list=16%, signal=49%
GO_SUPRAMOLECULAR_FIBER	GO_SUPRAMOLECULAR_FIBER	365	-0.36277	-1.40017	0.001048	0.080523	1	2858	tags=34%, list=28%, signal=45%

GO_EUCHROMATIN	GO_EUCHROMATIN	22	-0.54507	-1.39879	0.063401	0.080824	1	1675	tags=36%, list=16%, signal=43%
GO_NUCLEAR_MATRIX	GO_NUCLEAR_MATRIX	72	-0.42483	-1.39836	0.03607	0.080507	1	2803	tags=43%, list=27%, signal=59%
GO_NADH_DEHYDROGENASE_COMPLEX	GO_NADH_DEHYDROGENASE_COMPLEX	32	-0.49039	-1.39249	0.063291	0.083698	1	4980	tags=84%, list=48%, signal=162%
GO_REPLISOME	GO_REPLISOME	22	-0.51823	-1.39076	0.086765	0.084223	1	3965	tags=77%, list=38%, signal=125%
GO_BAF_TYPE_COMPLEX	GO_BAF_TYPE_COMPLEX	18	-0.5502	-1.3871	0.079886	0.085887	1	2758	tags=67%, list=27%, signal=91%
GO_U2_TYPE_SPLICEOSOMAL_COMPLEX	GO_U2_TYPE_SPLICEOSOMAL_COMPLEX	21	-0.51799	-1.38164	0.075823	0.089147	1	2469	tags=38%, list=24%, signal=50%

Enriched pathways in GO molecular function for CTSI high risk group

NAME	GS<br>follow link to MSigDB	SIZE	ES	NES	NOM p-val	FDR q-val	FWER p-val	RANK AT MAX	LEADING EDGE
GO_INTEGRIN_BINDING	GO_INTEGRIN_BINDING	93	0.65477	2.6964	0	0	0	1661	tags=46%, list=16%, signal=55%
GO_COLLAGEN_BINDING	GO_COLLAGEN_BINDING	50	0.6975	2.4789	0	0	0	1393	tags=54%, list=13%, signal=62%
GO_CYTOKINE_RECEPTOR_ACTIVITY	GO_CYTOKINE_RECEPTOR_ACTIVITY	69	0.64823	2.461	0	0	0	1434	tags=48%, list=14%, signal=55%
GO_SERINE_HYDROLASE_ACTIVITY	GO_SERINE_HYDROLASE_ACTIVITY	141	0.56427	2.4514	0	0	0	1302	tags=31%, list=13%, signal=35%
GO_GROWTH_FACTOR_BINDING	GO_GROWTH_FACTOR_BINDING	107	0.60212	2.4439	0	0	0	1211	tags=36%, list=12%, signal=41%
GO_CARGO_RECEPTOR_ACTIVITY	GO_CARGO_RECEPTOR_ACTIVITY	41	0.70365	2.3995	0	0	0	672	tags=41%, list=6%, signal=44%
GO_CARBOHYDRATE_BINDING	GO_CARBOHYDRATE_BINDING	167	0.54145	2.37	0	0	0	1112	tags=30%, list=11%, signal=33%
GO_SCAVENGER_RECEPTOR_ACTIVITY	GO_SCAVENGER_RECEPTOR_ACTIVITY	22	0.76985	2.346	0	0	0	424	tags=41%, list=4%, signal=43%
GO_PEPTIDE_ANTIGEN_BINDING	GO_PEPTIDE_ANTIGEN_BINDING	23	0.75041	2.3048	0	0	0	1963	tags=74%, list=19%, signal=91%
GO_CYTOKINE_ACTIVITY	GO_CYTOKINE_ACTIVITY	148	0.51837	2.2645	0	0	0	2440	tags=48%, list=23%, signal=62%
GO_ANTIGEN_BINDING	GO_ANTIGEN_BINDING	64	0.59599	2.2413	0	3.81E-04	0.003	2023	tags=55%, list=19%, signal=68%
GO_VIRUS_RECEPTOR_ACTIVITY	GO_VIRUS_RECEPTOR_ACTIVITY	56	0.60562	2.2104	0	3.50E-04	0.003	986	tags=39%, list=9%, signal=43%
GO_CYTOKINE_BINDING	GO_CYTOKINE_BINDING	71	0.57307	2.2002	0	3.23E-04	0.003	1053	tags=37%, list=10%, signal=40%

GO_GLYCOPROTEIN_BINDING	GO_GLYCOPROTEIN_BINDING	85	0.56334	2.1926	0	3.95E-04	0.004	1341	tags=36%, list=13%, signal=42%
GO_INSULIN_LIKE_GROWTH_FACTOR_BINDING	GO_INSULIN_LIKE_GROWTH_FACTOR_BINDING	21	0.7384	2.1894	0	3.69E-04	0.004	998	tags=38%, list=10%, signal=42%
GO_PEPTIDASE_REGULATOR_ACTIVITY	GO_PEPTIDASE_REGULATOR_ACTIVITY	145	0.50265	2.1857	0	3.46E-04	0.004	1164	tags=26%, list=11%, signal=29%
GO_IMMUNOGLOBULIN_BINDING	GO_IMMUNOGLOBULIN_BINDING	17	0.77603	2.1752	0	3.26E-04	0.004	1189	tags=41%, list=11%, signal=46%
GO_MONOSACCHARIDE_BINDING	GO_MONOSACCHARIDE_BINDING	57	0.59848	2.1737	0	3.07E-04	0.004	1112	tags=33%, list=11%, signal=37%
GO_PEPTIDASE_INHIBITOR_ACTIVITY	GO_PEPTIDASE_INHIBITOR_ACTIVITY	114	0.52363	2.1684	0	2.91E-04	0.004	1164	tags=26%, list=11%, signal=29%
GO_DEATH_RECEPTOR_ACTIVITY	GO_DEATH_RECEPTOR_ACTIVITY	17	0.77053	2.1629	0	2.77E-04	0.004	1732	tags=71%, list=17%, signal=85%
GO_CELL_ADHESION_MOLECULE_BINDING	GO_CELL_ADHESION_MOLECULE_BINDING	156	0.50202	2.1496	0	4.04E-04	0.006	1466	tags=36%, list=14%, signal=41%
GO_EXTRACELLULAR_MATRIX_STRUCTURAL_CONSTITUENT	GO_EXTRACELLULAR_MATRIX_STRUCTURAL_CONSTITUENT	59	0.58253	2.1419	0	3.85E-04	0.006	768	tags=34%, list=7%, signal=36%
GO_COMPLEMENT_BINDING	GO_COMPLEMENT_BINDING	17	0.7485	2.1264	0	4.92E-04	0.008	1239	tags=47%, list=12%, signal=53%
GO_HEPARIN_BINDING	GO_HEPARIN_BINDING	118	0.50359	2.1238	0	4.72E-04	0.008	697	tags=21%, list=7%, signal=22%
GO_PROTEOGLYCAN_BINDING	GO_PROTEOGLYCAN_BINDING	26	0.66606	2.109	0	6.33E-04	0.011	604	tags=31%, list=6%, signal=33%
GO_PROTEASE_BINDING	GO_PROTEASE_BINDING	86	0.53554	2.0933	0	7.78E-04	0.014	1174	tags=35%, list=11%, signal=39%
GO_MHC_PROTEIN_BINDING	GO_MHC_PROTEIN_BINDING	20	0.71659	2.0915	0	7.49E-04	0.014	1446	tags=45%, list=14%, signal=52%
GO_CHEMOKINE_ACTIVITY	GO_CHEMOKINE_ACTIVITY	35	0.61654	2.0725	0	7.68E-04	0.015	1741	tags=43%, list=17%, signal=51%

GO_HYDROLASE_ACTIVITY_HYDROLYZING_O_GLYCOSYL_COMPOUNDS	GO_HYDROLASE_ACTIVITY_HYDROLYZING_O_GLYCOSYL_COMPOUNDS	56	0.5622	2.0682	0	7.91E-04	0.016	1075	tags=39%, list=10%, signal=44%
GO_COPPER_ION_BINDING	GO_COPPER_ION_BINDING	41	0.59103	2.0401	0	0.001263	0.026	616	tags=22%, list=6%, signal=23%
GO_CHEMOKINE_RECEPTOR_BINDING	GO_CHEMOKINE_RECEPTOR_BINDING	43	0.58991	2.0392	0	0.001222	0.026	1741	tags=42%, list=17%, signal=50%
GO_EXTRACELLULAR_MATRIX_BINDING	GO_EXTRACELLULAR_MATRIX_BINDING	38	0.61561	2.0342	0	0.00127	0.028	1948	tags=53%, list=19%, signal=65%
GO_FIBRONECTIN_BINDING	GO_FIBRONECTIN_BINDING	21	0.68132	2.0268	0	0.001364	0.031	1393	tags=48%, list=13%, signal=55%
GO_CYTOKINE_RECEPTOR_BINDING	GO_CYTOKINE_RECEPTOR_BINDING	201	0.45096	2.0218	0	0.001366	0.032	2299	tags=43%, list=22%, signal=54%
GO_SERINE_TYPE_ENDOPEPTIDASE_INHIBITOR_ACTIVITY	GO_SERINE_TYPE_ENDOPEPTIDASE_INHIBITOR_ACTIVITY	60	0.53769	2.0178	0	0.001466	0.035	1162	tags=27%, list=11%, signal=30%
GO_GLYCOSAMINOGLYCAN_BINDING	GO_GLYCOSAMINOGLYCAN_BINDING	147	0.45793	2.0053	0	0.001906	0.047	1227	tags=28%, list=12%, signal=31%
GO_OXIDOREDUCTASE_ACTIVITY_ACTING_ON_PAIRED_DONORS_WITH_INCORPORATION_OR_REDUCTION_OF_MOLECULAR_OXYGEN	GO_OXIDOREDUCTASE_ACTIVITY_ACTING_ON_PAIRED_DONORS_WITH_INCORPORATION_OR_REDUCTION_OF_MOLECULAR_OXYGEN	90	0.49027	2.0046	0	0.001891	0.048	815	tags=19%, list=8%, signal=20%
GO_PROTEIN_LIPID_COMPLEX_BINDING	GO_PROTEIN_LIPID_COMPLEX_BINDING	21	0.64772	1.9281	0	0.004443	0.114	672	tags=33%, list=6%, signal=36%
GO_ENDOPEPTIDASE_ACTIVITY	GO_ENDOPEPTIDASE_ACTIVITY	280	0.39253	1.8466	0	0.010068	0.244	946	tags=19%, list=9%, signal=20%
GO_CXCR_CHEMOKINE_RECEPTOR_BINDING	GO_CXCR_CHEMOKINE_RECEPTOR_BINDING	15	0.6723	1.84	0.011561	0.010327	0.255	1686	tags=47%, list=16%, signal=56%

GO_MONOCARBOXYLIC_ACID_BINDING	GO_MONOCARBOXYLIC_ACID_BINDING	47	0.52052	1.8394	0	0.010146	0.257	962	tags=26%, list=9%, signal=28%
GO_LAMININ_BINDING	GO_LAMININ_BINDING	22	0.6126	1.8131	0.003497	0.012748	0.318	1948	tags=55%, list=19%, signal=67%
GO_OXIDOREDUCTASE_ACTIVITY_ACTING_ON_PEROXIDE_AS_ACCEPTOR	GO_OXIDOREDUCTASE_ACTIVITY_ACTING_ON_PEROXIDE_AS_ACCEPTOR	30	0.5613	1.7865	0.010169	0.016317	0.391	1247	tags=33%, list=12%, signal=38%
GO_FATTY_ACID_BINDING	GO_FATTY_ACID_BINDING	25	0.57635	1.7789	0.006557	0.016893	0.413	1193	tags=36%, list=11%, signal=41%
GO_OXIDOREDUCTASE_ACTIVITY_ACTING_ON_THE_CH_NH2_GROUP_OF_DONORS	GO_OXIDOREDUCTASE_ACTIVITY_ACTING_ON_THE_CH_NH2_GROUP_OF_DONORS	15	0.64207	1.7566	0.009259	0.019779	0.48	355	tags=20%, list=3%, signal=21%
GO_ANTIOXIDANT_ACTIVITY	GO_ANTIOXIDANT_ACTIVITY	47	0.49656	1.7547	0	0.019615	0.482	1020	tags=23%, list=10%, signal=26%
GO_OXIDOREDUCTASE_ACTIVITY_ACTING_ON_PAIRED_DONORS_WITH_INCORPORATION_OR_REDUCTION_OF_MOLECULAR_OXYGEN_2_OXOGLUTARATE_AS_ONE_DONOR_AND_INCORPORATION_OF_ONE_ATOM_EACH_OF_OXYGEN_INTO_BOTH_DONORS	GO_OXIDOREDUCTASE_ACTIVITY_ACTING_ON_PAIRED_DONORS_WITH_INCORPORATION_OR_REDUCTION_OF_MOLECULAR_OXYGEN_2_OXOGLUTARATE_AS_ONE_DONOR_AND_INCORPORATION_OF_ONE_ATOM_EACH_OF_OXYGEN_INTO_BOTH_DONORS	19	0.60708	1.7492	0.012945	0.020112	0.5	815	tags=32%, list=8%, signal=34%
GO_SULFUR_COMPOUND_BINDING	GO_SULFUR_COMPOUND_BINDING	177	0.38572	1.7384	0	0.021452	0.529	1543	tags=27%, list=15%, signal=31%
GO_DIOXYGENASE_ACTIVITY	GO_DIOXYGENASE_ACTIVITY	41	0.4988	1.7382	0.007968	0.021049	0.53	815	tags=27%, list=8%, signal=29%

GO_CYSTEINE_TYPE_ENDOPEPTIDASE_INHIBITOR_ACTIVITY	GO_CYSTEINE_TYPE_ENDOPEPTIDASE_INHIBITOR_ACTIVITY	40	0.50649	1.7249	0.003802	0.022872	0.566	1366	tags=28%, list=13%, signal=32%
GO_PROTEIN_PHOSPHORYLATED_AMINO_ACID_BINDING	GO_PROTEIN_PHOSPHORYLATED_AMINO_ACID_BINDING	21	0.57803	1.7123	0.009901	0.025181	0.604	404	tags=24%, list=4%, signal=25%
GO_CYSTEINE_TYPE_ENDOPEPTIDASE_INHIBITOR_ACTIVITY_INVOLVED_IN_APOPTOTIC_PROCESS	GO_CYSTEINE_TYPE_ENDOPEPTIDASE_INHIBITOR_ACTIVITY_INVOLVED_IN_APOPTOTIC_PROCESS	18	0.59352	1.7038	0.009836	0.026816	0.637	1019	tags=39%, list=10%, signal=43%
GO_CCR_CHEMOKINE_RECEPTOR_BINDING	GO_CCR_CHEMOKINE_RECEPTOR_BINDING	24	0.55404	1.6947	0.003333	0.028603	0.667	2337	tags=54%, list=23%, signal=70%
GO_ENZYME_INHIBITOR_ACTIVITY	GO_ENZYME_INHIBITOR_ACTIVITY	249	0.36302	1.6766	0	0.032509	0.716	1198	tags=22%, list=12%, signal=24%
GO_HYDROLASE_ACTIVITY_ACTING_ON_GLYCOSYL_BONDS	GO_HYDROLASE_ACTIVITY_ACTING_ON_GLYCOSYL_BONDS	74	0.42896	1.6568	0.00495	0.037039	0.765	1075	tags=30%, list=10%, signal=33%
GO_VITAMIN_TRANSPORTER_ACTIVITY	GO_VITAMIN_TRANSPORTER_ACTIVITY	18	0.56821	1.6281	0.013245	0.045473	0.834	838	tags=28%, list=8%, signal=30%
GO_PEPTIDASE_ACTIVITY	GO_PEPTIDASE_ACTIVITY	386	0.33917	1.6227	0	0.046263	0.842	1361	tags=22%, list=13%, signal=24%
GO_METALLOENDOPEPTIDASE_ACTIVITY	GO_METALLOENDOPEPTIDASE_ACTIVITY	80	0.41304	1.6099	0	0.049811	0.863	1361	tags=26%, list=13%, signal=30%
GO_SMAD_BINDING	GO_SMAD_BINDING	57	0.4278	1.5938	0.008264	0.055396	0.902	775	tags=21%, list=7%, signal=23%
GO_IRON_ION_BINDING	GO_IRON_ION_BINDING	96	0.40174	1.59	0	0.056187	0.904	771	tags=15%, list=7%, signal=16%

GO_OXIDOREDUCTASE_ACTIVITY_ACTING_ON_SINGLE_DONORS_WITH_INCORPORATION_OF_MOLECULAR_OXYGEN	GO_OXIDOREDUCTASE_ACTIVITY_ACTING_ON_SINGLE_DONORS_WITH_INCORPORATION_OF_MOLECULAR_OXYGEN	18	0.57105	1.5877	0.042169	0.056278	0.907	707	tags=33%, list=7%, signal=36%
GO_TETRAPYRROLE_BINDING	GO_TETRAPYRROLE_BINDING	95	0.39299	1.5729	0.005618	0.061246	0.928	1011	tags=16%, list=10%, signal=17%
GO_RAB_GUANYL_NUCLEOTIDE_EXCHANGE_FACTOR_ACTIVITY	GO_RAB_GUANYL_NUCLEOTIDE_EXCHANGE_FACTOR_ACTIVITY	16	0.58528	1.5668	0.025157	0.063016	0.936	555	tags=31%, list=5%, signal=33%
GO_NON_MEMBRANE_SPANNING_PROTEIN_TYROSINE_KINASE_ACTIVITY	GO_NON_MEMBRANE_SPANNING_PROTEIN_TYROSINE_KINASE_ACTIVITY	40	0.44485	1.5445	0.01087	0.073137	0.966	978	tags=23%, list=9%, signal=25%
GO_PROTEIN_DISULFIDE_OXIDOREDUCTASE_ACTIVITY	GO_PROTEIN_DISULFIDE_OXIDOREDUCTASE_ACTIVITY	17	0.54483	1.5423	0.04886	0.073282	0.969	2444	tags=65%, list=24%, signal=84%
GO_OXIDOREDUCTASE_ACTIVITY_ACTING_ON_PAIRED_DONORS_WITH_INCORPORATION_OR_REDUCTION_OF_MOLECULAR_OXYGEN_REDUCED_FLAVIN_OR_FLAVOPROTEIN_AS_ONE_DONOR_AND_INCORPORATION_OF_ONE_ATOM_OF_OXYGEN	GO_OXIDOREDUCTASE_ACTIVITY_ACTING_ON_PAIRED_DONORS_WITH_INCORPORATION_OR_REDUCTION_OF_MOLECULAR_OXYGEN_REDUCED_FLAVIN_OR_FLAVOPROTEIN_AS_ONE_DONOR_AND_INCORPORATION_OF_ONE_ATOM_OF_OXYGEN	18	0.53072	1.5289	0.029508	0.07886	0.978	2200	tags=39%, list=21%, signal=49%

GO_DISULFIDE_OXIDOREDUCTASE_ACTIVITY	GO_DISULFIDE_OXIDOREDUCTASE_ACTIVITY	21	0.51304	1.4962	0.043333	0.095826	0.991	2444	tags=62%, list=24%, signal=81%
GO_METALLOPEPTIDASE_ACTIVITY	GO_METALLOPEPTIDASE_ACTIVITY	125	0.36026	1.491	0.005376	0.098166	0.993	1796	tags=28%, list=17%, signal=33%

Enriched pathways in GO molecular function for CTSI low risk group

NAME	GS<br>follow link to MSigDB	SIZE	ES	NES	NOM p-val	FDR q-val	FWER p-val	RANK AT MAX	LEADING EDGE
GO_VOLTAGE_GATED_SODIUM_CHANNEL_ACTIVITY	GO_VOLTAGE_GATED_SODIUM_CHANNEL_ACTIVITY	15	-0.78652	-1.8984	0	0.027739	0.032	125	tags=20%, list=1%, signal=20%
GO_MICROTUBULE_MOTOR_ACTIVITY	GO_MICROTUBULE_MOTOR_ACTIVITY	45	-0.60837	-1.85	0	0.034622	0.078	1777	tags=44%, list=17%, signal=53%
GO_TUBULIN_BINDING	GO_TUBULIN_BINDING	182	-0.49016	-1.8037	0	0.052657	0.168	2451	tags=43%, list=24%, signal=55%
GO_DNA_HELICASE_ACTIVITY	GO_DNA_HELICASE_ACTIVITY	41	-0.60086	-1.7955	0	0.04724	0.199	2888	tags=56%, list=28%, signal=77%
GO_ALKALI_METAL_ION_BINDING	GO_ALKALI_METAL_ION_BINDING	16	-0.73447	-1.7804	0.001456	0.048461	0.244	885	tags=38%, list=9%, signal=41%
GO_DAMAGED_DNA_BINDING	GO_DAMAGED_DNA_BINDING	54	-0.56876	-1.7673	0.00134	0.048052	0.278	3755	tags=76%, list=36%, signal=118%
GO_SINGLE_STRANDED_DNA_BINDING	GO_SINGLE_STRANDED_DNA_BINDING	61	-0.53047	-1.7258	0.002597	0.074507	0.452	2349	tags=43%, list=23%, signal=55%
GO_UBIQUITIN_LIKE_PROTEIN_TRANSFERASE_ACTIVITY	GO_UBIQUITIN_LIKE_PROTEIN_TRANSFERASE_ACTIVITY	229	-0.457	-1.7072	0	0.087302	0.545	3785	tags=57%, list=36%, signal=88%
GO_SODIUM_CHANNEL_ACTIVITY	GO_SODIUM_CHANNEL_ACTIVITY	23	-0.64715	-1.6996	0.004317	0.084969	0.58	212	tags=17%, list=2%, signal=18%
GO_ALPHA_TUBULIN_BINDING	GO_ALPHA_TUBULIN_BINDING	19	-0.66052	-1.6811	0.010309	0.0971	0.682	1473	tags=42%, list=14%, signal=49%

GO_UBIQUITIN_LIKE_PROTEIN_LIGASE_ACTIVITY	GO_UBIQUITIN_LIKE_PROTEIN_LIGASE_ACTIVITY	108	-0.48375	-1.6811	0	0.088272	0.682	3785	tags=57%, list=36%, signal=89%
GO_POTASSIUM_ION_TRANSMEMBRANE_TRANSPORTER_ACTIVITY	GO_POTASSIUM_ION_TRANSMEMBRANE_TRANSPORTER_ACTIVITY	102	-0.48628	-1.6791	0.001233	0.083428	0.688	425	tags=12%, list=4%, signal=12%
GO_DNA_DEPENDENT_ATPASE_ACTIVITY	GO_DNA_DEPENDENT_ATPASE_ACTIVITY	59	-0.51941	-1.6636	0.001267	0.096158	0.764	3649	tags=59%, list=35%, signal=91%
GO_DNA_SECONDARY_STRUCTURE_BINDING	GO_DNA_SECONDARY_STRUCTURE_BINDING	21	-0.64015	-1.6434	0.005435	0.115516	0.854	2278	tags=57%, list=22%, signal=73%
GO_MICROTUBULE_BINDING	GO_MICROTUBULE_BINDING	132	-0.46036	-1.6265	0	0.109531	0.899	2451	tags=42%, list=24%, signal=54%
GO_TRANSFERASE_ACTIVITY_TRANSFERRING_ONE_CARBON_GROUPS	GO_TRANSFERASE_ACTIVITY_TRANSFERRING_ONE_CARBON_GROUPS	111	-0.46983	-1.6347	0	0.118501	0.876	2983	tags=47%, list=29%, signal=65%
GO_NEUROPEPTIDE_HORMONE_ACTIVITY	GO_NEUROPEPTIDE_HORMONE_ACTIVITY	25	-0.60605	-1.6301	0.007003	0.118151	0.897	928	tags=24%, list=9%, signal=26%
GO_DELAYED_RECTIFIER_POTASSIUM_CHANNEL_ACTIVITY	GO_DELAYED_RECTIFIER_POTASSIUM_CHANNEL_ACTIVITY	28	-0.59115	-1.6285	0.011527	0.113746	0.899	393	tags=11%, list=4%, signal=11%
GO_REPRESSING_TRANSCRIPTION_FACTOR_BINDING	GO_REPRESSING_TRANSCRIPTION_FACTOR_BINDING	40	-0.53913	-1.6175	0.007958	0.116501	0.917	2149	tags=43%, list=21%, signal=53%
GO_VOLTAGE_GATED_POTASSIUM_CHANNEL_ACTIVITY	GO_VOLTAGE_GATED_POTASSIUM_CHANNEL_ACTIVITY	61	-0.49555	-1.5894	0.002632	0.125116	0.964	393	tags=10%, list=4%, signal=10%
GO_PROTEIN_DEACETYLASE_ACTIVITY	GO_PROTEIN_DEACETYLASE_ACTIVITY	33	-0.55807	-1.6042	0.008174	0.128782	0.946	1915	tags=36%, list=18%, signal=44%
GO_BETA_TUBULIN_BINDING	GO_BETA_TUBULIN_BINDING	33	-0.55892	-1.6022	0.005405	0.125728	0.952	2428	tags=45%, list=23%, signal=59%

GO_NUCLEOBASE_CONTAINING_COMPOUND_KINASE_ACTIVITY	GO_NUCLEOBASE_CONTAINING_COMPOUND_KINASE_ACTIVITY	27	-0.56936	-1.594	0.012876	0.130598	0.96	2965	tags=56%, list=29%, signal=78%
GO_CYCLIN_DEPENDENT_PROTEIN_KINASE_ACTIVITY	GO_CYCLIN_DEPENDENT_PROTEIN_KINASE_ACTIVITY	18	-0.6368	-1.5901	0.014514	0.129462	0.964	2086	tags=50%, list=20%, signal=62%
GO_CHROMATIN_BINDING	GO_CHROMATIN_BINDING	315	-0.41505	-1.5802	0	0.133431	0.97	3389	tags=48%, list=33%, signal=69%
GO_HISTONE_BINDING	GO_HISTONE_BINDING	98	-0.45795	-1.5792	0.002439	0.1296	0.97	3382	tags=47%, list=33%, signal=69%
GO_HISTONE_KINASE_ACTIVITY	GO_HISTONE_KINASE_ACTIVITY	15	-0.64647	-1.5757	0.014577	0.129276	0.976	2417	tags=60%, list=23%, signal=78%
GO_HELICASE_ACTIVITY	GO_HELICASE_ACTIVITY	103	-0.4491	-1.5628	0.00123	0.143671	0.989	2888	tags=45%, list=28%, signal=61%
GO_RNA_POLYMERASE_ACTIVITY	GO_RNA_POLYMERASE_ACTIVITY	29	-0.55315	-1.5548	0.016854	0.15137	0.992	2940	tags=55%, list=28%, signal=77%
GO_EXONUCLEASE_ACTIVITY	GO_EXONUCLEASE_ACTIVITY	43	-0.51569	-1.5527	0.021477	0.149585	0.992	2893	tags=56%, list=28%, signal=77%
GO_HISTONE_DEACETYLASE_BINDING	GO_HISTONE_DEACETYLASE_BINDING	83	-0.45993	-1.548	0.003802	0.151305	0.995	1697	tags=29%, list=16%, signal=34%
GO_CALCIUM_CHANNEL_REGULATOR_ACTIVITY	GO_CALCIUM_CHANNEL_REGULATOR_ACTIVITY	29	-0.55568	-1.548	0.02507	0.146685	0.995	786	tags=24%, list=8%, signal=26%
GO_STRUCTURE_SPECIFIC_DNA_BINDING	GO_STRUCTURE_SPECIFIC_DNA_BINDING	89	-0.46203	-1.5465	0.006061	0.144382	0.996	2761	tags=40%, list=27%, signal=55%
GO_PROTEIN_METHYLTRANSFERASE_ACTIVITY	GO_PROTEIN_METHYLTRANSFERASE_ACTIVITY	47	-0.50402	-1.5395	0.012064	0.150398	0.998	3363	tags=57%, list=32%, signal=85%
GO_SODIUM_ION_TRANSMEMBRANE_TRANSPORTER_ACTIVITY	GO_SODIUM_ION_TRANSMEMBRANE_TRANSPORTER_ACTIVITY	96	-0.4486	-1.5299	0.002481	0.161245	0.999	1217	tags=16%, list=12%, signal=18%

GO_GABA_RECEPTOR_ACTIVITY	GO_GABA_RECEPTOR_ACTIVITY	18	-0.60494	-1.5203	0.029851	0.173969	0.999	2499	tags=44%, list=24%, signal=58%
GO_NEUROPEPTIDE_RECEPTOR_BINDING	GO_NEUROPEPTIDE_RECEPTOR_BINDING	21	-0.579	-1.5103	0.038516	0.18646	1	180	tags=14%, list=2%, signal=15%
GO_EXONUCLEASE_ACTIVITY_ACTIVE_WITH_EITHER_RIBO_OR_DEOXYRIBONUCLEIC_ACIDS_AND_PRODUCING_5_PHOSPHOMONOESTERS	GO_EXONUCLEASE_ACTIVITY_ACTIVE_WITH_EITHER_RIBO_OR_DEOXYRIBONUCLEIC_ACIDS_AND_PRODUCING_5_PHOSPHOMONOESTERS	29	-0.53859	-1.5074	0.032564	0.186493	1	2893	tags=59%, list=28%, signal=81%
GO_POTASSIUM_CHANNEL_ACTIVITY	GO_POTASSIUM_CHANNEL_ACTIVITY	82	-0.4471	-1.5007	0.012723	0.192437	1	1560	tags=17%, list=15%, signal=20%
GO_DEOXYRIBONUCLEASE_ACTIVITY	GO_DEOXYRIBONUCLEASE_ACTIVITY	44	-0.48954	-1.4954	0.015068	0.196975	1	2606	tags=43%, list=25%, signal=57%
GO_RNA_POLYMERASE_II_TRANSCRIPTION_COFACTOR_ACTIVITY	GO_RNA_POLYMERASE_II_TRANSCRIPTION_COFACTOR_ACTIVITY	56	-0.46629	-1.4926	0.014249	0.196788	1	2545	tags=41%, list=25%, signal=54%
GO_LYSINE_N_METHYLTRANSFERASE_ACTIVITY	GO_LYSINE_N_METHYLTRANSFERASE_ACTIVITY	31	-0.53072	-1.4843	0.026239	0.20855	1	2850	tags=48%, list=27%, signal=66%
GO_S_ADENOSYLMETHIONINE_DEPENDENT_METHYLTRANSFERASE_ACTIVITY	GO_S_ADENOSYLMETHIONINE_DEPENDENT_METHYLTRANSFERASE_ACTIVITY	72	-0.45577	-1.4797	0.011583	0.212235	1	3363	tags=51%, list=32%, signal=75%
GO_HISTONE_METHYLTRANSFERASE_ACTIVITY	GO_HISTONE_METHYLTRANSFERASE_ACTIVITY	38	-0.49682	-1.4777	0.033157	0.210719	1	3651	tags=61%, list=35%, signal=93%
GO_BASAL_TRANSCRIPTION_MACHINERY_BINDING	GO_BASAL_TRANSCRIPTION_MACHINERY_BINDING	16	-0.59687	-1.4776	0.047826	0.206303	1	3363	tags=63%, list=32%, signal=92%

GO_ACTININ_BINDING	GO_ACTININ_BINDING	23	-0.54971	-1.4756	0.044444	0.205195	1	471	tags=17%, list=5%, signal=18%
GO_FIBROBLAST_GROWTH_FACTOR_RECEPTOR_BINDING	GO_FIBROBLAST_GROWTH_FACTOR_RECEPTOR_BINDING	22	-0.57599	-1.4718	0.035398	0.207929	1	1936	tags=32%, list=19%, signal=39%
GO_CLATHRIN_BINDING	GO_CLATHRIN_BINDING	31	-0.52463	-1.4708	0.031637	0.2056	1	549	tags=16%, list=5%, signal=17%
GO_DICARBOXYLIC_ACID_TRANSMEMBRANE_TRANSPORTER_ACTIVITY	GO_DICARBOXYLIC_ACID_TRANSMEMBRANE_TRANSPORTER_ACTIVITY	24	-0.54327	-1.4692	0.043541	0.204457	1	1689	tags=33%, list=16%, signal=40%
GO_VOLTAGE_GATED_ION_CHANNEL_ACTIVITY	GO_VOLTAGE_GATED_ION_CHANNEL_ACTIVITY	128	-0.41225	-1.4642	0.007143	0.209293	1	786	tags=11%, list=8%, signal=12%
GO_DYNEIN_BINDING	GO_DYNEIN_BINDING	18	-0.58654	-1.4627	0.052632	0.208224	1	1706	tags=44%, list=16%, signal=53%
GO_PEPTIDE_N_ACETYLTRANSFERASE_ACTIVITY	GO_PEPTIDE_N_ACETYLTRANSFERASE_ACTIVITY	32	-0.51151	-1.4605	0.044928	0.207907	1	3781	tags=66%, list=36%, signal=103%
GO_MOTOR_ACTIVITY	GO_MOTOR_ACTIVITY	85	-0.43107	-1.4553	0.014599	0.213155	1	1777	tags=29%, list=17%, signal=35%
GO_LIGASE_ACTIVITY	GO_LIGASE_ACTIVITY	234	-0.38851	-1.4547	0.003341	0.21031	1	3922	tags=52%, list=38%, signal=81%
GO_INWARD_RECTIFIER_POTASSIUM_CHANNEL_ACTIVITY	GO_INWARD_RECTIFIER_POTASSIUM_CHANNEL_ACTIVITY	17	-0.583	-1.4543	0.061377	0.207461	1	259	tags=18%, list=2%, signal=18%
GO_GALACTOSYLTRANSFERASE_ACTIVITY	GO_GALACTOSYLTRANSFERASE_ACTIVITY	23	-0.54473	-1.4483	0.043228	0.214623	1	397	tags=17%, list=4%, signal=18%
GO_KINESIN_BINDING	GO_KINESIN_BINDING	23	-0.53781	-1.4449	0.051245	0.21753	1	2954	tags=48%, list=28%, signal=67%

GO_RNA_POLYMERASE_II_REPRESSING_TRANSCRIPTION_FACTOR_BINDING	GO_RNA_POLYMERASE_II_REPRESSING_TRANSCRIPTION_FACTOR_BINDING	19	-0.56213	-1.4446	0.055152	0.214526	1	1915	tags=37%, list=18%, signal=45%
GO_METHYLATED_HISTONE_BINDING	GO_METHYLATED_HISTONE_BINDING	28	-0.52	-1.4435	0.055398	0.212795	1	2278	tags=43%, list=22%, signal=55%
GO_UDP_GALACTOSYLTRANSFERASE_ACTIVITY	GO_UDP_GALACTOSYLTRANSFERASE_ACTIVITY	21	-0.56125	-1.4369	0.044993	0.221227	1	397	tags=19%, list=4%, signal=20%
GO_PURINE_NTP_DEPENDENT_HELICASE_ACTIVITY	GO_PURINE_NTP_DEPENDENT_HELICASE_ACTIVITY	70	-0.43975	-1.4353	0.029777	0.220538	1	3202	tags=47%, list=31%, signal=68%
GO_NAD_BINDING	GO_NAD_BINDING	48	-0.47208	-1.4333	0.024457	0.220589	1	3490	tags=56%, list=34%, signal=84%
GO_N_METHYLTRANSFERASE_ACTIVITY	GO_N_METHYLTRANSFERASE_ACTIVITY	49	-0.4678	-1.4327	0.021828	0.218348	1	2921	tags=51%, list=28%, signal=71%
GO_ATP_DEPENDENT_DNA_HELICASE_ACTIVITY	GO_ATP_DEPENDENT_DNA_HELICASE_ACTIVITY	28	-0.50457	-1.4267	0.070225	0.225978	1	3181	tags=46%, list=31%, signal=67%
GO_PROTEIN_N_TERMINUS_BINDING	GO_PROTEIN_N_TERMINUS_BINDING	89	-0.4206	-1.4253	0.019778	0.225283	1	3505	tags=57%, list=34%, signal=86%
GO_PHOSPHATIDYLCHOLINE_BINDING	GO_PHOSPHATIDYLCHOLINE_BINDING	15	-0.60592	-1.4237	0.053391	0.224752	1	957	tags=27%, list=9%, signal=29%
GO_NUCLEOTIDYLTRANSFERASE_ACTIVITY	GO_NUCLEOTIDYLTRANSFERASE_ACTIVITY	89	-0.42154	-1.4217	0.027194	0.22511	1	3030	tags=46%, list=29%, signal=64%
GO_ENDODEOXYRIBONUCLEASE_ACTIVITY	GO_ENDODEOXYRIBONUCLEASE_ACTIVITY	34	-0.48606	-1.4165	0.049519	0.231627	1	2888	tags=44%, list=28%, signal=61%
GO_CATION_CHANNEL_ACTIVITY	GO_CATION_CHANNEL_ACTIVITY	192	-0.38424	-1.4161	0.004525	0.228947	1	1560	tags=15%, list=15%, signal=17%
GO_VOLTAGE_GATED_CATION_CHANNEL_ACTIVITY	GO_VOLTAGE_GATED_CATION_CHANNEL_ACTIVITY	91	-0.4177	-1.4112	0.024814	0.234842	1	1341	tags=14%, list=13%, signal=16%

GO_CORE_PROMOTER_BINDING	GO_CORE_PROMOTER_BINDING	117	-0.39907	-1.4091	0.015403	0.235649	1	2721	tags=34%, list=26%, signal=46%
GO_MRNA_BINDING	GO_MRNA_BINDING	89	-0.41509	-1.4062	0.027811	0.238375	1	2412	tags=34%, list=23%, signal=44%
GO_CAMP_BINDING	GO_CAMP_BINDING	15	-0.5797	-1.4057	0.070175	0.236138	1	1216	tags=20%, list=12%, signal=23%
GO_CALMODULIN_DEPENDENT_PROTEIN_KINASE_ACTIVITY	GO_CALMODULIN_DEPENDENT_PROTEIN_KINASE_ACTIVITY	22	-0.52556	-1.4013	0.067919	0.24188	1	3175	tags=59%, list=31%, signal=85%
GO_DNA_POLYMERASE_ACTIVITY	GO_DNA_POLYMERASE_ACTIVITY	26	-0.5103	-1.3878	0.075949	0.265256	1	2314	tags=46%, list=22%, signal=59%
GO_RNA_POLYMERASE_II_TRANSCRIPTION_FACTOR_BINDING	GO_RNA_POLYMERASE_II_TRANSCRIPTION_FACTOR_BINDING	84	-0.41312	-1.3862	0.038319	0.264954	1	1976	tags=26%, list=19%, signal=32%
GO_PROTEIN_C_TERMINUS_BINDING	GO_PROTEIN_C_TERMINUS_BINDING	154	-0.38386	-1.3844	0.022472	0.264951	1	2404	tags=32%, list=23%, signal=42%
GO_3_5_EXONUCLEASE_ACTIVITY	GO_3_5_EXONUCLEASE_ACTIVITY	29	-0.50118	-1.383	0.065919	0.263943	1	2893	tags=55%, list=28%, signal=76%
GO_ENDORIBONUCLEASE_ACTIVITY_PRODUCING_5_PHOSPHOMONOESTERS	GO_ENDORIBONUCLEASE_ACTIVITY_PRODUCING_5_PHOSPHOMONOESTERS	20	-0.5301	-1.3818	0.082459	0.262998	1	3750	tags=65%, list=36%, signal=102%
GO_NADH_DEHYDROGENASE_ACTIVITY	GO_NADH_DEHYDROGENASE_ACTIVITY	28	-0.49981	-1.3815	0.070809	0.260446	1	4841	tags=82%, list=47%, signal=153%
GO_P53_BINDING	GO_P53_BINDING	48	-0.45127	-1.3809	0.052279	0.258212	1	2464	tags=35%, list=24%, signal=46%
GO_HYDROLASE_ACTIVITY_ACTING_ON_CARBON_NITROGEN_BUT_NOT_PEPTIDE_BONDS_IN_LINEAR_AMIDES	GO_HYDROLASE_ACTIVITY_ACTING_ON_CARBON_NITROGEN_BUT_NOT_PEPTIDE_BONDS_IN_LINEAR_AMIDES	61	-0.43682	-1.3801	0.051451	0.256681	1	1915	tags=26%, list=18%, signal=32%

GO_ACETYLTRANSFERASE_ACTIVITY	GO_ACETYLTRANSFERASE_ACTIVITY	53	-0.44255	-1.3799	0.063599	0.254035	1	3610	tags=51%, list=35%, signal=78%
GO_ANION_CATION_SYMPORTER_ACTIVITY	GO_ANION_CATION_SYMPORTER_ACTIVITY	44	-0.45418	-1.3799	0.065617	0.251124	1	1405	tags=20%, list=14%, signal=24%
GO_TRANSCRIPTION_FACTOR_ACTIVITY_RNA_POLYMERASE_II_TRANSCRIPTION_FACTOR_BINDING	GO_TRANSCRIPTION_FACTOR_ACTIVITY_RNA_POLYMERASE_II_TRANSCRIPTION_FACTOR_BINDING	88	-0.40958	-1.3771	0.040558	0.253315	1	2545	tags=35%, list=25%, signal=46%
GO_TRANSLATION_REGULATOR_ACTIVITY	GO_TRANSLATION_REGULATOR_ACTIVITY	20	-0.52902	-1.3762	0.072238	0.252016	1	1299	tags=30%, list=13%, signal=34%
GO_TRNA_BINDING	GO_TRNA_BINDING	20	-0.53364	-1.3755	0.090656	0.25041	1	2316	tags=35%, list=22%, signal=45%
GO_TRANSCRIPTION_COREPRESSOR_ACTIVITY	GO_TRANSCRIPTION_COREPRESSOR_ACTIVITY	160	-0.38032	-1.3729	0.015099	0.252572	1	2700	tags=37%, list=26%, signal=49%
GO_PHOSPHOTRANSFERASE_ACTIVITY_PHOSPHATE_GROUP_AS_ACCEPTOR	GO_PHOSPHOTRANSFERASE_ACTIVITY_PHOSPHATE_GROUP_AS_ACCEPTOR	20	-0.53826	-1.3698	0.090258	0.256291	1	2693	tags=45%, list=26%, signal=61%
GO_METAL_ION_TRANSMEMBRANE_TRANSPORTER_ACTIVITY	GO_METAL_ION_TRANSMEMBRANE_TRANSPORTER_ACTIVITY	280	-0.35869	-1.3666	0.010846	0.260075	1	1568	tags=16%, list=15%, signal=18%
GO_HISTONE_LYSINE_N_METHYLTRANSFERASE_ACTIVITY	GO_HISTONE_LYSINE_N_METHYLTRANSFERASE_ACTIVITY	28	-0.49348	-1.3665	0.089855	0.257361	1	2850	tags=46%, list=27%, signal=64%
GO_OXIDOREDUCTASE_ACTIVITY_ACTING_ON_NAD_P_H_QUINONE_OR_SIMILAR_COMPOUND_AS_ACCEPTOR	GO_OXIDOREDUCTASE_ACTIVITY_ACTING_ON_NAD_P_H_QUINONE_OR_SIMILAR_COMPOUND_AS_ACCEPTOR	38	-0.46613	-1.3612	0.083897	0.264631	1	3312	tags=50%, list=32%, signal=73%

GO_PROTEIN_SERINE_THREONINE_PHOSPHATASE_ACTIVITY	GO_PROTEIN_SERINE_THREONINE_PHOSPHATASE_ACTIVITY	43	-0.45183	-1.3596	0.085135	0.264807	1	2558	tags=33%, list=25%, signal=43%
GO_UBIQUITIN_LIKE_PROTEIN_CONJUGATING_ENZYME_ACTIVITY	GO_UBIQUITIN_LIKE_PROTEIN_CONJUGATING_ENZYME_ACTIVITY	21	-0.52594	-1.3551	0.084046	0.270873	1	3843	tags=67%, list=37%, signal=106%
GO_ACTIVATING_TRANSCRIPTION_FACTOR_BINDING	GO_ACTIVATING_TRANSCRIPTION_FACTOR_BINDING	44	-0.44932	-1.3539	0.077446	0.270216	1	728	tags=16%, list=7%, signal=17%
GO_UBIQUITIN_LIKE_PROTEIN_CONJUGATING_ENZYME_BINDING	GO_UBIQUITIN_LIKE_PROTEIN_CONJUGATING_ENZYME_BINDING	18	-0.53699	-1.3484	0.108597	0.278622	1	4599	tags=83%, list=44%, signal=149%
GO_DEACETYLASE_ACTIVITY	GO_DEACETYLASE_ACTIVITY	42	-0.45344	-1.3477	0.076618	0.277396	1	1915	tags=29%, list=18%, signal=35%
GO_PROTEIN_SERINE_THREONINE_KINASE_ACTIVITY	GO_PROTEIN_SERINE_THREONINE_KINASE_ACTIVITY	309	-0.35229	-1.3463	0.009504	0.2771	1	2908	tags=36%, list=28%, signal=48%
GO_DNA_DIRECTED_DNA_POLYMERASE_ACTIVITY	GO_DNA_DIRECTED_DNA_POLYMERASE_ACTIVITY	20	-0.53265	-1.3447	0.091808	0.277442	1	2120	tags=50%, list=20%, signal=63%
GO_METAL_CLUSTER_BINDING	GO_METAL_CLUSTER_BINDING	42	-0.45168	-1.3398	0.078877	0.284378	1	2171	tags=38%, list=21%, signal=48%
GO_EXTRACELLULAR_GLUTAMATE_GATED_ION_CHANNEL_ACTIVITY	GO_EXTRACELLULAR_GLUTAMATE_GATED_ION_CHANNEL_ACTIVITY	16	-0.54752	-1.3288	0.133013	0.30393	1	280	tags=19%, list=3%, signal=19%

GO_OXIDOREDUCTASE_ACTIVITY_ACTING_ON_THE_CH_OH_GROUP_OF_DONORS_NAD_OR_NADP_AS_ACCEPTOR	GO_OXIDOREDUCTASE_ACTIVITY_ACTING_ON_THE_CH_OH_GROUP_OF_DONORS_NAD_OR_NADP_AS_ACCEPTOR	79	-0.39818	-1.3259	0.082487	0.307165	1	3221	tags=37%, list=31%, signal=53%
GO_NADP_BINDING	GO_NADP_BINDING	39	-0.4416	-1.3258	0.093793	0.304444	1	2001	tags=26%, list=19%, signal=32%
GO_L_AMINO_ACID_TRANSMEMBRANE_TRANSPORTER_ACTIVITY	GO_L_AMINO_ACID_TRANSMEMBRANE_TRANSPORTER_ACTIVITY	37	-0.45955	-1.3235	0.092992	0.30634	1	1689	tags=30%, list=16%, signal=35%
GO_GATED_CHANNEL_ACTIVITY	GO_GATED_CHANNEL_ACTIVITY	225	-0.35476	-1.3234	0.025164	0.303719	1	2336	tags=20%, list=22%, signal=26%
GO_TRANSCRIPTION_FACTOR_ACTIVITY_PROTEIN_BINDING	GO_TRANSCRIPTION_FACTOR_ACTIVITY_PROTEIN_BINDING	424	-0.3405	-1.322	0.002101	0.300866	1	2758	tags=34%, list=27%, signal=44%
GO_ION_CHANNEL_BINDING	GO_ION_CHANNEL_BINDING	86	-0.39174	-1.3204	0.054878	0.300971	1	2200	tags=28%, list=21%, signal=35%
